# Nickel-Photocatalytic Deoxygenative Arylation toward β‑Methyl-Branched α‑Amino Acids

**DOI:** 10.1021/acs.orglett.6c00968

**Published:** 2026-04-20

**Authors:** Berjan Stouwie, Anna R. Emmerich, Thomas Weyhermüller, Sebastian B. Beil

**Affiliations:** † 28313 Max-Planck-Institute for Chemical Energy Conversion, Department for Electrosynthesis, Stiftstr. 34−36, 45470 Mülheim an der Ruhr, Germany; ‡ 28313 Max-Planck-Institute for Chemical Energy Conversion, Department of Inorganic Spectroscopy, Stiftstr. 34−36, 45470 Mülheim an der Ruhr, Germany; § Faculty of Chemistry, University of Duisburg-Essen, Universitätsstraße 7, 45177 Essen, Germany

## Abstract

β-Methyl-branched α-amino acids are important motifs in peptide therapeutics. However, only few methods on their synthesis have been reported to date. Herein we report an efficient, one-step synthesis of these β-Me-branched α-amino acids through the deoxygenative arylation of threonine, yielding a diverse scope of noncanonical α-amino acids with good functional group tolerance. Our method could also be extended to the deoxygenative arylation of 3-hydroxyproline, giving access to various therapeutically relevant arylated amino acids.

## Graphical abstract

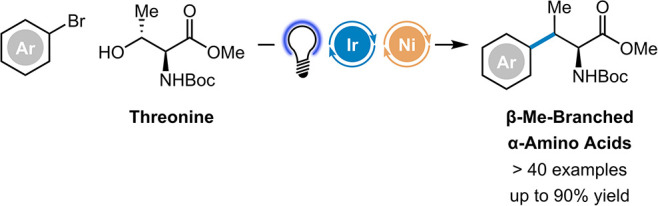

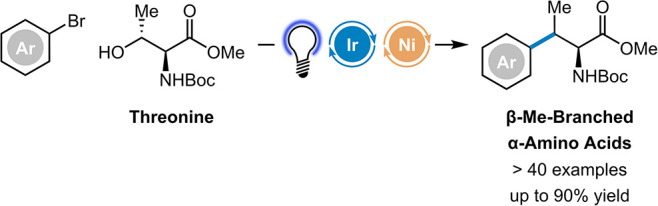

Peptide therapeutics have seen a rise in attention and importance in the last 20 years, and it is expected that the significance of this class of therapeutics will continue to grow.^1^ Nevertheless, most approved peptide drugs are based on natural peptides and have poor in vivo stability and bioavailability due to their linearity and high content of canonical amino acids (cAAs).^2^ Nevertheless, pharmaceutically active peptides can be improved by incorporating noncanonical amino acids (ncAAs), giving access to an almost infinite chemical space and allowing the precise fine-tuning of the metabolic stability and bioavailability.^3^

Especially the incorporation of β-Me-branched α-amino acids (β-Me-AAs) into peptide therapeutics is of high interest since these amino acids increase binding affinity due to a decreased conformational freedom within the peptide backbone and increased stability toward proteases.^2,4^ Several drugs and active natural products containing these motives have already been developed or discovered (Figure 1). The Somatostatin agonist L-779976 contains a β-Me-tryptophan moiety, which greatly increases its potency.^5^ It was also shown that β-methylation of the δ-opioid receptor antagonist TIPP greatly altered its receptor selectivity and biological properties.^6^ Similar effects were observed for an analog of the peptide hormone Angiotensin IV.^7^ Besides their incorporation in peptide therapeutics, β-Me-AAs are also found in small molecule drugs, such as the ribonucleotide reductase inhibitor TAS1553.^8^ Finally, β-Me-AAs are present in naturally active compounds, such as the narrow-spectrum antibiotic Hormaomycin, produced by *Streptomyces griseoflavus*.^9^ Besides these β-Me-AAs, there are drugs containing other β-alkylated amino acids like the Melanocortin-4 receptor ligands, containing a β-substituted proline motif.^10^

**Figure 1:**
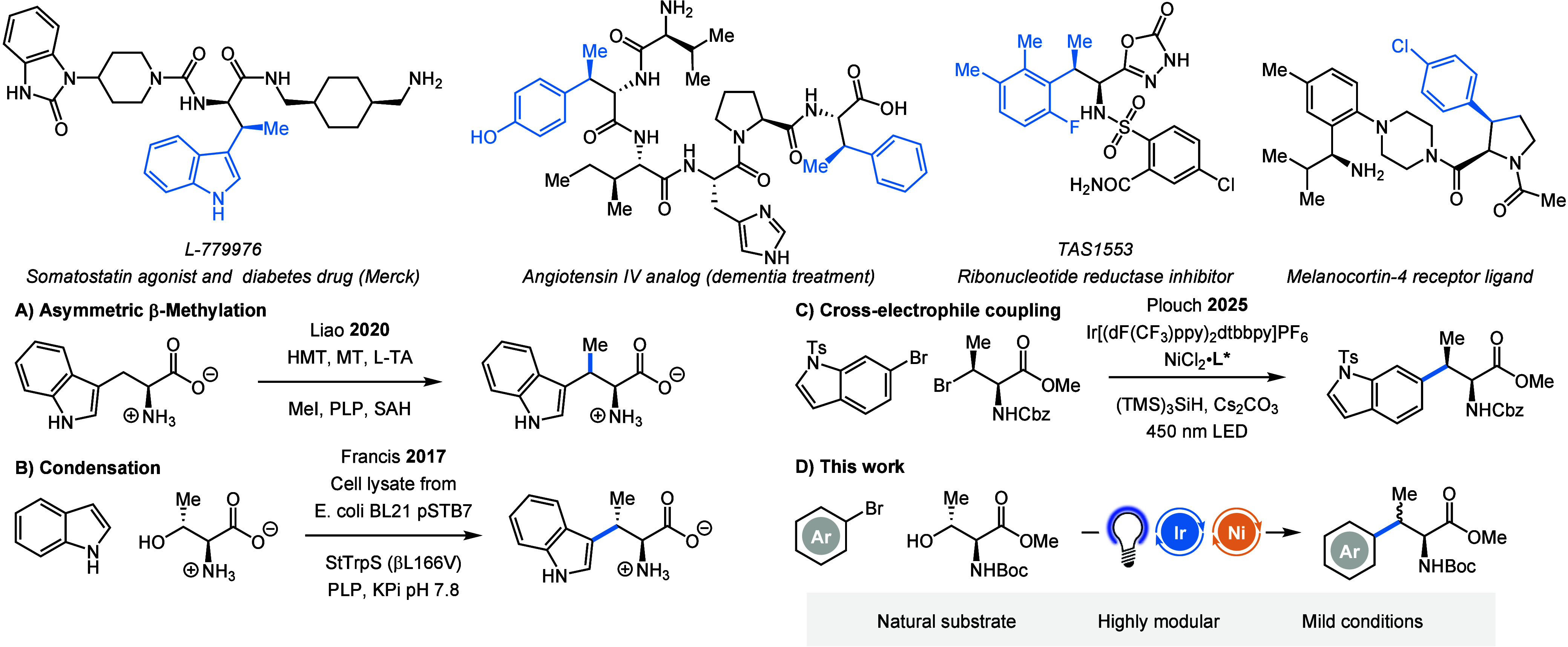
Examples of therapeutics and active natural compounds containing β-Me-AAs (top) and selected examples for their synthesis (bottom). (Bio)­catalytic access to β-Me-branched α-amino acids (A,B), photochemical cross-electrophile coupling (C), and our photocatalytic approach from threonine (D).

Despite their promising use in (peptide) therapeutics, the synthesis of these β-Me-branched ncAAs is highly challenging, and only few methods have been described to date. For example, Roff et al. reported the synthesis of β-Me-AAs through asymmetric hydrogenation of dehydroamino acids.^11^ Even though good enantiomeric excess could be obtained, this method is a 4-step synthesis starting from threonine and does not tolerate functional groups that are susceptible to hydrogenation. Most other reported syntheses are biochemical pathways, using enzymatic transformations. For instance, Sun et al. achieved the synthesis of β-Me-branched aromatic α-amino acids through the direct asymmetric amination of unsaturated acids.^12^ Similarly, Li et al. and Dunham et al. both reported an enzymatic transamination of racemic α-ketoacid.^13^ Although for these methods good *d.r.* and *e.e.* were obtained, they show low modularity due to a multistep synthesis of the starting materials, and no tolerance to heterocycles was reported for Sun et al. and Li et al. Dunham et al. reported good yields for heterocycles; however, they use high molecular weight amine donors and long reaction times. In a different approach, Liao et al. employed an enzymatic asymmetric β-methylation (Figure 1A).^14^ Although good *e.e.* were obtained, they used toxic methyl iodide, required multiple enzymes to work cooperatively, and only reported one example of an aromatic side group. Additionally, Francis et al. reported on the synthesis of β-methyltryptophan through the condensation of threonine and indole (Figure 1B).^15^ Even though this method starts from a natural amino acid and is a one-step synthesis, it only applies to the synthesis of β-methyltryptophans. Recently, Plouch et al. reported the diastereoselective synthesis of β-Me amino acids, through a cross-electrophile coupling of the β-bromo amino acid derived from threonine with bromo-indoles (Figure 1C). Although good *d.r.* were observed, they use an unnatural amino acid as starting material. Therefore, we were interested in developing a more modular bio-orthogonal method for the synthesis of these β-Me-AAs in a single step from natural and abundant threonine as starting material, already featuring the β-Me moiety (Figure 1D).

We evaluated various methods known for deoxygenative cross-coupling reactions. However, both a phosphine-enabled C–O bond activation and deoxygenation of the respective xanthate salt did not yield any arylated product (see SI for details).^16^ Converting the alcohol to the oxalate, followed by a double decarboxylation, yielded the arylated product, but in low yields.^17^ Therefore, we focused on the NHC-enabled deoxygenative arylation pioneered by the MacMillan group.^18^ Generally, the high bond-dissociation energy of the C­(*sp* ^3^)–OH bond poses a great challenge for deoxygenative arylation reactions. However, by employing redox-active adducts of alcohols with *N*-heterocyclic carbenes (NHCs) single electron transfer oxidation from an excited state photocatalyst becomes feasible, forming an alkyl radical after β-scission (a plausible mechanism is provided in the SI).^19^ Using nickel catalysis, cross-coupling of this alkyl radical with an aryl halide is achieved.

Starting from literature conditions,^18^ we set out to optimize the cross-coupling of 2-bromo-5-(trifluoromethyl)­pyridine (**1**) with Boc-Thr-OMe (**2**) (see SI for full optimization). A solvent screening for the cross-coupling step (Table 1, entries 1–3) revealed that changing from DMA to acetone increases the yield to 49%. However, using acetone for the alcohol activation step as well yielded no product (Table 1, entry 4). Increasing the reaction time for the condensation of **2** with the NHC from 10 min to 2 h greatly increased the yield, with only a minor further increase in yield observed for a 4 h condensation (Table 1, entries 5 and 6).

**Table 1:**
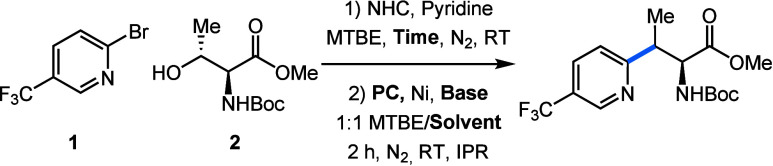
Summarized Optimization of the Deoxygenative Cross-Coupling of 2-Bromo-5-(trifluoromethyl)­pyridine (**1**) with Boc-Thr-OMe (**2**)

Entry	Solvent	Base	PC	Time	Yield^b^	*d.r.* ^b^
**1**	DMA	Quin.	Ir-1	10 min	36%	2.1:1
**2**	MeCN	Quin.	Ir-1	10 min	11%	1.9:1
**3**	Acetone	Quin.	Ir-1	10 min	49%	2.1:1
**4** ^c^	Acetone	Quin.	Ir-1	10 min	0%	-
**5**	Acetone	Quin.	Ir-1	2 h	70%	2.1:1
**6**	Acetone	Quin.	Ir-1	4 h	73%	2.1:1
**7**	Acetone	KOAc	Ir-1	2 h	83%	2.5:1
**8**	Acetone	KOAc	Ir-2	2 h	94%	2.8:1
**9**	Acetone	KOAc	4CzIPN	2 h	67%	2.3:1

a Reaction conditions: **1** (0.1 mmol); **2** (1.7 equiv); NHC (1.6 equiv); pyridine (1.6 equiv); MTBE (1 mL); PC (1.5 mol %); NiBr_2_·dtbbpy (5 mol %); base (1.75 equiv); solvent (1 mL); IPR: 450 nm (100%), 1000 rpm, 6800 rpm fan.

b ^19^F NMR yields of cross-coupling product vs benzotrifluoride as an internal standard in CDCl_3_.

c Acetone was used instead of MTBE in the first step. IPR: integrated photoreactor; Quin.: quinuclidine; Ir-1: Ir­(ppy)_2_(dtbbpy)­PF_6_, Ir-2: Ir­(F­(Me)­ppy)_2_(dtbbpy)­PF_6_; 4CzIPN: 1,2,3,5-tetrakis­(carbazol-9-yl)-4,6-dicyanobenzene.

Next, we screened multiple bases and found that KOAc instead of quinuclidine increases the yield, with a concomitant small increase in *d.r.* (Table 1, entry 7). Changing from Ir­(ppy)_2_(dtbbpy)­PF_6_ to Ir­(F­(Me)­ppy)_2_(dtbbpy)­PF_6_ boosted the yield even further to a final 94% (Table 1, entry 8). The organic photocatalyst 4CzIPN performed much worse, giving the cross-coupling product in only 67% yield (Table 1, entry 9). Despite numerous efforts and extensive screening of chiral ligands, we were not able to increase the diastereoselectivity of the reaction (see the SI for details). In many cases, the diastereomers could be separated by reversed-phase column chromatography, giving access to both products simultaneously.

Control experiments revealed that both the nickel and iridium catalyst are required (SI, Table S2, entries 2 and 3). Additionally, without base or light irradiation no product could be detected (SI, Table S2, entries 4 and 5). Omitting the degassing step prior to irradiation greatly reduced the yield to 39%, highlighting the harmful effect of oxygen on the yield (SI, Table S2, entry 6). Running the reaction without condensation of threonine with the NHC resulted in no arylation product, and instead the C–O coupling product could be detected by LCMS (SI, Table S2, entry 7). Neither aryl chlorides nor iodides were amenable under these conditions (see SI). Kinetic analysis exhibited fast performance, and the reaction was completely inhibited when TEMPO as a radical scavenger was added (see SI).

With the optimized reaction conditions in hand, we set out to explore the scope of applicable aryl halides. After assessing various aryl bromides, we noticed much poorer yields compared to the ones obtained in our optimization campaign. A small screening revealed that the yields could be increased by changing back from KOAc to quinuclidine as base (Scheme 1). Using bromobenzene yielded β-methylphenylalanine (**3**) in good yield. Both electron-donating groups (**4–7**) as well as various electron-withdrawing groups (**8–14**) were all tolerated, giving isolated yields ranging from 62% to 90%. We were happy to see that even an unprotected phenol (**15**) is tolerated, although showing a drop in yield. Additionally, an amide (**16**), boronic acid ester (**17**), and propargylic ether (**18**) are compatible with our reaction conditions, allowing for further modifications through either a Suzuki-coupling or alkyne–azide click reaction.^20^ Sadly, azides as starting materials were incompatible under these conditions (see SI for details). We were happy to see that even an unprotected pyrazole (**19**) is tolerated. Finally, 2-bromonaphthalene was used as a cross-coupling partner, giving the β-Me-naphthylalanine (**20**) in a good yield (80%). Besides *para*-substituted aryl bromides, this method also tolerates *ortho*-substituted aryl bromides (**21**) although with lower yield, as well as *meta*-substituted aryl halides (**22**). Both *N*-phthalimide (**23**) and *N*-tosyl (**24**) protected threonine yielded the product, although with significantly reduced yield. We also tried 1,4-dibromobenzene, which gave the 2-fold-coupled product (**25**). To test the applicability of this method to more complex substrates, we tested the deoxygenative cross-coupling of the dipeptide Boc-Thr-Ala-OMe. This gave the arylated product (**26**) in moderate yield, highlighting the possibility to functionalize (di)­peptides. Next, we explored the coupling of heteroaryl bromides. 3-Bromopyridine (**27**) is well tolerated, with a drop in yield observed for 3-bromo-2-methylpyridine (**28**) attributed to steric effects. 2-Bromopyridines (**29** and **30**) showed lower yields, although 2-bromo-5-(trifluoromethyl)­pyridine (**31**) gave the product in good yield. Crystallization of the minor diastereomer of this product revealed that the configuration of this diastereomer is (*S*,*S*) (see SI). We hypothesize that the major diastereomer therefore has the *R*-configuration at the β-carbon and the *S*-configuration at the α-carbon, the later remaining untouched throughout the reaction.

**Scheme 1:**
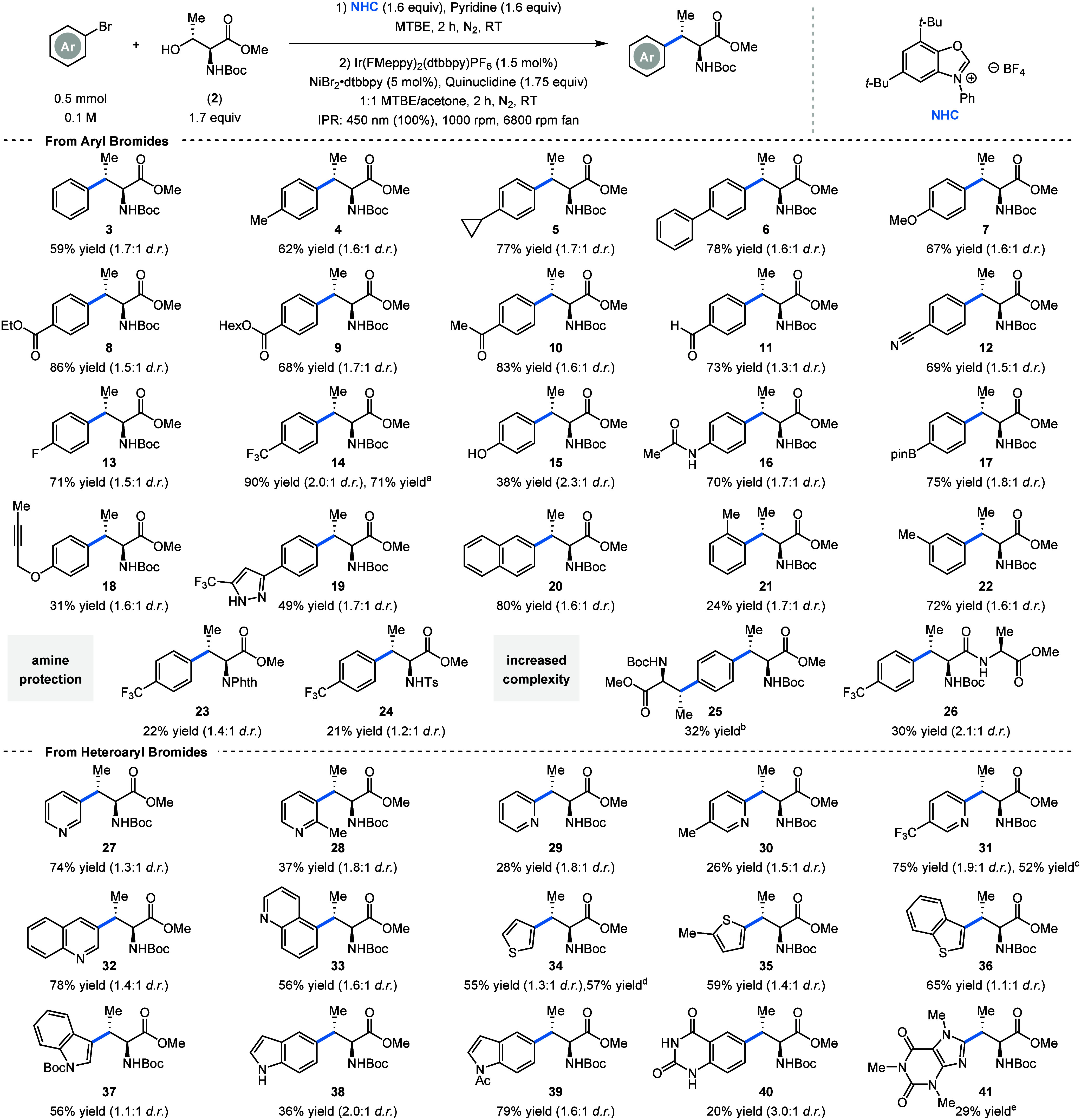
Scope of the Deoxygenative Arylation of Boc-Thr-OMe and Isolated Yields^f^

a 1.0 mmol scale.
b 0.25 mmol of 1,4-dibromobenzene.
c 1.5 mmol scale.
d 7.5 mol % NiBr_2_·dtbbpy, 22 mol % phthalimide.
e Only one diastereomer was isolated.
f Major diastereomer shown, and diastereomeric ratios are given in parentheses.

Additionally, quinolines (**32** and **33**) are tolerated, yielding the arylated product in up to 78% yield. Both 2- and 3-bromothiophenes (**34** and **35**) as well as benzothiophene (**36**) are tolerated. Using Boc-protected 3-bromoindole yielded the β-Me-tryptophan (**37**) in moderate yield. Likewise, 5-bromoindole can be used to yield β-Me-isotryptophan **38** in moderate yield, which increased to 79% with acetylation of the indole moiety (**39**). Finally, 6-bromoquinazoline-2,4­(1*H*,3*H*)-dione (**40**) and 8-bromocoffeine (**41**) yielded the heterocyclic products in low but synthetically acceptable yields.

To investigate the broader applicability of this method for the synthesis of (pharmaceutically) interesting ncAAs, we explored the arylation of 3-hydroxyproline. Proline is unique among the natural amino acids as it forms tertiary amides, preventing the formation of hydrogen bonds. Nevertheless, proline is able to stabilize many secondary structures, like turns and helices, and reduces the conformational freedom.^21^ β-Substitution of proline can further modulate these structures.^22^ β-Phenyl-proline for example combines the conformational properties of proline with that of an aromatic side chain, resulting in a rigid peptide backbone.^23^ Incorporating these amino acids in therapeutic peptides leads to significantly increased binding affinity and activity, demonstrated in an analogue of Substance P.^24^ Additionally, these moieties can be incorporated in small molecule drugs, as was demonstrated for a Rho-kinase inhibitor.^10,25^

We were delighted to see that using the same conditions as those for the deoxygenative arylation of threonine we were able to achieve deoxygenative arylation of protected 3-hydroxyproline (Boc-3-Hyp-OMe) in good yields (**42**–**46**) (Scheme 2). Arylated product **46** can be used as a precursor to a melanocortin-4 receptor ligand.^10^ Gratifyingly, all products were isolated as the *anti*-diastereomer selectively, most likely due to the more rigid cyclic structure.

**Scheme 2:**
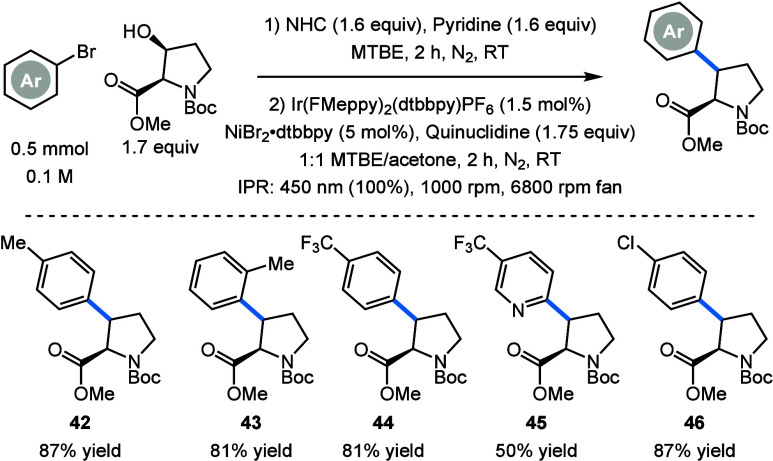
Scope of the Deoxygenative Arylation of Boc-3-Hyp-OMe

In conclusion, we report a novel synthetic route toward β-Me-branched α-amino acids through an *N*-heterocyclic carbene-enabled metallaphotoredox-catalyzed deoxygenative arylation. Starting from readily available threonine and 3-hydroxy proline amino acids, a broad scope of therapeutically relevant β-Me and β-alkyl-branched α-amino acids were synthesized. Despite numerous attempts, no improvement in diastereoselectivity was achieved. In the future, we aim to expand the concept of natural to noncanonical amino acids to democratize these motifs for peptide therapeutic developments.

## Supplementary Material



## Data Availability

The data underlying this study are openly available in Edmond at 10.17617/3.9REXV0.
