# Supplementary material for: Nickel-Photocatalytic Deoxygenative Arylation toward β‑Methyl-Branched α‑Amino Acids
Source: Org Lett. 2026 Apr 20;28(17):5383–8. doi: 10.1021/acs.orglett.6c00968 (PMC13140145; doi:10.1021/acs.orglett.6c00968)
Supplement: Supplementary file 1 [file ol6c00968_si_001.pdf]

# Supplementary Information

for

# Nickel-Photocatalytic Deoxygenative Arylation toward β-Methyl-branched α-Amino Acids

Berjan Stouwie,[a] Anna R. Emmerich,[a] Thomas Weyhermüller,[b] Sebastian B. Beil [a,c]*

[a] Max-Planck-Institute for Chemical Energy Conversion, Department for Electrosynthesis, Stiftstr. 34-36, 45470 Mülheim an der Ruhr, Germany, sebastian.beil@cec.mpg.de

[b] Max-Planck-Institute for Chemical Energy Conversion, Department of Inorganic Spectroscopy, Stiftstr. 34–36, 45470 Mülheim an der Ruhr, Germany.

[c] Faculty of Chemistry, University of Duisburg-Essen, Universitätsstraße 7, 45177 Essen, Germany.

## Table of Contents


1. General Remarks ..... S5

Flash Chromatography ..... S5

High Resolution Mass Spectrometry ..... S5

Gas Chromatography ..... S5

Nuclear Magnetic Resonance (NMR) Spectroscopy ..... S6

Ultra-performance liquid chromatography – mass spectrometry (UPLC-MS) ..... S6

X-ray Crystallography ..... S6

Photochemical Setup ..... S6

2. Reaction Optimization ..... S7
3. Unsuccessful Aryl Halide Substrates ..... S19
4. Use of Aryl Chloride and Aryl Iodide Substrates ..... S20
5. Control Experiments ..... S21
6. Kinetic Experiments ..... S22
7. Mechanistic Experiments ..... S23
8. Synthesis ..... S24

2,4-Di-*tert*-butyl-6-(phenylamino)phenol ..... S24

5,7-Di-*tert*-butyl-3-phenylbenzo[*d*]oxazol-3-ium tetrafluoroborate (NHC-1) ..... S24

2,4-Di-*tert*-butyl-6-(3,5-dimethylanilino)phenol ..... S25

2,4-Di-*tert*-butyl-6-(4-chloroanilino)phenol ..... S25

2,4-Di-*tert*-butyl-6-(4-methoxyanilino)phenol ..... S25

2,4-Di-*tert*-butyl-6-(4-trifluoromethoxyanilino)phenol ..... S26

2,4-Di-*tert*-butyl-6-(3,4-dichloroanilino)phenol ..... S26

2,4-Di-*tert*-butyl-6-(2-fluoroanilino)phenol ..... S26

5,7-Di-*tert*-butyl-3-(3,5-dimethylphenyl)benzo[*d*]oxazol-3-ium tetrafluoroborate (NHC-2) ..... S27

5,7-Di-*tert*-butyl-3-(4-chlorophenyl)benzo[*d*]oxazol-3-ium tetrafluoroborate (NHC-3) ..... S27

5,7-Di-*tert*-butyl-3-(4-methoxyphenyl)benzo[*d*]oxazol-3-ium tetrafluoroborate (NHC-4) ..... S28

5,7-Di-*tert*-butyl-3-(4-(trifluoromethoxy)phenyl)benzo[*d*]oxazol-3-ium tetrafluoroborate (NHC-5) ..... S28

5,7-Di-*tert*-butyl-3-(3,4-dichlorophenyl)benzo[*d*]oxazol-3-ium tetrafluoroborate (NHC-6) ..... S28

5,7-Di-*tert*-butyl-3-(2-fluorophenyl)benzo[*d*]oxazol-3-ium tetrafluoroborate (NHC-7) ..... S29

1-Bromo-4-(but-2-yn-1-yloxy)benzene ..... S29

Hexyl 4-bromobenzoate ..... S30

1-Acetyl-5-bromoindole ............ S30
4-Bromobenzyl azide ............ S30
1-Azido-4-bromobenzene ............ S31
4-Bromoazobenzene ............ S31
2-(4-Fluorophenyl)-5-methylpyridine ............ S31
$[Ir(F(Me)ppy)_2Cl]_2$ ............ S31
$Ir(F(Me)ppy)_2(dtbbpy)PF_6$ ............ S32
$NiBr_2$•dtbbpy ............ S32
Boc-Thr-Ala-OMe ............ S33
Tos-Thr-OMe ............ S33
Phth-Thr-OMe ............ S34
Boc-3-Hyp-OMe ............ S34
9. General procedure ............ S35
Procedure A for the Deoxygenative Arylation of Boc-Thr-OMe ............ S35
Procedure B for the Deoxygenative Arylation of Boc-3-Hyp-OMe ............ S35
10. Synthesis and Characterization of $\beta$-Me-Branched $\alpha$-Amino Acids ............ S36
Methyl 2-((*tert*-butoxycarbonyl)amino)-3-phenylbutanoate (3) ............ S36
Methyl 2-((*tert*-butoxycarbonyl)amino)-3-(4-methylphenyl)butanoate (4) ............ S36
Methyl 2-((*tert*-butoxycarbonyl)amino)-3-(4-cyclopropylphenyl)butanoate (5) ............ S36
Methyl 3-([1,1'-biphenyl]-4-yl)-2-((*tert*-butoxycarbonyl)amino)butanoate (6) ............ S37
Methyl 2-((*tert*-butoxycarbonyl)amino)-3-(4-methoxyphenyl)butanoate (7) ............ S37
Ethyl 4-(3-((*tert*-butoxycarbonyl)amino)-4-methoxy-4-oxobutan-2-yl)benzoate (8) ............ S38
Hexyl 4-(3-((*tert*-butoxycarbonyl)amino)-4-methoxy-4-oxobutan-2-yl)benzoate (9) ............ S38
Methyl 3-(4-acetylphenyl)-2-((*tert*-butoxycarbonyl)amino)butanoate (10) ............ S39
Methyl 2-((*tert*-butoxycarbonyl)amino)-3-(4-formylphenyl)butanoate (11) ............ S39
Methyl 2-((*tert*-butoxycarbonyl)amino)-3-(4-cyanophenyl)butanoate (12) ............ S39
Methyl 2-((*tert*-butoxycarbonyl)amino)-3-(4-fluorophenyl)butanoate (13) ............ S40
Methyl 2-((*tert*-butoxycarbonyl)amino)-3-(4-(trifluoromethyl)phenyl)butanoate (14) ............ S40
Methyl 2-((*tert*-butoxycarbonyl)amino)-3-(4-hydroxyphenyl)butanoate (15) ............ S41
Methyl 3-(4-acetamidophenyl)-2-((*tert*-butoxycarbonyl)amino)butanoate (16) ............ S41
Methyl 2-((*tert*-butoxycarbonyl)amino)-3-(4-(4,4,5,5-tetramethyl-1,3,2-dioxaborolan-2-yl)phenyl)butanoate (17) ............ S42
Methyl 3-(4-(but-2-yn-1-yloxy)phenyl)-2-((*tert*-butoxycarbonyl)amino)butanoate (18) ............ S42

Methyl 2-((*tert*-butoxycarbonyl)amino)-3-(4-(5-(trifluoromethyl)-1H-pyrazol-3-yl)phenyl)butanoate (19) ........ S43
Methyl 2-((*tert*-butoxycarbonyl)amino)-3-(naphthalen-2-yl)butanoate (20) ........ S43
Methyl 2-((*tert*-butoxycarbonyl)amino)-3-(2-methylphenyl)butanoate (21) ........ S44
Methyl 2-((*tert*-butoxycarbonyl)amino)-3-(3-methylphenyl)butanoate (22) ........ S44
Methyl 2-(1,3-dioxoisoindolin-2-yl)-3-(4-(trifluoromethyl)phenyl)butanoate (23) ........ S44
Methyl 2-((4-methylphenyl)sulfonamido)-3-(4-(trifluoromethyl)phenyl)butanoate (24) ........ S45
Dimethyl 3,3'-(1,4-phenylene)-bis(2-((*tert*-butoxycarbonyl)amino)butanoate) (25) ........ S46
Methyl 2-((*tert*-butoxycarbonyl)amino)-3-(4-(trifluoromethyl)phenyl)butanoyl)-L-alaninate (26) . S46
Methyl 2-((*tert*-butoxycarbonyl)amino)-3-(pyridin-3-yl)butanoate (27) ........ S46
Methyl 2-((*tert*-butoxycarbonyl)amino)-3-(2-methylpyridin-3-yl)butanoate (28) ........ S47
Methyl 2-((*tert*-butoxycarbonyl)amino)-3-(pyridin-2-yl)butanoate (29) ........ S47
Methyl 2-((*tert*-butoxycarbonyl)amino)-3-(5-methylpyridin-2-yl)butanoate (30) ........ S48
Methyl 2-((*tert*-butoxycarbonyl)amino)-3-(5-(trifluoromethyl)pyridin-2-yl)butanoate (31) ........ S49
Methyl 2-((*tert*-butoxycarbonyl)amino)-3-(quinolin-3-yl)butanoate (32) ........ S49
Methyl 2-((*tert*-butoxycarbonyl)amino)-3-(quinolin-5-yl)butanoate (33) ........ S50
Methyl 2-((*tert*-butoxycarbonyl)amino)-3-(thiophen-3-yl)butanoate (34) ........ S50
Methyl 2-((*tert*-butoxycarbonyl)amino)-3-(5-methylthiophen-2-yl)butanoate (35) ........ S51
Methyl 3-(benzo[b]thiophen-3-yl)-2-((*tert*-butoxycarbonyl)amino)butanoate (36) ........ S51
Tert-butyl 3-(3-((*tert*-butoxycarbonyl)amino)-4-methoxy-4-oxobutan-2-yl)-1*H*-indole-1-carboxylate (37) ........ S52
Methyl 2-((*tert*-butoxycarbonyl)amino)-3-(1*H*-indol-5-yl)butanoate (38) ........ S53
Methyl 3-(1-acetyl-1*H*-indol-5-yl)-2-((*tert*-butoxycarbonyl)amino)butanoate (39) ........ S53
Methyl 2-((*tert*-butoxycarbonyl)amino)-3-(2,4-dioxo-1,2,3,4-tetrahydroquinazolin-6-yl)butanoate (40) ........ S54
Methyl 2-((*tert*-butoxycarbonyl)amino)-3-(1,3,7-trimethyl-2,6-dioxo-2,3,6,7-tetrahydro-1*H*-purin-8-yl)butanoate (41) ........ S54
1-(*tert*-butyl) 2-methyl 3-(4-methylphenyl)pyrrolidine-1,2-dicarboxylate (42) ........ S54
1-(*tert*-butyl) 2-methyl 3-(2-methylphenyl)pyrrolidine-1,2-dicarboxylate (43) ........ S55
1-(*tert*-butyl) 2-methyl 3-(4-(trifluoromethyl)phenyl)pyrrolidine-1,2-dicarboxylate (44) ........ S55
1-(*tert*-butyl) 2-methyl 3-(5-(trifluoromethyl)pyridin-2-yl)pyrrolidine-1,2-dicarboxylate (45) ........ S56
1-(*tert*-butyl) 2-methyl -3-(4-chlorophenyl)pyrrolidine-1,2-dicarboxylate (46) ........ S56
11. Bibliography ........ S58
12. Crystallographic Data ........ S59

13. NMR Spectra ........................................................................................................................ S61

# 1. General Remarks

All chemicals and solvents were purchased from commercial suppliers and used without further purification unless specified. Photocatalysts were previously synthesized in the group or in this work if described in the experimental section.[1]

## Flash Chromatography

Preparative column chromatography was performed on prepacked puriFlash™ silica columns (15 µm or 30 µm, PF-15SIHP-F0012, PF-15SIHP-F0025, PF-25SIHC-F0025, PF-15SIHP-F0040, PF-25SIHC-F0120 Interchim, Montlucon Cedex, France) using a puriFlash™-System (puriFlash™ XS520Plus, Interchim, Montlucon Cedex, France) with an integrated UV detector.

Reversed phase column chromatography was performed with a prepacked Sepacore™ C18 column (Büchi Labortechnik GmbH, Essen, Germany), using a preparative chromatography system (Büchi Labortechnik GmbH, Essen, Germany) with a Büchi Control Unit C-620, an UV detector Büchi UV photometer C-635, a Büchi fraction collector C-660 and two Pump Modules C-605 for adjusting the solvent mixtures or a puriFlash™-System (puriFlash™ XS520Plus, Interchim, Montlucon Cedex, France) with an integrated UV detector. Mixtures of water (MilliQ™) and acetonitrile were used as eluents.

## High Resolution Mass Spectrometry

High resolution mass spectra were recorded using a Q Exactive Plus (Thermo Fischer Scientific, San Jose, CA, USA), or Q Exactive GC Orbitrap with Trace 1310 GC (Thermo Fischer Scientific, San Jose, CA, USA) spectrometer. For the GC-MS a 30 m long Zebron ZB-1 column (inner diameter: 250 µm; film thickness 0.25 µm) was used. The temperature started at 35 °C and was heated to 320 °C with a temperature rise of 15 °C/min. Ammonia was used for chemical ionization (CI).

## Gas Chromatography

Analysis of crude reaction mixtures and purified products were performed using a GC-2030 (Shimadzu, Kyoto, Japan) equipped with a flame ionization detector (FID) and a quartz capillary column HI-5 MS (Avantor VWR, Radnor, USA) with following specification: length of 30 m, inner diameter of 0.25 mm and a stationary phase ((5%-phenyl)dimethylsiloxane) of 0.25 µm thickness. Hydrogen was used as carrier gas with a constant velocity of 40 cm/s. Measurements were performed at an injector temperature of 270 °C and a detector temperature of 320 °C, starting at 50 °C (holding for 1 min) and heating to 300 °C (holding for 4.71 min) with a temperature ramp of 17.5 °C/min (method: 2_medium, total program time: 20.0 min).

GC-MS analysis was performed on a GCMS-QP2010SE (Shimadzu, Kyoto, Japan) equipped with an electron ionisation (EI) source and a quadrupole mass analyser. HI-5MS quartz capillary column (Avantor VWR, Radnor, USA) with a length of 30 m, 0.25 mm inner diameter and a stationary phase ((5% phenyl)dimethylsiloxane) of 0.25 µm thickness was used. Helium is the carrier gas at a constant velocity of 30 cm/s. The temperature started at 50 °C (held for 1 min) and was heated to 300 °C (held for 4.71 min) with a temperature rise of 17.5 °C/min.

## Nuclear Magnetic Resonance (NMR) Spectroscopy

NMR spectra were recorded at 25 °C on a Bruker AVANCE III HD 500 or a Bruker Ascend Evo 400 NMR spectrometer, respectively with a Bruker Prodigy probe 80 K or a Bruker iProbe BBFO (Bruker BioSpin GmbH, Rheinstetten, Germany) using $CDCl_3$, $CD_3CN$, acetone-$d_6$ or DMSO-$d_6$ as deuterated solvent. All chemical shifts are reported in δ-scale as parts per million [ppm] (multiplicity, coupling constant J, number of protons), relative to the solvent residual peaks as the internal standard. Coupling constants J are given in Hertz [Hz]. Besides $^1H$, $^{13}C$ and $^{19}F$ experiments, the 2D techniques $^1H$,$^1H$-COSY, $^1H$,$^{13}C$-HSQC and $^1H$,$^{13}C$-HMBC were used assisting to assign the signals. The following abbreviations were used to describe the signals: s (singlet), d (doublet), t (triplet), q (quartet), pent (pentet), sext (sextet), hept (heptet), m (multiplet), br (broad signal). The spectra obtained were evaluated with MestReNova 14 (Mestrelab Research S.L., Spain).

## Ultra-performance liquid chromatography – mass spectrometry (UPLC-MS)

Ultra performance liquid chromatography – mass spectrometry (UPLC-MS) was performed on a Waters™ ACQUITY™ UPLC™ H-Class PLUS System (Waters Corporation, Milford, USA) using a quaternary solvent manager (ACQ H-CLASS QSM PLUS), a sample manager with flow-through needle (ACQ H-Class FTN-H PLUS) design, a column heater (ACQUITY UPLC CM-A) and an ACQUITY UPLC® BEH C18 1.7 µm 2.1 x 50 mm column. Mass spectra were measured using a single quadrupole mass detection (ACQUITY QDa Detector) employing ESI+. Acetonitrile (HPLC-MS grade) and water (Milli-Q®) were used as eluents, eluent with 0.1% (v/v) formic acid was added directly before mass detection using a second isocratic solvent manager (Waters™ ACQ Isoc Solvent Mgr).

## X-ray Crystallography

Single crystal measurements were carried out on a Bruker D8 Venture KAPPA diffractometer equipped with INCOATEC multilayer mirrors, Mo-DIAMOND and Cu-mircofocus sealed tube sources and Oxford Cryostream 800 temperature control.

## Photochemical Setup

All photochemical reactions were performed in an integrated photoreactor PennPhD M2 from Sigma Aldrich or Acceled. As light source 450 nm LED plates was used at 100% intensity. Stirring was kept at 1000 rpm with maximum fan speed of 6800 rpm.

NMR spectra and HRMS data are available at the Edmond repository, DOI: https://doi.org/10.17617/3.9REXV0

## 2. Reaction Optimization

Initially, we started by screening multiple methods for the deoxygenative cross-coupling of Boc-Thr-OMe with 2-bromo-5-(trifluoromethyl)pyridine. Fang *et al.* reported the activation of benzylic alcohols with triphenylphosphine.[2] The deoxygenated alkyl radical could undergo nickel-catalyzed cross coupling with benzothiazolyl bromides. Using their method we did not observe the cross-coupled product (Scheme S1).

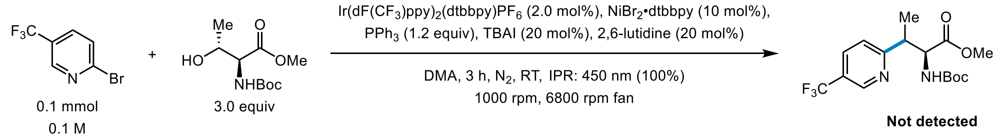


**Scheme S1. Deoxygenative arylation of Boc-Thr-OMe through phosphine activation.**

Additionally, Guo *et al.* reported the activation of alcohols through conversion to their respective xanthate salt.[3] Single electron transfer from an exited state photocatalyst produces the xanthate radical, that couples with the triphenylphosphine to yield the phosphoranyl radical. Double β-scission then affords deoxygenated alkyl radicals, that could undergo Giese addition. Using a similar approach, we tried to achieve nickel-catalyzed cross-coupling but no product could be detected, possibly due to poisoning of the nickel catalyst (Scheme S2).

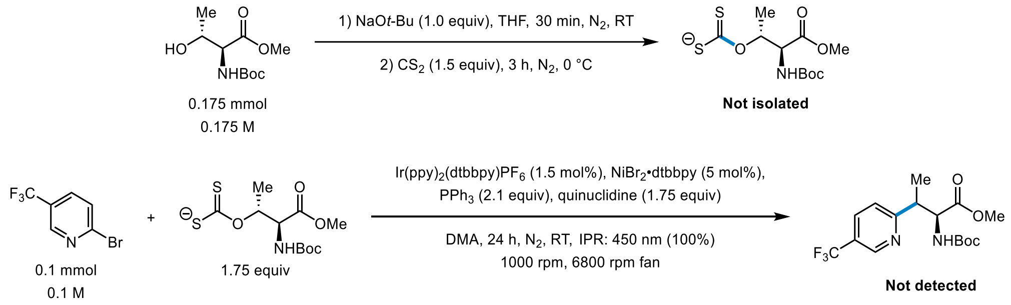


**Scheme S2. Deoxygenative arylation of Boc-Thr-OMe through xanthate formation.**

Lastly, we tried activation of the alcohol by converting it to its corresponding oxalate, based on the work of Zhang *et al.*[4] Double decarboxylation of the oxidized oxalate generates a carbon-centered radical that can engage in nickel-catalyzed cross-coupling. Using this procedure, the desired product could be obtained in 34% yield (Table S1). However, screening of multiple bases did not lead to an increased yield.

**Table S1. Deoxygenative arylation of Boc-Thr-OMe through oxalate activation ($^{19}F$ NMR ($CDCl_3$ vs. $PhCF_3$ as internal standard)).**

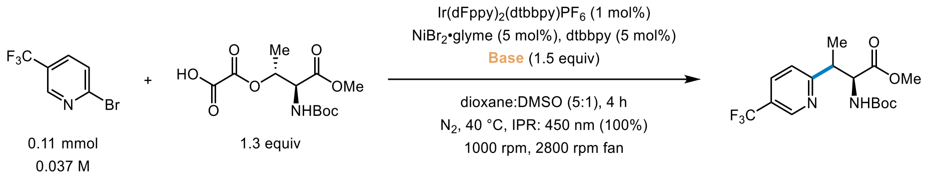


| Entry | Base | Yield | *d.r.* |
|---|---|---|---|
| **1** | $Cs_2CO_3$ | 34% | 1.9:1 |
| **2** | $K_2CO_3$ | 10% | 1.9:1 |
| **3** | $Na_2CO_3$ | 0% | - |
| **4** | $NaHCO_3$ | 0% | - |
| **5** | CsF | 8% | 2.0:1 |
| **6** | Quinuclidine | 0% | - |
| **7** | TMG | 0% | - |
| **8** | BTMG | 5% | 1.4:1 |
| **9** | DBU | 1% | 2.3:1 |
| **10** | DABCO | 3% | 1.9:1 |
| **11** | DBN | 2% | 1.8:1 |
| **12** | DIPEA | 0% | - |

Therefore, we focused on the activation of the alcohol through an *N*-heterocyclic carbene (NHC) which did yield the desired cross-coupling product.[5]

## General Procedure for Reaction Optimization (0.1 mmol scale):

An 8 mL vial was charged with NHC (0.16 mmol, 1.6 equiv) and a magnetic stir bar. After the vial was vacuumed and refilled with nitrogen gas thrice, Boc-Thr-OMe (0.17 mmol, 1.7 equiv) in dry MTBE (0.8 mL) was added and the mixture was stirred at room temperature for 5 min. Then, a pyridine solution (0.16 mmol, 1.6 equiv) in dry MTBE (0.2 mL) was added dropwise at room temperature over the course of 1 min. The resulting suspension was stirred at room temperature for 10 minutes, during which an orange color appeared. Another 8 mL vial was charged with Ir(ppy)$_2$(dtbbpy)PF$_6$ (1.5 µmol, 0.015 equiv), NiBr$_2$•dtbbpy (5.0 µmol, 0.05 equiv), quinuclidine (0.175 mmol, 1.75 equiv), 2-bromo-5-trifluoromethyl)pyridine (0.1 mmol, 1.0 equiv) and a magnetic stir bar. Dry acetone (1.0 mL) was added to this vial under an atmosphere of nitrogen. 1 mL of the NHC suspension was transferred to a syringe under air. Then a syringe filter and new needle were installed on the syringe, before the NHC suspension was injected through the syringe filter into the other solution. The reaction mixture was sparged with nitrogen for 10 minutes before sealing with parafilm. The vial was stirred at 1000 rpm stir rate and irradiated with 450 nm LEDs at 100% light intensity with a maximum fan speed of 6800 rpm in a Penn PhD M2 Integrated Photoreactor for 2 hours. 12.4 µl benzotrifluoride was added as internal standard and the mixture was analyzed by GC, GC-MS, UHPLC-MS and NMR.

Screening of multiple solvents showed that switching from DMA to acetone increased the yield of the cross-coupling product from 36% to 49% (Scheme S3).

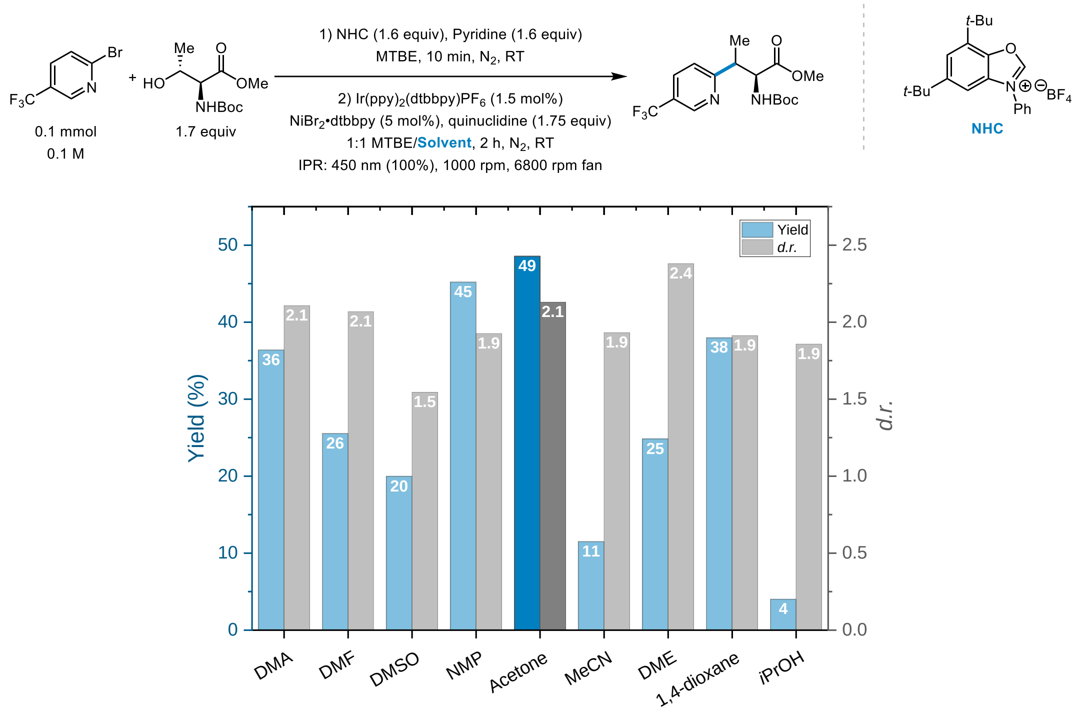


**Scheme S3. Optimization of the solvent for the metallaphotoredox-enabled cross-coupling ($^{19}$F NMR (CDCl$_3$ vs. PhCF$_3$ as internal standard)).**

Next, we looked at multiple solvents for the condensation of threonine with the NHC (Scheme S4). Ethers, such as MTBE and CpOMe performed best, although $Et_2O$ performed significantly worse. Aromatic solvents, like fluorobenzene, benzotrifluoride and toluene also showed good yields, but nevertheless lower than MTBE. Using acetone as solvent for the condensation yielded no cross-coupling product. During the condensation of threonine with the NHC, no precipitate was formed with acetone as solvent, whereas for other solvents a precipitate formed.

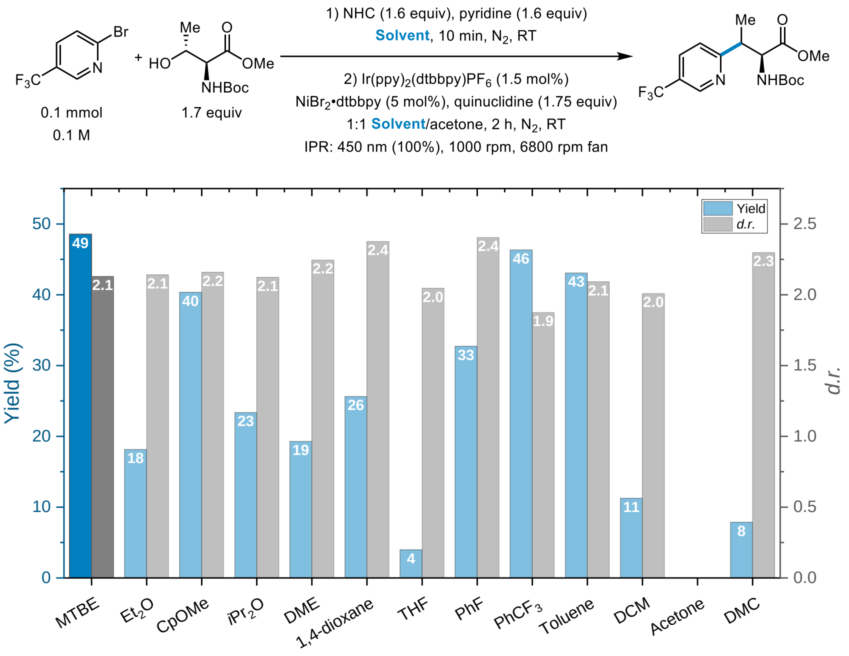


**Scheme S4. Optimization of the solvent for the threonine-NHC condensation ($^{19}$F NMR ($CDCl_3$ vs. $PhCF_3$ as internal standard)).**

Screening of various bipyridine ligands revealed that the dtbbpy ligand is superior (Scheme S5). Moreover, using a preformed catalyst gave better yields than adding the nickel-salt and ligand to the reaction mixture. Additionally, the $NiBr_2$•dtbbpy complex gave higher yields than $NiCl_2$•dtbbpy. None of the screened ligands led to improvements in the *d.r.*

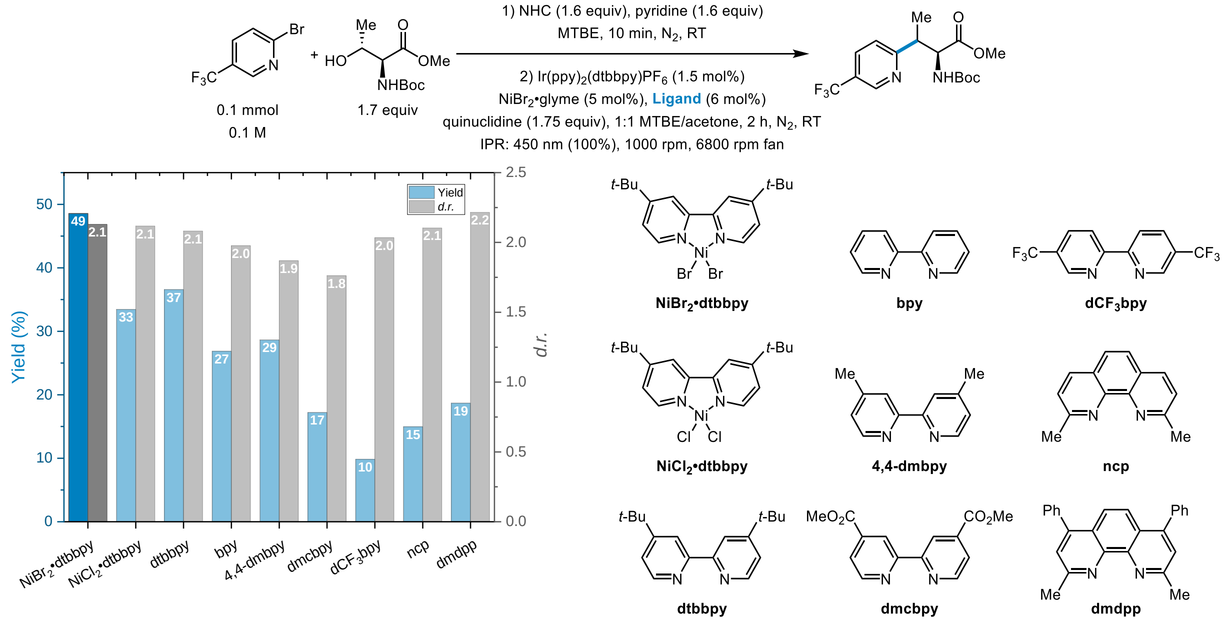


**Scheme S5. Optimization of the ligand for the metallaphotoredox-enabled cross-coupling ($^{19}F$ NMR ($CDCl_3$ vs. $PhCF_3$ as internal standard)).**

Changing the reaction time and temperature of the condensation step showed no improvement of the yield upon increasing the reaction time to 1 hour as well as increasing the reaction temperature to 40 °C (Scheme S6). Increasing the reaction time to 2 hours greatly increased the yield, with only a minor increase observed for a reaction time of 4 hours. We also observed fading of the bright orange color of the reaction mixture upon increasing the reaction time.

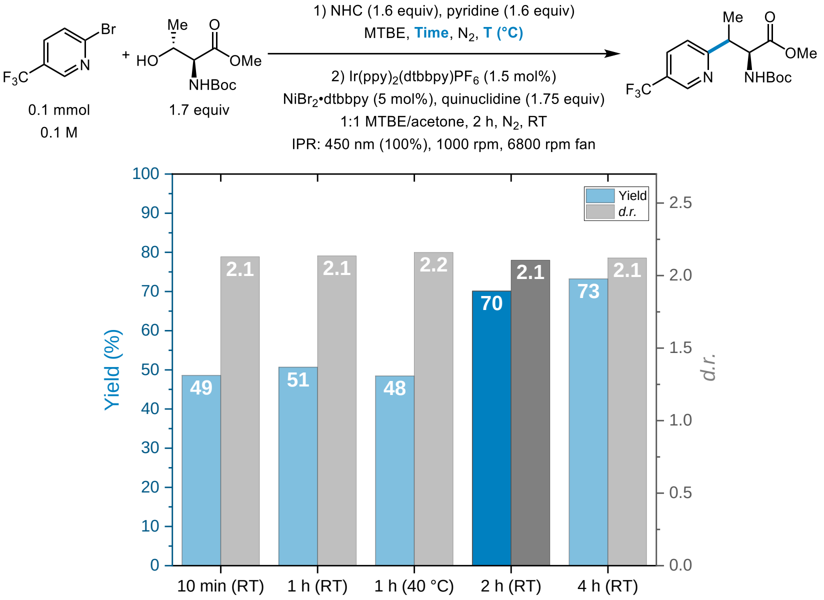


**Scheme S6. Optimization of the reaction time and temperature for the threonine-NHC condensation ($^{19}$F NMR ($CDCl_3$ vs. $PhCF_3$ as internal standard)).**

Multiple substituted pyridines were screened for the condensation step between the NHC and threonine (Scheme S7). None of the substituted pyridines worked better than unsubstituted pyridine. Especially the 2,6-disubstituted pyridines performed poorer, except for 2,6-di-*tert*-butyl-4-methylpyridine. Additionally, electron poor pyridines performed significantly worse.

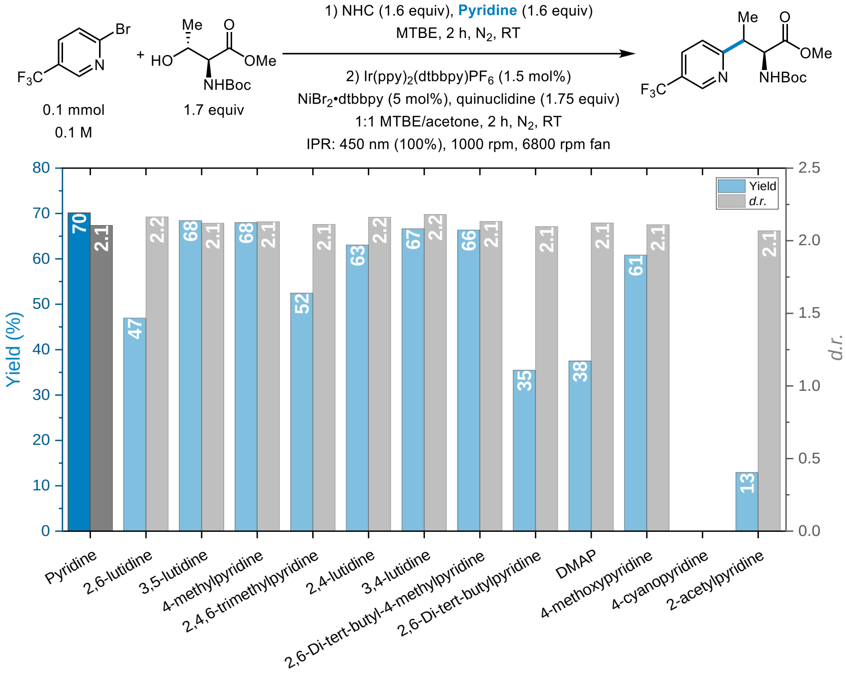


**Scheme S7. Optimization of the base for the threonine-NHC Condensation ($^{19}$F NMR ($CDCl_3$ vs. $PhCF_3$ as internal standard)).**

Similarly, no improvements to the yield were observed by changing the NHC (Scheme S8). Moreover, the *d.r.* was constant across the screened NHCs.

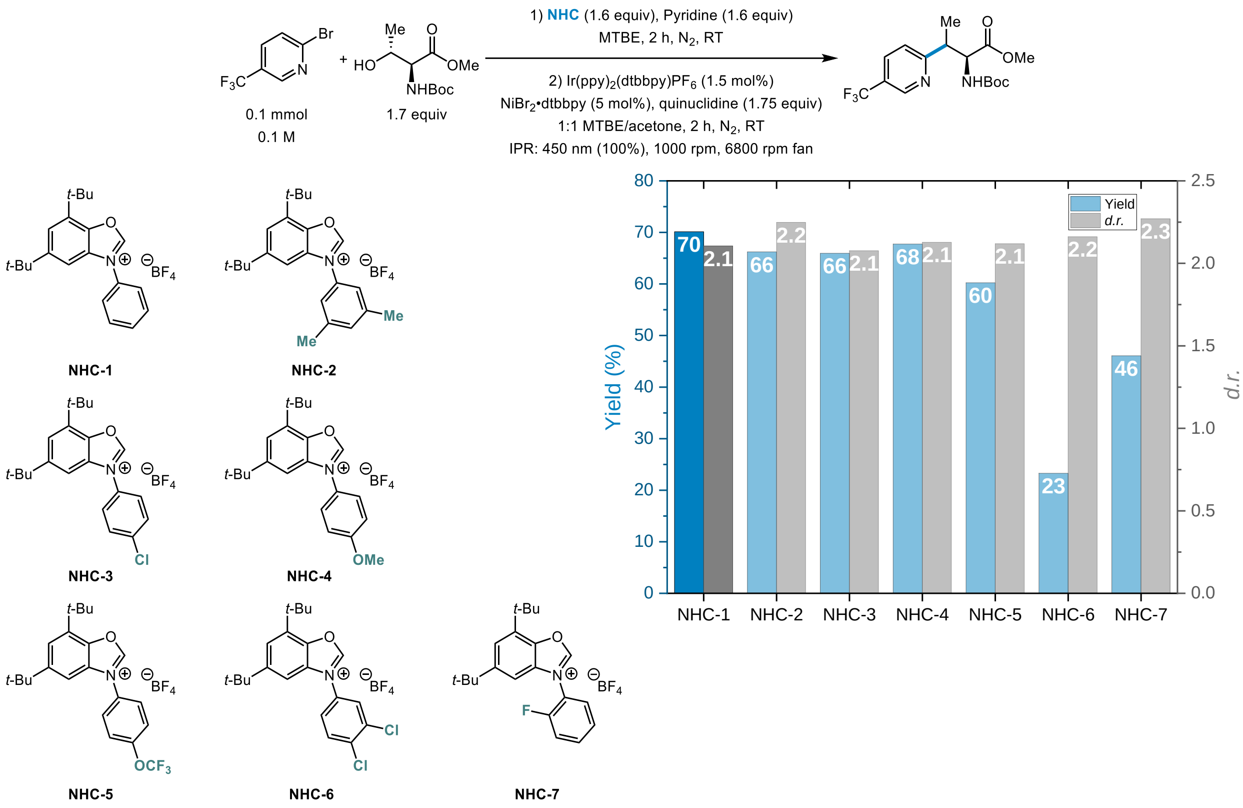


**Scheme S8. Optimization of the NHC for the threonine-NHC condensation ($^{19}$F NMR ($CDCl_3$ vs. $PhCF_3$ as internal standard)).**

Screening various bases, both organic and inorganic, revealed that using KOAc increases the yield to 83% with a concomitant increase in *d.r.* (Scheme S9). Interestingly, BTMG gave a major improvement in *d.r.* although with a reduced yield.

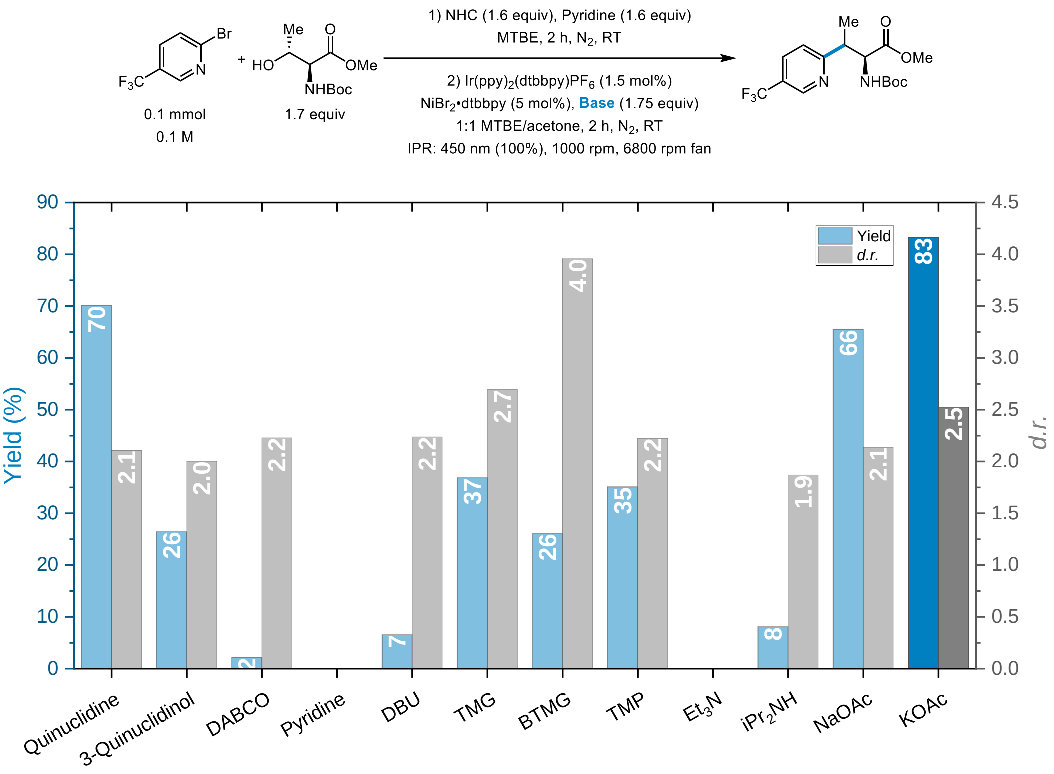


**Scheme S9. Optimization of the base for the metallaphotoredox-enabled cross-coupling ($^{19}F$ NMR ($CDCl_3$ vs. $PhCF_3$ as internal standard)).**

Screening of various photocatalysts showed a small dependency of the yield on the screened photocatalysts (Scheme S10). PC-2 gave a small improvement in yield compared to PC-1, whereas the organic photocatalyst 4CzIPN (PC-4) performed worse.

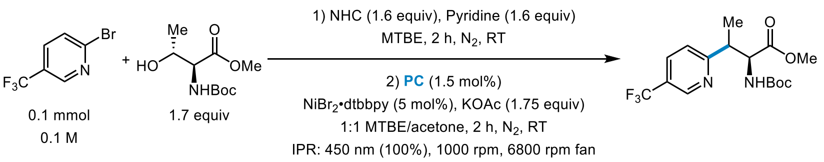


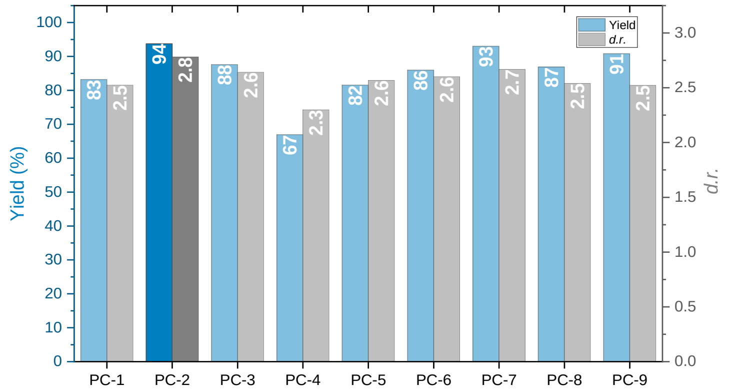


| | |
|---|---|
| PC-1 | Ir(ppy)$_2$(dtbbpy)PF$_6$ |
| PC-2 | Ir(F(Me)ppy)$_2$(dtbbpy)PF$_6$ |
| PC-3 | Ir(dF(Me)ppy)$_2$(dtbbpy)PF$_6$ |
| PC-4 | 4CzIPN |
| PC-5 | Ir(dF(CF$_3$)ppy)$_2$(dtbbpy)PF$_6$ |
| PC-6 | Ir(dFppy)$_2$(bpy)PF$_6$ |
| PC-7 | Ir(dF(OMe)ppy)$_2$(dtbbpy)PF$_6$ |
| PC-8 | Ir(dFppy)$_2$(dtbbpy)PF$_6$ |
| PC-9 | Ir(F(CF$_3$)ppy)$_2$(dtbbpy)PF$_6$ |

**Scheme S10. Optimization of the photocatalyst for the metallaphotoredox-enabled cross-coupling ($^{19}$F NMR ($CDCl_3$ vs. $PhCF_3$ as internal standard)).**

To improve the *d.r.*, some chiral ligands were screened. Sadly, no improvement to the *d.r.* was observed for chiral PyOx, BiOX, BOX and PyBOX ligands (Scheme S11).

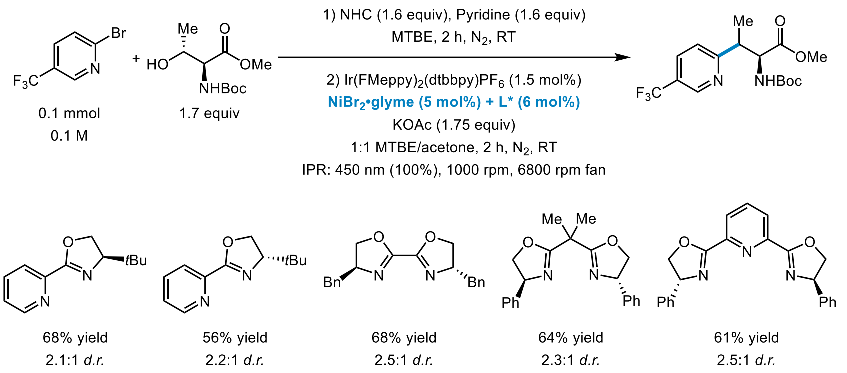


**Scheme S11. Optimization of the *d.r.* for the metallaphotoredox-enabled cross-coupling through chiral ligands ($^{19}$F NMR ($CDCl_3$ vs. $PhCF_3$ as internal standard)).**

Similarly, various terpyridine and phosphine-based ligands (chiral and achiral) were screened without any observed improvement to the *d.r.* (Scheme S12).

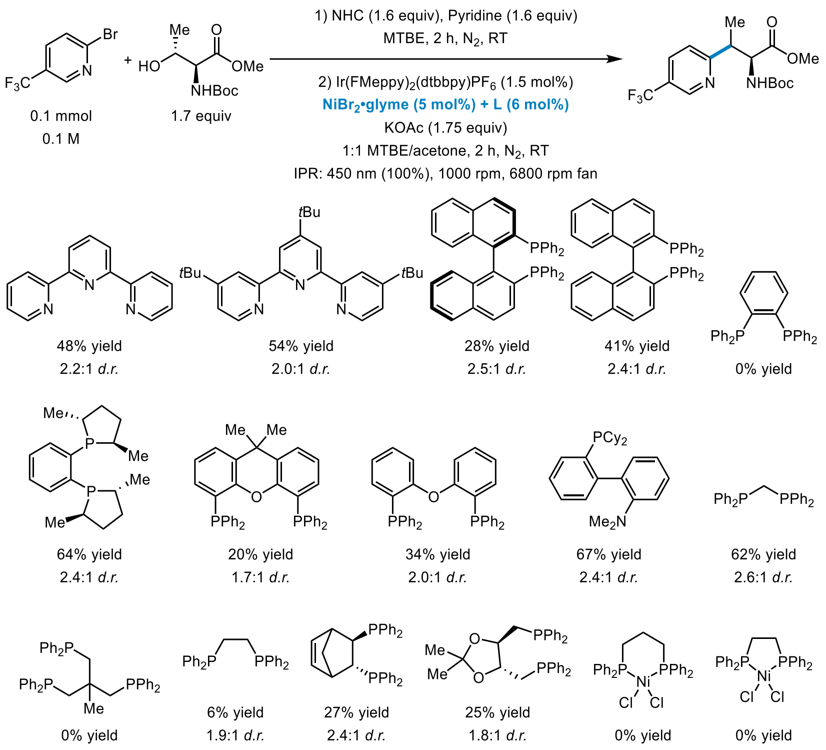


**Scheme S12. Optimization of the *d.r.* for the metallaphotoredox-enabled cross-coupling through terpyridine and phosphine ligands ($^{19}$F NMR ($CDCl_3$ vs. $PhCF_3$ as internal standard)).**

Since we observed poor yields using KOAc as base for other aryl halides, we wanted to test the effect of chiral ligands when using quinuclidine as base (Scheme S13). Unfortunately, this also did not give the product with an improved *d.r.*

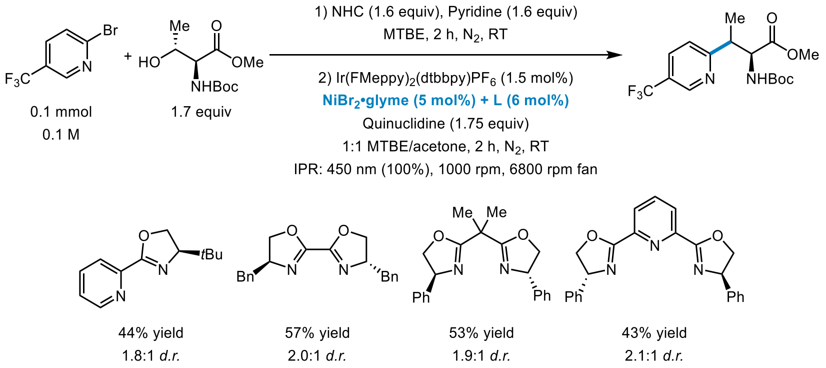


**Scheme S13. Optimization of the *d.r.* for the metallaphotoredox-enabled cross-coupling through chiral ligands using quinuclidine as base ($^{19}$F NMR ($CDCl_3$ vs. $PhCF_3$ as internal standard)).**

Similar results were obtained when 4-bromobenzonitrile was used as aryl halide (Scheme S14).

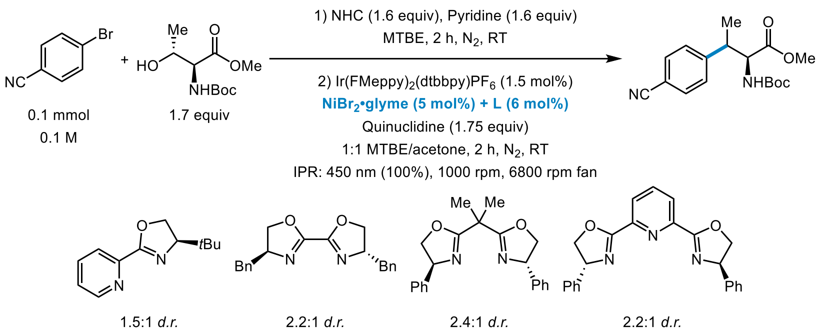


**Scheme S14. Optimization of the *d.r.* for the metallaphotoredox-enabled cross-coupling of 4-bromobenzonitrile through chiral ligands (GC-FID).**

Finally, the addition of 4-methylpyridine was investigated as Zhang *et al.* observed major improvements to the *e.e.* upon its addition.[6] However, no major improvements to the *d.r.* were observed (Scheme S15).

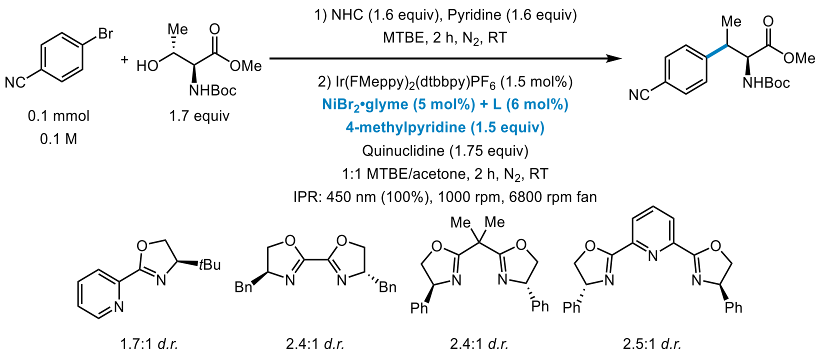


**Scheme S15. Optimization of the *d.r.* for the metallaphotoredox-enabled cross-coupling through chiral ligands and the addition of 4-methylpyridine (GC-FID).**

## 3. Unsuccessful Aryl Halide Substrates

Some aryl halides did not yield the cross-coupling product (Scheme S16). For example, dimethylaniline, azides and a diazobenzene are not tolerated. For both azides, reduction to the respective amine was observed, likely by energy transfer catalyzed nitrogen release. Additionally, bromoferrocene did not yield the desired product. Finally, various nitrogen-containing bicyclic compounds yielded no cross-coupling product.

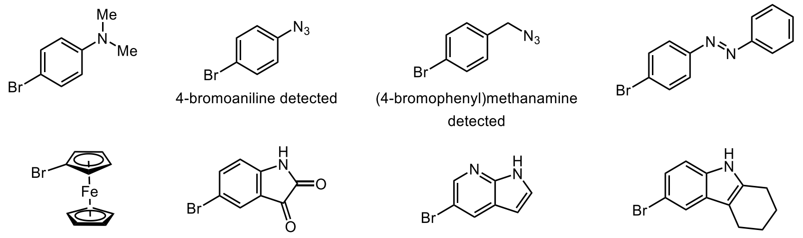


**Scheme S16. Overview of failed aryl halides.**

## 4. Use of Aryl Chloride and Aryl Iodide Substrates

Since aryl chlorides are more cost-effective than aryl bromides, we also attempted their use in the cross-coupling. However, the aryl chlorides that we screened yielded only traces or no product at all, as confirmed by GC-MS. Additionally, we screened some aryl iodides as they generally show higher reactivities. For 4-fluoroiodobenzene the cross-coupling product was obtained in 14% yield. For both 4-iodotoluene and 2-iodopyridine only traces of product were detected by GC-MS.

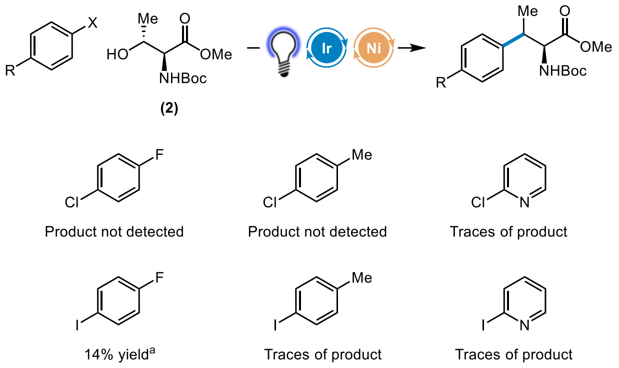


**Scheme S17. Overview of tested aryl chlorides and aryl iodides.**

[a] $^{19}F$ NMR yield of cross-coupling product vs. 1,4-difluorobenzene as internal standard in DMSO-$d_6$.

## 5. Control Experiments

**Table S2. Control experiments of the deoxygenative cross-coupling.[a]**

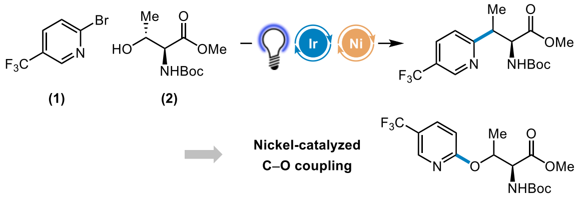


| Entry | Deviation | Yield[b] |
|---|---|---|
| **1** | None | 70% |
| **2** | No $NiBr_2$•dtbbpy | 0% |
| **3** | No $Ir(FMeppy)_2(dtbbpy)PF_6$ | 0% |
| **4** | No quinuclidine | 0% |
| **5** | No light | 0% |
| **6** | No sparging with $N_2$ | 39% |
| **7** | No NHC (C–O coupling) | 0% |

[a] Reaction conditions: **1** (0.1 mmol); **2** (1.7 equiv); NHC (1.6 equiv); pyridine (1.6 equiv); MTBE (1 ml); $Ir(F(Me)ppy)_2(dtbbpy)PF_6$ (1.5 mol%); $NiBr_2$·dtbbpy (5 mol%); quinuclidine (1.75 equiv); acetone (1 ml); IPR: 450 nm (100%), 1000 rpm, 6800 rpm fan. [b] $^{19}F$-NMR yields of cross-coupling product vs. benzotrifluoride as internal standard in $CDCl_3$.

## 6. Kinetic Experiments

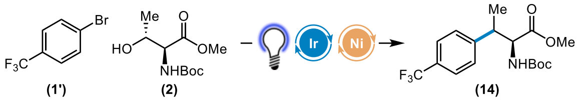


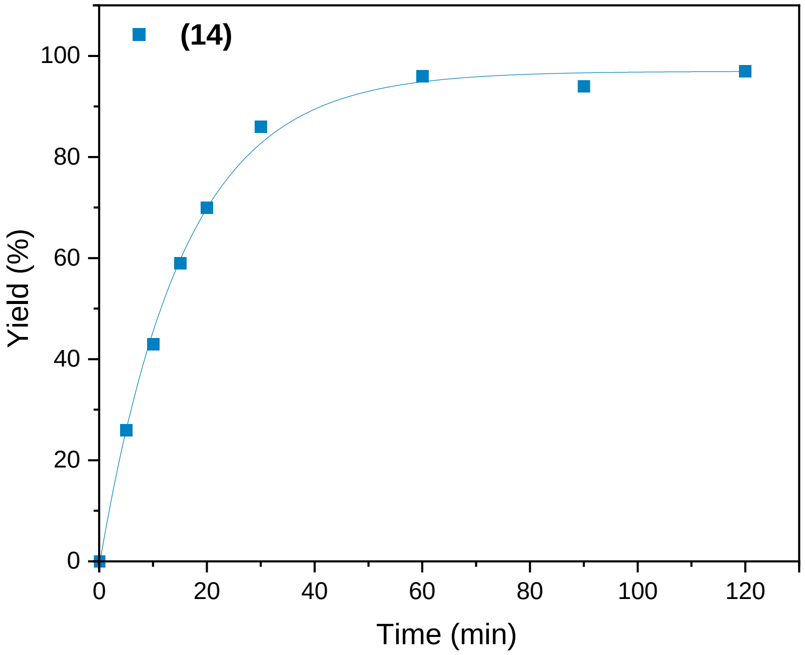


**Scheme S18**. **Kinetic experiments of the deoxygenative cross-coupling of 4-bromobenzotrifluoride (1').**[a]

[a] Reaction conditions: **1'** (0.1 mmol); **2** (1.7 equiv); NHC (1.6 equiv); pyridine (1.6 equiv); MTBE (1 ml); Ir(F(Me)ppy)$_2$(dtbbpy)PF$_6$ (1.5 mol%); NiBr$_2$·dtbbpy (5 mol%); quinuclidine (1.75 equiv); acetone (1 ml); IPR: 450 nm (100%), 1000 rpm, 6800 rpm fan. $^{19}$F-NMR yields of cross-coupling product vs. 4-methylbenzotrifluoride as internal standard in DMSO-d$_6$.

## 7. Mechanistic Experiments

We carried out radical trapping experiments using TEMPO. Upon the addition of TEMPO, no product formation could be detected and the corresponding TEMPO adduct could be detected by HPLC-MS.

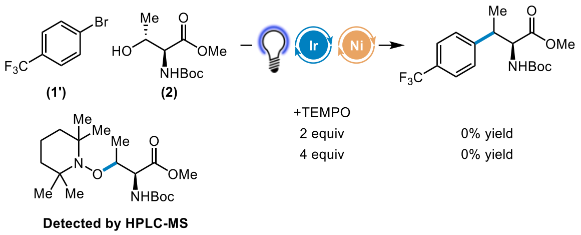


**Scheme S19. Radical trapping experiments of the deoxygenative cross-coupling.[a]**

[a] Reaction conditions: **1'** (0.1 mmol); **2** (1.7 equiv); NHC (1.6 equiv); Pyridine (1.6 equiv); MTBE (1 ml); $Ir(F(Me)ppy)_2(dtbbpy)PF_6$ (1.5 mol%); $NiBr_2$·dtbbpy (5 mol%); Quinuclidine (1.75 equiv); Acetone (1 ml); IPR: 450 nm (100%), 1000 rpm, 6800 rpm fan. $^{19}F$-NMR yields of cross-coupling product vs. 4-methylbenzotrifluoride as internal standard in DMSO-$d_6$.

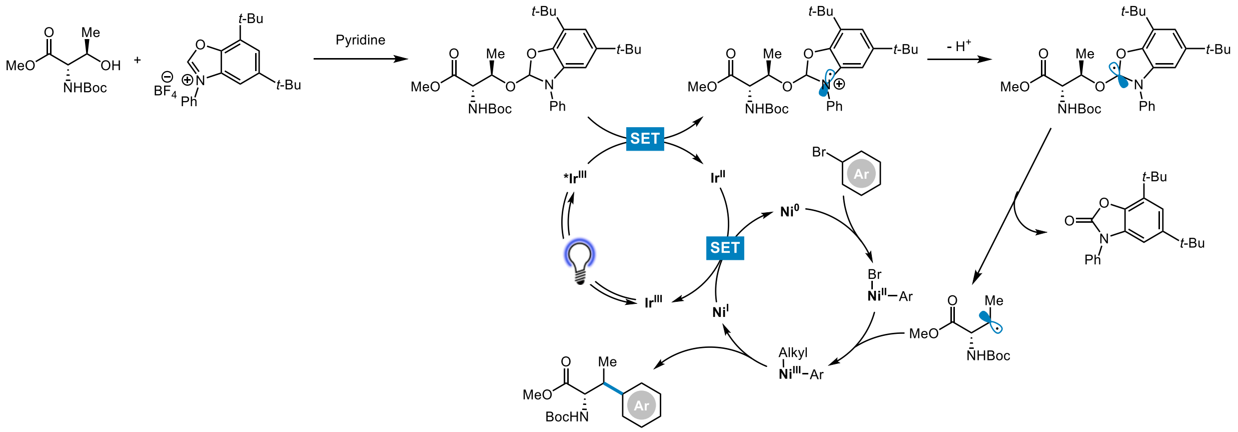


**Scheme S 20. Proposed mechanism for the deoxygenative cross-coupling.[5]**

# 8. Synthesis

## 2,4-Di-*tert*-butyl-6-(phenylamino)phenol

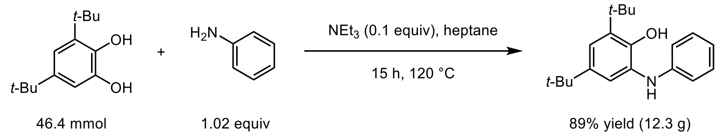


**Step 1:** Procedure adapted from literature.[5] To a suspension of 3,5-di-*tert*-butylcatechol (10.3 g, 46.4 mmol, 1.0 equiv) and aniline (4.3 ml, 47.3 mmol, 1.02 equiv) in *n*-heptane (27 mL) was added triethylamine (0.64 ml, 4.64 mmol, 0.1 equiv). The resulting mixture was heated with a heating block to reflux with Dean-Stark trap under air for 16 h. (The suspension formed a brown homogenous solution upon heating.) The resulting reaction mixture was slowly cooled down to room temperature. A solid precipitated during this process and the entire reaction mixture was stored in the freezer overnight. Then the desired product was collected by filtration, washed with ice-cold pentane and air-dried. The colorless solid (12.3 g, 41.3 mmol, 89% yield) was pure enough for the next step.

**$^1$H NMR** (400 MHz, $CDCl_3$): δ = 7.28 – 7.17 (m, 3H), 7.04 (d, *J* = 2.4 Hz, 1H), 6.86 (t, *J* = 7.3 Hz, 1H), 6.68 (d, *J* = 7.9 Hz, 2H), 6.43 (s, 1H), 4.99 (s, 1H), 1.45 (s, 9H), 1.27 (s, 9H) ppm.

**$^{13}$C NMR** (101 MHz, $CDCl_3$): δ = 149.6, 146.9, 142.3, 135.4, 129.5, 127.8, 122.2, 121.7, 119.9, 115.3, 35.1, 34.5, 31.7, 29.7 ppm.

**GCMS:** $R_f$ = 14.220 min, m/z = 297 $[M]^+$.

## 5,7-Di-*tert*-butyl-3-phenylbenzo[*d*]oxazol-3-ium tetrafluoroborate (NHC-1)

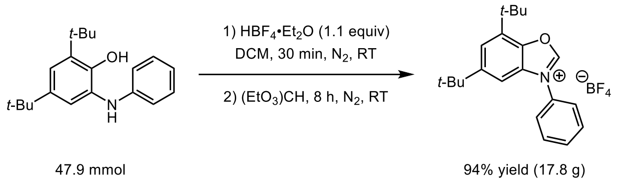


**Step 2:** Procedure adapted from literature.[5] Tetrafluoroboric acid diethyl ether complex (6.9 mL, 50.3 mmol, 1.05 equiv) was added dropwise to a solution of 2,4-di-*tert*-butyl-6-(phenylamino)phenol (14.3 g, 47.9 mmol, 1.00 equiv) with 50 mL of dry DCM in a round bottom flask under nitrogen atmosphere. After finishing the addition, the suspension became a purple homogenous solution. After stirring at room temperature for an additional 30 min, a suspension appeared and the solvent was evaporated in vacuo. The resulting solid was dissolved in triethyl orthoformate (120 mL) at room temperature and the flask was sparged with $N_2$. The mixture was stirred for 8 hours, during which a precipitate appeared. The desired product was collected by filtration as a colorless powder. The solid was further washed with 50 ml diethyl ether to give the pure NHC salt (17.8 g, 45.1 mmol, 94% yield) as an off-white solid.

**$^1$H NMR** (400 MHz, Chloroform-*d*): δ = 10.21 (s, 1H), 7.84 – 7.79 (m, 2H), 7.76 – 7.71 (m, 3H), 7.71 (d, *J* = 1.8 Hz, 1H), 7.37 (d, *J* = 1.8 Hz, 1H), 1.57 (s, 9H), 1.37 (s, 9H) ppm.

**$^{13}$C NMR** (101 MHz, $CDCl_3$): δ = 155.5, 153.9, 146.2, 138.3, 132.4, 131.1, 130.1, 129.3, 125.3, 125.2, 108.0, 36.0, 35.1, 31.6, 30.0 ppm.

**GCMS:** $R_f$ = 14.547 min, m/z = 308 $[M\text{-}BF_4]^+$.

All other NHCs were synthesized using different aniline derivatives using the same protocol.

## 2,4-Di-*tert*-butyl-6-(3,5-dimethylanilino)phenol

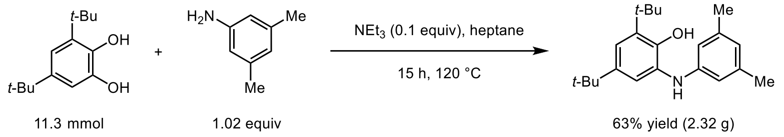


Synthesized according to the above-described protocol. Obtained as a colorless solid (2.32 g, 7.12 mmol, 63% yield).

**$^1$H NMR** (400 MHz, $CDCl_3$): δ = 7.23 (d, *J* = 2.4 Hz, 1H), 7.05 (d, *J* = 2.4 Hz, 1H), 6.52 (s, 1H), 6.42 (s, 1H), 6.32 (s, 2H), 4.89 (s, 1H), 2.24 (s, 6H), 1.45 (s, 9H), 1.27 (s, 9H) ppm.

**$^{13}$C NMR** (101 MHz, $CDCl_3$): δ = 149.5, 146.9, 142.1, 139.3, 135.3, 128.0, 122.0, 121.8, 121.7, 113.1, 35.1, 34.5, 31.7, 29.6, 21.5 ppm.

**GCMS:** $R_f$ = 14.873 min, m/z = 325 $[M]^+$.

## 2,4-Di-*tert*-butyl-6-(4-chloroanilino)phenol

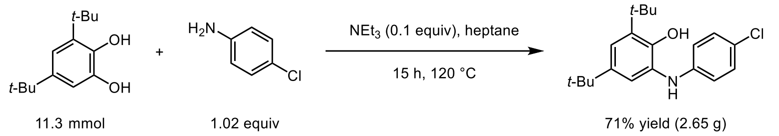


Synthesized according to the above-described protocol. Obtained as an off-white solid (2.65 g, 7.97 mmol, 71% yield).

**$^1$H NMR** (400 MHz, $CDCl_3$): δ = 7.24 (d, *J* = 2.4 Hz, 1H), 7.19 – 7.13 (m, 2H), 7.01 (d, *J* = 2.4 Hz, 1H), 6.63 – 6.57 (m, 2H), 6.33 (s, 1H), 4.99 (s, 1H), 1.45 (s, 9H), 1.27 (s, 9H) ppm.

**$^{13}$C NMR** (101 MHz, $CDCl_3$): δ = 149.4, 145.6, 142.6, 135.6, 129.4, 127.5, 124.7, 122.4, 121.5, 116.5, 35.2, 34.5, 31.7, 29.6 ppm.

**GCMS:** $R_f$ = 15.433 min, m/z = 331 and 333 $[M]^+$.

## 2,4-Di-*tert*-butyl-6-(4-methoxyanilino)phenol

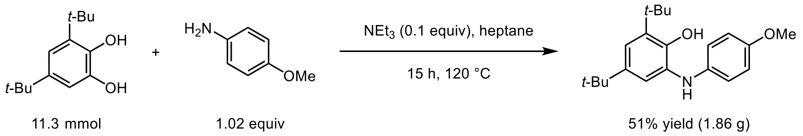


Synthesized according to the above-described protocol. Obtained as a colorless solid (1.86 g, 5.68 mmol, 51% yield).

**$^1$H NMR** (400 MHz, $CDCl_3$): δ = 7.19 (s, 1H), 7.01 (s, 1H), 6.79 (d, *J* = 8.9 Hz, 2H), 6.64 (d, *J* = 8.9 Hz, 2H), 6.50 (s, 1H), 4.79 (s, 1H), 3.76 (s, 3H), 1.44 (s, 9H), 1.26 (s, 9H) ppm.

**$^{13}$C NMR** (101 MHz, $CDCl_3$): δ = 153.8, 149.2, 142.2, 140.5, 135.3, 129.1, 121.6, 121.0, 116.8, 114.9, 55.8, 35.1, 34.5, 31.7, 29.7 ppm.

**GCMS:** $R_f$ = 15.433 min, m/z = 327 $[M]^+$.

## 2,4-Di-*tert*-butyl-6-(4-trifluoromethoxyanilino)phenol

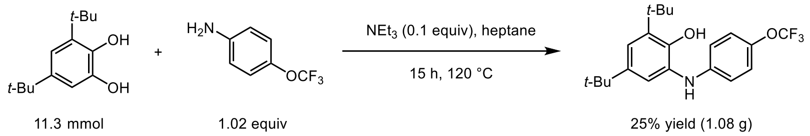


Synthesized according to the above-described protocol. Obtained as a brown solid (1.08 g, 2.84 mmol, 25% yield).

**$^1$H NMR** (400 MHz, $CDCl_3$): δ = 7.24 (d, *J* = 2.4 Hz, 1H), 7.06 (d, *J* = 8.6 Hz, 2H), 7.01 (d, *J* = 2.4 Hz, 1H), 6.68 – 6.60 (m, 2H), 6.30 (s, 1H), 5.05 (s, 1H), 1.44 (s, 9H), 1.27 (s, 9H) ppm.

**$^{13}$C NMR** (101 MHz, $CDCl_3$): δ = 149.4, 145.7, 142.6, 142.4 (q, *J* = 2.1 Hz), 135.7, 127.4, 122.6, 121.6, 120.8 (q, *J* = 258.2 Hz), 115.8, 35.2, 34.6, 31.7, 29.7 ppm.

**$^{19}$F NMR** (376 MHz, $CDCl_3$): δ = -58.4 ppm.

**GCMS:** $R_f$ = 14.123 min, m/z = 381 $[M]^+$.

## 2,4-Di-*tert*-butyl-6-(3,4-dichloroanilino)phenol

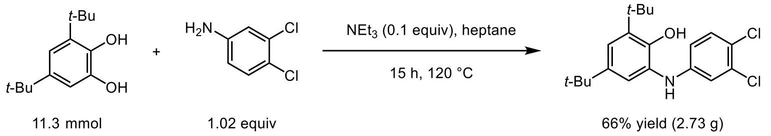


Synthesized according to the above-described protocol. Obtained as an off-white solid (2.73 g, 7.47 mmol, 66% yield).

**$^1$H NMR** (400 MHz, $CDCl_3$): δ = 7.26 – 7.21 (m, 2H), 6.99 (d, *J* = 2.4 Hz, 1H), 6.76 (d, *J* = 2.7 Hz, 1H), 6.50 (dd, *J* = 8.7, 2.7 Hz, 1H), 6.17 (s, 1H), 5.04 (s, 1H), 1.44 (s, 9H), 1.27 (s, 9H) ppm.

**$^{13}$C NMR** (101 MHz, $CDCl_3$): δ = 149.3, 146.6, 142.8, 135.9, 133.2, 131.0, 126.8, 122.8, 122.8, 121.5, 116.8, 114.8, 35.2, 34.6, 31.7, 29.6 ppm.

**GCMS:** $R_f$ = 16.573 min, m/z = 365 and 367 $[M]^+$.

## 2,4-Di-*tert*-butyl-6-(2-fluoroanilino)phenol

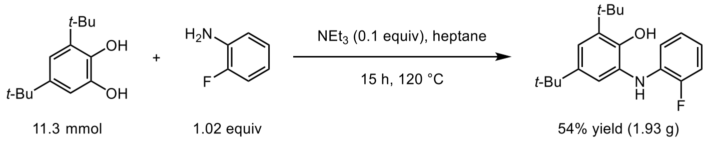


Synthesized according to the above-described protocol. Obtained as a colorless solid (1.93 g, 6.11 mmol, 54% yield).

**$^1$H NMR** (400 MHz, $CDCl_3$): δ = 7.25 (d, *J* = 2.5 Hz, 1H), 7.08 (ddd, *J* = 11.5, 8.1, 1.5 Hz, 1H), 7.03 (d, *J* = 2.4 Hz, 1H), 6.94 (t, *J* = 8.6 Hz, 1H), 6.83 – 6.73 (m, 1H), 6.52 (td, *J* = 8.4, 1.6 Hz, 1H), 6.34 (s, 1H), 5.25 (d, *J* = 3.2 Hz, 1H), 1.45 (s, 9H), 1.27 (s, 9H) ppm.

**$^{13}$C NMR** (101 MHz, $CDCl_3$): δ = 152.4 (d, *J* = 239.6 Hz), 149.7, 142.5, 135.6, 135.2 (d, *J* = 11.3 Hz), 126.7, 124.8 (d, *J* = 3.5 Hz), 122.5, 121.7, 119.6 (d, *J* = 7.0 Hz), 115.7 (d, *J* = 2.6 Hz), 115.1 (d, *J* = 18.4 Hz), 35.2, 34.5, 31.7, 29.6 ppm.

**$^{19}$F NMR** (376 MHz, $CDCl_3$): δ = -135.61 (ddt, *J* = 11.9, 8.5, 4.2 Hz) ppm.

**HRMS** (ESI+) m/z: calcd. for $C_{20}H_{26}NOFNa$ $[M+Na]^+$ 338.1891, found 338.1889.

## 5,7-Di-*tert*-butyl-3-(3,5-dimethylphenyl)benzo[*d*]oxazol-3-ium tetrafluoroborate (NHC-2)

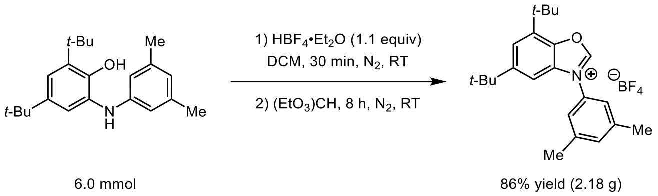


Synthesized according to the above-described protocol. Obtained as a colorless solid (2.18 g, 5.15 mmol, 86% yield).

**$^{1}$H NMR** (400 MHz, $CDCl_3$): δ = 10.23 (s, 1H), 7.71 (d, *J* = 1.8 Hz, 1H), 7.40 (s, 3H), 7.35 (s, 1H), 2.48 (s, 6H), 1.57 (s, 9H), 1.38 (s, 9H) ppm.

**$^{13}$C NMR** (101 MHz, $CDCl_3$): δ = 155.2, 153.9, 146.2, 141.6, 138.4, 134.2, 129.8, 129.3, 125.2, 122.5, 108.2, 36.0, 35.1, 31.6, 30.0, 21.4 ppm.

**GCMS:** $R_f$ = 15.127 min, m/z = 336 $[M-BF_4]^+$.

## 5,7-Di-*tert*-butyl-3-(4-chlorophenyl)benzo[*d*]oxazol-3-ium tetrafluoroborate (NHC-3)

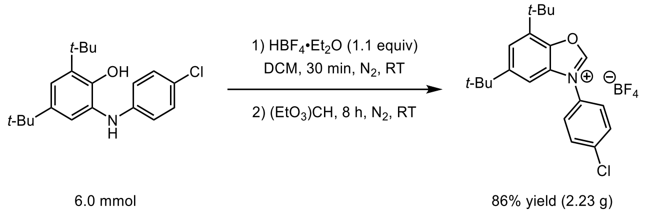


Synthesized according to the above-described protocol. Obtained as a colorless solid (2.23 g, 5.18 mmol, 86% yield).

**$^{1}$H NMR** (400 MHz, $CDCl_3$): δ = 10.21 (s, 1H), 7.83 – 7.75 (m, 2H), 7.74 – 7.65 (m, 3H), 7.31 (d, *J* = 1.8 Hz, 1H), 1.57 (s, 9H), 1.36 (s, 9H) ppm.

**$^{13}$C NMR** (101 MHz, $CDCl_3$): δ = 155.9, 154.0, 146.2, 138.8, 138.4, 131.3, 129.2, 128.5, 126.9, 125.3, 107.7, 36.0, 35.1, 31.6, 30.0.

**GCMS:** $R_f$ = 15.533 min, m/z = 342 and 344 $[M-BF_4]^+$.

## 5,7-Di-*tert*-butyl-3-(4-methoxyphenyl)benzo[*d*]oxazol-3-ium tetrafluoroborate (NHC-4)

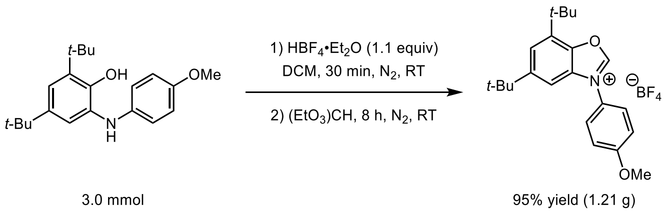


Synthesized according to the above-described protocol. Obtained as a blue solid (1.21 g, 2.85 mmol, 95% yield).

**$^1$H NMR** (400 MHz, $CDCl_3$): δ = 10.20 (s, 1H), 7.74 (d, *J* = 8.6 Hz, 2H), 7.71 (d, *J* = 1.7 Hz, 1H), 7.37 (d, *J* = 1.7 Hz, 1H), 7.20 (d, *J* = 8.3 Hz, 2H), 3.93 (s, 3H), 1.57 (s, 9H), 1.37 (s, 9H) ppm.

**$^{13}$C NMR** (101 MHz, $CDCl_3$): δ = 162.3, 155.2, 153.7, 146.1, 138.2, 129.6, 126.7, 125.0, 122.4, 116.2, 108.1, 56.0, 36.0, 35.1, 31.5, 29.9 ppm.

**GCMS:** $R_f$ = 15.843 min, m/z = 338 $[M\text{-}BF_4]^+$.

## 5,7-Di-*tert*-butyl-3-(4-(trifluoromethoxy)phenyl)benzo[*d*]oxazol-3-ium tetrafluoroborate (NHC-5)

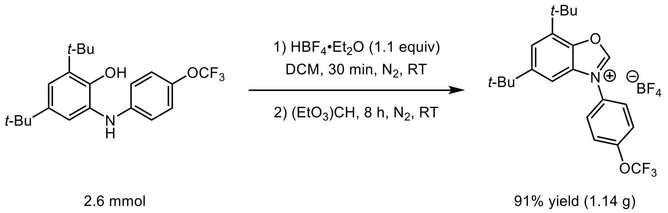


Synthesized according to the above-described protocol. Obtained as a light pink solid (1.14 g, 2.4 mmol, 91% yield).

**$^1$H NMR** (400 MHz, $CDCl_3$): δ = 10.18 (s, 1H), 7.95 – 7.87 (m, 2H), 7.71 (d, *J* = 1.8 Hz, 1H), 7.55 (d, *J* = 8.5 Hz, 2H), 7.32 (d, *J* = 1.9 Hz, 1H), 1.57 (s, 9H), 1.37 (s, 9H) ppm.

**$^{13}$C NMR** (101 MHz, $CDCl_3$): δ = 156.1, 154.0, 151.7 (q, *J* = 1.9 Hz), 146.2, 138.3, 129.2, 128.2, 127.6, 125.2, 122.9, 120.4 (q, *J* = 259.8 Hz), 107.7, 36.0, 35.1, 31.6, 29.9 ppm.

**$^{19}$F NMR** (376 MHz, $CDCl_3$): δ = -57.71, -151.34 (d, *J* = 18.7 Hz) ppm.

**GCMS:** $R_f$ = 14.173 min, m/z = 392 $[M\text{-}BF_4]^+$.

## 5,7-Di-*tert*-butyl-3-(3,4-dichlorophenyl)benzo[*d*]oxazol-3-ium tetrafluoroborate (NHC-6)

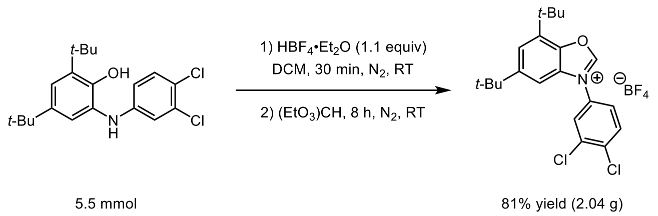


Synthesized according to the above-described protocol. Obtained as a colorless solid (2.04 g, 4.4 mmol, 81% yield).

**$^1$H NMR** (400 MHz, $CD_3CN$): δ = 9.85 (s, 1H), 7.95 (d, *J* = 8.5 Hz, 1H), 7.94 (d, *J* = 2.6 Hz, 1H) , 7.83 (d, *J* = 1.8 Hz, 1H), 7.70 (dd, *J* = 8.7, 2.5 Hz, 1H), 7.55 (d, *J* = 1.8 Hz, 1H), 1.57 (s, 9H), 1.38 (s, 9H) ppm.

**$^{13}$C NMR** (101 MHz, $CD_3CN$): δ = 156.5, 154.9, 146.8, 138.3, 137.4, 135.1, 133.6, 130.5, 130.5, 128.8, 126.6, 126.3, 109.8, 36.8, 35.6, 31.5, 29.9 ppm.

**GCMS:** $R_f$ = 16.443 min, m/z = 376 and 378 $[M-BF_4]^+$.

## 5,7-Di-*tert*-butyl-3-(2-fluorophenyl)benzo[*d*]oxazol-3-ium tetrafluoroborate (NHC-7)

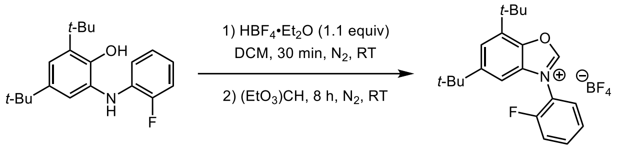


Synthesized according to the above-described protocol. Obtained as a colorless solid (2.09 g, 5.1 mmol, 93% yield).

**$^1$H NMR** (400 MHz, $CDCl_3$): δ = 10.29 (s, 1H), 8.08 (ddd, *J* = 8.8, 7.4, 1.7 Hz, 1H), 7.83 – 7.76 (m, 1H), 7.75 (d, *J* = 1.8 Hz, 1H), 7.59 (t, *J* = 7.8 Hz, 1H), 7.50 (ddd, *J* = 9.8, 8.4, 1.2 Hz, 1H), 7.29 (t, *J* = 2.0 Hz, 1H), 1.60 (s, 9H), 1.38 (s, 9H) ppm.

**$^{13}$C NMR** (101 MHz, $CDCl_3$): δ = 156.7, 155.9 (d, *J* = 255.9 Hz), 154.3, 146.0, 138.4, 134.8 (d, *J* = 7.6 Hz), 129.1, 128.7, 127.0 (d, *J* = 4.0 Hz), 125.5, 117.82 (d, *J* = 12.4 Hz), 117.70 (d, *J* = 18.1 Hz), 108.2 (d, *J* = 2.5 Hz), 36.1, 35.2, 31.6, 30.1 ppm.

**$^{19}$F NMR** (376 MHz, $CDCl_3$): δ = -120.35 – -120.53 (m), -151.63 (d, *J* = 20.1 Hz) ppm.

**HRMS** (ESI+) m/z: calcd. for $C_{21}H_{25}NOF$ $[M-BF_4]^+$ 326.1915, found 326.1914.

## 1-Bromo-4-(but-2-yn-1-yloxy)benzene

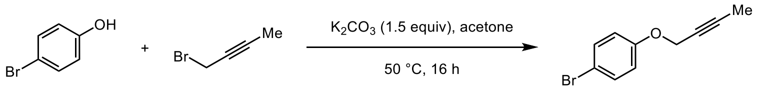


Procedure adapted from literature.[7] To a suspension of 4-bromophenol (865 mg, 5.0 mmol, 1.0 equiv) and $K_2CO_3$ (1.05 g, 7.5 mmol, 1.5 equiv) in acetone (10 mL) was added 1-bromo-2-butyne (0.66 ml, 7.5 mmol, 1.5 equiv). The reaction mixture was heated with a heating block to 50 °C for 16 h. Afterwards, the reaction mixture was filtered and the solvent evaporated *in vacuo*. The residue was purified by silica gel flash column chromatography (EtOAc:cyclohexane = 0:100 → 6:94) to afford the product as a colorless solid (1.02 g, 4.5 mmol, 91% yield).

**$^1$H NMR** (400 MHz, $CDCl_3$): δ = 7.43 – 7.34 (m, 2H), 6.89 – 6.81 (m, 2H), 4.62 (q, *J* = 2.4 Hz, 2H), 1.86 (t, *J* = 2.3 Hz, 3H) ppm.

**$^{13}$C NMR** (101 MHz, $CDCl_3$): δ = 157.1, 132.4, 116.9, 113.6, 84.3, 73.8, 56.8, 3.8 ppm.

**GCMS:** $R_f$ = 10.430 min, m/z = 224 and 226 $[M]^+$.

## Hexyl 4-bromobenzoate

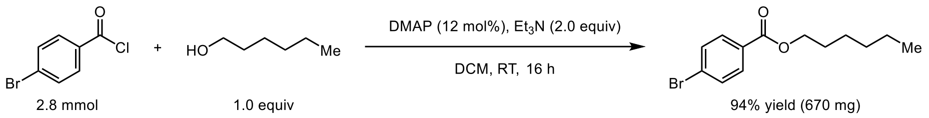


Procedure adapted from literature.[8] 4-Bromobenzoyl chloride (609 mg, 2.8 mmol, 1.1 equiv) was dissolved in DCM (5 mL) and added to a mixture of hexan-1-ol (256 mg, 2.5 mmol, 1.0 equiv), DMAP (36.5 mg, 0.3 mmol, 12 mol%) and $Et_3N$ (0.70 ml, 5.1 mmol, 2.0 equiv) in DCM (5 mL). The homogeneous mixture stirred overnight at room temperature, during which a precipitate formed. Then, the reaction was quenched by adding 15 ml of $NH_4Cl$ (aq. 10%) and extracted with DCM three times. The organic layer was washed with brine, dried over $MgSO_4$, filtered and concentrated *in vacuo*. The crude product was purified by silica gel flash column chromatography (EtOAc:cyclohexane = 5:95) to give the pure product as a clear colorless liquid (670 mg, 2.35 mmol, 94% yield).

**$^1$H NMR** (400 MHz, $CDCl_3$): δ = 7.94 – 7.85 (m, 2H), 7.62 – 7.53 (m, 2H), 4.31 (t, *J* = 6.8 Hz, 2H), 1.82 – 1.70 (m, 2H), 1.48 – 1.39 (m, 2H), 1.38 – 1.30 (m, 4H), 0.94 – 0.87 (m, 3H) ppm.

**$^{13}$C NMR** (101 MHz, $CDCl_3$): δ = 166.1, 131.8, 131.2, 129.6, 128.0, 65.6, 31.6, 28.8, 25.8, 22.7, 14.1 ppm.

**GCMS:** $R_f$ = 12.487 min, m/z = 237 and 239 $[M]^+$.

## 1-Acetyl-5-bromoindole

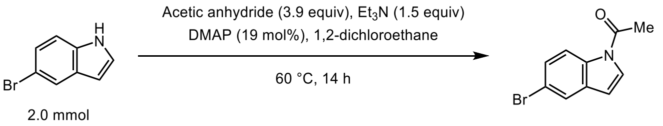


Procedure adapted from literature.[9] 5-Bromoindole (393 mg, 2.0 mmol, 1 equiv) and DMAP (46 mg, 0.38 mmol, 19 mol%) were dissolved in 5 ml 1,2-dichloroethane. Triethylamine (0.42 mL, 3.0 mmol, 1.5 equiv) and acetic anhydride (0.73 mL, 7.8 mmol, 3.9 equiv) were added. The mixture was heated to 60 °C with a heating block for 14 hours. Afterwards, the mixture was cooled down and sat. aq. $NH_4Cl$ (10 ml) was added. The mixture was transferred to a separatory funnel and extracted with EtOAc (3 x 10 ml). The organic layer was dried over $MgSO_4$, filtered and concentrated *in vacuo*. The crude product was purified by silica gel flash column chromatography (EtOAc:cyclohexane = 25:75) to give the pure product as a colorless solid (435 mg, 1.8 mmol, 91% yield).

**$^1$H NMR** (400 MHz, $CDCl_3$): δ = 8.33 (d, *J* = 8.8 Hz, 1H), 7.70 (t, *J* = 1.6 Hz, 1H), 7.48 – 7.39 (m, 2H), 6.58 (ddd, *J* = 3.8, 1.6, 0.8 Hz, 1H), 2.63 (d, *J* = 1.1 Hz, 3H) ppm.

**$^{13}$C NMR** (101 MHz, $CDCl_3$): δ = 168.7, 134.4, 132.2, 128.1, 126.4, 123.6, 118.1, 117.1, 108.5, 24.0.

**GCMS:** $R_f$ **=** 12.280 min, m/z = 284 and 286 $[M]^+$.

## 4-Bromobenzyl azide

4-Bromobenzyl azide was synthesized according to a procedure adapted from literature.[10] All spectroscopic data matched literature report.

## 1-Azido-4-bromobenzene

1-Azido-4-bromobenzene was synthesized according to a procedure adapted from literature.[11] All spectroscopic data matched literature report.

## 4-Bromoazobenzene

4-Bromoazobenzene was synthesized according to a procedure adapted from literature.[12] All spectroscopic data matched literature report.

## 2-(4-Fluorophenyl)-5-methylpyridine

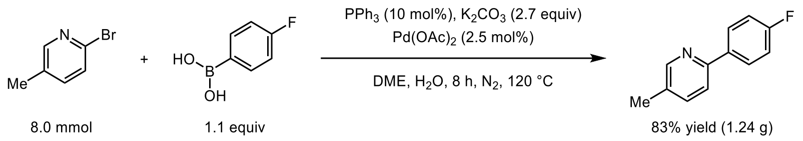


Procedure adapted from literature.[1] A 3-neck round bottom flask was charged with 2-bromo-5-methylpyridine (1.37 g, 8.00 mmol, 1.0 equiv), (4-fluorophenyl)boronic acid (1.24 g, 8.80 mmol, 1.1 equiv), triphenylphosphine (210 mg, 0.80 mmol, 10 mol%), and potassium carbonate (2.98 g, 21.6 mmol, 2.7 equiv). Dimethoxyethane (16 mL, 0.5 M) and water (10 mL, 0.75 M) were added and the reaction was sparged with $N_2$ for 15 min at room temperature. Then palladium acetate (46.0 mg, 0.20 mmol, 2.5 mol%) was added, and the reaction was sparged with $N_2$ for an additional 15 min at room temperature. The reaction was heated to reflux (120 °C) with a heating block for 8 h under $N_2$ atmosphere and for another 15 h at 80 °C. Upon completion, the reaction was cooled to room temperature and diluted with ammonium chloride (sat. aq., 70 mL). The organic layer was extracted with DCM (3 x 70 mL) and washed with brine (70 mL). The organic layer was dried over $MgSO_4$, filtered, and concentrated to afford a yellow oil. The crude residue was purified by silica flash column chromatography (EtOAc:cyclohexane = 0:100 → 10:90) to afford colorless crystals (1.24 g, 6.62 mmol, 83% yield).

**$^{1}$H NMR** (400 MHz, $CDCl_3$): δ = 8.53 – 8.47 (m, 1H), 7.99 – 7.90 (m, 2H), 7.62 – 7.52 (m, 2H), 7.19 – 7.09 (m, 2H), 2.37 (s, 3H) ppm.

**$^{13}$C NMR** (101 MHz, $CDCl_3$): δ = 163.4 (d, *J* = 247.8 Hz), 153.9, 150.1, 137.6, 135.6 (d, *J* = 3.2 Hz), 131.7, 128.56 (d, *J* = 8.3 Hz), 119.9, 115.7 (d, *J* = 21.5 Hz), 18.3 ppm.

**$^{19}$F NMR** (376 MHz, $CDCl_3$): δ = -113.8 ppm.

**GCMS:** $R_f$ = 10.737 min, m/z = 187 $[M]^+$.

## $[Ir(F(Me)ppy)_2Cl]_2$

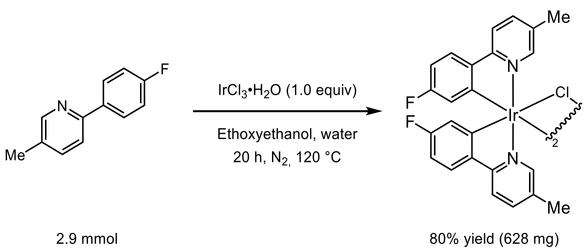


Procedure adapted from literature.[1] A 2-neck round bottom flask was charged with 2-(4-fluorophenyl)-5-methylpyridine (538 mg, 2.9 mmol, 2.2 equiv) and $IrCl_3xH_2O$ (410 mg, 1.3 mmol, 1.0 equiv), then evacuated and backfilled with nitrogen (5x). Ethoxyethanol:water (3:1, 20 ml; previously degassed for ca.

1 h by sparging with nitrogen) was added under nitrogen and the reaction was heated to 120 °C with a heating block for 20 h. The yellow mixture was filtered and the yellow solids were washed with water and cyclohexane (ca. 200 mL each). The fine yellow powder was dried under high vacuum overnight to afford the dimeric intermediate as yellow powder (628 mg, 1.04 mmol, 80% yield). No further characterization was performed.

## $Ir(F(Me)ppy)_2(dtbbpy)PF_6$

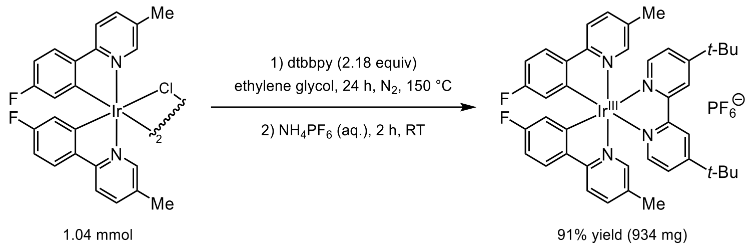


Procedure adapted from literature.[1] A 3-neck round bottom flask was charged with dimer $Ir_2$ (627 mg, 1.04 mmol, 1.0 equiv) and 4-*tert*-butyl-2-(4-*tert*-butylpyridin-2-yl)pyridine (614 mg, 2.3 mmol, 2.18 equiv), then evacuated and backfilled with nitrogen (5x). Ethylene glycol (40 mL, 0.025 M, previously degassed for 30 min by sparging with nitrogen) was added under nitrogen. The suspension was then heated to 150 °C with a heating block for 22 h, during which it became homogeneous. The reaction was then cooled to room temperature and an aqueous solution of ammonium hexafluorophosphate (1 g in 10 mL, 6 mL) was added. A precipitate formed immediately, and the suspension was stirred for 2 h at room temperature. The solids were collected by filtration and were washed with water and cyclohexane (ca. 100 mL each). Recrystallization from acetone/cyclohexane (1:1) afforded the product as a fine yellow powder (934 mg, 0.95 mmol, 91% yield). Additional characterization data was reported.[13]

**$^1$H NMR** (400 MHz, Acetone-$d_6$): δ = 8.87 (d, *J* = 2.1 Hz, 2H), 8.12 (d, *J* = 8.4 Hz, 2H), 8.04 (d, *J* = 5.9 Hz, 2H), 7.93 (dd, *J* = 8.6, 5.6 Hz, 2H), 7.82 (dd, *J* = 8.5, 2.1 Hz, 2H), 7.74 (dd, *J* = 5.9, 2.0 Hz, 2H), 7.48 (d, *J* = 2.0 Hz, 2H), 6.80 (td, *J* = 8.8, 2.6 Hz, 2H), 5.93 (dd, *J* = 9.6, 2.6 Hz, 2H), 2.11 (s, 6H), 1.43 (s, 18H) ppm.

**$^{13}$C NMR** (101 MHz, Acetone-$d_6$): δ = 165.6, 165.2, 163.1, 156.9, 154.3 (d, *J* = 5.5 Hz), 151.3, 149.1, 141.5 (d, *J* = 2.2 Hz), 140.7, 134.6, 127.5 (d, *J* = 9.2 Hz), 126.6, 123.0, 120.5, 118.3 (d, *J* = 17.8 Hz), 110.2 (d, *J* = 23.6 Hz), 36.5, 30.4, 18.0.

**$^{19}$F NMR** (376 MHz, Acetone-$d_6$): δ = -72.60 (d, *J* = 707.5 Hz), -111.52 (td, *J* = 9.4, 5.5 Hz) ppm.

**HRMS** (ESI+) m/z: calcd. for $C_{42}H_{42}N_4F_2Ir$ $[M-PF_6]^+$ 833.3001, found 833.2999.

## $NiBr_2$•dtbbpy

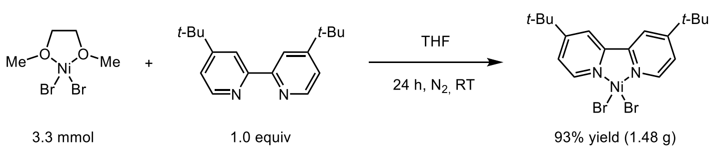


Procedure adapted from literature.[14] To a solution of 4,4'-di-*tert*-butyl-2,2'-bipyridine (873 mg, 3.26 mmol, 1.0 equiv) in 16 ml dry THF was added $NiBr_2$•glyme while stirring (1.01 g, 3.26 mmol, 1.0 equiv). The reaction mixture was sparged with N2 and stirred under $N_2$ atmosphere for 24 hours, during which a blue/green suspension formed. $Et_2O$ (60 mL) was then added and the resulting blue/green precipitate was collected by filtration and washed with 60 ml $Et_2O$. The residue was then resuspended in 50 ml MTBE and

stirred for 2 h, filtered and washed with 50 ml MTBE. The solid was dried *in vacuo* to yield (dtbbpy)$NiBr_2$ as a pale green solid (1.48 g, 3.04 mmol, 93% yield). The complex was used without further purification.

## Boc-Thr-Ala-OMe

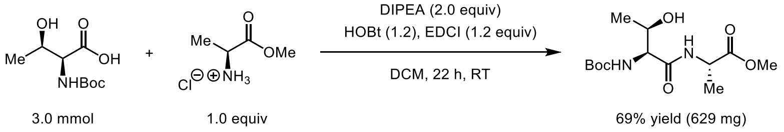


Procedure adapted from literature.[15] To a solution of Boc-Threonine (654 mg, 3.0 mmol, 1 equiv) and *L*-alanine methyl ester hydrochloride (417 mg, 3.0 mmol, 1 equiv) in 10 ml dichloromethane was added diisopropylethylamine (1.0 ml, 5.9 mmol, 2.0 equiv), HOBt (490 mg, 3.5 mmol, 1.2 equiv) and EDCI (693 mg, 3.5 mmol, 1.2 equiv). The reaction mixture was stirred at room temperature for 22 hours and was then quenched with sat. aq. $NH_4Cl$. The aqueous layer was extracted with dichloromethane three times. The combined organic layers were washed successively with aqueous hydrochloric acid (0.5 M), sat. aq. $NaHCO_3$ and brine and dried over $MgSO_4$, filtered and concentrated *in vacuo*. The crude product was purified using reverse phase flash column chromatography (MeCN:$H_2O$ = 10:90 → 30:70) to obtain the product as a clear oil (629 mg, 2.1 mmol, 69% yield).

**$^1$H NMR** (400 MHz, $CDCl_3$): δ = 7.19 (d, *J* = 7.5 Hz, 1H), 5.58 (d, *J* = 7.9 Hz, 1H), 4.53 (p, *J* = 7.3 Hz, 1H), 4.33 – 4.23 (m, 1H), 4.11 (d, *J* = 6.1 Hz, 1H), 3.72 (s, 3H), 3.30 (s, 1H), 1.42 (s, 9H), 1.38 (d, *J* = 7.3 Hz, 3H), 1.17 (d, *J* = 6.4 Hz, 3H) ppm.

**$^{13}$C NMR** (101 MHz, $CDCl_3$): δ = 173.2, 171.1, 156.5, 80.4, 67.2, 58.2, 52.6, 48.2, 28.4, 18.1, 18.0.

**UP-LCMS:** $R_f$ = 1.162 min, m/z = 327 $[M+Na]^+$.

## Tos-Thr-OMe

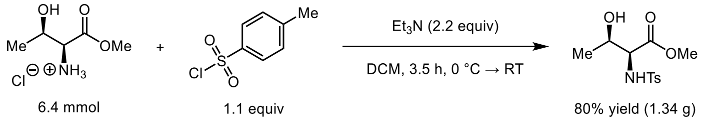


Procedure adapted from literature.[16] To a solution of *L*-Threonine methyl ester hydrochloride (991 mg, 5.8 mmol, 1 equiv) in 10 ml DCM triethyl amine (1.8 ml, 12.9 mmol, 2.2 equiv) was added. The mixture was cooled in an ice-bath and *p*-toluenesulfonyl chloride (1.22 g, 6.4 mmol, 1.1 equiv) was added slowly. After stirring at 0 °C for 30 minutes, the mixture was allowed to warm up to room temperature and stirred for 3 hours. The reaction mixture was concentrated *in vacuo* and the residue dissolved in 100 ml EtOAc. The organic layer was washed with 1 M $KHSO_4$ (50 ml) and sat. aq. $NaHCO_3$ (50 ml), dried with brine, $MgSO_4$, filtered and concentrated *in vacuo*. The crude product was purified by silica gel flash column chromatography (EtOAc:cyclohexane = 15:85 → 50:50) to give the pure product as a colorless solid (1.34 g, 4.67 mmol, 80% yield).

**$^1$H NMR** (400 MHz, $CDCl_3$): δ = 7.76 – 7.68 (m, 2H), 7.33 – 7.27 (m, 2H), 5.34 (d, *J* = 9.5 Hz, 1H), 4.20 – 4.08 (m, 1H), 3.82 (dd, *J* = 9.5, 3.0 Hz, 1H), 3.52 (s, 3H), 2.42 (s, 3H), 1.92 (d, *J* = 5.6 Hz, 1H), 1.28 (d, *J* = 6.3 Hz, 3H) ppm.

**$^{13}$C NMR** (101 MHz, $CDCl_3$): δ = 170.8, 143.9, 136.8, 129.8, 127.4, 68.5, 61.0, 52.8, 21.7, 20.0 ppm.

**UP-LCMS:** $R_f$ = 1.360 min, m/z = 288 $[M+H]^+$.

## Phth-Thr-OMe

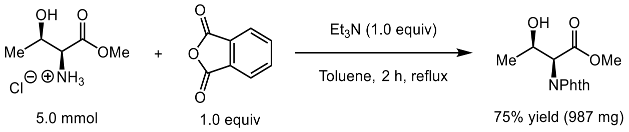


Procedure adapted from literature.[17] To a suspension of *L*-Threonine methyl ester hydrochlorizde (851 mg, 5.0 mmol, 1 equiv) and phthalic anhydride (745 mg, 5.0 mmol, 1 equiv) in 25 ml toluene, triethyl amine (0.7 ml, 5.0 mmol, 1 equiv) was added. The resulting mixture was heated with a heating block to refluc with Dean-Stark trap under air for 2 h. The reaction mixture was cooled down, concentrated *in vacuo* and the residue dissolved in 50 ml EtOAc. The organic layer was washed with 10% citric acid (50 ml), water (50 ml) and sat. aq. $NaHCO_3$ (50 ml), dried with brine, $MgSO_4$, filtered and concentrated *in vacuo* to give the pure product as a colorless solid (987 mg, 3.74 mmol, 75% yield).

**$^1$H NMR** (400 MHz, $CDCl_3$): δ = 7.91 (dd, *J* = 5.5, 3.0 Hz, 2H), 7.79 (dd, *J* = 5.5, 3.1 Hz, 2H), 4.99 (d, *J* = 4.2 Hz, 1H), 4.72 – 4.59 (m, 1H), 4.07 (d, *J* = 9.8 Hz, 1H), 3.80 (s, 3H), 1.22 (d, *J* = 6.6 Hz, 3H) ppm.

**$^{13}$C NMR** (101 MHz, $CDCl_3$): δ = 168.9, 168.5, 134.8, 131.8, 124.1, 66.8, 59.3, 53.1, 20.3 ppm.

**UP-LCMS:** $R_f$ = 1.300 min, m/z = 264 $[M+H]^+$.

## Boc-3-Hyp-OMe

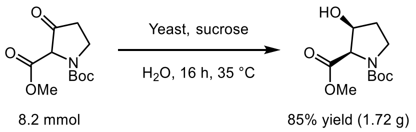


Procedure adapted from literature.[18] 1-*O*-tert-butyl 2-*O*-methyl 3-oxopyrrolidine-1,2-dicarboxylate (2.02 g, 8.2 mmol), D-sucrose (42.2 g) and yeast from *Saccharomyces cerevisiae* (25.7 g) were added to 250 ml $H_2O$. The resulting suspension was heated to 35 °C with a heating block and stirred for 16 h, centrifuged and filtered. The filtrate was extracted with ethyl acetate (4 × 200 ml). The combined organic layer was washed with brine, dried over $MgSO_4$, filtered, and concentrated to afford a pale-yellow oil. The crude reaction mixture was purified by reverse phase flash column chromatography (MeCN:$H_2O$ = 20:80 → 35:65) to yield the product as a clear oil (1.72 g, 7.0 mmol, 85% yield). An *e.e.* of 80% is reported in literature.[18]

**$^1$H NMR** (400 MHz, $CDCl_3$): δ = 4.64 – 4.53 (m, 1H), 4.41 (d, *J* = 6.7 Hz, 0.4H), 4.34 (d, *J* = 6.6 Hz, 0.6H), 3.75 (s, 3H), 3.70 – 3.55 (m, 1H), 3.54 – 3.38 (m, 1H), 2.59 (s, 1H), 2.05 (ddt, *J* = 28.0, 12.7, 6.4 Hz, 2H), 1.44 (s, 3.5H), 1.40 (s, 5.5H) ppm. Summary of rotamers.

**$^{13}$C NMR** (101 MHz, $CDCl_3$): δ = 171.1, 171.0, 154.5, 154.0, 80.4, 80.3, 72.5, 71.6, 64.1, 63.4, 52.3, 52.2, 44.4, 43.9, 32.9, 32.4, 28.5, 28.4 ppm. Summary of rotamers.

**UP-LCMS:** $R_f$ = 1.246 min, m/z = 268 $[M+Na]^+$.

# 9. General procedure

## Procedure A for the Deoxygenative Arylation of Boc-Thr-OMe

A 20 mL vial was charged with NHC-1 (316.3 mg, 0.80 mmol, 1.6 equiv) and a magnetic stir bar. After the vial was vacuumed and refilled with nitrogen gas thrice, Boc-Thr-OMe (203.5 mg, 0.875 mmol, 1.7 equiv) in dry MTBE (4.0 mL) was added and the mixture was stirred at room temperature for 5 min. Then, a pyridine solution (63.5 mg, 0.80 mmol, 1.6 equiv) in dry MTBE (1.0 mL) was added dropwise at room temperature over the course of 1 min. The resulting suspension was stirred at r.t. for 2 h, during which an orange color appeared. Another 20 mL vial was charged with Ir(FMeppy)$_2$(dtbbpy)PF$_6$ (7.4 mg, 7.5 µmol, 0.015 equiv), NiBr$_2$•dtbbpy (12.2 mg, 25 µmol, 0.05 equiv), quinuclidine (96.8 mg, 0.875 mmol, 1.75 equiv), arylhalide (0.5 mmol, 1.0 equiv) and a magnetic stir bar. Dry acetone (5.0 mL) was added to this vial under an atmosphere of nitrogen. 5 ml of the NHC suspension was transferred to a syringe under air. Then a syringe filter and new needle were installed on the syringe, before the NHC suspension was injected through the syringe filter into the other solution. The reaction mixture was sparged with nitrogen for 10 minutes before sealing with parafilm. The vial was stirred at 1000 rpm stir rate and irradiated with 450 nm LEDs at 100% light intensity with maxed fan speed of 6800 rpm in a Penn PhD M2 Integrated Photoreactor for 2 hours. The solvent was evaporated *in vacuo* and the residue was purified by silica flash column chromatography or reverse phase flash chromatography.

## Procedure B for the Deoxygenative Arylation of Boc-3-Hyp-OMe

A 20 mL vial was charged with NHC-1 (316.3 mg, 0.80 mmol, 1.6 equiv) and a magnetic stir bar. After the vial was vacuumed and refilled with nitrogen gas thrice, Boc-3-Hyp-OMe (214.6 mg, 0.875 mmol, 1.7 equiv) in dry MTBE (4.0 mL) was added and the reaction stirred at r.t. for 5 min. Then, a pyridine solution (63.5 mg, 0.80 mmol, 1.6 equiv) in dry MTBE (1.0 mL) was added dropwise at room temperature over the course of 1 min. The resulting suspension was stirred at r.t. for 2 h, during which an orange color appeared. Another 20 mL vial was charged with Ir(FMeppy)$_2$(dtbbpy)PF$_6$ (7.4 mg, 7.5 µmol, 0.015 equiv), NiBr$_2$•dtbbpy (12.2 mg, 25 µmol, 0.05 equiv), quinuclidine (96.8 mg, 0.875 mmol, 1.75 equiv), arylhalide (0.5 mmol, 1.0 equiv) and a magnetic stir bar. Dry acetone (5.0 mL) was added to this vial under an atmosphere of nitrogen. 5 ml of the NHC suspension was transferred to a syringe under air. Then a syringe filter and new needle were installed on the syringe, before the NHC suspension was injected through the syringe filter into the other solution. The reaction mixture was sparged with nitrogen for 10 minutes before sealing with parafilm. The vial was stirred at 1000 rpm stir rate and irradiated with 450 nm LEDs at 100% light intensity with maxed fan speed of 6800 rpm in a Penn PhD M2 Integrated Photoreactor for 2 hours. The solvent was evaporated *in vacuo* and the residue was purified by silica flash column chromatography or reverse phase flash chromatography.

## 10. Synthesis and Characterization of β-Me-Branched α-Amino Acids

### Methyl 2-((*tert*-butoxycarbonyl)amino)-3-phenylbutanoate (3)

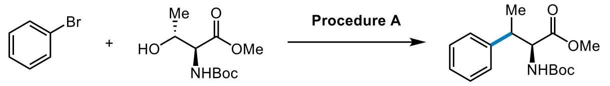


Bromobenzene (78.8 mg, 0.5 mmol) was used with general procedure A. The crude reaction mixture was purified by reverse phase flash column chromatography (MeCN:$H_2O$ = 50:50 → 65:35) to yield the product as a mixture of two diastereomers as clear oil (87.0 mg, 0.30 mmol, 59% yield, 1.7:1 *d.r.*).

**$^1$H NMR** (400 MHz, DMSO-$d_6$): δ = 7.33 (d, *J* = 8.7 Hz, ≈0.3H), 7.31 – 7.15 (m, 5H), 6.95 (d, *J* = 8.5 Hz, ≈0.7H), 4.19 (t, *J* = 9.1 Hz, 1H), 3.65 (s, 2H), 3.37 (s, 1H), 3.21 – 2.96 (m, 1H), 1.35 (s, 3H), 1.26 (s, 6H), 1.24 (d, *J* = 7.0 Hz, 1H), 1.16 (d, *J* = 7.1 Hz, 2H) ppm. Sum of diastereomers.

**$^{13}$C NMR** (101 MHz, DMSO-$d_6$): δ = 172.4, 172.1, 155.5, 155.1, 142.8, 142.4, 128.1, 127.6, 127.5, 126.6, 126.4, 78.3, 78.2, 59.7, 59.2, 51.5, 51.3, 40.9, 40.6, 28.1, 28.0, 18.6, 16.9 ppm. Sum of diastereomers.

**HRMS** (ESI+) m/z: calcd. for $C_{16}H_{23}N_1O_4Na$ $[M+Na]^+$ 316.1519, found 316.1519.

### Methyl 2-((*tert*-butoxycarbonyl)amino)-3-(4-methylphenyl)butanoate (4)

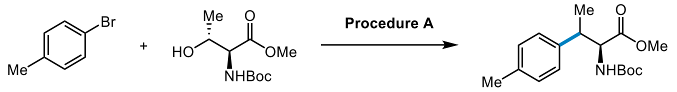


4-Bromotoluene (86 mg, 0.5 mmol) was used with general procedure A. The crude reaction mixture was purified by reverse phase flash column chromatography (MeCN:$H_2O$ = 50:50 → 75:25) to yield the product as a mixture of two diastereomers as clear oil (95.4 mg, 0.31 mmol, 62% yield, 1.6:1 *d.r.*).

**$^1$H NMR** (400 MHz, DMSO-$d_6$): δ = 7.35 (d, J = 8.6 Hz, ≈0.3H), 7.18 – 7.04 (m, 4H), 6.93 (d, J = 8.5 Hz, ≈0.7H), 4.13 (td, J = 8.5, 3.7 Hz, 1H), 3.64 (s, 2H), 3.38 (s, 1H), 3.13 – 2.97 (m, 1H), 2.25 (s, 3H), 1.35 (s, 3H), 1.27 (s, 6H), 1.20 (d, J = 7.1 Hz, 1H), 1.13 (d, J = 7.1 Hz, 2H) ppm. Sum of diastereomers.

**$^{13}$C NMR** (101 MHz, DMSO-$d_6$): δ = 172.5, 172.2, 155.6, 155.2, 139.8, 139.3, 135.6, 135.5, 128.7, 127.4, 127.4, 78.3, 59.8, 59.4, 51.6, 51.3, 40.4, 28.2, 28.0, 20.6, 20.6, 18.7, 17.1 ppm. Sum of diastereomers.

**HRMS** (ESI+) m/z: calcd. for $C_{17}H_{25}N_1O_4Na$ $[M+Na]^+$ 330.1676, found 330.1675.

### Methyl 2-((*tert*-butoxycarbonyl)amino)-3-(4-cyclopropylphenyl)butanoate (5)

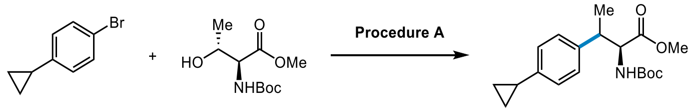


1-Bromo-4-cyclopropylbenzene (98.6 mg, 0.5 mmol) was used with general procedure A. The crude reaction mixture was purified by reverse phase flash column chromatography (MeCN:$H_2O$ = 60:40 → 70:30) to yield the product as a mixture of two diastereomers as clear oil (128 mg, 0.38 mmol, 77% yield, 1.7:1 *d.r.*).

**$^1$H NMR** (400 MHz, DMSO-$d_6$): δ = 7.29 (d, *J* = 8.6 Hz, ≈0.3H), 7.11 (d, *J* = 8.3 Hz, ≈1H), 7.07 (d, *J* = 8.3 Hz, ≈1H), 6.97 (d, *J* = 6.8 Hz, 2H), 6.87 (d, *J* = 8.5 Hz, ≈0.7H), 4.13 (td, *J* = 8.6, 5.9 Hz, 1H), 3.64 (s, 2H), 3.39 (s, 1H), 3.14 – 2.96 (m, 1H), 1.86 (tt, *J* = 8.4, 5.1 Hz, 1H), 1.35 (s, 3H), 1.27 (s, 6H), 1.20 (d, *J* = 7.0 Hz, 1H), 1.12 (d, *J* = 7.1 Hz, 2H), 0.95 – 0.84 (m, 2H), 0.68 – 0.57 (m, 2H) ppm. Sum of diastereomers.

**$^{13}$C NMR** (101 MHz, DMSO-$d_6$): δ = 172.4, 172.1, 155.5, 155.1, 141.8, 141.7, 139.6, 139.2, 127.4, 127.4, 125.1, 125.1, 78.2, 59.7, 59.3, 51.5, 51.3, 40.4, 40.1, 28.1, 28.0, 18.6, 16.9, 14.6, 9.1, 9.1, 9.0 ppm. Sum of diastereomers.

**GC-HRMS** ($R_f$ = 14.80 min) (CI) m/z: calcd. for $C_{19}H_{28}N_1O_4$ $[M+H]^+$ 334.2013, found 334.2014.

**GC-HRMS** ($R_f$ = 14.90 min) (CI) m/z: calcd. for $C_{19}H_{28}N_1O_4$ $[M+H]^+$ 334.2013, found 334.2017.

## Methyl 3-([1,1'-biphenyl]-4-yl)-2-((*tert*-butoxycarbonyl)amino)butanoate (6)

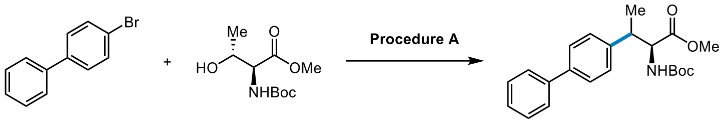


1-Bromo-4-phenylbenzene (118 mg, 0.5 mmol) was used with general procedure A. The crude reaction mixture was purified by reverse phase flash column chromatography (MeCN:$H_2O$ = 60:40 → 75:25) to yield the product as a mixture of two diastereomers as clear oil (145 mg, 0.39 mmol, 78% yield, 1.6:1 *d.r.*).

**$^{1}$H NMR** (400 MHz, DMSO-$d_6$): δ = 7.63 (dd, *J* = 6.9, 1.6 Hz, 2H), 7.58 (dd, *J* = 8.3, 2.6 Hz, 2H), 7.45 (t, *J* = 7.7 Hz, 2H), 7.41 – 7.33 (m, ≈2.3H), 7.31 (d, *J* = 8.4 Hz, 1H), 7.04 (d, *J* = 8.5 Hz, ≈0.6H), 4.23 (q, *J* = 9.0 Hz, 1H), 3.67 (s, 2H), 3.43 (s, 1H), 3.20 (p, *J* = 7.0 Hz, ≈0.4H), 3.16 – 3.05 (m, ≈0.6H), 1.36 (s, 3H), 1.28 – 1.24 (m, 7H), 1.20 (d, *J* = 7.1 Hz, 2H) ppm. Sum of diastereomers.

**$^{13}$C NMR** (101 MHz, DMSO-$d_6$): δ = 172.4, 172.0, 155.6, 155.2, 142.1, 141.6, 140.0, 139.9, 138.4, 138.4, 128.8, 128.2, 128.1, 127.2, 127.2, 126.5, 126.5, 126.4, 126.4, 78.3, 78.3, 59.5, 59.2, 51.6, 51.4, 40.4, 40.3, 28.1, 28.0, 18.5, 16.8 ppm. Sum of diastereomers.

**HRMS** (ESI+) m/z: calcd. for $C_{22}H_{27}N_1O_4Na$ $[M+Na]^+$ 392.1832, found 392.1832.

## Methyl 2-((*tert*-butoxycarbonyl)amino)-3-(4-methoxyphenyl)butanoate (7)

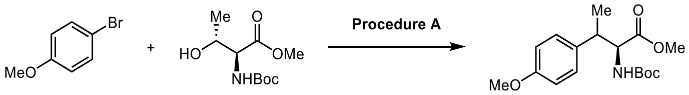


4-Bromoanisole (93.1 mg, 0.5 mmol) was used with general procedure A. The crude reaction mixture was purified by reverse phase flash column chromatography (MeCN:$H_2O$ = 50:50 → 70:30) to yield the product as a mixture of two diastereomers as clear oil (109 mg, 0.34 mmol, 67% yield, 1.6:1 *d.r.*).

**$^{1}$H NMR** (400 MHz, DMSO-$d_6$): δ = 7.34 (d, *J* = 8.5 Hz, ≈0.3H), 7.17 (d, *J* = 8.8 Hz, 1H), 7.12 (d, *J* = 8.8 Hz, 1H), 6.92 (d, *J* = 8.3 Hz, ≈0.6H), 6.83 (dd, *J* = 8.6, 1.6 Hz, 2H), 4.15 – 4.05 (m, 1H), 3.71 (s, 3H), 3.64 (s, 2H), 3.38 (s, 1H), 3.12 – 2.95 (m, 1H), 1.35 (s, 3H), 1.27 (s, 6H), 1.20 (d, *J* = 7.1 Hz, 1H), 1.12 (d, *J* = 7.1 Hz, 2H) ppm. Sum of diastereomers.

**$^{13}$C NMR** (101 MHz, DMSO-$d_6$): δ = 172.6, 172.2, 157.9, 157.9, 155.6, 155.2, 134.7, 134.2, 128.6, 113.5, 78.3, 78.3, 59.9, 59.5, 55.0, 51.6, 51.4, 40.0, 28.2, 28.0, 18.7, 17.2 ppm. Sum of diastereomers.

**GC-HRMS** ($R_f$ = 14.17 min) (CI) m/z: calcd. for $C_{17}H_{26}N_1O_5$ $[M+H]^+$ 324.1805, found 324.1804.

**GC-HRMS** ($R_f$ = 14.26 min) (CI) m/z: calcd. for $C_{17}H_{26}N_1O_5$ $[M+H]^+$ 324.1805, found 324.1805.

## Ethyl 4-(3-((*tert*-butoxycarbonyl)amino)-4-methoxy-4-oxobutan-2-yl)benzoate (8)

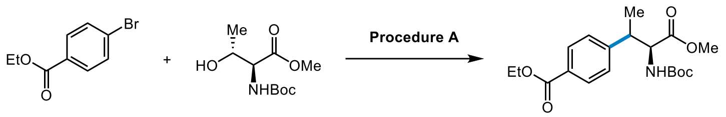


Ethyl 4-bromobenzoate (115.1, 0.5 mmol) was used with general procedure A. The crude reaction mixture was purified by silica flash chromatography (EtOAc:cyclohexane = 0:100 → 15:85) to yield the product as a mixture of two diastereomers as clear oil (157 mg, 0.43 mmol, 86% yield, 1.5:1 *d.r.*). Analysis was performed on minor pure fractions.

**Diastereomer 1:**

**$^1$H NMR** (400 MHz, DMSO-d$_6$): δ = 7.87 (d, *J* = 8.4 Hz, 2H), 7.41 (d, *J* = 8.4 Hz, 2H), 7.10 (d, *J* = 8.6 Hz, 1H), 4.29 (q, *J* = 7.1 Hz, 2H), 4.22 (t, *J* = 9.1 Hz, 1H), 3.66 (s, 3H), 3.17 (p, *J* = 7.1 Hz, 1H), 1.30 (t, *J* = 7.1 Hz, 3H), 1.24 (s, 9H), 1.18 (d, *J* = 7.1 Hz, 3H) ppm.

**$^{13}$C NMR** (101 MHz, DMSO-d$_6$): δ = 172.1, 165.6, 155.1, 148.5, 129.0, 128.2, 128.0, 78.3, 60.5, 58.9, 51.6, 40.7, 28.0, 18.2, 14.1 ppm.

**HRMS** (ESI+) m/z: calcd. for $C_{19}H_{27}N_1O_6Na$ [M+Na]$^+$ 388.1731, found 388.1728.

**Diastereomer 2:**

**$^1$H NMR** (400 MHz, DMSO-d$_6$): δ = 7.87 (d, *J* = 8.4 Hz, 2H), 7.41 (d, *J* = 8.9 Hz, 1H), 7.37 (d, *J* = 8.4 Hz, 2H), 4.30 (q, *J* = 7.1 Hz, 2H), 4.23 (t, *J* = 8.4 Hz, 1H), 3.40 (s, 3H), 3.23 (p, *J* = 7.3 Hz, 1H), 1.34 (s, 9H), 1.31 (t, *J* = 7.1 Hz, 3H), 1.25 (d, *J* = 7.1 Hz, 3H) ppm.

**$^{13}$C NMR** (101 MHz, DMSO-*d*$_6$): δ = 171.8, 165.6, 155.5, 148.0, 129.0, 128.3, 128.0, 78.4, 60.6, 59.2, 51.5, 40.8, 28.1, 16.6, 14.1 ppm.

**HRMS** (ESI+) m/z: calcd. for $C_{19}H_{27}N_1O_6Na$ [M+Na]$^+$ 388.1731, found 388.1728.

## Hexyl 4-(3-((*tert*-butoxycarbonyl)amino)-4-methoxy-4-oxobutan-2-yl)benzoate (9)

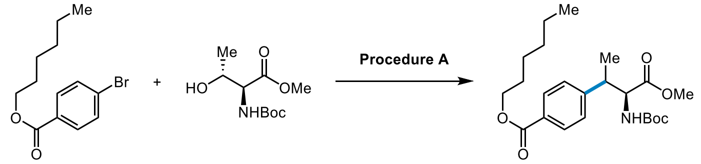


Hexyl 4-bromobenzoate (143 mg, 0.5 mmol) was used with general procedure A. The crude reaction mixture was purified by silica flash chromatography (EtOAc:cyclohexane = 0:100 → 10:90), followed by reverse phase flash column chromatography (MeCN:$H_2O$ = 60:40 → 90:10), to yield the product as a mixture of two diastereomers as yellow oil (144 mg, 0.34 mmol, 68% yield, 1.7:1 *d.r.*).

**$^1$H NMR** (400 MHz, DMSO-*d*$_6$): δ = 7.86 (dd, *J* = 8.3, 2.3 Hz, 2H), 7.40 (d, *J* = 8.2 Hz, ≈1.3H), 7.36 (d, *J* = 8.3 Hz, 1H), 7.06 (d, *J* = 8.7 Hz, ≈0.7H), 4.23 (t, *J* = 6.6 Hz, 3H), 3.65 (s, 2H), 3.41 (s, 1H), 3.25 (p, *J* = 6.5 Hz, ≈0.4H), 3.17 (p, *J* = 7.9 Hz, ≈0.6H), 1.67 (p, *J* = 6.8 Hz, 2H), 1.42 – 1.25 (m, 10H), 1.23 (s, 6H), 1.18 (d, *J* = 7.1 Hz, 2H), 0.90 – 0.80 (m, 3H) ppm. Sum of diastereomers.

**$^{13}$C NMR** (101 MHz, DMSO-$d_6$): δ = 172.0, 171.8, 165.6, 165.6, 155.5, 155.1, 148.4, 148.0, 129.0, 128.9, 128.3, 128.2, 128.0, 128.0, 78.3, 78.2, 64.5, 64.4, 59.2, 58.9, 51.6, 51.4, 40.9, 40.8, 30.9, 28.1, 28.1, 28.0, 27.9, 25.1, 22.0, 18.1, 16.4, 13.7 ppm. Sum of diastereomers.

**HRMS** (ESI+) m/z: calcd. for $C_{23}H_{35}N_1O_6Na$ $[M+Na]^+$ 444.2357, found 444.2357.

## Methyl 3-(4-acetylphenyl)-2-((*tert*-butoxycarbonyl)amino)butanoate (10)

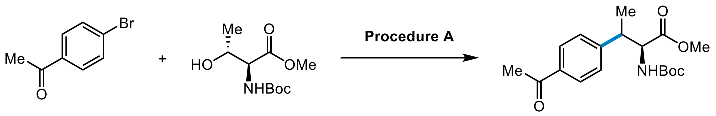


4-Bromoacetophenone (101 mg, 0.5 mmol) was used with general procedure A. The crude reaction mixture was purified by reverse phase flash column chromatography (MeCN:$H_2O$ = 40:60 → 65:35) to yield the product as a mixture of two diastereomers as clear oil (140 mg, 0.42 mmol, 83% yield, 1.6:1 *d.r.*).

**$^1$H NMR** (500 MHz, DMSO-$d_6$): δ = 7.87 (dd, J = 8.4, 1.6 Hz, 2H), 7.45 (d, J = 8.7 Hz, ≈0.5H), 7.41 (d, J = 8.1 Hz, 1H), 7.37 (d, J = 8.3 Hz, 1H), 7.15 (d, J = 8.5 Hz, ≈0.5H), 4.28 – 4.17 (m, 1H), 3.66 (s, ≈1.8H), 3.41 (s, ≈1.2H), 3.23 (p, J = 7.1 Hz, ≈0.4H), 3.16 (dq, J = 9.8, 7.1 Hz, ≈0.6H), 2.56 – 2.52 (m, 3H), 1.34 (s, 3H), 1.27 – 1.21 (m, 7H), 1.17 (d, J = 7.1 Hz, 2H) ppm. Sum of diastereomers.

**$^{13}$C NMR** (126 MHz, DMSO-$d_6$): δ = 197.6, 197.5, 172.3, 171.9, 155.6, 155.2, 148.5, 148.1, 135.4, 135.3, 128.2, 128.2, 128.0, 128.0, 78.4, 78.4, 59.2, 59.0, 51.7, 51.5, 40.8, 40.6, 28.1, 28.0, 26.7, 18.3, 16.6 ppm. Sum of diastereomers.

**HRMS** (ESI+) m/z: calcd. for $C_{18}H_{25}N_1O_5Na$ $[M+Na]^+$ 358.1625, found 358.1624.

## Methyl 2-((*tert*-butoxycarbonyl)amino)-3-(4-formylphenyl)butanoate (11)

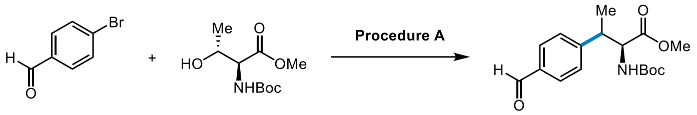


4-Bromobenzaldehyde (92.3 mg, 0.5 mmol) was used with general procedure A. The crude reaction mixture was purified by reverse phase flash column chromatography (MeCN:$H_2O$ = 40:60 → 70:30) to yield the product as a mixture of two diastereomers as clear oil (117 mg, 0.36 mmol, 73% yield, 1.3:1 *d.r.*).

**$^1$H NMR** (400 MHz, DMSO-$d_6$): δ = 9.97 (s, 1H), 7.83 (d, *J* = 8.1 Hz, 2H), 7.50 (d, *J* = 8.1 Hz, 1H), 7.46 (d, *J* = 8.1 Hz, 1H), 7.42 (d, *J* = 8.8 Hz, ≈0.3H), 7.15 (d, *J* = 8.8 Hz, ≈0.6H), 4.26 (q, *J* = 9.1 Hz, 1H), 3.66 (s, 2H), 3.41 (s, 1H), 3.26 (q, *J* = 6.9 Hz, ≈0.4H), 3.23 – 3.10 (m, ≈0.6H), 1.34 (s, 3H), 1.26 (d, *J* = 7.0 Hz, 1H), 1.24 (s, 6H), 1.19 (d, *J* = 7.1 Hz, 2H) ppm. Sum of diastereomers.

**$^{13}$C NMR** (101 MHz, DMSO-$d_6$): δ = 192.6, 172.0, 171.8, 155.5, 155.1, 150.1, 149.6, 134.9, 134.8, 129.4, 129.4, 128.5, 128.5, 78.4, 78.3, 59.1, 58.9, 51.6, 51.5, 41.0, 40.9, 28.1, 27.9, 18.2, 16.5 ppm. Sum of diastereomers.

**HRMS** (ESI+) m/z: calcd. for $C_{17}H_{23}N_1O_5Na$ $[M+Na]^+$ 344.1468, found 344.1466.

## Methyl 2-((*tert*-butoxycarbonyl)amino)-3-(4-cyanophenyl)butanoate (12)

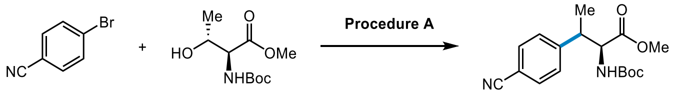

4-Bromobenzonitrile (90.5 mg, 0.5 mmol) was used with general procedure A. The crude reaction mixture was purified by reverse phase flash column chromatography (MeCN:$H_2O$ = 40:60 → 65:35) to yield the product as a mixture of two diastereomers as clear oil (109 mg, 0.34 mmol, 69% yield, 1.5:1 *d.r.*).

**$^{1}$H NMR** (500 MHz, DMSO-$d_6$): δ = 7.75 (d, *J* = 8.0 Hz, 2H), 7.54 – 7.41 (m, ≈2.6H), 7.22 (d, *J* = 8.8 Hz, ≈0.5H), 4.28 – 4.23 (m, ≈0.5H), 4.23 – 4.17 (m, ≈0.3H), 3.66 (s, ≈1.2), 3.43 (s, ≈1.9H), 3.26 (p, *J* = 7.1 Hz, ≈0.6H), 3.16 (dq, *J* = 10.1, 7.1 Hz, ≈0.4H), 1.33 (s, ≈5H), 1.28 – 1.19 (m, ≈6H), 1.16 (d, *J* = 7.0 Hz, ≈1.2H) ppm. Sum of diastereomers.

**$^{13}$C NMR** (126 MHz, DMSO-$d_6$): δ = 172.1, 171.8, 155.6, 155.1, 148.8, 148.4, 132.1, 132.0, 129.0, 128.9, 118.9, 118.9, 109.5, 109.4, 78.7, 78.5, 78.4, 59.0, 58.8, 51.8, 51.6, 40.9, 28.1, 28.0, 18.1, 16.2 ppm. Sum of diastereomers.

**HRMS** (ESI+) m/z: calcd. for $C_{17}H_{22}N_2O_4Na$ $[M+Na]^+$ 341.1472, found 341.1472.

## Methyl 2-((*tert*-butoxycarbonyl)amino)-3-(4-fluorophenyl)butanoate (13)

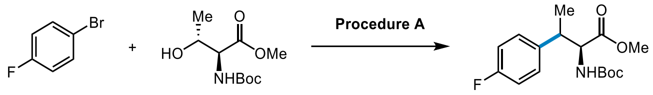


Bromo-4-fluorobenzene (89.2 mg, 0.5 mmol) was used with general procedure A. The crude reaction mixture was purified by reverse phase flash column chromatography (MeCN:$H_2O$ = 40:60 → 75:25) to yield the product as a mixture of two diastereomers as clear oil (111 mg, 0.36 mmol, 71% yield, 1.5:1 *d.r.*).

**$^{1}$H NMR** (400 MHz, DMSO-$d_6$): δ = 7.37 (d, *J* = 8.8 Hz, ≈0.3H), 7.33 – 7.20 (m, 2H), 7.09 (t, *J* = 8.9 Hz, 2H), 7.05 (d, *J* = 8.6 Hz, ≈0.7H), 4.15 (q, *J* = 8.5 Hz, 1H), 3.65 (s, 2H), 3.40 (s, 3H), 3.16 (q, *J* = 7.3 Hz, ≈0.4H), 3.11 – 2.99 (m, ≈0.6H), 1.35 (s, 3H), 1.26 (s, 6H), 1.22 (d, *J* = 7.0 Hz, 1H), 1.14 (d, *J* = 7.1 Hz, 2H) ppm. Sum of diastereomers.

**$^{13}$C NMR** (101 MHz, DMSO-$d_6$): δ = 172.3, 172.0, 161.0 (d, *J* = 242.2 Hz), 160.9 (d, *J* = 242.0 Hz), 155.5, 155.1, 138.9 (d, *J* = 3.0 Hz), 138.4 (d, *J* = 3.2 Hz), 129.5 (d, *J* = 7.9 Hz), 129.4 (d, *J* = 8.1 Hz), 114.8 (d, *J* = 21.0 Hz), 114.7 (d, *J* = 21.1 Hz), 78.3, 78.3, 59.6, 59.3, 51.6, 51.4, 40.1, 40.0, 28.1, 28.0, 18.5, 16.9 ppm. Sum of diastereomers.

**$^{19}$F NMR** (376 MHz, DMSO-$d_6$): δ = -116.38 (td, *J* = 9.0, 4.5 Hz), -116.70 (tt, *J* = 9.0, 5.2 Hz) ppm. Sum of diastereomers.

**GC-HRMS** ($R_f$ = 12.64 min) (CI) m/z: calcd. for $C_{16}H_{23}N_1O_4F$ $[M+H]^+$ 312.1606, found 312.1603.

**GC-HRMS** ($R_f$ = 12.71 min) (CI) m/z: calcd. for $C_{16}H_{23}N_1O_4F$ $[M+H]^+$ 312.1606, found 312.1604.

## Methyl 2-((*tert*-butoxycarbonyl)amino)-3-(4-(trifluoromethyl)phenyl)butanoate (14)

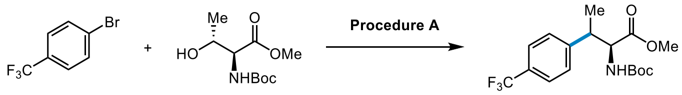


1-Bromo-4-(trifluoromethyl)benzene (113 mg, 0.5 mmol) was used with general procedure A. The crude reaction mixture was purified by reverse phase flash column chromatography (MeCN:$H_2O$ = 50:50 → 65:35) to yield the product as a mixture of two diastereomers as clear oil (163 mg, 0.45 mmol, 90% yield, 2.0:1 *d.r.*).

The reaction was upscaled to twice the scale using 1-bromo-4-(trifluoromethyl)benzene (226 mg, 1.0 mmol) with general procedure A. The crude reaction mixture was purified by reverse phase flash column chromatography (MeCN:$H_2O$ = 50:50 → 65:35) to yield the product as a mixture of two diastereomers as clear oil (257 mg, 0.71 mmol, 71% yield, 2.0:1 *d.r.*).

**$^1$H NMR** (500 MHz, DMSO-$d_6$): δ = 7.64 – 7.56 (m, 2H), 7.50 (d, *J* = 8.1 Hz, ≈1.2H), 7.47 – 7.40 (m, ≈1.2H), 7.15 (d, *J* = 8.8 Hz, ≈0.6H), 4.32 (t, *J* = 8.3 Hz, ≈0.3H), 4.27 (t, *J* = 9.3 Hz, ≈0.5H), 3.66 (s, ≈1.7H), 3.43 (s, ≈1.2H), 3.31 (p, *J* = 7.3 Hz, ≈0.4H), 3.25 – 3.16 (m, ≈0.6H), 1.32 (s, 3H), 1.26 (d, *J* = 7.1 Hz, ≈1.2H), 1.22 (s, 5H), 1.19 (d, *J* = 6.9 Hz, 2H) ppm. Sum of diastereomers.

**$^{13}$C NMR** (126 MHz, DMSO-$d_6$): δ = 172.1, 171.8, 155.6, 155.2, 147.7, 147.3, 128.7, 128.6, 127.6 (q, *J* = 31.7 Hz), 127.5 (q, *J* = 31.6 Hz), 124.9 (dq, *J* = 7.9, 3.7 Hz), 124.4 (q, *J* = 271.6 Hz), 124.4 (q, *J* = 271.6 Hz), 78.4, 78.3, 59.2, 59.0, 51.6, 51.4, 40.9, 28.0, 27.9, 18.2, 16.2 ppm. Sum of diastereomers.

**$^{19}$F NMR** (470 MHz, DMSO-$d_6$): δ = -61.32 – -61.40 (m) ppm. Sum of diastereomers.

**HRMS** (ESI+) m/z: calcd. for $C_{17}H_{22}N_1O_4F_3Na$ $[M+Na]^+$ 384.1393, found 384.1392.

## Methyl 2-((*tert*-butoxycarbonyl)amino)-3-(4-hydroxyphenyl)butanoate (15)

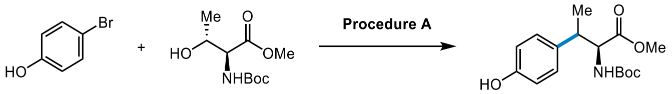


4-Bromophenol (86.8 mg, 0.5 mmol) was used with general procedure A. The crude reaction mixture was purified by reverse phase flash column chromatography (MeCN:$H_2O$ = 30:70 → 45:55) to yield the product as a mixture of two diastereomers as colorless powder (58.2 mg, 0.19 mmol, 38% yield, 2.3:1 *d.r.*).

**$^1$H NMR** (400 MHz, DMSO-d$_6$): δ = 9.18 (s, 1H), 7.25 (d, *J* = 8.6 Hz, ≈0.2H), 7.03 (d, *J* = 8.3 Hz, ≈1.4H), 6.98 (d, *J* = 8.5 Hz, ≈0.6H), 6.77 (d, *J* = 8.4 Hz, ≈0.7H), 6.69 – 6.61 (m, 2H), 4.08 (t, *J* = 8.8 Hz, 1H), 3.63 (s, 2H), 3.38 (s, 1H), 3.04 – 2.91 (m, 1H), 1.36 (s, 2H), 1.28 (s, 7H), 1.18 (d, *J* = 7.0 Hz, 1H), 1.11 (d, *J* = 7.1 Hz, 2H) ppm. Sum of diastereomers.

**$^{13}$C NMR** (101 MHz, DMSO-d$_6$): δ = 172.5, 172.2, 155.9, 155.9, 155.5, 155.2, 132.7, 132.4, 128.4, 114.9, 78.3, 60.0, 59.5, 51.5, 51.3, 40.1, 28.1, 28.0, 18.7, 17.3 ppm. Sum of diastereomers.

**GC-HRMS** ($R_f$ = 14.51 min) (CI) m/z: calcd. for $C_{16}H_{24}N_1O_5$ $[M+H]^+$ 310.1649, found 310.1650.

**GC-HRMS** ($R_f$ = 14.57 min) (CI) m/z: calcd. for $C_{16}H_{24}N_1O_5$ $[M+H]^+$ 310.1649, found 310.1651.

## Methyl 3-(4-acetamidophenyl)-2-((*tert*-butoxycarbonyl)amino)butanoate (16)

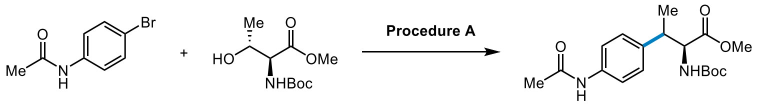


*N*-(4-Bromophenyl)acetamide (107 mg, 0.5 mmol) was used with general procedure A. The crude reaction mixture was purified by reverse phase flash column chromatography (MeCN:$H_2O$ = 30:70 → 45:55) to yield the product as a mixture of two diastereomers as white powder (123 mg, 0.35 mmol, 70% yield, 1.7:1 *d.r.*).

**$^1$H NMR** (400 MHz, DMSO-$d_6$): δ = 9.84 (s, 1H), 7.47 (d, *J* = 8.1 Hz, 2H), 7.30 (d, *J* = 8.7 Hz, ≈0.3H), 7.16 (d, *J* = 8.2 Hz, ≈1.2H), 7.11 (d, *J* = 8.3 Hz, ≈0.8H), 6.88 (d, *J* = 8.4 Hz, ≈0.5H), 4.13 (t, *J* = 8.7 Hz, 1H), 3.64 (s, 2H),

3.39 (s, 1H), 3.05 (dp, $J$ = 15.9, 7.4 Hz, 1H), 2.02 (s, 3H), 1.36 (s, 3H), 1.27 (s, 6H), 1.21 (d, $J$ = 7.0 Hz, 1H), 1.13 (d, $J$ = 7.1 Hz, 2H) ppm. Sum of diastereomers.

**$^{13}$C NMR** (101 MHz, DMSO-$d_6$): δ = 172.4, 172.1, 168.1, 168.0, 155.5, 155.2, 137.8, 137.8, 137.2, 136.8, 127.7, 118.8, 118.8, 78.3, 59.8, 59.3, 51.5, 51.3, 40.3, 40.0, 28.1, 28.0, 23.9, 18.5, 17.0 ppm. Sum of diastereomers.

**HRMS** (ESI+) m/z: calcd. for $C_{18}H_{26}N_2O_5Na$ $[M+Na]^+$ 373.1734, found 373.1733.

## Methyl 2-((*tert*-butoxycarbonyl)amino)-3-(4-(4,4,5,5-tetramethyl-1,3,2-dioxaborolan-2-yl)phenyl)butanoate (17)

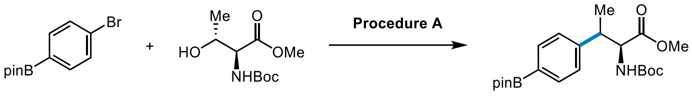


2-(4-Bromophenyl)-4,4,5,5-tetramethyl-1,3,2-dioxaborolane (145 mg, 0.5 mmol) was used with general procedure A. The crude reaction mixture was purified by silica flash chromatography (EtOAc:cyclohexane = 0:100 → 10:90) to yield the product as a mixture of two diastereomers as clear oil (157 mg, 0.37 mmol, 75% yield, 1.8:1 *d.r.*).

**$^{1}$H NMR** (400 MHz, DMSO-$d_6$): δ = 7.59 (d, $J$ = 7.8 Hz, 2H), 7.34 (d, $J$ = 8.7 Hz, ≈0.3H), 7.27 (d, $J$ = 7.7 Hz, ≈1.2H), 7.22 (d, $J$ = 7.8 Hz, ≈0.8H), 6.96 (d, $J$ = 8.5 Hz, ≈0.7H), 4.27 – 4.12 (m, 1H), 3.65 (s, 2H), 3.40 (s, 1H), 3.21 – 2.98 (m, 1H), 1.35 (s, 3H), 1.28 (s, 12H), 1.26 (s, 6H), 1.22 (d, $J$ = 7.0 Hz, 1H), 1.15 (d, $J$ = 7.1 Hz, 2H) ppm. Sum of diastereomers.

**$^{13}$C NMR** (101 MHz, DMSO-$d_6$): δ = 172.2, 171.8, 155.5, 155.1, 146.3, 145.9, 134.4, 134.3, 127.1, 127.1, 83.5, 83.4, 78.3, 78.3, 59.4, 59.1, 51.5, 51.4, 40.9, 40.7, 28.1, 28.0, 24.6, 24.6, 24.6, 18.3, 16.8 ppm. Sum of diastereomers.[1]

**$^{11}$B NMR** (160 MHz, DMSO-$d_6$): δ = 30.5 ppm. Sum of diastereomers.

**HRMS** (ESI+) m/z: calcd. for $C_{22}H_{34}N_1O_6B_1Na$ $[M+Na]^+$ 442.2371, found 442.2371.

## Methyl 3-(4-(but-2-yn-1-yloxy)phenyl)-2-((*tert*-butoxycarbonyl)amino)butanoate (18)

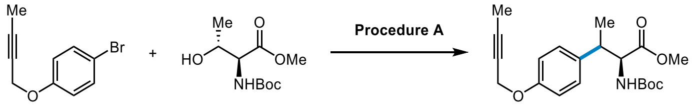


1-Bromo-4-(but-2-yn-1-yloxy)benzene (113 mg, 0.5 mmol) was used with general procedure A. The crude reaction mixture was purified by reverse phase flash column chromatography (MeCN:$H_2O$ = 40:60 → 75:25) to yield the product as a mixture of two diastereomers as clear oil (56.1 mg, 0.16 mmol, 31% yield, 1.6:1 *d.r.*).

**$^{1}$H NMR** (400 MHz, DMSO-$d_6$): δ = 7.29 (d, $J$ = 8.6 Hz, ≈0.3H), 7.17 (d, $J$ = 8.4 Hz, ≈1.2H), 7.13 (d, $J$ = 8.4 Hz, ≈0.8H), 6.87 (d, $J$ = 8.1 Hz, ≈2.7H), 4.68 (q, $J$ = 2.4 Hz, 2H), 4.17 – 4.07 (m, 1H), 3.64 (s, 2H), 3.39 (s, 1H), 3.15 – 2.89 (m, 1H), 1.82 (q, $J$ = 2.3 Hz, 3H), 1.36 (s, 3H), 1.28 (s, 6H), 1.21 (d, $J$ = 7.1 Hz, 1H), 1.13 (d, $J$ = 7.1 Hz, 2H) ppm. Sum of diastereomers.

[1] C($sp^2$)-B carbon signal is not visible.

**$^{13}$C NMR** (101 MHz, DMSO-$d_6$): δ = 172.5, 172.2, 156.1, 156.1, 155.5, 155.2, 135.2, 134.8, 128.5, 128.5, 114.4, 114.3, 83.3, 78.3, 74.9, 74.8, 59.8, 59.4, 55.7, 55.7, 51.5, 51.3, 40.0, 39.8, 28.1, 28.0, 18.6, 17.1, 3.1, 3.1 ppm. Sum of diastereomers.

**HRMS** (ESI+) m/z: calcd. for $C_{20}H_{27}N_1O_5Na$ $[M+Na]^+$ 384.1781, found 384.1780.

## Methyl 2-((*tert*-butoxycarbonyl)amino)-3-(4-(5-(trifluoromethyl)-1H-pyrazol-3-yl)phenyl)butanoate (19)

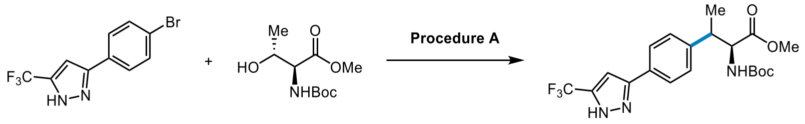


3-(4-Bromophenyl)-5-(trifluoromethyl)-1H-pyrazole (147 mg, 0.5 mmol) was used with general procedure A. The crude reaction mixture was purified by silica flash chromatography (EtOAc:cyclohexane = 0:100 → 25:75) to yield the product as a mixture of two diastereomers as colorless solid (105 mg, 0.24 mmol, 49% yield, 1.7:1 *d.r.*).

**$^{1}$H NMR** (400 MHz, DMSO-$d_6$): δ = 14.00 (s, 1H), 7.74 (d, *J* = 7.1 Hz, 2H), 7.38 (d, *J* = 8.0 Hz, ≈1.4H), 7.34 (d, *J* = 8.2 Hz, 1H), 7.12 (s, 1H), 7.06 (d, *J* = 8.6 Hz, ≈0.7H), 4.29 – 4.18 (m, 1H), 3.66 (s, 2H), 3.42 (s, 1H), 3.26 – 3.17 (m, ≈0.4H), 3.17 – 3.02 (m, ≈0.7H), 1.34 (s, 3H), 1.30 – 1.22 (m, 7H), 1.19 (d, *J* = 7.1 Hz, 2H) ppm. Sum of diastereomers.

**$^{13}$C NMR** (101 MHz, DMSO-$d_6$): δ = 172.3, 172.0, 155.6, 155.2, 144.0, 143.9, 143.8, 143.4, 142.1 (q, *J* = 36.8 Hz), 128.4, 128.4, 126.5, 126.4, 125.4, 121.8 (q, *J* = 268.1 Hz), 100.7, 78.4, 78.3, 59.4, 59.1, 51.6, 51.4, 40.6, 40.5, 28.1, 28.0, 26.3, 18.3, 16.6 ppm. Sum of diastereomers.

**$^{19}$F NMR** (376 MHz, DMSO-$d_6$): δ = -60.5, -60.6 ppm. Sum of diastereomers.

**HRMS** (ESI+) m/z: calcd. for $C_{20}H_{24}N_3O_4F_3Na$ $[M+Na]^+$ 450.1611, found 450.1610.

## Methyl 2-((*tert*-butoxycarbonyl)amino)-3-(naphthalen-2-yl)butanoate (20)

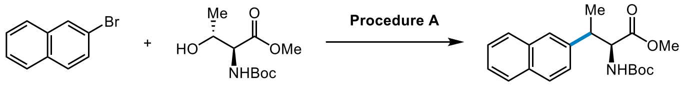


2-Bromonaphthaline (105 mg, 0.5 mmol) was used with general procedure A. The crude reaction mixture was purified by reverse phase flash column chromatography (MeCN:$H_2O$ = 50:50 → 75:25) to yield the product as a mixture of two diastereomers as clear oil (137 mg, 0.40 mmol, 80% yield, 1.6:1 *d.r.*).

**$^{1}$H NMR** (400 MHz, DMSO-$d_6$): δ = 7.90 – 7.78 (m, 3H), 7.79 – 7.67 (m, 1H), 7.53 – 7.37 (m, ≈3.3H), 7.12 (d, *J* = 8.5 Hz, ≈0.7H), 4.37 – 4.25 (m, 1H), 3.67 (s, 2H), 3.35 (s, 1H), 3.33 – 3.17 (m, 1H), 1.36 – 1.31 (m, 4H), 1.25 (d, *J* = 7.1 Hz, 2H), 1.20 (s, 6H) ppm. Sum of diastereomers.

**$^{13}$C NMR** (101 MHz, DMSO-$d_6$): δ = 172.5, 172.1, 155.6, 155.2, 140.5, 140.1, 132.9, 132.0, 127.6, 127.6, 127.4, 126.2, 126.1, 126.0, 126.0, 125.9, 125.6, 125.5, 78.4, 78.3, 59.6, 59.2, 51.6, 51.4, 41.0, 40.7, 28.1, 28.0, 18.7, 16.9 ppm. Sum of diastereomers.

**HRMS** (ESI+) m/z: calcd. for $C_{20}H_{25}NO_4Na$ $[M+Na]^+$ 366.1676, found 366.1675.

## Methyl 2-((*tert*-butoxycarbonyl)amino)-3-(2-methylphenyl)butanoate (21)

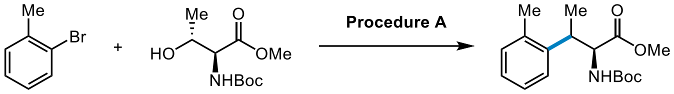


1-Bromo-2-methylbenzene (86.8 mg, 0.5 mmol) was used with general procedure A. The crude reaction mixture was purified by reverse phase flash column chromatography (MeCN:$H_2O$ = 50:50 → 70:30) to yield the product as a mixture of two diastereomers as clear oil (36.5 mg, 0.12 mmol, 24 % yield, 1.7:1 *d.r.*).

**$^1$H NMR** (400 MHz, DMSO-$d_6$): δ = 7.42 (d, *J* = 8.8 Hz, ≈0.4H), 7.24 (d, *J* = 7.5 Hz, ≈0.6H), 7.18 (d, *J* = 8.5 Hz, ≈0.4H), 7.16 – 7.01 (m, ≈3.6H), 4.35 (dd, *J* = 10.1, 8.4 Hz, ≈0.4H), 4.21 (t, *J* = 8.4 Hz, ≈0.6H), 3.65 (s, ≈1.5H), 3.45 (q, *J* = 7.2 Hz, ≈0.8H), 3.38 (s, ≈1.4H), 3.26 (dt, *J* = 10.0, 6.9 Hz, ≈0.4H), 2.31 (s, 1H), 2.26 (s, 2H), 1.34 (s, 4H), 1.28 (s, 4H), 1.18 (d, *J* = 6.6 Hz, 2H), 1.05 (d, *J* = 6.9 Hz, 1H) ppm. Sum of diastereomers.

**$^{13}$C NMR** (101 MHz, DMSO-$d_6$): δ = 172.8, 172.3, 155.6, 155.2, 141.2, 140.7, 135.9, 135.0, 130.3, 130.0, 126.8, 126.2, 126.0, 125.9, 125.9, 125.3, 78.3, 78.2, 58.4, 57.7, 51.6, 51.5, 36.2, 35.7, 28.2, 28.1, 19.1, 19.1, 18.9, 17.0 ppm. Sum of diastereomers.

**HRMS** (ESI+) m/z: calcd. for $C_{17}H_{25}NO_4Na$ $[M+Na]^+$ 330.1676, found 330.1677.

## Methyl 2-((*tert*-butoxycarbonyl)amino)-3-(3-methylphenyl)butanoate (22)

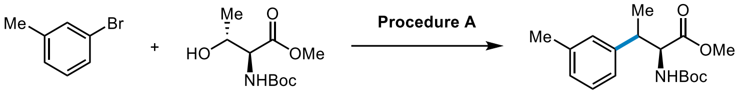


1-bromo-3-methylbenzene (85.8 mg, 0.5 mmol) was used with general procedure A. The crude reaction mixture was purified by reverse phase flash column chromatography (MeCN:$H_2O$ = 50:50 → 70:30) to yield the product as a mixture of two diastereomers as clear oil (110 mg, 0.36 mmol, 72% yield, 1.6:1 *d.r.*).

**$^1$H NMR** (400 MHz, DMSO-$d_6$): δ = 7.37 (d, *J* = 8.7 Hz, ≈0.3H), 7.16 (t, *J* = 7.3 Hz, 1H), 7.10 – 6.93 (m, ≈3.6H), 4.17 (td, *J* = 8.8, 1.8 Hz, 1H), 3.64 (s, 2H), 3.38 (s, 1H), 3.14 – 2.97 (m, 3H), 2.27 (s, 3H), 1.36 (s, 3H), 1.27 (s, 6H), 1.22 (d, *J* = 7.1 Hz, 1H), 1.13 (d, *J* = 7.1 Hz, 2H) ppm. Sum of diastereomers.

**$^{13}$C NMR** (101 MHz, DMSO-$d_6$): δ = 172.5, 172.2, 155.6, 155.2, 142.8, 142.4, 137.1, 128.2, 128.1, 127.3, 127.2, 124.7, 124.6, 78.3, 78.3, 59.7, 59.2, 51.6, 51.3, 40.8, 40.5, 28.2, 28.0, 21.1, 21.1, 18.8, 17.0 ppm. Sum of diastereomers.

**HRMS** (ESI+) m/z: calcd. for $C_{17}H_{25}NO_4Na$ $[M+Na]^+$ 330.1676, found 330.1677.

## Methyl 2-(1,3-dioxoisoindolin-2-yl)-3-(4-(trifluoromethyl)phenyl)butanoate (23)

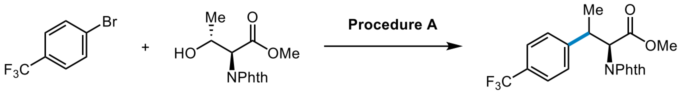


1-Bromo-4-(trifluoromethyl)benzene (114 mg, 0.5 mmol) and Phth-Thr-OMe (230 mg, 0.88 mmol, 1.75 equiv) was used with general procedure A. The crude reaction mixture was purified by reverse phase flash column chromatography (MeCN:$H_2O$ = 50:50 → 65:45) to yield the two diastereomers as clear oil (43.6 (18.3+25.3)mg, 0.11 mmol, 22% yield, 1.4:1 *d.r.*).

**Diastereomer 1:**

**$^1$H NMR** (400 MHz, $CDCl_3$): δ = 7.70 (dd, *J* = 5.5, 3.1 Hz, 2H), 7.64 (dd, *J* = 5.6, 3.0 Hz, 2H), 7.38 (d, *J* = 8.4 Hz, 2H), 7.27 (d, *J* = 7.4 Hz, 2H), 4.97 (d, *J* = 10.7 Hz, 1H), 4.07 (dq, *J* = 10.7, 6.8 Hz, 1H), 3.76 (s, 3H), 1.57 (d, *J* = 6.8 Hz, 3H) ppm.

**$^{13}$C NMR** (101 MHz, $CDCl_3$): δ = 169.0, 167.3, 146.6, 134.3, 131.3, 129.2 (q, *J* = 32.4 Hz), 128.0, 125.4 (q, *J* = 3.8 Hz), 123.6, 56.5, 53.0, 39.6, 21.1 ppm.[2]

**$^{19}$F NMR** (376 MHz, $CDCl_3$): δ = -62.6 ppm.

**HRMS** (ESI+) m/z: calcd. for $C_{20}H_{16}NO_4F_3Na$ $[M+Na]^+$ 414.0924, found 414.0922.

**Diastereomer 2:**

**$^1$H NMR** (400 MHz, $CDCl_3$): δ = 7.92 (dd, *J* = 5.4, 3.1 Hz, 2H), 7.79 (dd, *J* = 5.5, 3.0 Hz, 2H), 7.60 (d, *J* = 8.1 Hz, 2H), 7.50 (d, *J* = 8.1 Hz, 2H), 5.09 (d, *J* = 10.1 Hz, 1H), 3.99 (dq, *J* = 10.1, 7.1 Hz, 1H), 3.56 (s, 3H), 1.19 (d, *J* = 7.2 Hz, 3H) ppm.

**$^{13}$C NMR** (101 MHz, $CDCl_3$): δ = 168.7, 167.7, 148.1, 134.6, 131.7, 129.2 (q, *J* = 32.3 Hz), 128.2, 125.7 (q, *J* = 3.8 Hz), 124.0, 56.7, 52.7, 39.3, 19.3 ppm.[3]

**$^{19}$F NMR** (376 MHz, $CDCl_3$): δ = -62.4 ppm.

**HRMS** (ESI+) m/z: calcd. for $C_{20}H_{16}NO_4F_3Na$ $[M+Na]^+$ 414.0924, found 414.0922.

## Methyl 2-((4-methylphenyl)sulfonamido)-3-(4-(trifluoromethyl)phenyl)butanoate (24)

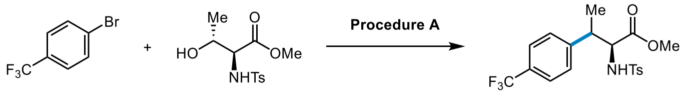


1-Bromo-4-(trifluoromethyl)benzene (114 mg, 0.5 mmol) and Tos-Thr-OMe (251 mg, 0.88 mmol, 1.75 equiv) was used with general procedure A. The crude reaction mixture was purified by reverse phase flash column chromatography (MeCN:$H_2O$ = 50:50 → 65:35) to yield the product as a mixture of two diastereomers as clear oil (43.0 mg, 0.10 mmol, 21% yield, 1.2:1 *d.r.*).

**$^1$H NMR** (500 MHz, DMSO-$d_6$): δ = 8.58 – 8.22 (m, 1H), 7.55 (d, *J* = 8.1 Hz, 1H), 7.52 (d, *J* = 8.3 Hz, 1H), 7.51 – 7.48 (m, 1H), 7.35 (dd, *J* = 8.3, 6.5 Hz, 3H), 7.29 – 7.23 (m, 1H), 7.22 – 7.17 (m, 1H), 3.92 (d, *J* = 8.1 Hz, ≈0.5H), 3.88 (d, *J* = 9.8 Hz, ≈0.5H), 3.43 (s, ≈1.5H), 3.19 – 3.11 (m, ≈1.9H), 3.06 (dq, *J* = 9.9, 7.0 Hz, ≈0.5H), 2.33 (s, ≈1.5H), 2.33 (s, ≈1.5H), 1.21 (d, *J* = 7.0 Hz, ≈1.5H), 1.10 (d, *J* = 7.0 Hz, ≈1.5H) ppm.

**$^{13}$C NMR** (126 MHz, DMSO-$d_6$): δ = 171.1, 170.6, 146.9 (q, *J* = 1.4 Hz), 146.2 (q, *J* = 1.4 Hz), 142.5, 142.4, 137.9, 137.5, 129.3, 129.2, 128.6, 128.6, 127.5 (q, *J* = 31.5 Hz), 127.3 (q, *J* = 31.6 Hz), 126.4, 126.3, 125.0 (q, *J* = 3.7 Hz), 124.9 (q, *J* = 4.2 Hz), 124.4 (d, *J* = 272.6 Hz), 124.3 (q, *J* = 271.9 Hz), 61.3, 61.0, 51.8, 51.5, 41.3, 41.2, 20.9, 20.9, 18.0, 16.4 ppm. Sum of diastereomers.

**$^{19}$F NMR** (470 MHz, DMSO-$d_6$): δ = -60.8, -60.9 ppm. Sum of diastereomers.

**HRMS** (ESI+) m/z: calcd. for $C_{19}H_{20}NO_4F_3SNa$ $[M+Na]^+$ 438.0958, found 438.0957.

[2] $CF_3$ carbon signals are not visible due to low intensity.
[3] $CF_3$ carbon signals are not visible due to low intensity.

## Dimethyl 3,3'-(1,4-phenylene)-bis(2-((*tert*-butoxycarbonyl)amino)butanoate) (25)

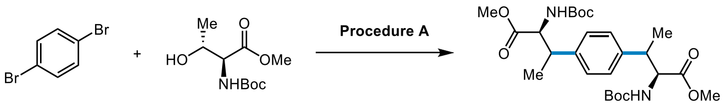

1,4-Dibromobenzene (61.8 mg, 0.25 mmol) was used with general procedure A. The crude reaction mixture was purified by reverse phase flash column chromatography (MeCN:$H_2O$ = 50:50 → 70:30) to yield the product as a mixture of diastereomers as clear oil (80.4 mg, 0.16 mmol, 32% yield).

**$^1$H NMR** (400 MHz, DMSO-$d_6$): δ = 7.35 (t, *J* = 8.4 Hz, ≈0.7H), 7.14 (dt, *J* = 22.4, 4.6 Hz, 4H), 6.95 (dd, *J* = 14.8, 8.4 Hz, 1H), 6.62 (t, *J* = 8.1 Hz, ≈0.2H), 4.20 – 3.93 (m, 2H), 3.64 (s, ≈3.6H), 3.37 – 3.28 (m, ≈2.3H), 3.13 – 2.88 (m, 2H), 1.34 (s, 6H), 1.28 – 1.17 (m, 14H), 1.11 (dd, *J* = 7.1, 2.4 Hz, 4H) ppm. Sum of diastereomers.

**$^{13}$C NMR** (101 MHz, DMSO-$d_6$): δ = 172.6, 172.5, 172.4, 172.2, 155.6, 155.2, 141.2, 141.0, 140.7, 140.5, 127.5, 127.4, 127.4, 78.4, 78.3, 59.9, 59.8, 59.4, 59.4, 51.6, 51.3, 51.2, 40.6, 40.5, 40.2, 28.2, 28.1, 18.7, 17.0, 17.0 ppm. Sum of diastereomers.

**HRMS** (ESI+) m/z: calcd. for $C_{26}H_{40}N_2O_8Na$ $[M+Na]^+$ 531.2677, found 531.2678.

## Methyl 2-((*tert*-butoxycarbonyl)amino)-3-(4-(trifluoromethyl)phenyl)butanoyl)-L-alaninate (26)

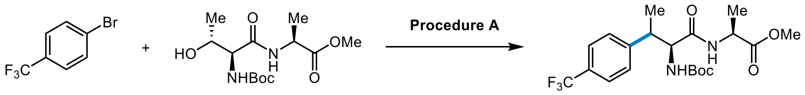

1-Bromo-4-(trifluoromethyl)benzene (114 mg, 0.5 mmol) and Boc-Thr-Ala-OMe (268 mg, 0.88 mmol, 1.7 equiv) was used with general procedure A. The crude reaction mixture was purified by reverse phase flash column chromatography (MeCN:$H_2O$ = 30:70 → 55:45) to yield the product as a mixture of two diastereomers as colorless powder (64.1 mg, 0.15 mmol, 30% yield, 2.1:1 *d.r.*).

**$^1$H NMR** (400 MHz, DMSO-$d_6$): δ = 8.56 (d, *J* = 6.7 Hz, ≈0.7H), 8.34 (d, *J* = 6.9 Hz, ≈0.3H), 7.61 (d, *J* = 8.0 Hz, 2H), 7.51 – 7.43 (m, 2H), 6.79 (d, *J* = 9.6 Hz, ≈0.3H), 6.66 (d, *J* = 9.5 Hz, ≈0.7H), 4.31 (q, *J* = 6.9 Hz, 1H), 4.24 (t, *J* = 9.5 Hz, 1H), 3.64 (s, 2H), 3.56 (s, 1H), 3.43 – 3.35 (m, ≈0.4H), 3.06 (p, *J* = 7.1 Hz, ≈0.7H), 1.31 (d, *J* = 7.3 Hz, 2H), 1.27 (s, 3H), 1.23 (d, *J* = 7.1 Hz, 3H), 1.17 (s, 7H) ppm. Sum of diastereomers.

**$^{13}$C NMR** (101 MHz, DMSO-$d_6$): δ = 172.8, 172.7, 170.8, 170.1, 155.1, 154.7, 148.5, 147.8, 128.7, 128.6, 127.0 (q, *J* = 31.4 Hz), 126.9 (q, *J* = 31.2 Hz), 124.6, 124.6, 124.5 (q, *J* = 271.7 Hz), 124.4 (q, *J* = 271.8 Hz), 78.1, 77.8, 58.8, 58.4, 51.7, 51.7, 47.6, 47.5, 41.7, 41.0, 28.0, 27.9, 18.1, 16.9, 16.8, 14.6 ppm. Sum of diastereomers.

**$^{19}$F NMR** (376 MHz, DMSO-$d_6$): δ = -60.85 – -60.97 (m) ppm. Sum of diastereomers.

**HRMS** (ESI+) m/z: calcd. for $C_{20}H_{28}N_2O_5F_3$ $[M+H]^+$ 433.1945, found 433.1944.

## Methyl 2-((*tert*-butoxycarbonyl)amino)-3-(pyridin-3-yl)butanoate (27)

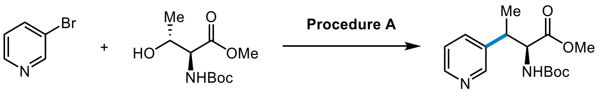

3-Bromopyridine (78.6 mg, 0.5 mmol) was used with general procedure A. The crude reaction mixture was purified by reverse phase flash column chromatography (MeCN:$H_2O$ = 40:60 → 55:45) to yield the product as a mixture of two diastereomers as colorless powder (109 mg, 0.37 mmol, 74% yield, 1.3:1 *d.r.*).

**$^1$H NMR** (400 MHz, DMSO-$d_6$): δ = 8.53 – 8.37 (m, 2H), 7.73 – 7.63 (m, 1H), 7.46 (d, *J* = 8.8 Hz, ≈0.4H), 7.30 (dt, *J* = 7.9, 3.9 Hz, 1H), 7.22 (d, *J* = 8.8 Hz, ≈0.6H), 4.22 (dt, *J* = 15.9, 8.8 Hz, 1H), 3.66 (s, 2H), 3.43 (s, 1H), 3.21 (p, *J* = 7.1 Hz, ≈0.4H), 3.16 – 3.04 (m, ≈0.6H), 1.34 (s, 3H), 1.24 (s, 7H), 1.19 (d, *J* = 7.1 Hz, 2H) ppm. Sum of diastereomers.

**$^{13}$C NMR** (101 MHz, DMSO-$d_6$): δ = 172.1, 171.8, 155.5, 155.1, 149.6, 149.1, 147.9, 147.7, 138.1, 137.7, 135.1, 134.8, 123.2, 78.4, 78.3, 59.1, 58.8, 51.7, 51.5, 38.4, 28.1, 28.0, 18.2, 16.3 ppm. Sum of diastereomers.

**GC-HRMS** ($R_f$ = 13.23 min) (CI) m/z: calcd. for $C_{15}H_{23}N_2O_4$ $[M+H]^+$ 295.1652, found 295.1653.

**GC-HRMS** ($R_f$ = 13.31 min) (CI) m/z: calcd. for $C_{15}H_{23}N_2O_4$ $[M+H]^+$ 295.1652, found 295.1653.

## Methyl 2-((*tert*-butoxycarbonyl)amino)-3-(2-methylpyridin-3-yl)butanoate (28)

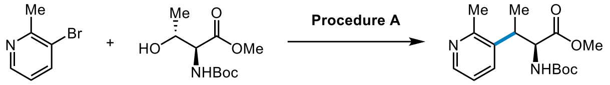


3-Bromo-2-methylpyridine (85.9 mg, 0.5 mmol) was used with general procedure A. The crude reaction mixture was purified by reverse phase flash column chromatography (MeCN:$H_2O$ = 30:70 → 40:60) to yield the product as a mixture of two diastereomers as clear oil (57.6 mg, 0.19 mmol, 38% yield, 1.8:1 *d.r.*).

**$^1$H NMR** (400 MHz, DMSO-$d_6$): δ = 8.26 (dd, *J* = 4.8, 1.6 Hz, 1H), 7.61 (d, *J* = 7.9 Hz, ≈0.7H), 7.52 (d, *J* = 7.8 Hz, ≈0.3H), 7.43 (d, *J* = 8.9 Hz, ≈0.6H), 7.27 (d, *J* = 8.6 Hz, ≈0.3H), 7.20 – 7.10 (m, 1H), 4.37 (t, *J* = 9.3 Hz, ≈0.3H), 4.27 (t, *J* = 8.3 Hz, ≈0.7H), 3.66 (s, 1H), 3.53 – 3.46 (m, 1H), 3.44 (s, 2H), 3.29 – 3.22 (m, ≈0.3H), 2.52 (s, 1H), 2.48 (s, 2H), 1.32 (s, 5H), 1.27 (s, 3H), 1.19 (d, *J* = 7.0 Hz, 2H), 1.10 (d, *J* = 7.0 Hz, 1H) ppm. Sum of diastereomers.

**$^{13}$C NMR** (101 MHz, DMSO-$d_6$): δ = 172.3, 171.9, 156.2, 155.5, 155.4, 155.1, 146.6, 146.4, 136.3, 135.7, 134.5, 133.4, 121.2, 121.2, 78.3, 78.3, 57.8, 57.4, 51.7, 51.6, 36.2, 35.7, 28.1, 28.0, 22.0, 21.9, 18.3, 16.2 ppm. Sum of diastereomers.

**GC-HRMS** ($R_f$ = 13.43 min) (CI) m/z: calcd. for $C_{16}H_{25}N_2O_4$ $[M+H]^+$ 309.1809, found 309.1808.

**GC-HRMS** ($R_f$ = 13.55 min) (CI) m/z: calcd. for $C_{16}H_{25}N_2O_4$ $[M+H]^+$ 309.1809, found 309.1808.

## Methyl 2-((*tert*-butoxycarbonyl)amino)-3-(pyridin-2-yl)butanoate (29)

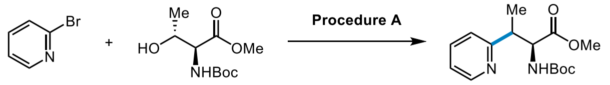


2-Bromopyridine (81.2 mg, 0.5 mmol) was used with general procedure A. The crude reaction mixture was purified by reverse phase flash column chromatography (MeCN:$H_2O$ = 30:70 → 50:50) to yield the two diastereomers as clear oil (41.8 (26.7+15.1) mg, 0.14 mmol, 28% yield, 1.8:1 *d.r.*).

**Diastereomer 1:**

**$^1$H NMR** (400 MHz, $CDCl_3$): δ = 8.54 (d, *J* = 4.1 Hz, 1H), 7.64 (td, *J* = 7.7, 1.9 Hz, 1H), 7.24 – 7.13 (m, 2H), 5.51 (d, *J* = 8.6 Hz, 1H), 4.60 (dd, *J* = 8.6, 4.9 Hz, 1H), 3.65 (s, 3H), 3.54 – 3.36 (m, 1H), 1.43 – 1.26 (m, 12H) ppm.

**$^{13}$C NMR** (101 MHz, $CDCl_3$): δ = 172.1, 161.1, 155.4, 148.7, 137.0, 122.4, 122.2, 79.9, 58.1, 52.3, 43.7, 28.4, 15.3 ppm.

**HRMS** (ESI+) m/z: calcd. for $C_{15}H_{23}N_2O_4$ $[M+H]^+$ 295.1652, found 295.1650.

**Diastereomer 2:**

**$^1$H NMR** (400 MHz, $CDCl_3$)δ 8.48 (dd, *J* = 4.9, 0.9 Hz, 1H), 7.59 (td, *J* = 7.8, 2.0 Hz, 1H), 7.16 – 7.07 (m, 2H), 6.37 (d, *J* = 9.4 Hz, 1H), 4.56 (dd, *J* = 9.4, 4.6 Hz, 1H), 3.65 – 3.55 (m, 4H), 1.43 (s, 9H), 1.38 (d, *J* = 7.3 Hz, 3H) ppm.

**$^{13}$C NMR** (101 MHz, $CDCl_3$): δ = 172.5, 162.0, 156.4, 149.0, 136.9, 122.9, 122.0, 79.7, 58.0, 52.2, 42.0, 28.5, 18.2 ppm.

**HRMS** (ESI+) m/z: calcd. for $C_{15}H_{23}N_2O_4$ $[M+H]^+$ 295.1652, found 295.1651.

## Methyl 2-((*tert*-butoxycarbonyl)amino)-3-(5-methylpyridin-2-yl)butanoate (30)

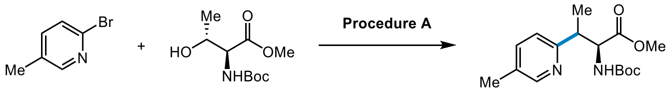


2-Bromo-5-methylpyridine (87.0, 0.5 mmol) was used with general procedure A. The crude reaction mixture was purified by reverse phase flash column chromatography (MeCN:$H_2O$ = 40:60 → 60:40) to yield the two diastereomers as colorless powder (40.7 (16.4+24.3) mg, 0.13 mmol, 26% yield, 1.5:1 *d.r.*).

**Diastereomer 1:**

**$^1$H NMR** (400 MHz, DMSO-$d_6$): δ = 8.31 (d, *J* = 2.5 Hz, 1H), 7.51 (dd, *J* = 8.0, 2.3 Hz, 1H), 7.25 (d, *J* = 8.8 Hz, 1H), 7.12 (d, *J* = 7.9 Hz, 1H), 4.39 (t, *J* = 8.5 Hz, 1H), 3.42 (s, 3H), 3.25 (p, *J* = 7.1 Hz, 1H), 2.25 (s, 3H), 1.35 (s, 9H), 1.20 (d, *J* = 7.1 Hz, 3H) ppm.

**$^{13}$C NMR** (101 MHz, DMSO-$d_6$): δ = 172.0, 158.7, 155.5, 148.9, 136.8, 130.7, 121.6, 78.3, 58.1, 51.4, 42.0, 28.1, 17.5, 16.1 ppm.

**HRMS** (ESI+) m/z: calcd. for $C_{16}H_{25}N_2O_4$ $[M+H]^+$ 309.1809, found 309.1806.

**Diastereomer 2:**

**$^1$H NMR** (400 MHz, DMSO-$d_6$): δ = 8.34 (d, *J* = 2.3 Hz, 1H), 7.52 (dd, *J* = 8.0, 2.3 Hz, 1H), 7.14 (d, *J* = 7.8 Hz, 1H), 6.95 (d, *J* = 8.5 Hz, 1H), 4.34 (t, *J* = 7.9 Hz, 1H), 3.57 (s, 3H), 3.31 – 3.24 (m, 1H), 2.25 (s, 3H), 1.32 (s, 9H), 1.18 (d, *J* = 7.1 Hz, 3H) ppm.

**$^{13}$C NMR** (101 MHz, DMSO-$d_6$): δ = 172.3, 158.6, 155.3, 149.1, 137.0, 131.0, 122.6, 78.4, 58.1, 51.5, 41.1, 28.0, 17.8, 17.5 ppm.

**HRMS** (ESI+) m/z: calcd. for $C_{16}H_{25}N_2O_4$ $[M+H]^+$ 309.1809, found 309.1806.

## Methyl 2-((*tert*-butoxycarbonyl)amino)-3-(5-(trifluoromethyl)pyridin-2-yl)butanoate (31)

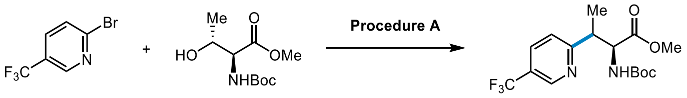

2-Bromo-5-(trifluoromethyl)pyridine (112 mg, 0.5 mmol) was used with general procedure A. The crude reaction mixture was purified by silica flash chromatography (EtOAc:cyclohexane = 0:100 → 15:85) to yield the product as a mixture of two diastereomers as clear oil (136 mg, 0.38 mmol, 75% yield, 1.9:1 *d.r.*). The diastereomers were partially separated by reverse phase flash column chromatography (MeCN:$H_2O$ = 60:40 → 70:30). Analysis was performed on minor pure fractions.

The reaction was upscaled to three times the scale using 2-bromo-5-(trifluoromethyl)pyridine (343 mg, 1.5 mmol) with general procedure A. The crude reaction mixture was purified by silica flash chromatography (EtOAc:cyclohexane = 0:100 → 15:85) to yield the product as a mixture of two diastereomers as clear oil (281 mg, 0.77 mmol, 52% yield, 1.9:1 *d.r.*).

**Diastereomer 1:**

**$^1$H NMR** (400 MHz, Chloroform-*d*): δ = 8.81 – 8.74 (m, 1H), 7.84 (dd, *J* = 8.2, 2.4 Hz, 1H), 7.32 (d, *J* = 8.2 Hz, 1H), 5.39 (d, *J* = 8.7 Hz, 1H), 4.66 (dd, *J* = 8.7, 4.9 Hz, 1H), 3.68 (s, 3H), 3.59 – 3.50 (m, 1H), 1.39 (d, *J* = 7.4 Hz, 3H), 1.36 (s, 9H) ppm.

**$^{13}$C NMR** (101 MHz, Chloroform-*d*): δ = 171.8, 165.5, 155.3, 146.6 (m), 134.0 – 133.2 (m), 125.3 – 124.8 (m), 123.7 (q, *J* = 273.0 Hz), 122.2, 80.1, 57.8, 52.4, 44.1, 28.3, 15.1 ppm.

**$^{19}$F NMR** (376 MHz, Chloroform-*d*): δ = -62.3 ppm.

**HRMS** (ESI+) m/z: calcd. for $C_{16}H_{21}N_2O_4F_3Na$ $[M+Na]^+$ 385.1346, found 385.1347.

**Diastereomer 2:**

**$^1$H NMR** (400 MHz, Chloroform-*d*): δ = 8.76 (d, *J* = 2.4 Hz, 1H), 7.83 (dd, *J* = 8.2, 2.4 Hz, 1H), 7.27 (d, *J* = 8.2 Hz, 1H), 6.11 (d, *J* = 9.5 Hz, 1H), 4.63 (dd, *J* = 9.5, 4.7 Hz, 1H), 3.69 (qd, *J* = 7.2, 4.7 Hz, 1H), 3.59 (s, 3H), 1.43 (s, 9H), 1.39 (d, *J* = 7.2 Hz, 3H) ppm.

**$^{13}$C NMR** (101 MHz, Chloroform-*d*): δ = 172.2, 166.3, 156.3, 146.1 (q, *J* = 4.1 Hz), 133.9 (q, *J* = 3.4 Hz), 125.0 (q, *J* = 33.1 Hz), 123.6 (q, *J* = 272.1 Hz), 122.7, 79.9, 57.6, 52.3, 42.4, 28.4, 17.9 ppm.

**$^{19}$F NMR** (376 MHz, Chloroform-*d*): δ = -62.4 ppm.

**HRMS** (ESI+) m/z: calcd. for $C_{16}H_{21}N_2O_4F_3Na$ $[M+Na]^+$ 385.1346, found 385.1345.

## Methyl 2-((*tert*-butoxycarbonyl)amino)-3-(quinolin-3-yl)butanoate (32)

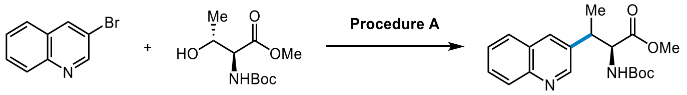

3-Bromoquinoline (106 mg, 0.5 mmol) was used with general procedure A. The crude reaction mixture was purified by reverse phase flash column chromatography (MeCN:$H_2O$ = 40:60 → 65:35) to yield the product as a mixture of two diastereomers as colorless powder (134 mg, 0.39 mmol, 78% yield, 1.4:1 *d.r.*).

**$^1$H NMR** (400 MHz, DMSO-$d_6$): δ = 8.83 (dd, *J* = 5.6, 2.2 Hz, 1H), 8.24 – 8.19 (m, 1H), 8.16 (d, *J* = 2.2 Hz, 1H), 7.98 (d, *J* = 8.5 Hz, 1H), 7.91 (t, *J* = 6.8 Hz, 1H), 7.70 (td, *J* = 6.8, 1.9 Hz, 1H), 7.57 (t, *J* = 7.5 Hz, 1H), 7.51 (d, *J* = 8.8 Hz, ≈0.4H), 7.30 (d, *J* = 8.7 Hz, ≈0.7H), 4.42 (t, *J* = 8.2 Hz, ≈0.4H), 4.36 (t, *J* = 9.1 Hz, ≈0.6H), 3.68 (s, 2H), 3.51 – 3.46 (m, ≈0.6H), 3.44 (s, 1H), 3.34 – 3.23 (m, ≈0.4H), 1.36 (d, *J* = 7.0 Hz, 1H), 1.31 (d, *J* = 7.1 Hz, 2H), 1.28 (s, 3H), 1.16 (s, 6H) ppm. Sum of diastereomers.

**$^{13}$C NMR** (101 MHz, DMSO-$d_6$): δ = 172.1, 171.7, 155.5, 155.2, 151.4, 151.1, 146.7, 146.7, 135.7, 135.4, 133.7, 133.7, 129.0, 128.9, 128.6, 128.5, 127.8, 127.8, 127.6, 127.5, 126.5, 126.4, 78.4, 78.3, 59.0, 59.0, 51.7, 51.6, 38.6, 38.5, 28.0, 27.9, 18.2, 16.2 ppm. Sum of diastereomers.

**HRMS** (ESI+) m/z: calcd. for $C_{19}H_{25}N_2O_4$ $[M+H]^+$ 345.1809, found 345.1807.

## Methyl 2-((*tert*-butoxycarbonyl)amino)-3-(quinolin-5-yl)butanoate (33)

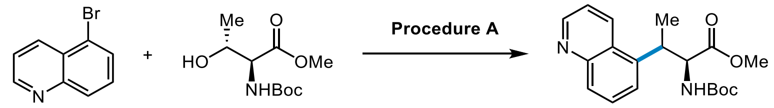


5-Bromoquinoline (106 mg, 0.5 mmol) was used with general procedure A. The crude reaction mixture was purified by reverse phase flash column chromatography (MeCN:$H_2O$ = 30:70 → 45:55) to yield the two diastereomers as colorless powder (97.2 (59.2+38.0) mg, 0.28 mmol, 56% yield, 1.6:1 *d.r.*).

**Diastereomer 1:**

**$^1$H NMR** (500 MHz, DMSO-$d_6$): δ = 8.90 (dd, J = 4.1, 1.6 Hz, 1H), 8.48 (d, J = 8.3 Hz, 1H), 7.93 (d, J = 8.6 Hz, ≈0.2H), 7.90 (d, J = 8.4 Hz, ≈0.8H), 7.72 (dd, J = 8.4, 7.3 Hz, 1H), 7.60 (dd, J = 8.6, 4.1 Hz, 1H), 7.57 – 7.54 (m, ≈0.8H), 7.50 (d, J = 7.3 Hz, ≈0.2H), 7.47 (d, J = 9.0 Hz, ≈0.8H), 7.05 (d, J = 9.1 Hz, ≈0.2H), 4.44 – 4.35 (m, 1H), 4.13 (p, J = 7.0 Hz, 1H), 3.34 (s, 3H), 1.36 (d, J = 7.0 Hz, 3H), 1.29 (s, 7H), 0.82 (s, 2H) ppm. Sum of rotamers.

**$^{13}$C NMR** (126 MHz, DMSO-$d_6$): δ = 171.9, 171.8, 155.5, 153.8, 150.0, 148.0, 139.2, 138.9, 131.0, 130.8, 128.9, 128.1, 128.0, 126.1, 125.9, 125.0, 125.0, 121.4, 121.3, 78.4, 78.1, 59.2, 58.7, 52.0, 51.7, 35.1, 34.5, 28.1, 27.3, 16.4, 14.9 ppm. Sum of rotamers.

**HRMS** (EI) m/z: calcd. for $C_{19}H_{24}N_2O_4$ $[M]^+$ 344.1731, found 344.1735.

**Diastereomer 2:**

**$^1$H NMR** (400 MHz, DMSO-$d_6$): δ = 8.90 (dd, *J* = 4.1, 1.6 Hz, 1H), 8.58 (d, *J* = 8.6 Hz, 1H), 7.90 (d, *J* = 8.3 Hz, 1H), 7.71 (t, *J* = 7.9 Hz, 1H), 7.57 (dd, *J* = 8.7, 4.1 Hz, 1H), 7.50 (d, *J* = 7.3 Hz, 1H), 7.23 (d, *J* = 8.4 Hz, 1H), 4.52 (t, *J* = 8.7 Hz, 1H), 3.98 (p, *J* = 7.1 Hz, 1H), 3.63 (s, 3H), 1.27 (d, *J* = 7.0 Hz, 3H), 1.24 (s, 9H) ppm.

**$^{13}$C NMR** (101 MHz, DMSO-$d_6$): δ = 172.0, 155.1, 149.8, 148.1, 139.7, 131.8, 128.9, 127.8, 126.3, 123.8, 121.0, 78.3, 58.0, 51.6, 35.1, 27.9, 18.7 ppm.

**HRMS** (EI) m/z: calcd. for $C_{19}H_{24}N_2O_4$ $[M]^+$ 344.1731, found 344.1733.

## Methyl 2-((*tert*-butoxycarbonyl)amino)-3-(thiophen-3-yl)butanoate (34)

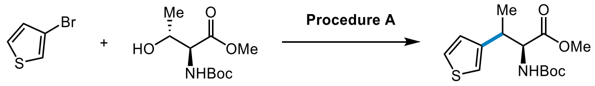

3-Bromothiophene (81.6 mg, 0.5 mmol) was used with general procedure A. The crude reaction mixture was purified by reverse phase flash column chromatography (MeCN:$H_2O$ = 40:60 → 75:25) to yield the product as a mixture of two diastereomers as clear oil (82.7 mg, 0.28 mmol, 55% yield, 1.3:1 *d.r.*).

**$^{1}$H NMR** (400 MHz, DMSO-$d_6$): δ = 7.42 (t, *J* = 3.3 Hz, 1H), 7.31 (d, *J* = 8.6 Hz, ≈0.5H), 7.25 (d, *J* = 1.8 Hz, ≈0.5H), 7.19 (d, *J* = 1.9 Hz, ≈0.5H), 7.03 (t, *J* = 3.5 Hz, 1H), 6.94 (d, *J* = 8.4 Hz, ≈0.6H), 4.20 (t, *J* = 8.0 Hz, ≈0.4H), 4.12 (t, *J* = 8.5 Hz, ≈0.6H), 3.63 (s, 2H), 3.46 (s, 1H), 3.30 – 3.17 (m, 1H), 1.35 (s, 3H), 1.30 (s, 6H), 1.21 (d, *J* = 7.1 Hz, 1H), 1.16 (d, *J* = 7.1 Hz, 2H) ppm. Sum of diastereomers.

**$^{13}$C NMR** (101 MHz, DMSO-$d_6$): δ = 172.2, 172.2, 155.6, 155.2, 143.4, 143.2, 127.3, 127.3, 125.6, 125.5, 121.4, 121.2, 78.4, 78.3, 59.2, 59.2, 51.6, 51.5, 36.2, 36.1, 28.2, 28.1, 18.2, 16.9 ppm. Sum of diastereomers.

**GC-HRMS** ($R_f$ = 12.80 min) (CI) m/z: calcd. for $C_{14}H_{22}N_1O_4S$ $[M+H]^+$ 300.1264, found 300.1263.

**GC-HRMS** ($R_f$ = 12.88 min) (CI) m/z: calcd. for $C_{14}H_{22}N_1O_4S$ $[M+H]^+$ 300.1264, found 300.1263.

## Methyl 2-((*tert*-butoxycarbonyl)amino)-3-(5-methylthiophen-2-yl)butanoate (35)

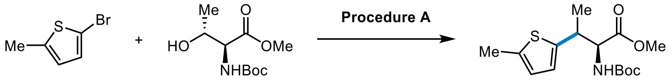


2-Bromo-5-methylthiophene (89.9 mg, 0.5 mmol) was used with general procedure A. The crude reaction mixture was purified by reverse phase flash column chromatography (MeCN:$H_2O$ = 50:50 → 65:35) to yield the product as a mixture of two diastereomers as clear oil (92.3 mg, 0.29 mmol, 59% yield, 1.4:1 *d.r.*). Analysis was performed on minor pure fractions.

**Diastereomer 1:**

**$^{1}$H NMR** (400 MHz, DMSO-$d_6$): δ = 7.30 (d, *J* = 8.9 Hz, 1H), 6.63 (d, *J* = 3.4 Hz, 1H), 6.61 – 6.56 (m, 1H), 4.18 (t, *J* = 8.1 Hz, 1H), 3.52 (s, 3H), 3.40 (p, *J* = 7.0 Hz, 1H), 2.37 (d, *J* = 1.2 Hz, 3H), 1.37 (s, 9H), 1.22 (d, *J* = 6.8 Hz, 3H) ppm.

**$^{13}$C NMR** (101 MHz, DMSO-$d_6$): δ = 171.6, 155.5, 143.2, 137.2, 124.7, 124.6, 124.2, 78.4, 59.7, 51.7, 51.7, 36.4, 28.1, 28.0, 18.0, 14.9 ppm.

**HRMS** (ESI+) m/z: calcd. for $C_{15}H_{23}N_1O_4S_1Na$ $[M+Na]^+$ 336.1240, found 336.1238.

**Diastereomer 2:**

**$^{1}$H NMR** (400 MHz, DMSO-$d_6$): δ = 6.80 (d, *J* = 8.6 Hz, 1H), 6.69 (d, *J* = 3.4 Hz, 1H), 6.60 (dd, *J* = 3.4, 1.3 Hz, 1H), 4.11 (t, *J* = 8.2 Hz, 1H), 3.64 (s, 3H), 3.42 – 3.32 (m, 1H), 2.37 (d, *J* = 1.1 Hz, 3H), 1.33 (s, 9H), 1.20 (d, *J* = 7.1 Hz, 3H) ppm.

**$^{13}$C NMR** (101 MHz, DMSO-$d_6$): δ = 171.7, 155.2, 143.0, 137.2, 124.7, 78.5, 59.6, 51.7, 36.4, 28.0, 19.1, 14.9 ppm.

**HRMS** (ESI+) m/z: calcd. for $C_{15}H_{23}N_1O_4S_1Na$ $[M+Na]^+$ 336.1240, found 336.1238.

## Methyl 3-(benzo[b]thiophen-3-yl)-2-((*tert*-butoxycarbonyl)amino)butanoate (36)

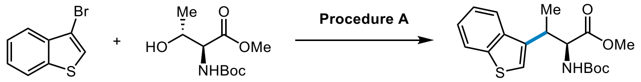

3-Bromo-1-benzothiophene (106 mg, 0.5 mmol) was used with general procedure A. The crude reaction mixture was purified by reverse phase flash column chromatography (MeCN:$H_2O$ = 50:50 → 75:25) to yield the product as a mixture of two diastereomers as clear oil (114 mg, 0.33 mmol, 65% yield, 1.1:1 *d.r.*).

**$^1$H NMR** (400 MHz, DMSO-$d_6$): δ = 7.97 (d, *J* = 7.9 Hz, 1H), 7.85 (d, *J* = 7.9 Hz, ≈0.6H), 7.77 (d, *J* = 7.9 Hz, ≈0.5H), 7.50 (d, *J* = 8.4 Hz, 1H), 7.46 – 7.33 (m, ≈2.4H), 7.20 (d, *J* = 8.3 Hz, ≈0.6H), 4.49 (t, *J* = 8.1 Hz, ≈0.5H), 4.38 (dd, *J* = 8.9, 6.6 Hz, ≈0.5H), 3.70 (p, *J* = 6.6 Hz, ≈0.5H), 3.65 – 3.52 (m, ≈2.5H), 3.42 (s, 1H), 1.37 – 1.23 (m, 12H) ppm. Sum of diastereomers.

**$^{13}$C NMR** (101 MHz, DMSO-$d_6$): δ = 172.0, 171.8, 155.6, 155.3, 139.7, 139.6, 138.2, 137.9, 137.3, 137.1, 124.3, 124.3, 124.1, 124.0, 123.2, 123.0, 122.9, 122.3, 121.8, 121.2, 78.4, 78.4, 57.9, 57.7, 51.7, 51.6, 34.7, 34.0, 28.1, 28.1, 17.4, 16.0 ppm. Sum of diastereomers.

**GC-HRMS** ($R_f$ = 15.63 min) (CI) m/z: calcd. for $C_{18}H_{24}N_1O_4S_1$ $[M+H]^+$ 350.1421, found 350.1418.

**GC-HRMS** ($R_f$ = 15.72 min) (CI) m/z: calcd. for $C_{18}H_{24}N_1O_4S_1$ $[M+H]^+$ 350.1421, found 350.1424.

## Tert-butyl 3-(3-((*tert*-butoxycarbonyl)amino)-4-methoxy-4-oxobutan-2-yl)-1*H*-indole-1-carboxylate (37)

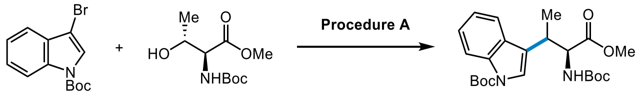


*tert*-Butyl 3-bromoindole-1-carboxylate (148 mg, 0.5 mmol) was used with general procedure A. The crude reaction mixture was purified by silica flash chromatography (EtOAc:cyclohexane = 0:100 → 15:85), followed by reverse phase flash column chromatography (MeCN:$H_2O$ = 50:50 → 95: 5) to yield the two diastereomers as clear oil (120 (58.4+61.7) mg, 0.28 mmol, 56% yield, 1.1:1 *d.r.*).

**Diastereomer 1:**

**$^1$H NMR** (400 MHz, DMSO-$d_6$): δ = 8.04 (d, *J* = 8.2 Hz, 1H), 7.62 (d, *J* = 7.7 Hz, 1H), 7.51 (s, 1H), 7.31 (td, *J* = 7.8, 1.4 Hz, 1H), 7.24 (td, *J* = 7.4, 1.3 Hz, 1H), 7.15 (d, *J* = 8.5 Hz, 1H), 4.41 (t, *J* = 8.3 Hz, 1H), 3.62 (s, 3H), 3.42 (p, *J* = 7.2 Hz, 1H), 1.62 (s, 9H), 1.34 – 1.19 (m, 12H) ppm.

**$^{13}$C NMR** (101 MHz, DMSO-$d_6$): δ = 171.9, 155.3, 149.0, 134.8, 129.4, 124.2, 122.7, 122.4, 121.6, 119.4, 114.7, 83.5, 78.4, 57.6, 51.6, 32.4, 28.0, 27.7, 17.5 ppm.

**HRMS** (ESI+) m/z: calcd. for $C_{23}H_{32}N_2O_6Na$ $[M+Na]^+$ 455.2153, found 455.2153.

**Diastereomer 2:**

**$^1$H NMR** (400 MHz, DMSO-$d_6$): δ = 8.03 (d, *J* = 8.1 Hz, 1H), 7.57 (d, *J* = 7.7 Hz, 1H), 7.48 (s, 1H), 7.32 (t, *J* = 7.8 Hz, 2H), 7.25 (t, *J* = 7.5 Hz, 1H), 4.39 (dd, *J* = 8.9, 6.1 Hz, 1H), 3.60 – 3.47 (m, 4H), 1.62 (s, 9H), 1.32 (s, 9H), 1.29 (d, *J* = 7.2 Hz, 3H) ppm.

**$^{13}$C NMR** (101 MHz, DMSO-$d_6$): δ = 171.9, 155.6, 149.0, 134.6, 129.3, 124.3, 122.8, 122.5, 121.9, 119.0, 114.8, 83.6, 78.3, 57.8, 51.7, 31.7, 28.0, 27.7, 15.5 ppm.

**HRMS** (ESI+) m/z: calcd. for $C_{23}H_{32}N_2O_6Na$ $[M+Na]^+$ 455.2153, found 455.2150.

## Methyl 2-((*tert*-butoxycarbonyl)amino)-3-(1*H*-indol-5-yl)butanoate (38)

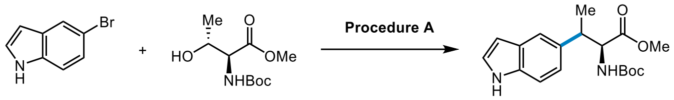


5-Bromoindole (98.9 mg, 0.5 mmol) was used with general procedure A. The crude reaction mixture was purified by reverse phase flash column chromatography (MeCN:$H_2O$ = 40:60 → 55:45) to yield one diastereomer pure as a clear oil and a mixture of two diastereomers as clear oil (59.4 mg, 0.18 mmol, 36% yield, 2.0:1 *d.r.*).

**$^1$H NMR** (500 MHz, DMSO-d$_6$): δ = 11.06 – 10.93 (m, 1H), 7.41 (s, ≈0.7H), 7.37 – 7.26 (m, ≈2.5H), 6.98 (dd, *J* = 8.4, 1.8 Hz, ≈0.7H), 6.94 (dt, *J* = 8.9, 1.9 Hz, ≈0.3H), 6.74 (d, *J* = 8.1 Hz, ≈0.6H), 6.39 – 6.34 (m, 1H), 4.22 – 4.13 (m, 1H), 3.65 (s, 2H), 3.33 (s, 1H), 3.21 – 3.02 (m, 1H), 1.36 (s, ≈2.5H), 1.28 (d, *J* = 7.2 Hz, 1H), 1.25 (s, ≈6.5H), 1.21 (d, *J* = 7.2 Hz, 1H) ppm. Sum of diastereomers.

**$^{13}$C NMR** (126 MHz, DMSO-d$_6$): δ = 172.9, 172.4, 155.7, 155.3, 134.9, 132.9, 132.6, 127.7, 125.5, 125.4, 120.9, 120.6, 118.8, 118.6, 111.2, 111.1, 101.0, 78.4, 78.3, 60.5, 60.0, 51.6, 51.3, 41.0, 40.6, 28.2, 28.1, 19.4, 17.8 ppm. Sum of diastereomers.

**$^1$H NMR** (400 MHz, DMSO-d$_6$): δ = 10.99 (s, 1H), 7.40 (d, *J* = 1.6 Hz, 1H), 7.33 – 7.26 (m, 2H), 6.97 (dd, *J* = 8.4, 1.7 Hz, 1H), 6.77 (d, *J* = 8.1 Hz, 1H), 6.38 – 6.32 (m, 1H), 4.16 (t, *J* = 8.6 Hz, 1H), 3.65 (s, 3H), 3.18 – 3.06 (m, 1H), 1.25 (s, 9H), 1.19 (d, *J* = 7.1 Hz, 3H) ppm.

**$^{13}$C NMR** (101 MHz, DMSO-d$_6$): δ = 172.9, 155.3, 134.9, 133.0, 127.6, 125.4, 120.6, 118.8, 111.2, 100.9, 78.3, 59.9, 51.5, 40.6, 28.0, 19.4 ppm.

**HRMS** (ESI+) m/z: calcd. for $C_{18}H_{24}N_2O_4Na$ $[M+Na]^+$ 355.1628, found 355.1632.

## Methyl 3-(1-acetyl-1*H*-indol-5-yl)-2-((*tert*-butoxycarbonyl)amino)butanoate (39)

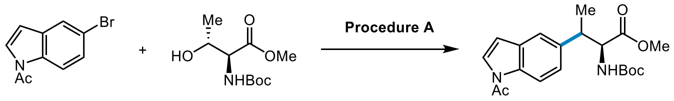


1-Acetyl-5-bromoindole (118 mg, 0.5 mmol) was used with general procedure A. The crude reaction mixture was purified by reverse phase flash column chromatography (MeCN:$H_2O$ = 50:50 → 55:45) to yield the product as a mixture of two diastereomers as clear oil (148 mg, 0.40 mmol, 79% yield, 1.6:1 *d.r.*).

**$^1$H NMR** (500 MHz, DMSO-d$_6$): δ = 8.23 (d, *J* = 8.2 Hz, 1H), 7.82 – 7.76 (m, 1H), 7.50 (s, ≈0.6H), 7.45 (s, ≈0.4H), 7.39 (d, *J* = 8.4 Hz, ≈0.3H), 7.23 (d, *J* = 8.7 Hz, ≈0.6H), 7.18 (d, *J* = 8.5 Hz, ≈0.4H), 7.00 – 6.93 (m, ≈0.5H), 6.70 – 6.66 (m, 1H), 4.28 – 4.19 (m, 1H), 3.66 (s, 2H), 3.36 (s, 1H), 3.28 – 3.15 (m, 1H), 2.62 (s, 3H), 1.35 (s, 3H), 1.30 (d, *J* = 6.8 Hz, 1H), 1.24 (s, 6H), 1.22 (d, *J* = 7.0 Hz, 2H) ppm. Sum of diastereomers.

**$^{13}$C NMR** (126 MHz, DMSO-d$_6$): δ = 172.6, 172.2, 169.3, 169.3, 155.6, 155.3, 138.0, 137.6, 133.9, 133.8, 130.4, 127.3, 124.4, 124.4, 119.7, 119.6, 115.6, 115.6, 108.3, 108.2, 78.4, 78.3, 60.1, 59.7, 51.6, 51.4, 40.9, 40.6, 28.1, 28.0, 23.7, 18.9, 17.4 ppm. Sum of diastereomers.

**HRMS** (ESI+) m/z: calcd. for $C_{20}H_{26}N_2O_5Na$ $[M+Na]^+$ 397.1734, found 397.1738.

## Methyl 2-((*tert*-butoxycarbonyl)amino)-3-(2,4-dioxo-1,2,3,4-tetrahydroquinazolin-6-yl)butanoate (40)

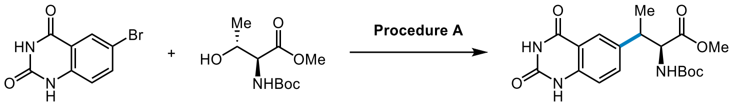

6-Bromo-1H-quinazoline-2,4-dione (120 mg, 0.5 mmol) was used with general procedure A. The crude reaction mixture was purified by reverse phase flash column chromatography (MeCN:$H_2O$ = 30:70 → 35:65), followed by silica flash chromatography (EtOAc:cyclohexane = 25:75 → 75:25) to yield the product as a mixture of two diastereomers as colorless solid (38.1 mg, 0.10 mmol, 20% yield, 3.0:1 *d.r.*).

**$^1$H NMR** (400 MHz, DMSO-$d_6$): δ = 11.24 – 11.18 (m, 1H), 11.09 – 11.03 (m, 1H), 7.80 – 7.75 (m, ≈0.8H), 7.74 – 7.67 (m, ≈0.3H), 7.59 – 7.49 (m, 1H), 7.37 (d, *J* = 8.7 Hz, ≈0.2H), 7.18 – 7.04 (m, ≈1.7H), 4.20 (t, *J* = 8.1 Hz, ≈0.2H), 4.14 (t, *J* = 9.0 Hz, ≈0.7H), 3.65 (s, 2H), 3.44 (s, 1H), 3.22 (p, *J* = 6.5 Hz, ≈0.3H), 3.12 (p, *J* = 7.9 Hz, ≈0.8H), 1.31 (s, 2H), 1.23 (s, 8H), 1.16 (d, *J* = 7.1 Hz, 3H) ppm. Sum of diastereomers.

**$^{13}$C NMR** (101 MHz, DMSO-$d_6$): δ = 172.1, 171.8, 162.8, 162.8, 155.5, 155.2, 150.2, 139.6, 139.5, 137.0, 136.6, 134.7, 134.3, 126.1, 125.5, 115.2, 114.0, 113.9, 78.4, 78.3, 59.4, 59.2, 51.6, 51.5, 40.0, 39.9, 28.1, 27.9, 18.2, 16.3 ppm. Sum of diastereomers.

**HRMS** (ESI+) m/z: calcd. for $C_{18}H_{23}N_3O_6Na$ $[M+Na]^+$ 400.1479, found 400.1480.

## Methyl 2-((*tert*-butoxycarbonyl)amino)-3-(1,3,7-trimethyl-2,6-dioxo-2,3,6,7-tetrahydro-1*H*-purin-8-yl)butanoate (41)

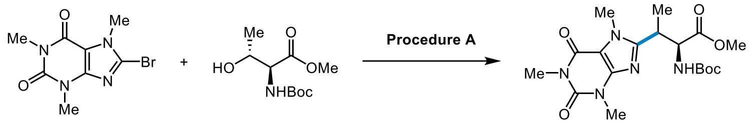

4-Bromocaffeine (138 mg, 0.5 mmol) was used with general procedure A. The crude reaction mixture was purified by reverse phase flash column chromatography (MeCN:$H_2O$ = 30:70 → 40:60) to yield the product as a single diastereomer as clear oil (59.2 mg, 0.14 mmol, 29% yield).

**Diastereomer 1:**

**$^1$H NMR** (400 MHz, DMSO-$d_6$): δ = 7.48 (d, *J* = 9.1 Hz, 1H), 4.50 (t, *J* = 8.8 Hz, 1H), 3.87 (s, 3H), 3.55 (s, 3H), 3.54 – 3.47 (m, 1H), 3.37 (s, 3H), 3.19 (s, 3H), 1.35 (s, 9H), 1.21 (d, *J* = 7.0 Hz, 3H) ppm.

**$^{13}$C NMR** (101 MHz, DMSO-$d_6$): δ = 171.1, 155.3, 155.1, 154.5, 150.9, 147.3, 106.2, 78.6, 56.1, 52.1, 32.1, 31.1, 29.3, 28.0, 27.4, 15.4 ppm.

**HRMS** (ESI+) m/z: calcd. for $C_{18}H_{27}N_5O_6Na$ $[M+Na]^+$ 432.1854, found 432.1855.

## 1-(*tert*-butyl) 2-methyl 3-(4-methylphenyl)pyrrolidine-1,2-dicarboxylate (42)

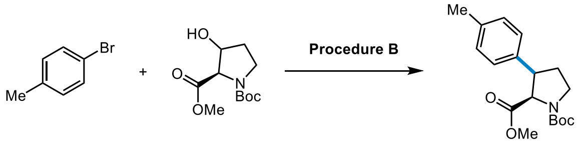

4-Bromotoluene (85.5 mg, 0.5 mmol) was used with general procedure B. The crude reaction mixture was purified by silica flash chromatography (EtOAc:cyclohexane = 0:100 → 20:80) to yield the product as a single diastereomer as clear oil (138 mg, 0.43 mmol, 87% yield).

**$^1$H NMR** (400 MHz, $CDCl_3$): δ = 7.18 – 7.08 (m, 4H), 4.35 (d, *J* = 6.1 Hz, ≈0.3H), 4.21 (d, *J* = 6.8 Hz, ≈0.7H), 3.79 – 3.72 (m, 1H), 3.71 – 3.67 (m, 3H), 3.66 – 3.53 (m, 1H), 3.46 – 3.35 (m, 1H), 2.33 (s, 3H), 2.32 – 2.21 (m, 1H), 2.08 – 1.94 (m, 1H), 1.48 (s, 3H), 1.41 (s, 6H) ppm. Summary of rotamers.

**$^{13}$C NMR** (101 MHz, $CDCl_3$): δ = 173.4, 173.3, 154.4, 153.8, 138.0, 137.7, 137.1, 136.9, 129.6, 129.6, 127.0, 126.9, 80.3, 80.2, 66.1, 65.4, 52.3, 52.1, 49.8, 48.6, 46.3, 46.2, 33.2, 32.5, 28.6, 28.4, 21.2 ppm. Summary of rotamers.

**HRMS** (ESI+) m/z: calcd. for $C_{18}H_{25}N_1O_4Na$ $[M+Na]^+$ 342.1676, found 342.1677.

## 1-(*tert*-butyl) 2-methyl 3-(2-methylphenyl)pyrrolidine-1,2-dicarboxylate (43)

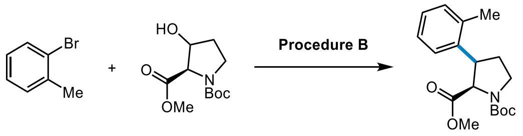


2-Bromotoluene (85.5 mg, 0.5 mmol) was used with general procedure B. The crude reaction mixture was purified by silica flash chromatography (EtOAc:cyclohexane = 0:100 → 20:80) to yield the product as a single diastereomer as clear oil (129 mg, 0.40 mmol, 81% yield).

**$^1$H NMR** (400 MHz, DMSO-d$_6$): δ = 7.33 (d, *J* = 7.7 Hz, ≈0.6H), 7.25 (d, *J* = 7.5 Hz, ≈0.4H), 7.23 – 7.10 (m, 3H), 4.14 (d, *J* = 5.9 Hz, ≈0.3H), 4.10 (d, *J* = 6.9 Hz, ≈0.7H), 3.68 – 3.62 (m, 1H), 3.62 (s, 2H), 3.61 (s, 1H), 3.60 – 3.53 (m, 1H), 3.52 – 3.41 (m, 1H), 2.26 (s, 1H), 2.24 (s, 2H), 2.22 – 2.15 (m, 1H), 2.05 – 1.91 (m, 1H), 1.42 (s, 3H), 1.33 (s, 6H) ppm. Summary of rotamers.

**$^{13}$C NMR** (101 MHz, DMSO-d$_6$): δ = 172.7, 172.3, 153.4, 152.7, 139.0, 138.5, 135.6, 130.3, 130.2, 126.7, 126.4, 125.6, 125.3, 79.2, 79.1, 65.5, 65.1, 51.9, 46.0, 44.8, 43.7, 31.6, 31.2, 28.0, 27.8, 19.0 ppm. Summary of rotamers.

**HRMS** (ESI+) m/z: calcd. for $C_{18}H_{25}N_1O_4Na$ $[M+Na]^+$ 342.1676, found 342.1678.

## 1-(*tert*-butyl) 2-methyl 3-(4-(trifluoromethyl)phenyl)pyrrolidine-1,2-dicarboxylate (44)

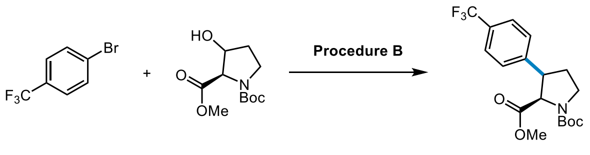


1-Bromo-4(trifluoromethyl)benzene (112 mg, 0.5 mmol) was used with general procedure B. The crude reaction mixture was purified by silica flash chromatography (EtOAc:cyclohexane = 0:100 → 20:80) to yield the product as a single diastereomer as clear oil (152 mg, 0.41 mmol, 81% yield).

**$^1$H NMR** (400 MHz, DMSO-*d*$_6$): δ = 7.69 (d, *J* = 8.5 Hz, 2H), 7.60 – 7.51 (m, 2H), 4.21 (d, *J* = 7.3 Hz, ≈0.3H), 4.17 (d, *J* = 7.9 Hz, ≈0.7H), 3.68 – 3.57 (m, 1H), 3.62 (s, 2H), 3.60 (s, 1H), 3.57 – 3.47 (m, 1H), 3.47 – 3.37 (m, 1H), 2.30 – 2.17 (m, 1H), 2.14 – 2.00 (m, 1H), 1.42 (s, 3H), 1.33 (s, 6H) ppm. Summary of rotamers.

$^{13}$**C NMR** (101 MHz, DMSO-*d*$_6$): δ = 172.3, 171.8, 153.3, 152.6, 145.4, 144.9, 128.2, 128.1, 127.8 (q, *J* = 31.8 Hz), 125.4, 125.4, 124.2 (q, J = 272 Hz), 79.3, 79.2, 65.3, 65.0, 51.8, 51.8, 49.1, 48.0, 46.1, 46.0, 32.5, 31.9, 28.0, 27.8 ppm. Summary of rotamers.

$^{19}$**F NMR** (376 MHz, DMSO-*d*$_6$): δ = -61.0.

**HRMS** (ESI+) m/z: calcd. for $C_{18}H_{22}F_3N_1O_4Na$ $[M+Na]^+$ 396.1393, found 396.1397.

## 1-(*tert*-butyl) 2-methyl 3-(5-(trifluoromethyl)pyridin-2-yl)pyrrolidine-1,2-dicarboxylate (45)

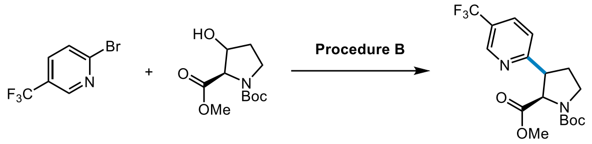


2-Bromo-5-(trifluoromethyl)-pyridine (113 mg, 0.5 mmol) was used with general procedure B. The crude reaction mixture was purified by silica flash chromatography (EtOAc:cyclohexane = 0:100 → 20:80) to yield the product as a single diastereomer as clear oil (93.8 mg, 0.25 mmol, 50% yield ).

$^{1}$**H NMR** (400 MHz, DMSO-d$_6$): δ = 8.97 – 8.91 (m, 1H), 8.18 (dd, *J* = 8.5, 2.5 Hz, 1H), 7.63 (d, *J* = 8.2 Hz, 1H), 4.51 (d, *J* = 6.0 Hz, ≈0.3H), 4.48 (d, *J* = 6.8 Hz, ≈0.7H), 3.81 – 3.68 (m, 1H), 3.63 (s, 2H), 3.62 (s, 1H), 3.61 – 3.54 (m, 1H), 3.50 – 3.38 (m, 1H), 2.34 – 2.21 (m, 1H), 2.20 – 2.05 (m, 1H), 1.40 (s, 3H), 1.33 (s, 6H) ppm. Summary of rotamers.

$^{13}$**C NMR** (101 MHz, DMSO-d$_6$): δ = 172.4, 172.0, 164.2, 163.8, 153.3, 152.6, 146.0 (q, J = 4.2 Hz), 134.4 (q, J = 3.5 Hz), 123.9 (q, J = 32.3 Hz), 123.7 (q, J = 272.2 Hz), 123.0, 122.9, 79.2, 79.1, 63.7, 63.4, 51.8, 51.8, 50.4, 49.4, 46.2, 46.0, 31.6, 31.1, 28.0, 27.8 ppm. Summary of rotamers.

$^{19}$**F NMR** (376 MHz, DMSO-d$_6$): δ = -60.9 ppm.

**HRMS** (ESI+) m/z: calcd. for C17H21F3N2O4Na [M+Na]+ 397.1346, found 397.1348.

## 1-(*tert*-butyl) 2-methyl -3-(4-chlorophenyl)pyrrolidine-1,2-dicarboxylate (46)

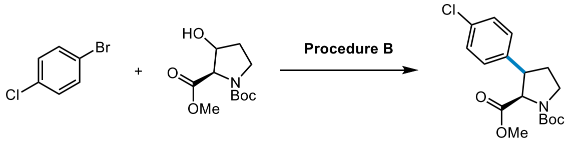


1-Bromo-4-chlorobenzene (95.7 mg, 0.5 mmol) was used with general procedure B. The crude reaction mixture was purified by reverse phase flash column chromatography (MeCN:$H_2O$ = 50:50 → 65:35) to yield the product as a single diastereomer as clear oil (148 mg, 0.44 mmol, 87% yield).

$^{1}$**H NMR** (400 MHz, $CDCl_3$): δ = 7.30 – 7.21 (m, 2H), 7.19 – 7.09 (m, 2H), 4.28 (d, *J* = 6.3 Hz, ≈0.4H), 4.16 (d, *J* = 6.8 Hz, ≈0.6H), 3.76 – 3.49 (m, 5H), 3.43 – 3.32 (m, 1H), 2.33 – 2.18 (m, 1H), 2.02 – 1.88 (m, 1H), 1.44 (s, ≈3H), 1.38 (s, ≈6H) ppm. Summary of rotamers.

$^{13}$**C NMR** (101 MHz, $CDCl_3$): δ = 172.9, 172.7, 154.2, 153.5, 139.3, 138.9, 133.0, 132.7, 128.9, 128.8, 128.4, 128.3, 80.3, 80.2, 65.7, 65.1, 52.2, 52.0, 49.3, 48.1, 46.1, 45.9, 32.9, 32.2, 28.4, 28.2 ppm. Summary of rotamers.

**HRMS** (ESI+) m/z: calcd. for $C_{17}H_{22}NO_4ClNa$ $[M+Na]^+$ 362.1130, found 362.1128.

## 11. Bibliography


(1) Stache, E. E.; Ertel, A. B.; Tomislav, R.; Doyle, A. G. Generation of Phosphoranyl Radicals via Photoredox Catalysis Enables Voltage-Independent Activation of Strong C-O Bonds. *ACS Catal.* **2018**, *8*, 11134–11139.

(2) Fang, J.; Jian, Z.; Liu, H.; Wang, Y.; Yu, X.; Mou, Z.; Wang, H. Nickel metallaphotoredox-catalyzed C–O bond activation/Csp2–Csp3 coupling enabled by phosphine. *Org. Chem. Front.* **2024**, *11*, 3058–3065.

(3) Guo, H.-M.; Wu, X. Selective deoxygenative alkylation of alcohols via photocatalytic domino radical fragmentations. *Nat. Commun.* **2021**, *12*, 5365.

(4) Zhang, X.; MacMillan, D. W. C. Alcohols as Latent Coupling Fragments for Metallaphotoredox Catalysis: sp3–sp2 Cross-Coupling of Oxalates with Aryl Halides. *J. Am. Chem. Soc.* **2016**, *138*, 13862–13865.

(5) Dong, Z.; MacMillan, D. W. C. Metallaphotoredox-enabled deoxygenative arylation of alcohols. *Nature* **2021**, *598*, 451–456.

(6) Zhang, L.-L.; Gao, Y.-Z.; Cai, S.-H.; Yu, H.; Shen, S.-J.; Ping, Q.; Yang, Z.-P. Ni-catalyzed enantioconvergent deoxygenative reductive cross-coupling of unactivated alkyl alcohols and aryl bromides. *Nat. Commun.* **2024**, *15*, 2733.

(7) Li, J.; Zhang, X.; Yao, Y.; Gao, Y.; Yang, W.; Zhao, W. Palladium-Catalyzed Suzuki-Miyaura Cross-Coupling of Oxygen-Substituted Allylboronates with Aryl/Vinyl (Pseudo)Halides. *J. Org. Chem.* **2022**, *87*, 6951–6959.

(8) Huang, Z. C.; Ruan, Z. L.; Xu, H.; Dai, H. X. Ring expansion of 3-hydroxyoxindoles to 4-quinolones via palladium-catalyzed C-C(acyl) bond cleavage. *Chem. Commun.* **2024**, *61*, 109–112.

(9) Kirchberg, S.; Frohlich, R.; Studer, A. Stereoselective palladium-catalyzed carboaminoxylations of indoles with arylboronic acids and TEMPO. *Angew. Chem. Int. Ed.* **2009**, *48*, 4235–4238.

(10) Valderrama-Callejon, R.; Vargas, E. L.; Alonso, I.; Tortosa, M.; Belen Cid, M. Diboron Reagents in N-N Bond Cleavage of Hydrazines, N-Nitrosamines, and Azides: Reactivity and Mechanistic Insights. *Chem. Eur. J.* **2025**, *31*, e202404081.

(11) Awolade, P.; Cele, N.; Kerru, N.; Singh, P. Synthesis, antimicrobial evaluation, and in silico studies of quinoline-1H-1,2,3-triazole molecular hybrids. *Mol. Divers.* **2021**, *25*, 2201–2218.

(12) Saint-Louis, C. J.; Warner, D. J.; Keane, K. S.; Kelley, M. D.; Meyers, C. M.; Blackstock, S. C. Photo-Electroswitchable Arylaminoazobenzenes. *J. Org. Chem.* **2021**, *86*, 11341–11353.

(13) Lowry, M. S.; Goldsmith, J. I.; Slinker, J. D.; Rohl, R.; Pascal, R. A.; Malliaras, G. G.; Bernhard, S. Single-Layer Electroluminescent Devices and Photoinduced Hydrogen Production from an Ionic Iridium(III) Complex. *Chemistry of Materials* **2005**, *17*, 5712–5719.

(14) Cusumano, A. Q.; Chaffin, B. C.; Doyle, A. G. Mechanism of Ni-Catalyzed Photochemical Halogen Atom-Mediated C(sp(3))-H Arylation. *J. Am. Chem. Soc.* **2024**, *146*, 15331–15344.

(15) Fenner, S.; Wilson, Z. E.; Ley, S. V. The Total Synthesis of the Bioactive Natural Product Plantazolicin A and Its Biosynthetic Precursor Plantazolicin B. *Chem. Eur. J.* **2016**, *22*, 15902–15912.

(16) Ferreira, P. M.; Maia, H. L.; Monteiro, L. S.; Sacramento, J. High yielding synthesis of dehydroamino acid and dehydropeptide derivatives. *J. Chem. Soc., Perkin Trans. 1* **1999**, 3697–3703.

(17) Liu, H.; Pattabiraman, V. R.; Vederas, J. C. Stereoselective Syntheses of 4-Oxa Diaminopimelic Acid and Its Protected Derivatives via Aziridine Ring Opening. *Org. Lett.* **2007**, *9*, 4211–4214.

(18) W. Knight, D.; William Sibley, A. Total synthesis of (-)-slaframine from (2R,3S)-3-hydroxyproline. *J. Chem. Soc., Perkin Trans. 1* **1997**, 2179-2188.

# 12. Crystallographic Data

The minor diastereomer of **31** was crystallized by slow evaporation of a mixture of MeCN/$H_2O$ = 2/1.

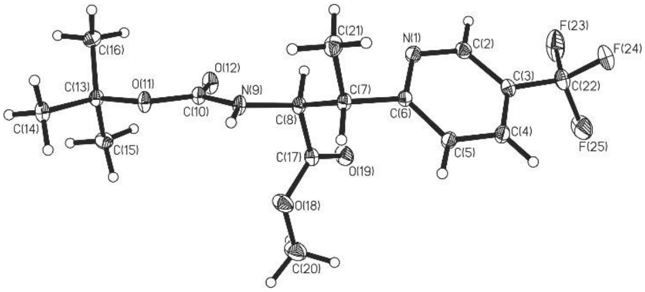

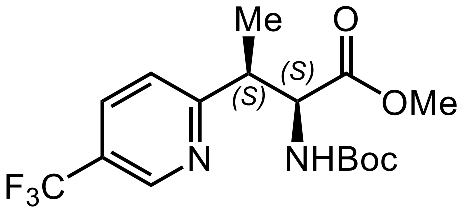


**Figure S1. Thermal ellipsoid plot of the minor diastereomer of 31, drawn at the 50% probability level (Mo-radiation).**

**Table S3. Crystal data and structure refinement for the minor diastereomer of 31 (Mo-radiation).**

| | | |
|---|---|---|
| CCDC number | 2530744 | |
| Empirical formula | $C_{16}H_{21}F_3N_2O_4$ | |
| Formula weight | 362.35 g $mol^{-1}$ | |
| Temperature | 100(2) K | |
| Wavelength | 0.71073 Å | |
| Crystal system | Monoclinic | |
| Space group | $P2_1$ ; No. 5 | |
| Unit cell dimensions | a = 5.1604(5) Å | $\alpha$= 90°. |
| | b = 18.2460(15) Å | $\beta$= 104.279(3)°. |
| | c = 9.4834(8) Å | $\gamma$ = 90°. |
| Volume | 865.34(13) $Å^3$ | |
| Z | 2 | |
| Density (calculated) | 1.391 g $cm^{-3}$ | |
| Absorption coefficient | 0.120 $mm^{-1}$ | |
| F(000) | 380 | |
| Crystal size | 0.446 x 0.292 x 0.052 $mm^3$ | |
| Theta range for data collection | 2.22 to 36.38°. | |
| Index ranges | -8<=h<=8, -30<=k<=30, -15<=l<=15 | |
| Reflections collected | 43733 | |
| Independent reflections | 8277 [R(int) = 0.0338] | |
| Completeness to theta = 25.24° | 99.8 % | |
| Absorption correction | Semi-empirical from equivalents | |
| Max. and min. transmission | 0.99 and 0.92 | |
| Refinement method | Full-matrix least-squares on $F^2$ | |
| Data / restraints / parameters | 8277 / 1 / 234 | |
| Goodness-of-fit on $F^2$ | 1.035 | |
| Final R indices [I>2sigma(I)] | $R_1$ = 0.0266, $wR_2$ = 0.0738 | |
| R indices (all data) | $R_1$ = 0.0276, $wR_2$ = 0.0745 | |
| Absolute structure parameter | -0.05(9) | |
| Largest diff. peak and hole | 0.301 and -0.201 e.$Å^{-3}$ | |

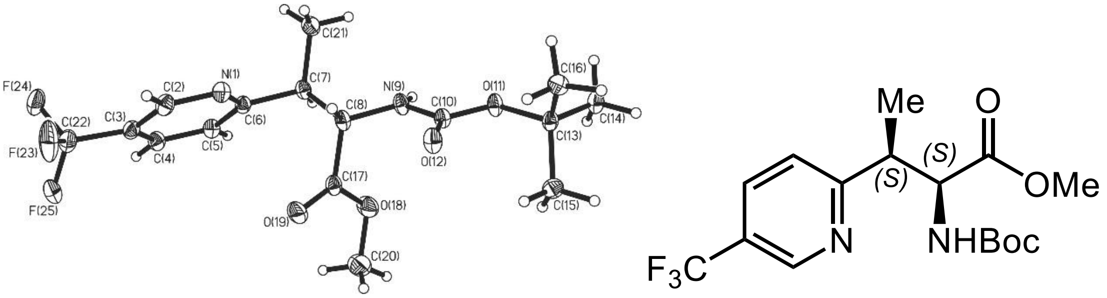


**Figure S2. Thermal ellipsoid plot of the minor diastereomer of 31, drawn at the 50% probability level (Cu-radiation).**

**Table S4. Crystal data and structure refinement for the minor diastereomer of 31 (Cu-radiation). Thermal ellipsoid drawn at the 50% probability level.**

| | | |
|---|---|---|
| CCDC number | 2530745 | |
| Empirical formula | $C_{16}H_{21}F_3N_2O_4$ | |
| Formula weight | 362.35 g mol$^{-1}$ | |
| Temperature | 100(2) K | |
| Wavelength | 1.54178 Å | |
| Crystal system | Monoclinic | |
| Space group | $P2_1$ ; No. 4 | |
| Unit cell dimensions | a = 5.1711(11) Å | $\alpha$= 90°. |
| | b = 18.266(4) Å | $\beta$= 104.218(5)°. |
| | c = 9.531(2) Å | $\gamma$ = 90°. |
| Volume | 872.7(3) Å$^3$ | |
| Z | 2 | |
| Density (calculated) | 1.379 g cm$^{-3}$ | |
| Absorption coefficient | 1.033 mm$^{-1}$ | |
| F(000) | 380 | |
| Crystal size | 0.388 x 0.121 x 0.087 mm$^3$ | |
| Theta range for data collection | 4.79 to 74.60°. | |
| Index ranges | -6<=h<=6, -22<=k<=22, -11<=l<=11 | |
| Reflections collected | 34975 | |
| Independent reflections | 3523 [R(int) = 0.0395] | |
| Completeness to theta = 67.68° | 99.8 % | |
| Absorption correction | Semi-empirical from equivalents | |
| Max. and min. transmission | 0.92 and 0.69 | |
| Refinement method | Full-matrix least-squares on F$^2$ | |
| Data / restraints / parameters | 3523 / 1 / 235 | |
| Goodness-of-fit on F$^2$ | 1.042 | |
| Final R indices [I>2sigma(I)] | $R_1$ = 0.0315, $wR_2$ = 0.0850 | |
| R indices (all data) | $R_1$ = 0.0315, $wR_2$ = 0.0850 | |
| Absolute structure parameter | 0.03(8) | |
| Extinction coefficient | 0.0059(14) | |
| Largest diff. peak and hole | 0.259 and -0.147 e.Å$^{-3}$ | |

# 13. NMR Spectra

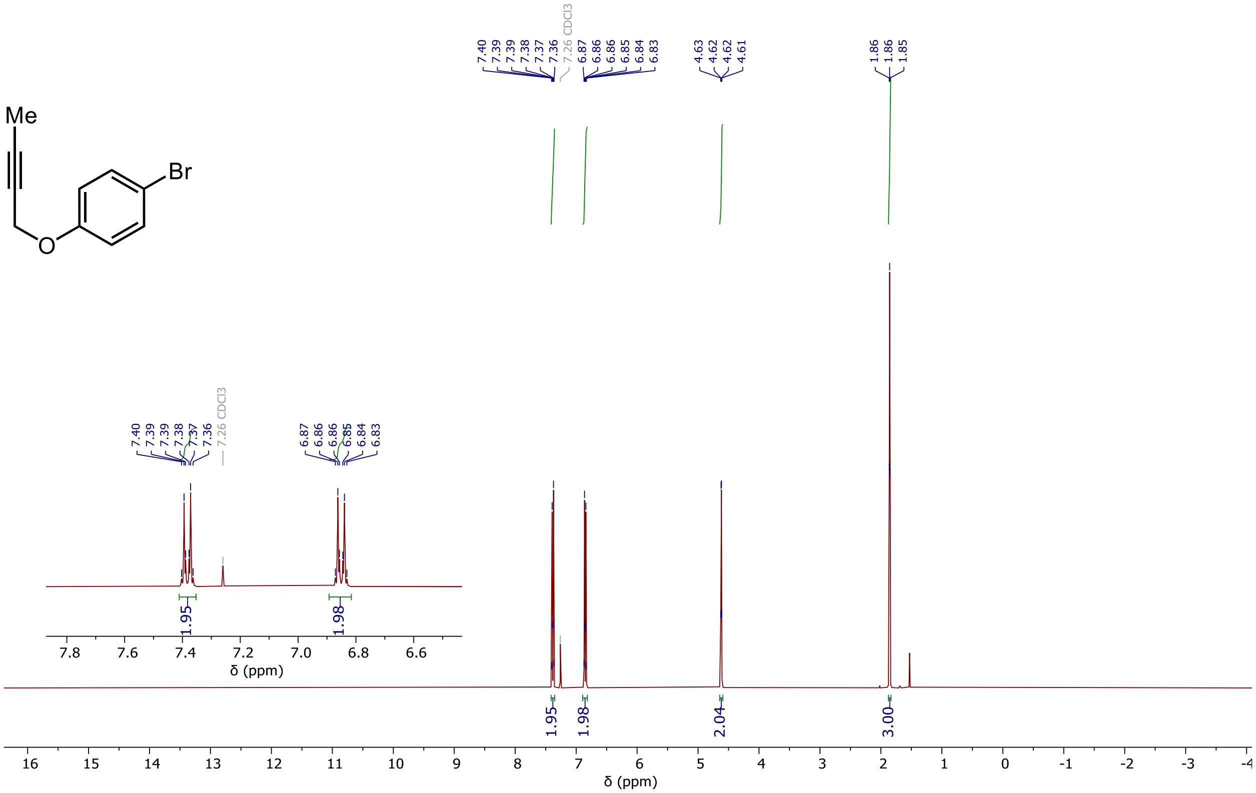

**Figure S3.** **$^1$H NMR spectra of 1-bromo-4-(but-2-yn-1-yloxy)benzene (400 MHz, $CDCl_3$).**

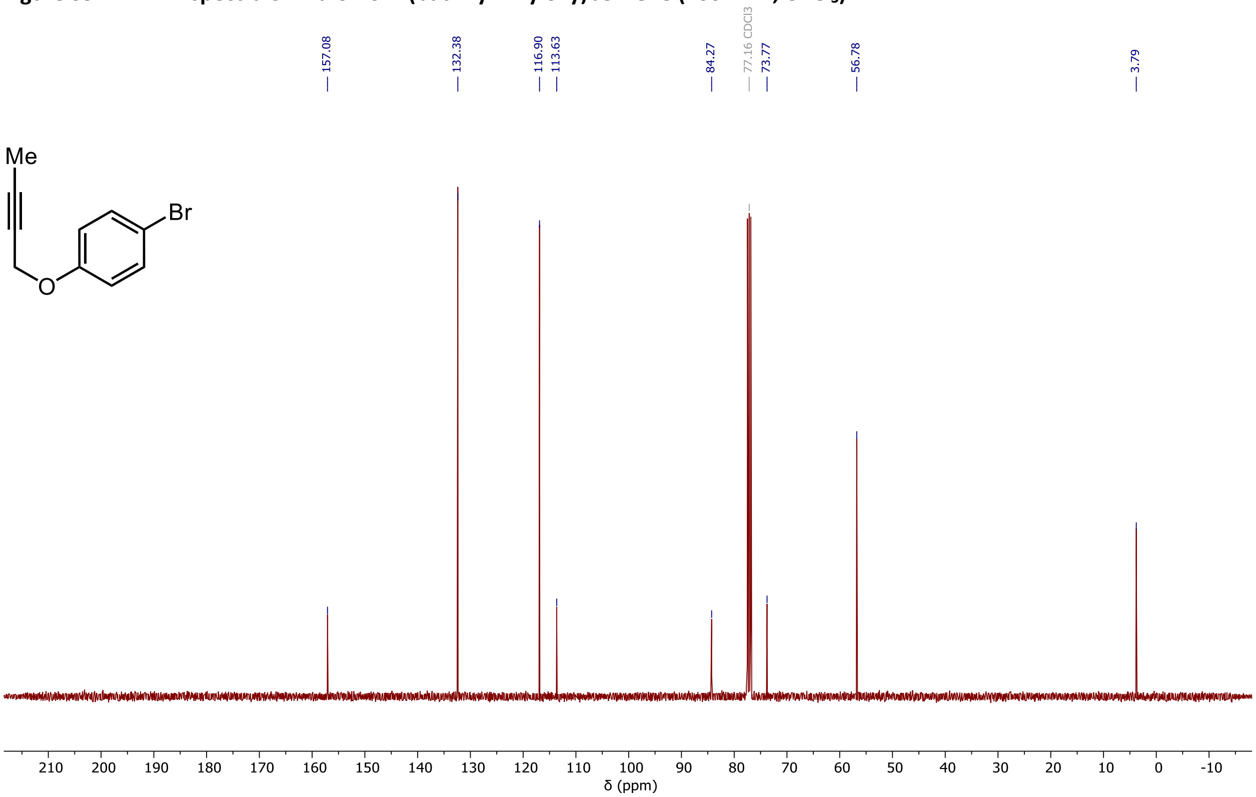

**Figure S4.** **$^{13}$C NMR spectra of 1-bromo-4-(but-2-yn-1-yloxy)benzene (101 MHz, $CDCl_3$).**

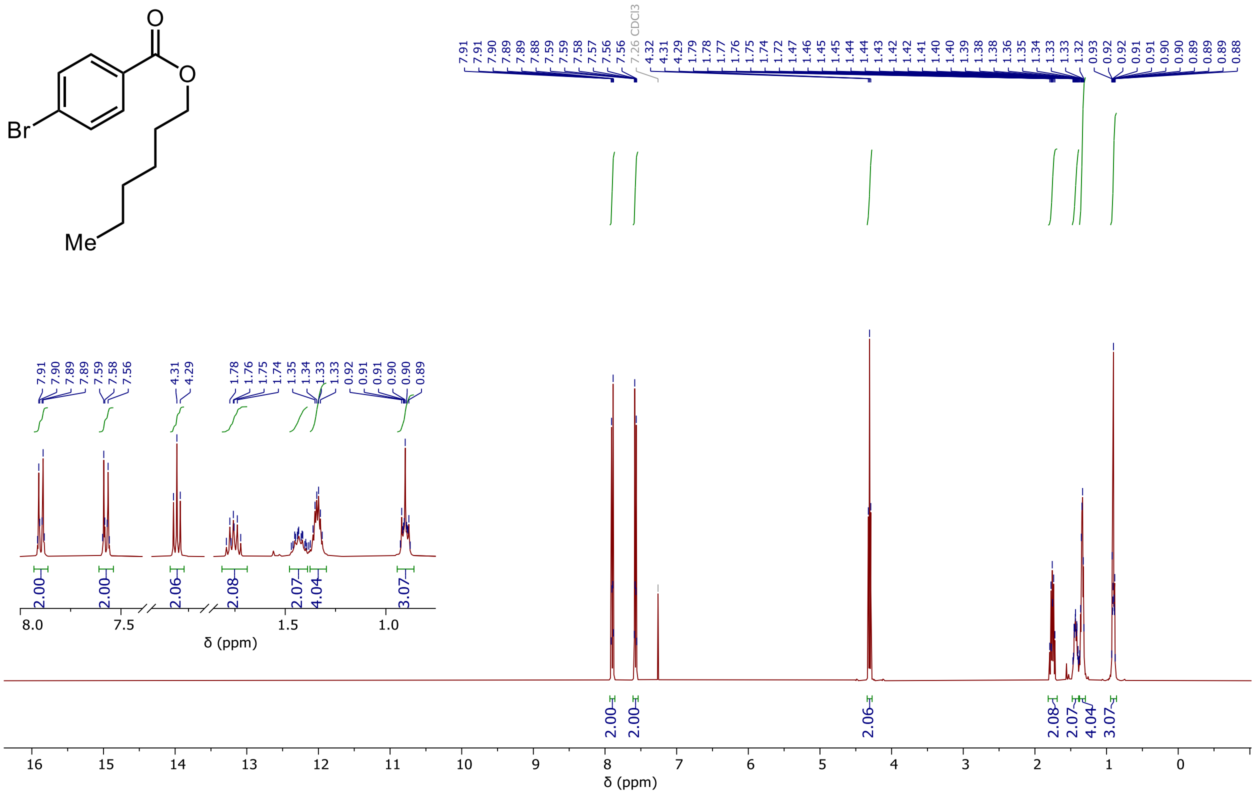


**Figure S5.** **$^{1}H$ NMR spectra of hexyl 4-bromobenzoate (400 MHz, $CDCl_3$).**

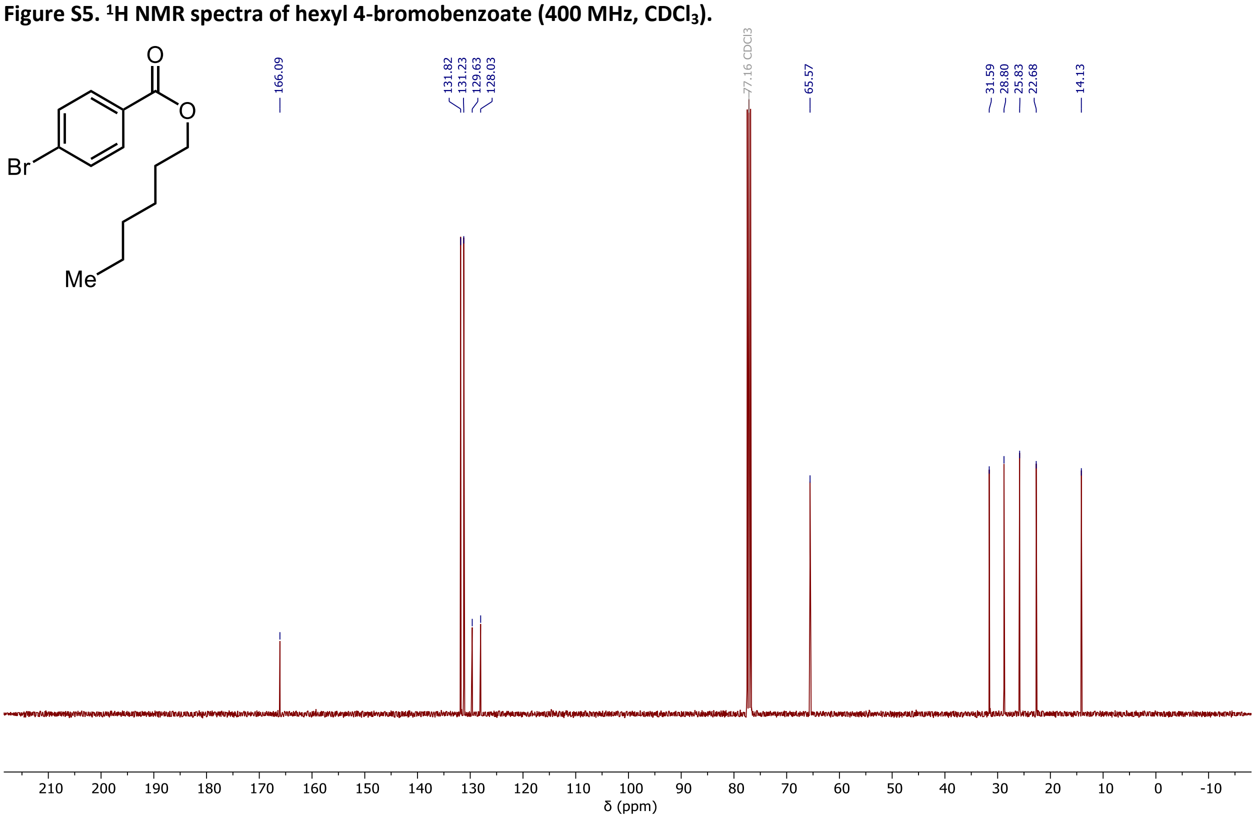


**Figure S6.** **$^{13}C$ NMR spectra of hexyl 4-bromobenzoate (101 MHz, $CDCl_3$).**

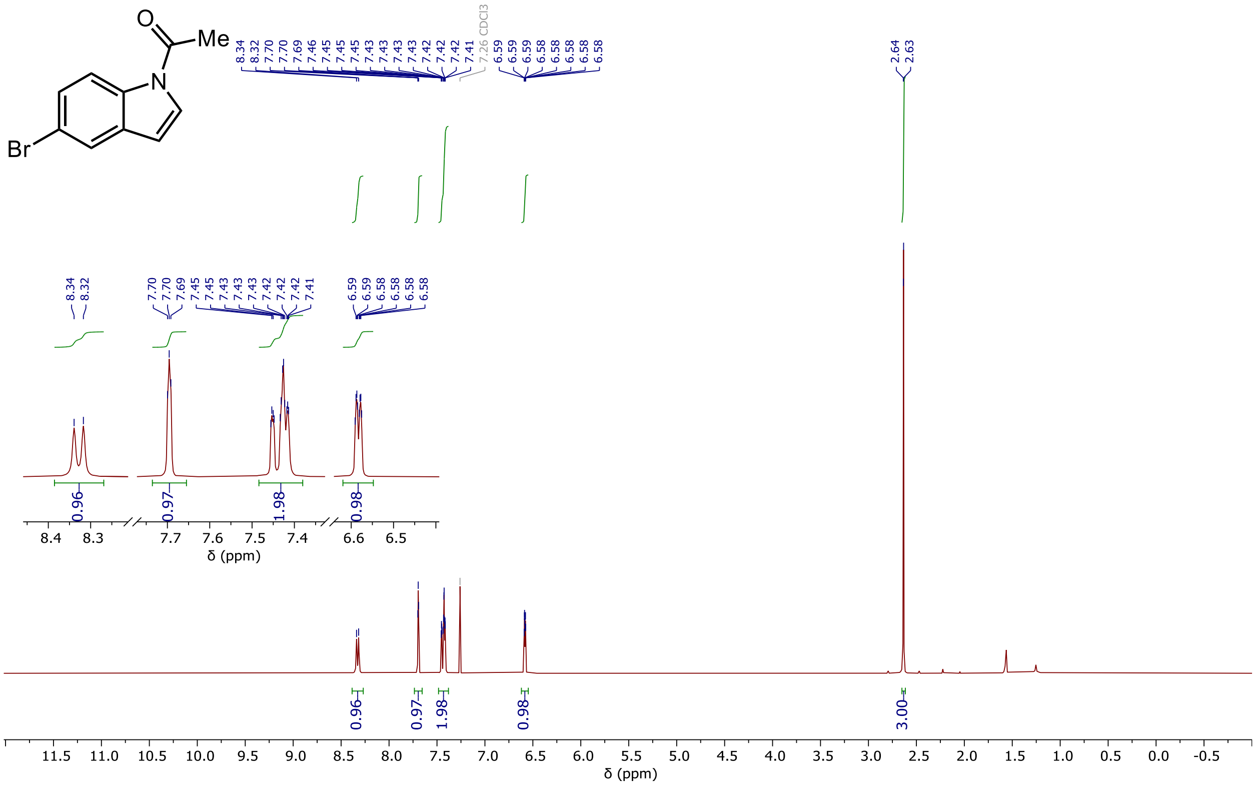


**Figure S7.** **$^{1}$H NMR spectra of 1-acetyl-5-bromoindole (400 MHz, $CDCl_3$).**

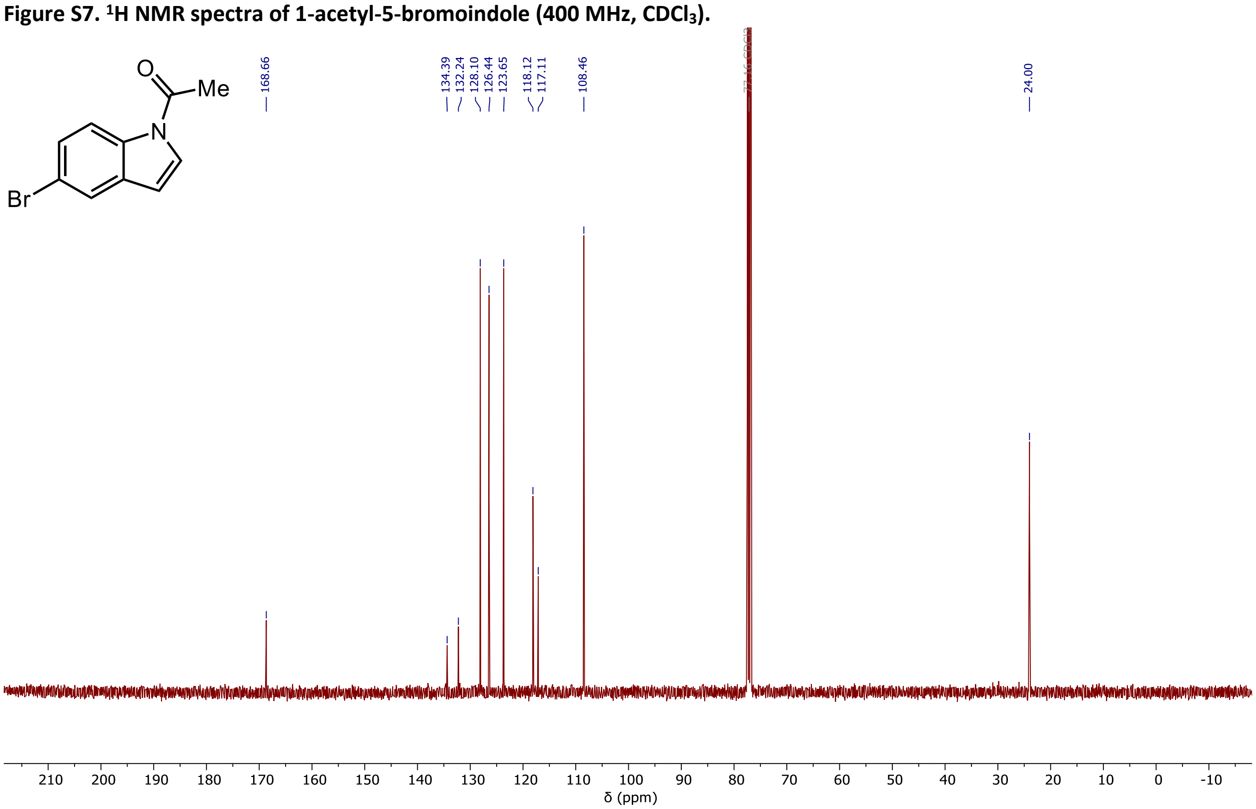


**Figure S8.** **$^{13}$C NMR spectra of 1-acetyl-5-bromoindole (101 MHz, $CDCl_3$).**

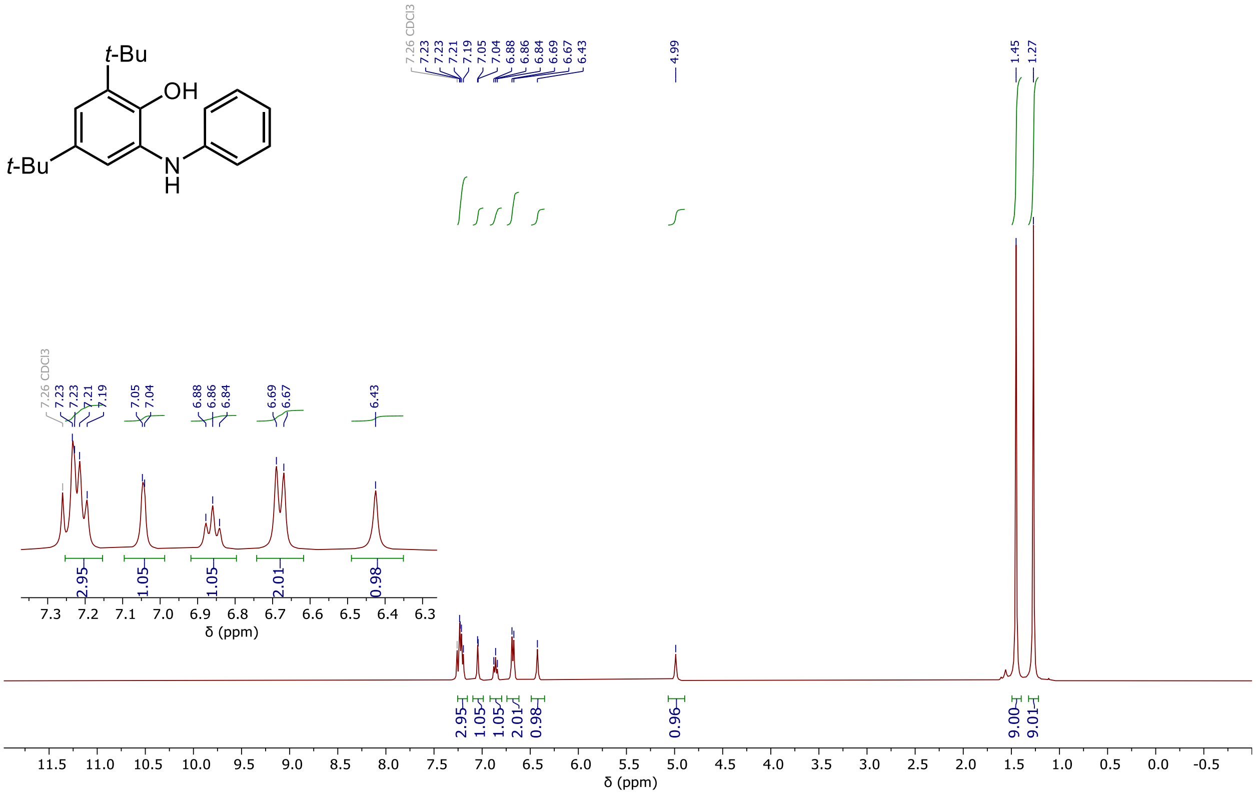

**Figure S9. $^1$H NMR spectra of 2,4-di-tert-butyl-6-(phenylamino)phenol (400 MHz, $CDCl_3$).**

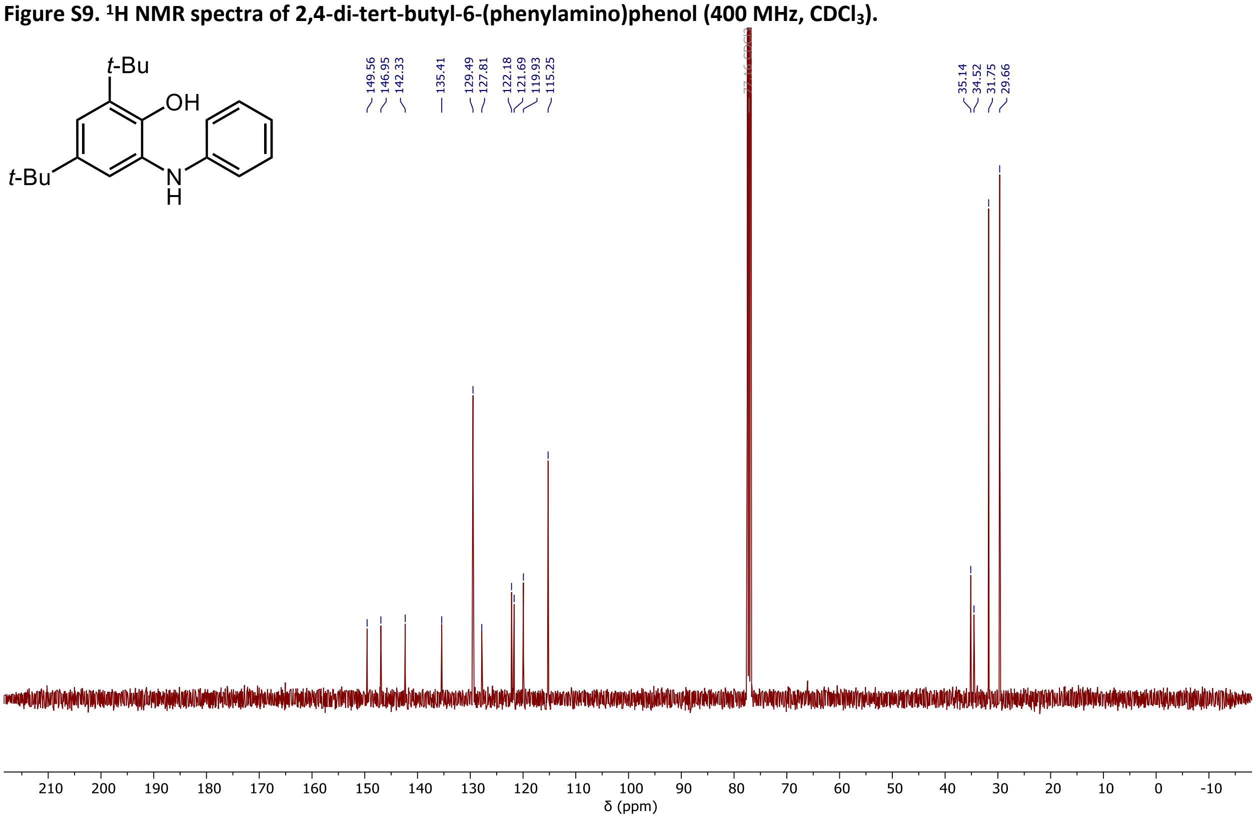

**Figure S10. $^{13}$C NMR spectra of 2,4-di-tert-butyl-6-(phenylamino)phenol (101 MHz, $CDCl_3$).**

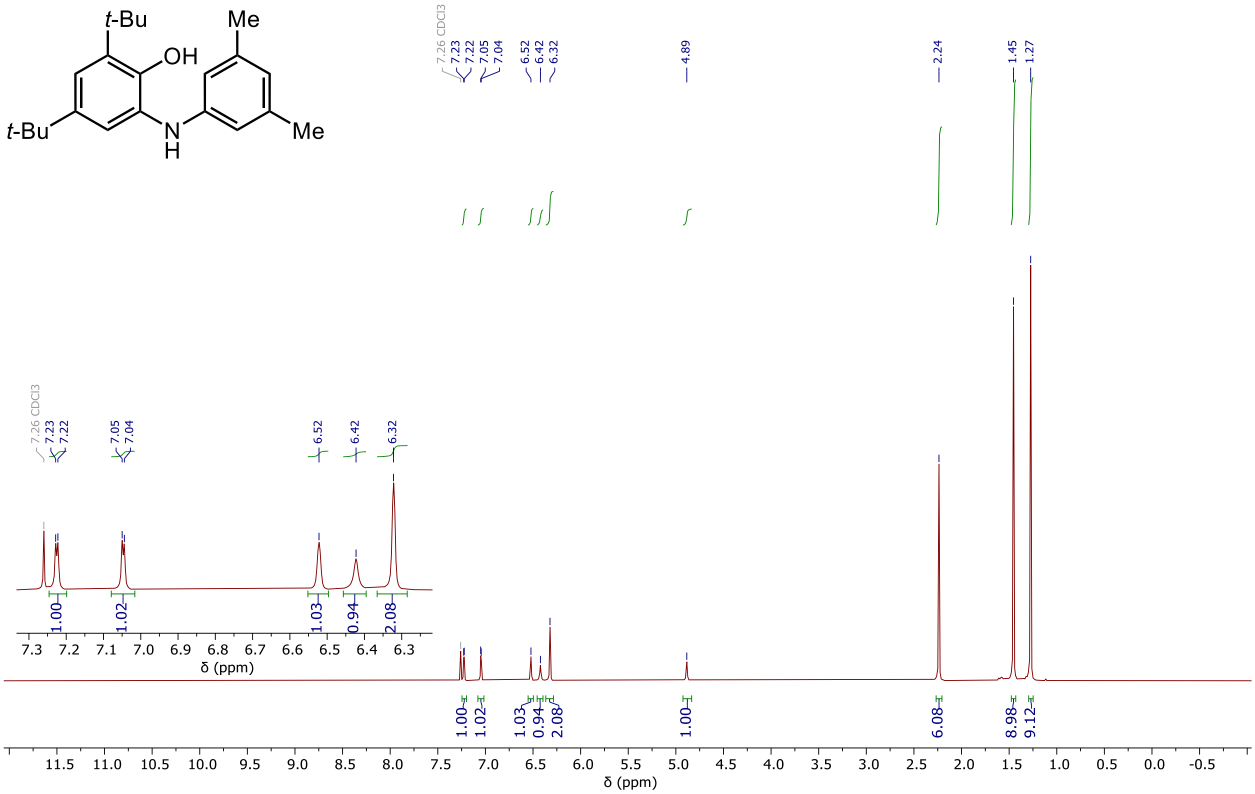

**Figure S11.** $^{1}H$ **NMR spectra of 2,4-di-tert-butyl-6-(3,5-dimethylanilino)phenol (400 MHz,** $CDCl_3$**).**

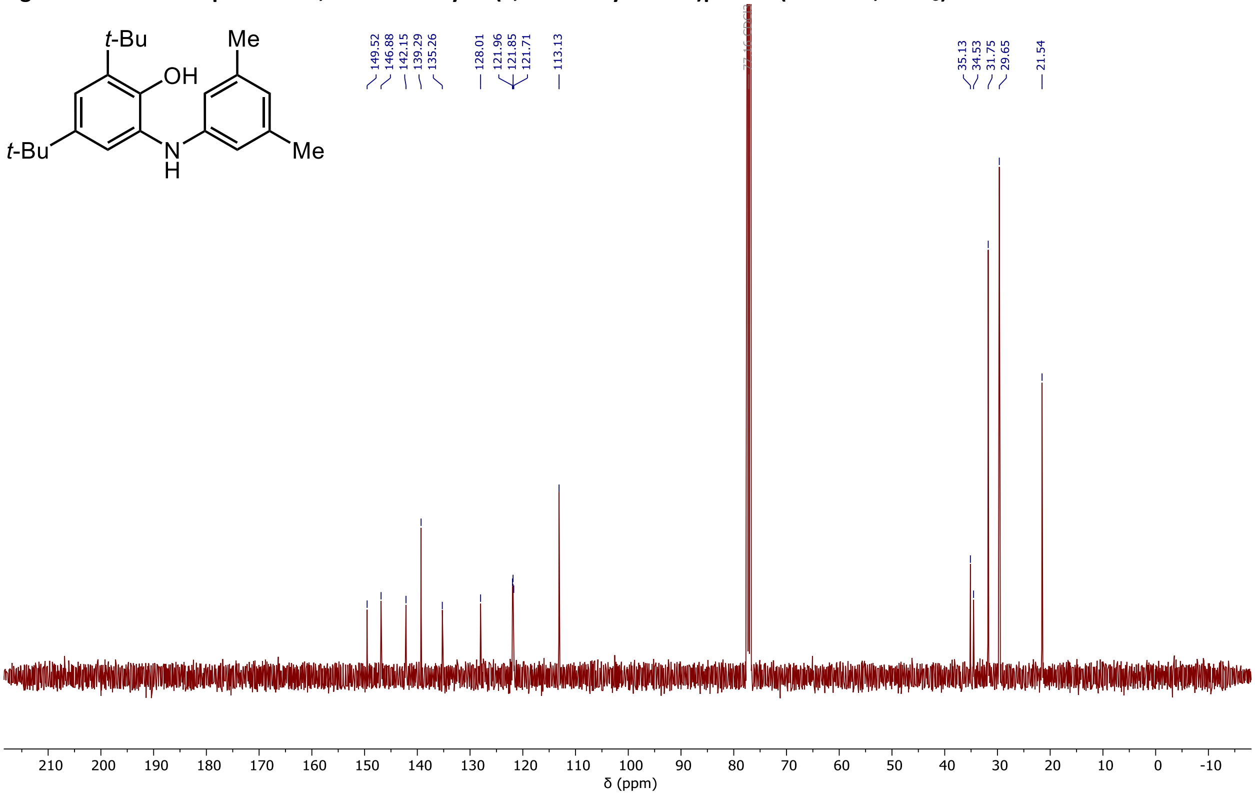

**Figure S12.** $^{13}C$ **NMR spectra of 2,4-di-tert-butyl-6-(3,5-dimethylanilino)phenol (101 MHz,** $CDCl_3$**).**

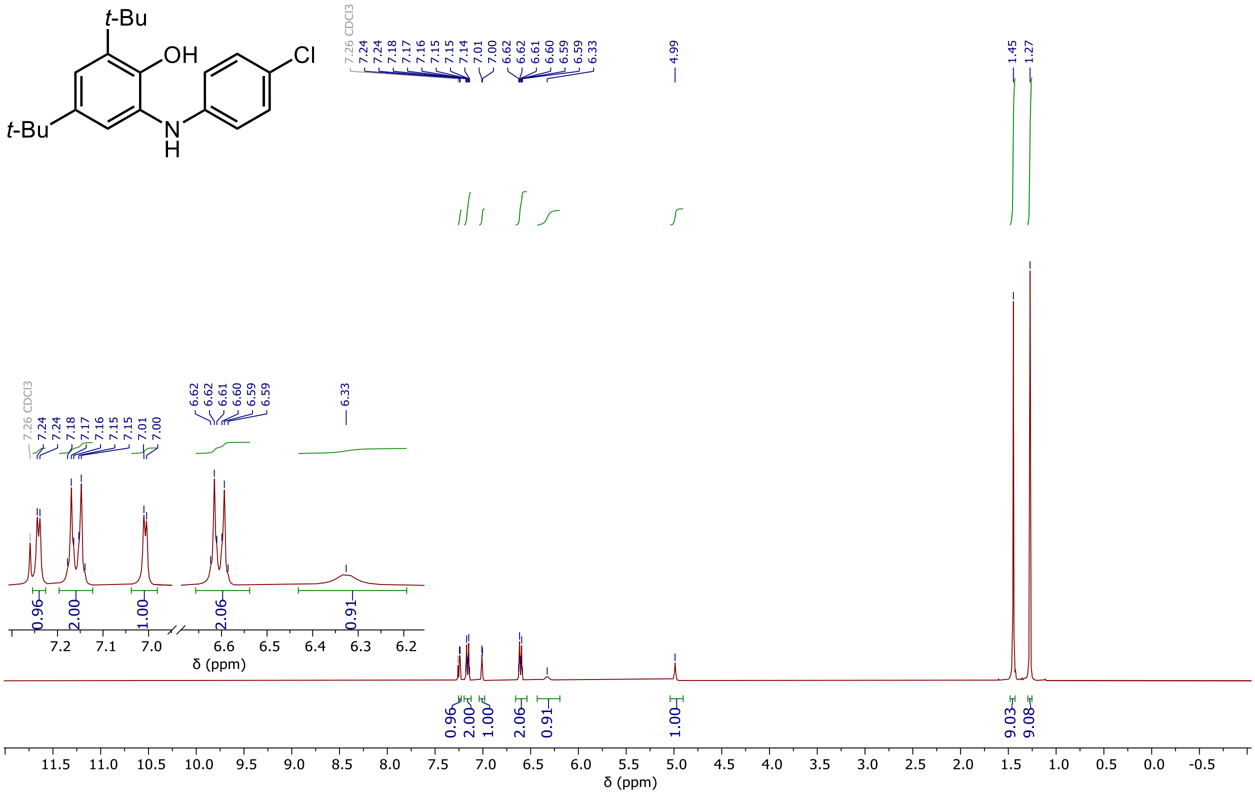

**Figure S13.** $^1$H NMR spectra of 2,4-di-tert-butyl-6-(4-chloroanilino)phenol (400 MHz, $CDCl_3$).

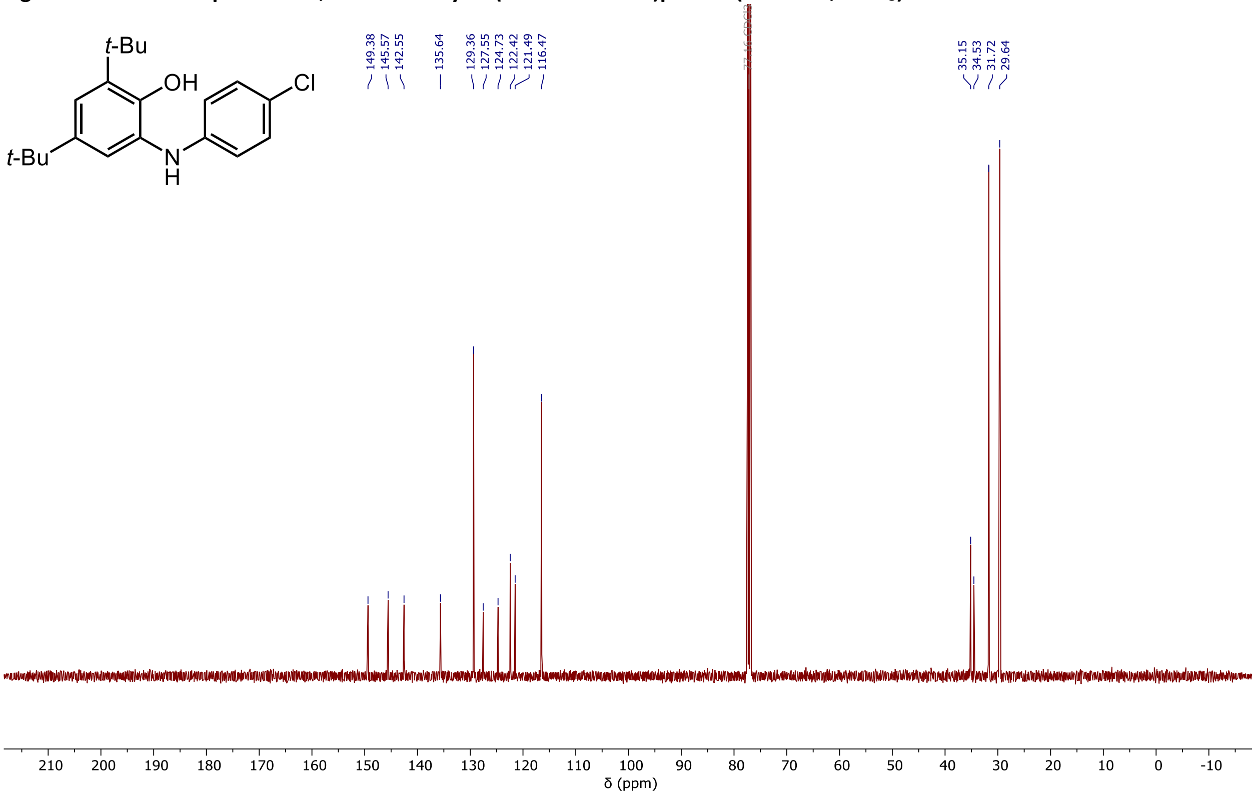

**Figure S14.** $^{13}$C NMR spectra of 2,4-di-tert-butyl-6-(4-chloroanilino)phenol (101 MHz, $CDCl_3$).

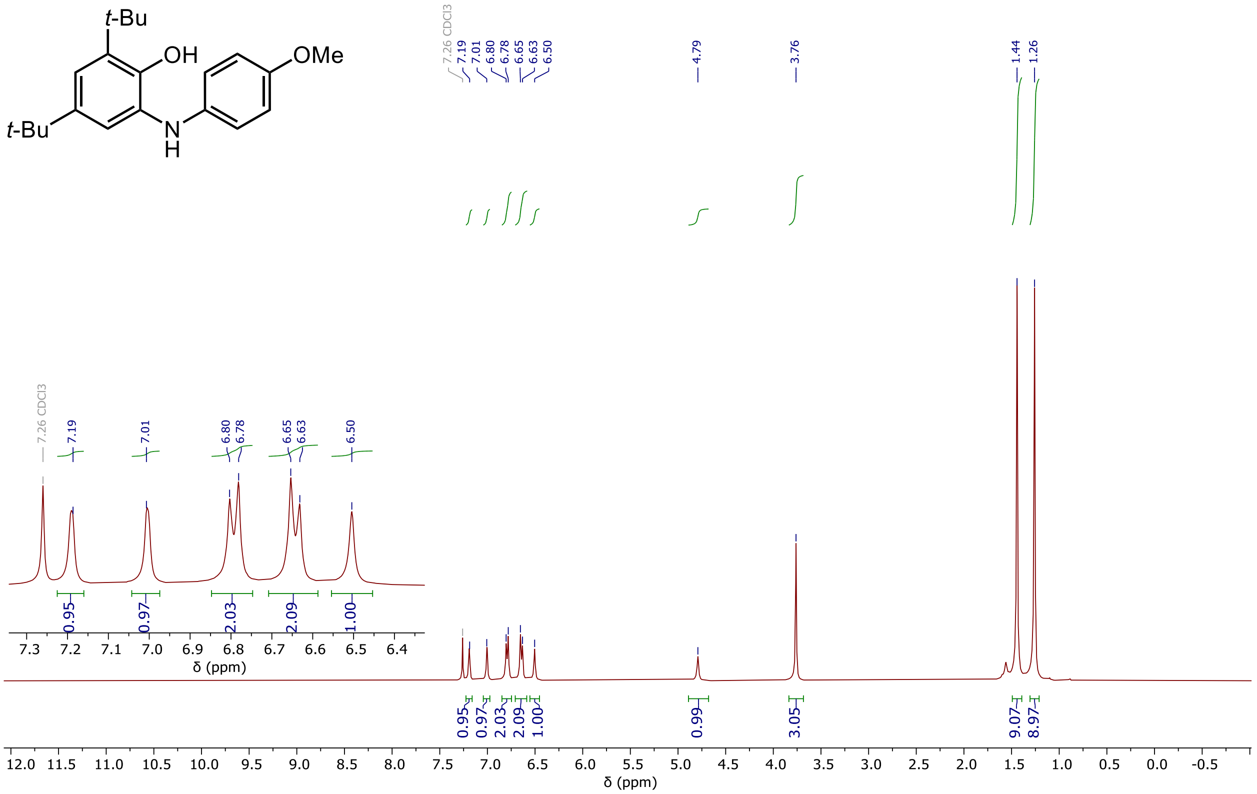

**Figure S15.** $^{1}H$ **NMR spectra of 2,4-di-tert-butyl-6-(4-methoxyanilino)phenol (400 MHz,** $CDCl_3$**).**

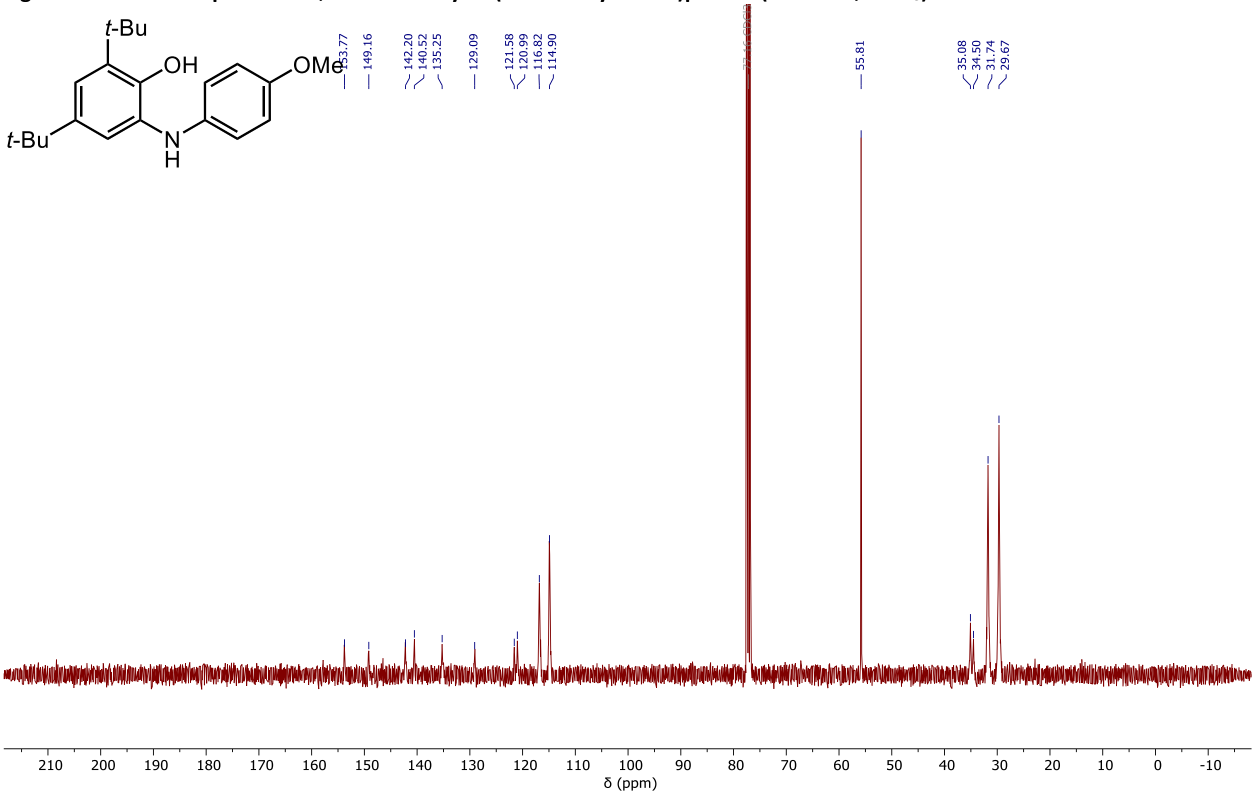

**Figure S16.** $^{13}C$ **NMR spectra of 2,4-di-tert-butyl-6-(4-methoxyanilino)phenol (101 MHz,** $CDCl_3$**).**

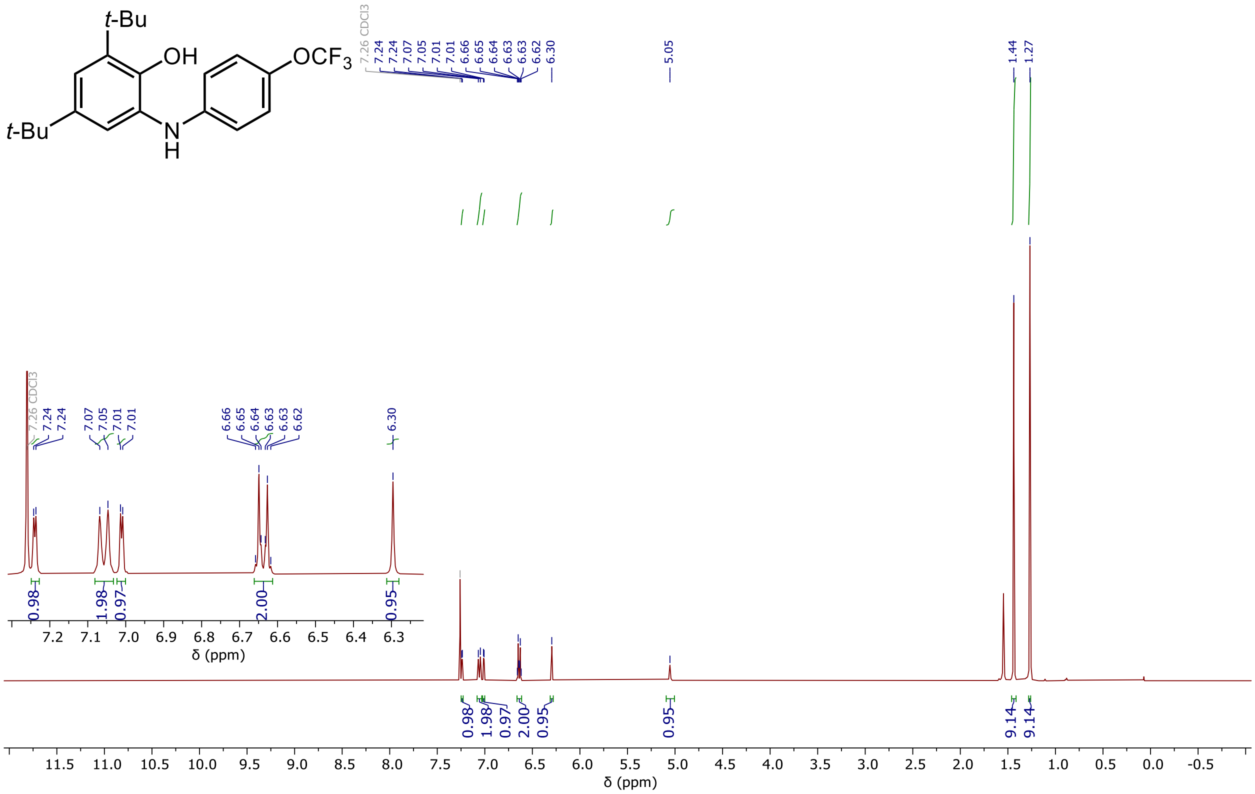

**Figure S17.** **$^{1}$H NMR spectra of 2,4-di-tert-butyl-6-(4-trifluoromethoxyanilino)phenol (400 MHz, $CDCl_3$).**

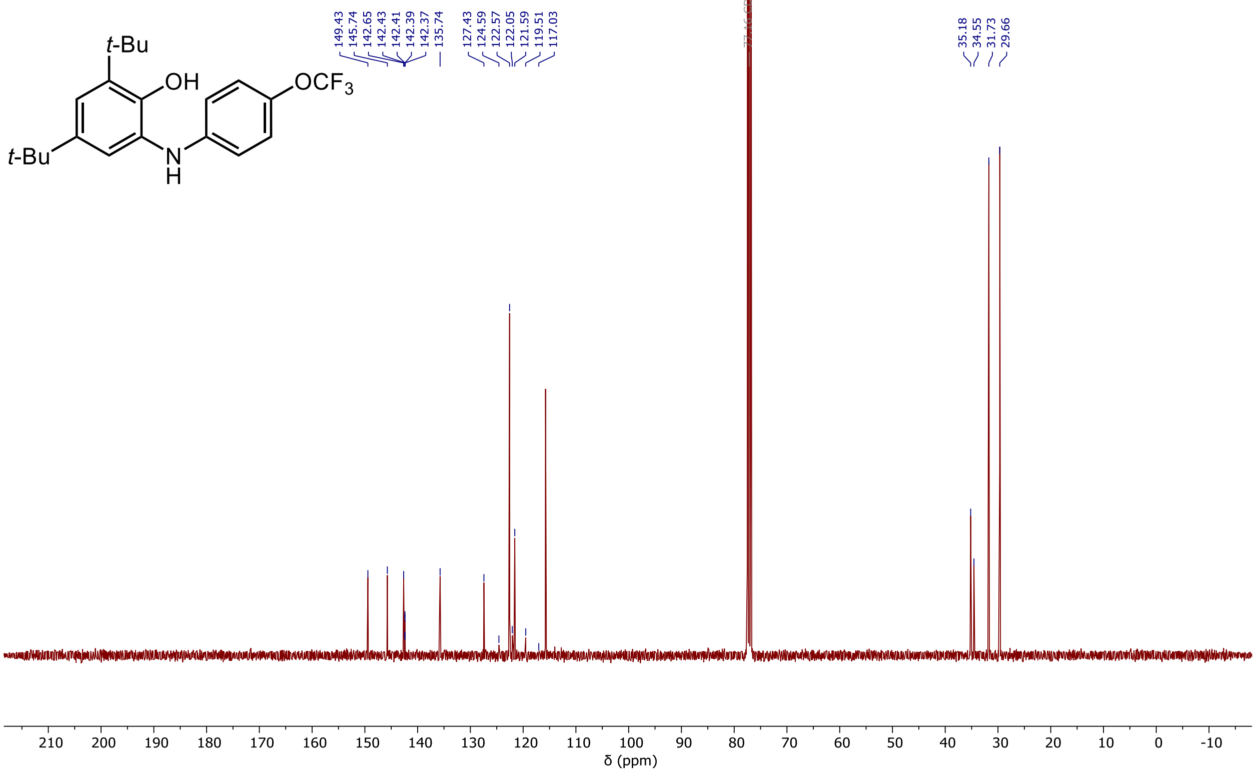

**Figure S18.** **$^{13}$C NMR spectra of 2,4-di-tert-butyl-6-(4-trifluoromethoxyanilino)phenol (101 MHz, CDCl3).**

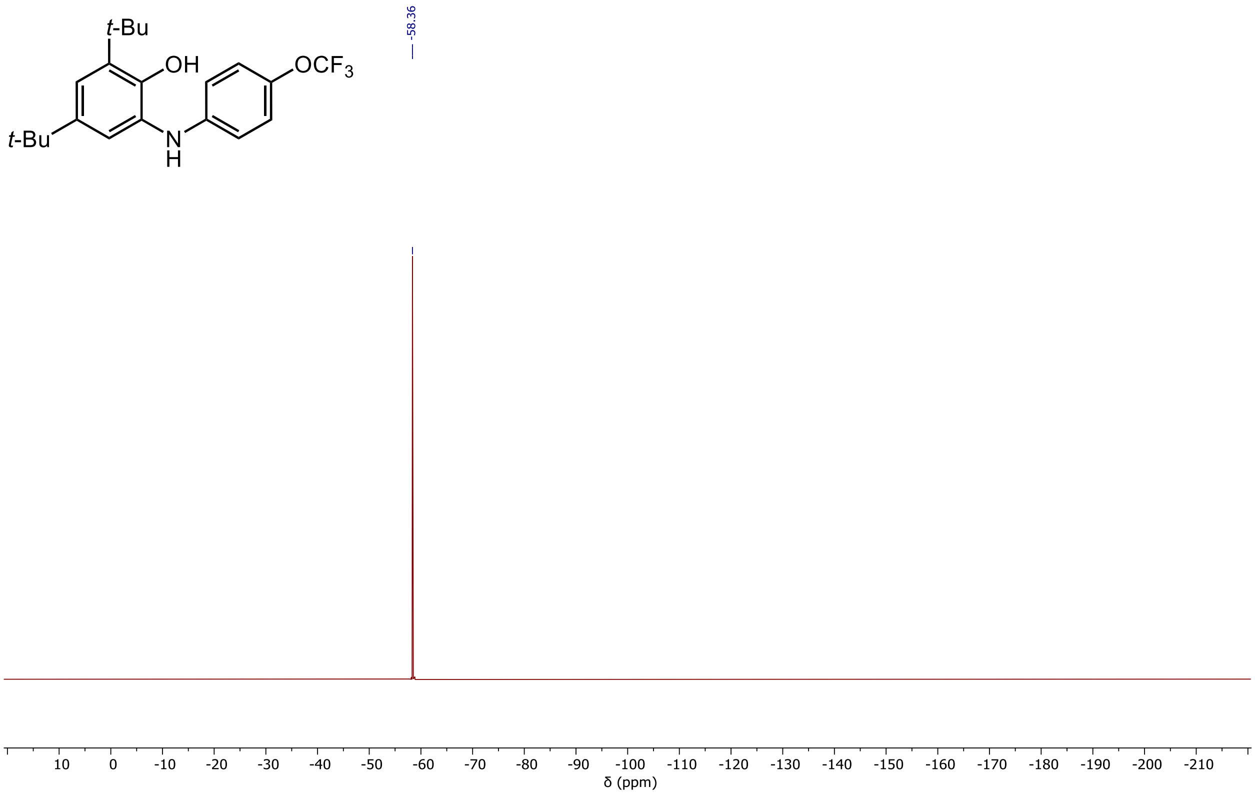

**Figure S19.** **$^{19}$F NMR spectra of 2,4-di-tert-butyl-6-(4-trifluoromethoxyanilino)phenol (376 MHz, $CDCl_3$).**

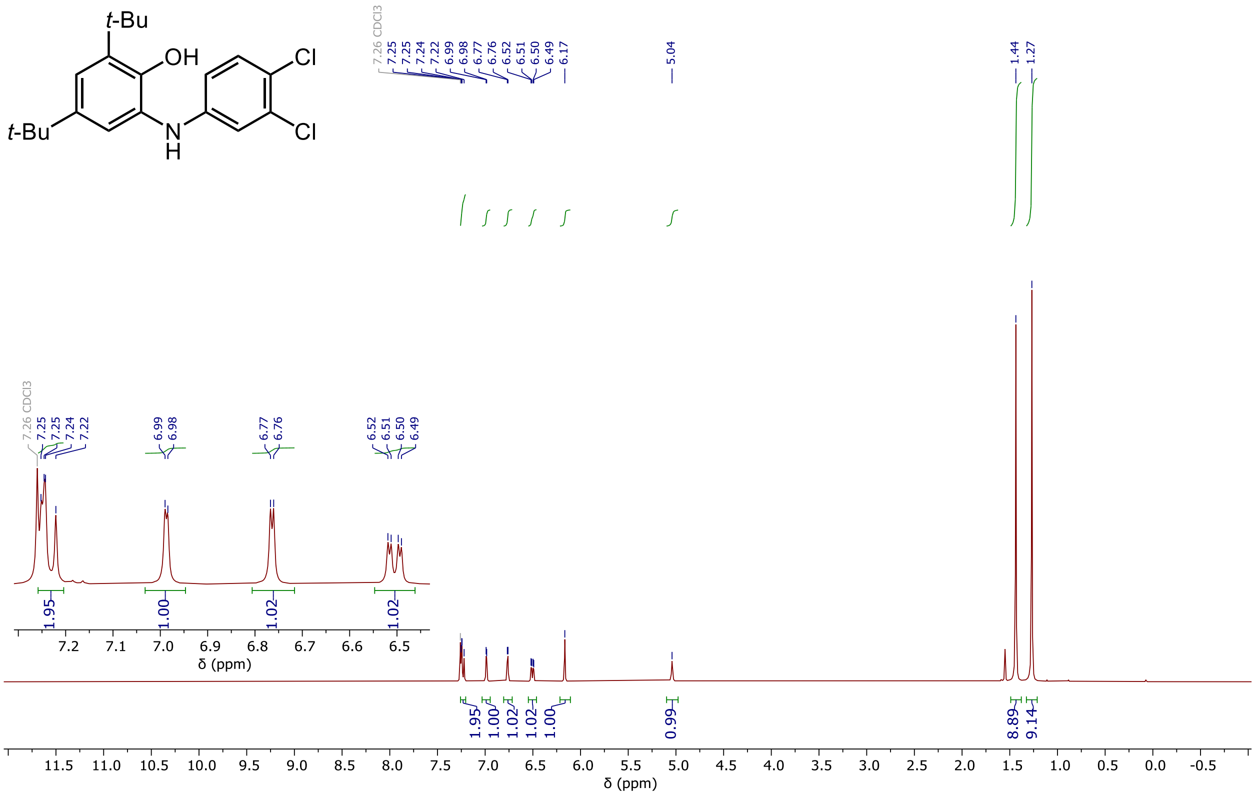

**Figure S20.** **$^{1}$H NMR spectra of 2,4-di-tert-butyl-6-(3,4-dichloroanilino)phenol (400 MHz, $CDCl_3$).**

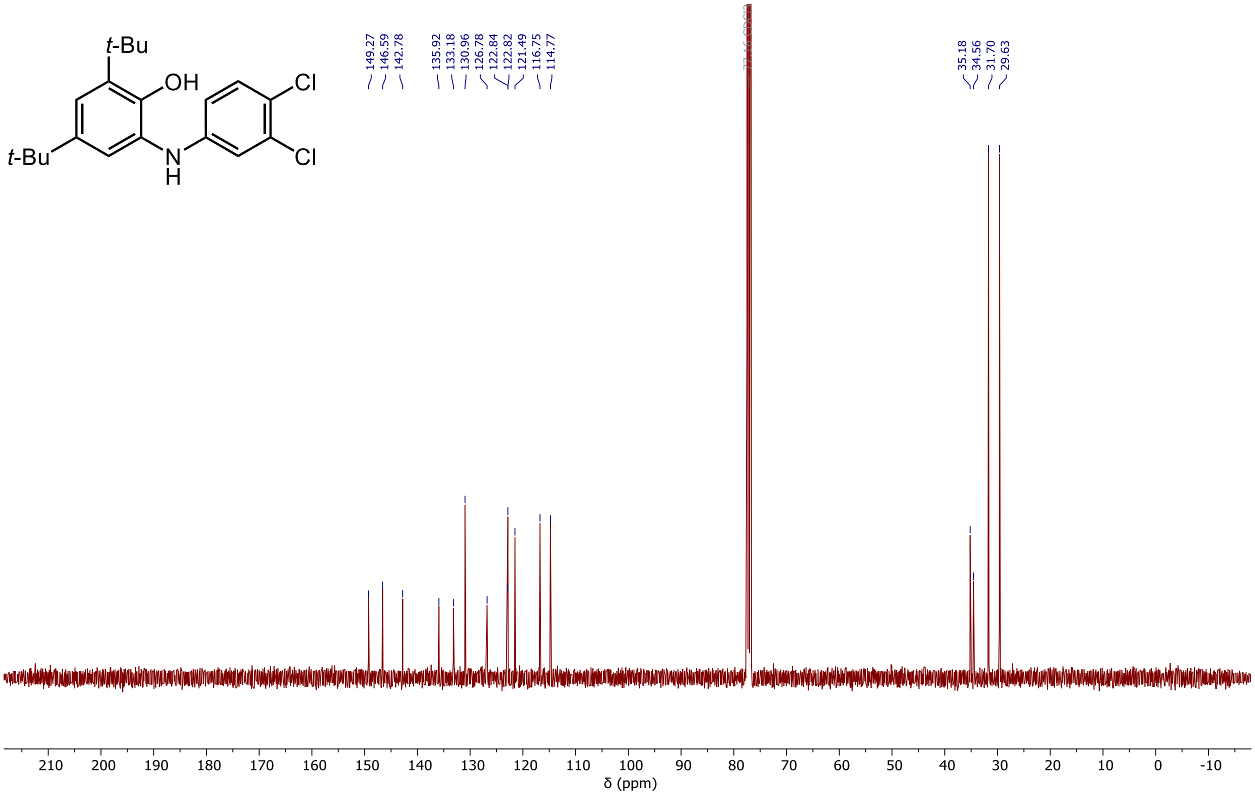

**Figure S21.** $^{13}$**C NMR spectra of 2,4-di-tert-butyl-6-(3,4-dichloroanilino)phenol (101 MHz, $CDCl_3$).**

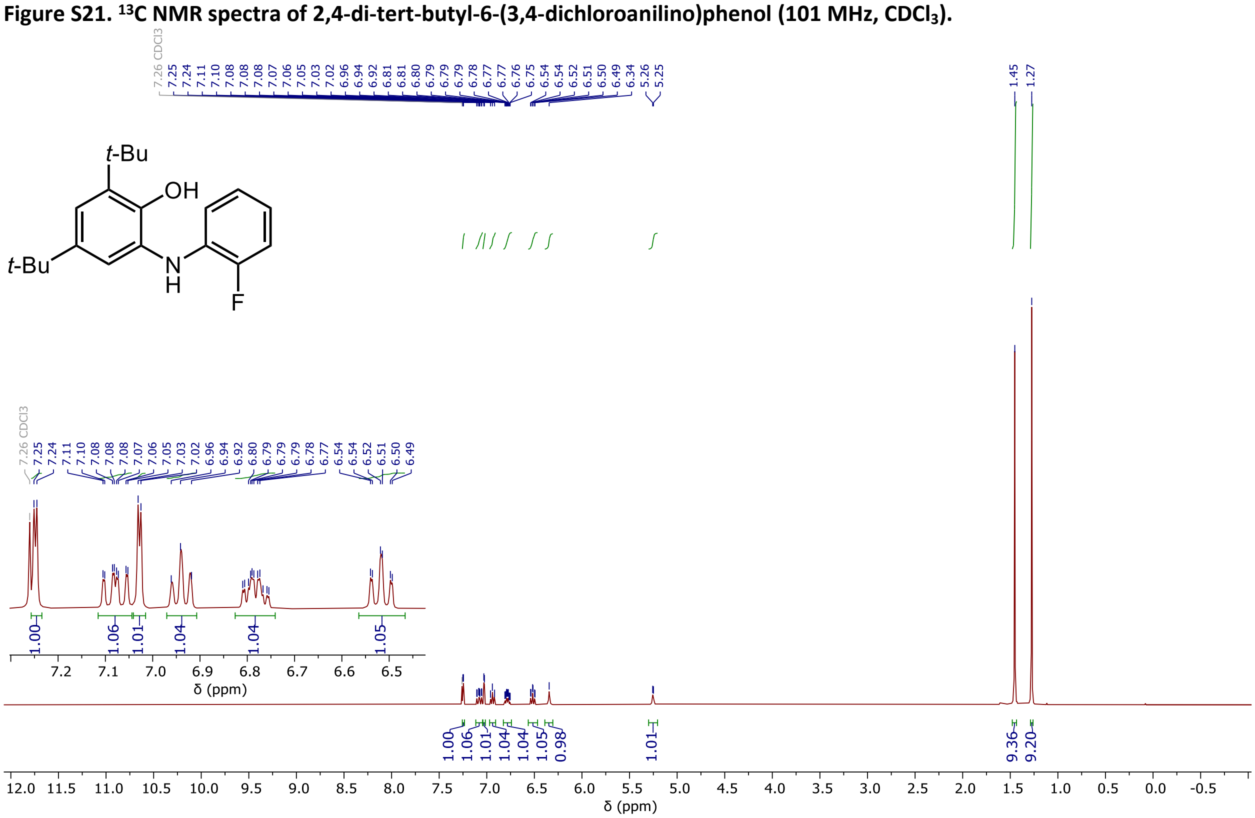

**Figure S22.** $^{1}$**H NMR spectra of 2,4-di-tert-butyl-6-(2-fluoroanilino)phenol (400 MHz, $CDCl_3$).**

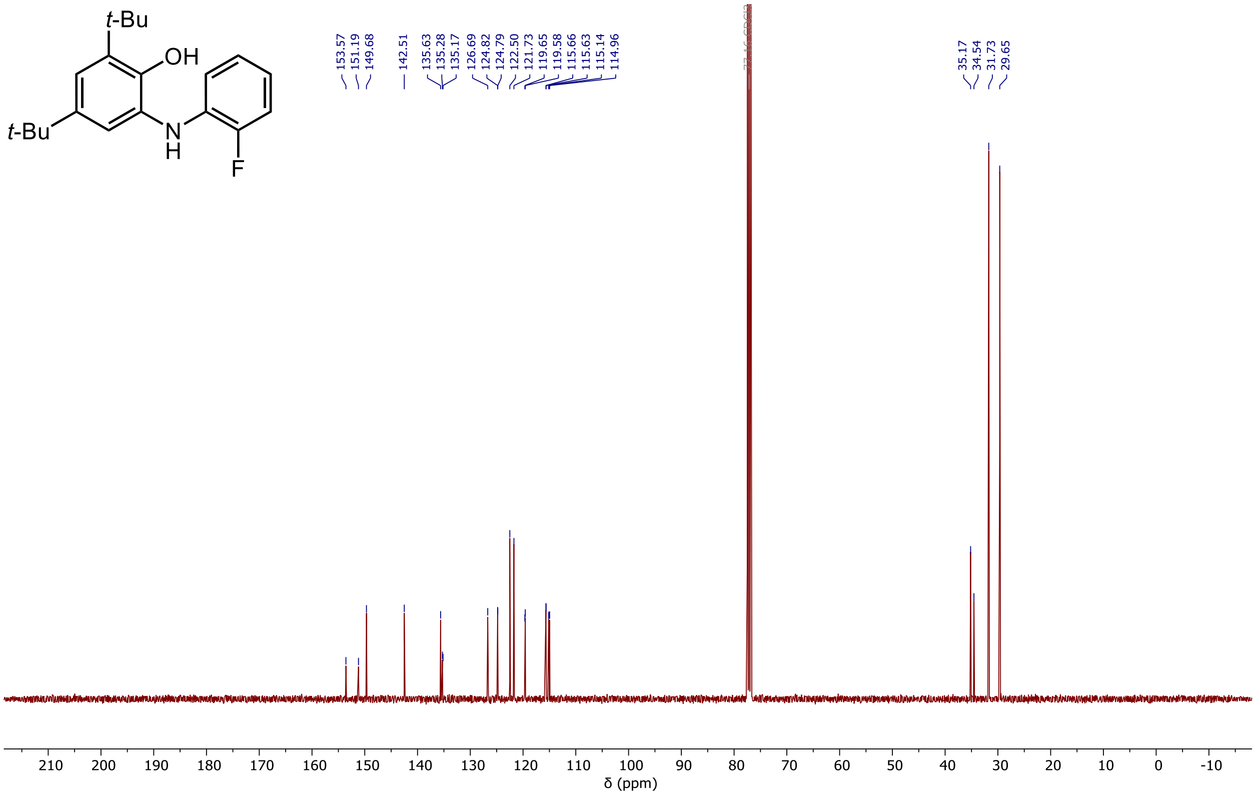


**Figure S23.** $^{13}$**C NMR spectra of 2,4-di-tert-butyl-6-(2-fluoroanilino)phenol (101 MHz, CDCl3).**

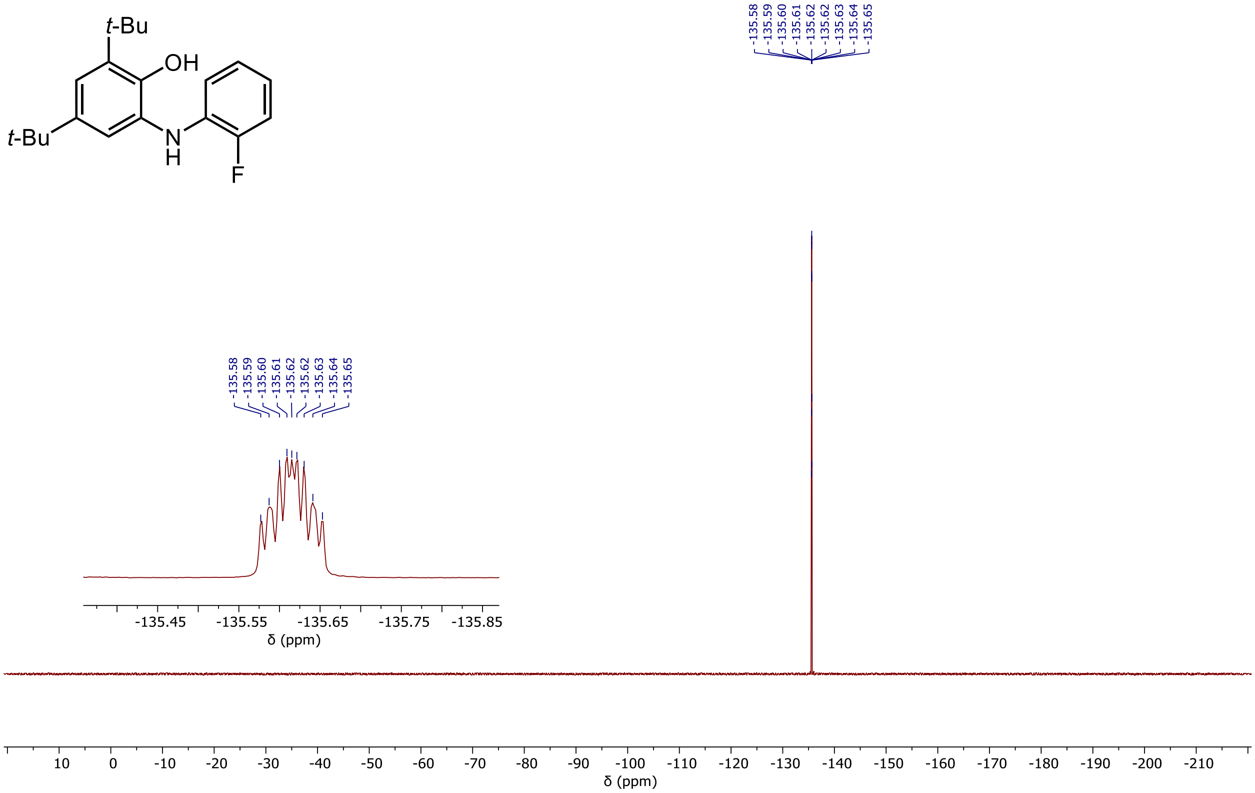


**Figure S24.** $^{19}$**F NMR spectra of 2,4-di-tert-butyl-6-(2-fluoroanilino)phenol (376 MHz, $CDCl_3$).**

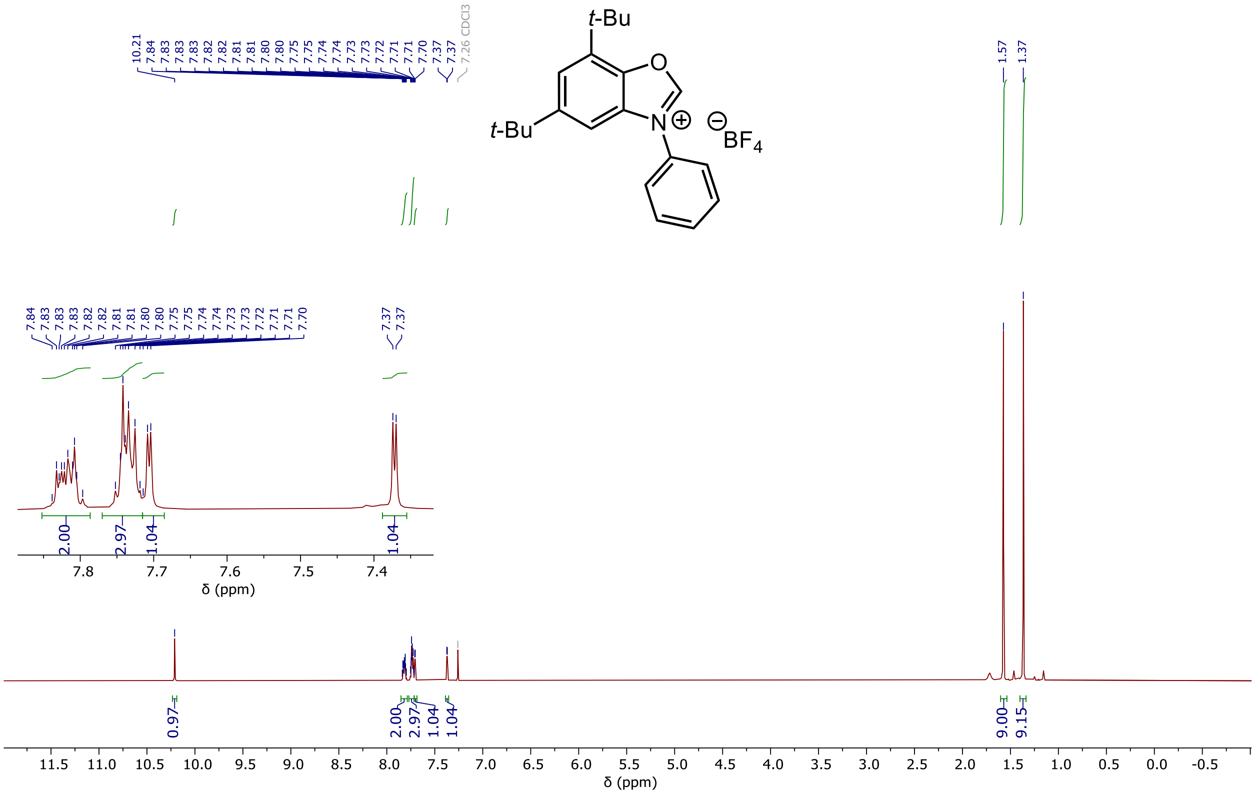

**Figure S25.** **$^{1}H$ NMR spectra of 5,7-di-tert-butyl-3-phenylbenzo[d]oxazol-3-ium tetrafluoroborate (NHC-1) (400 MHz, $CDCl_3$).**

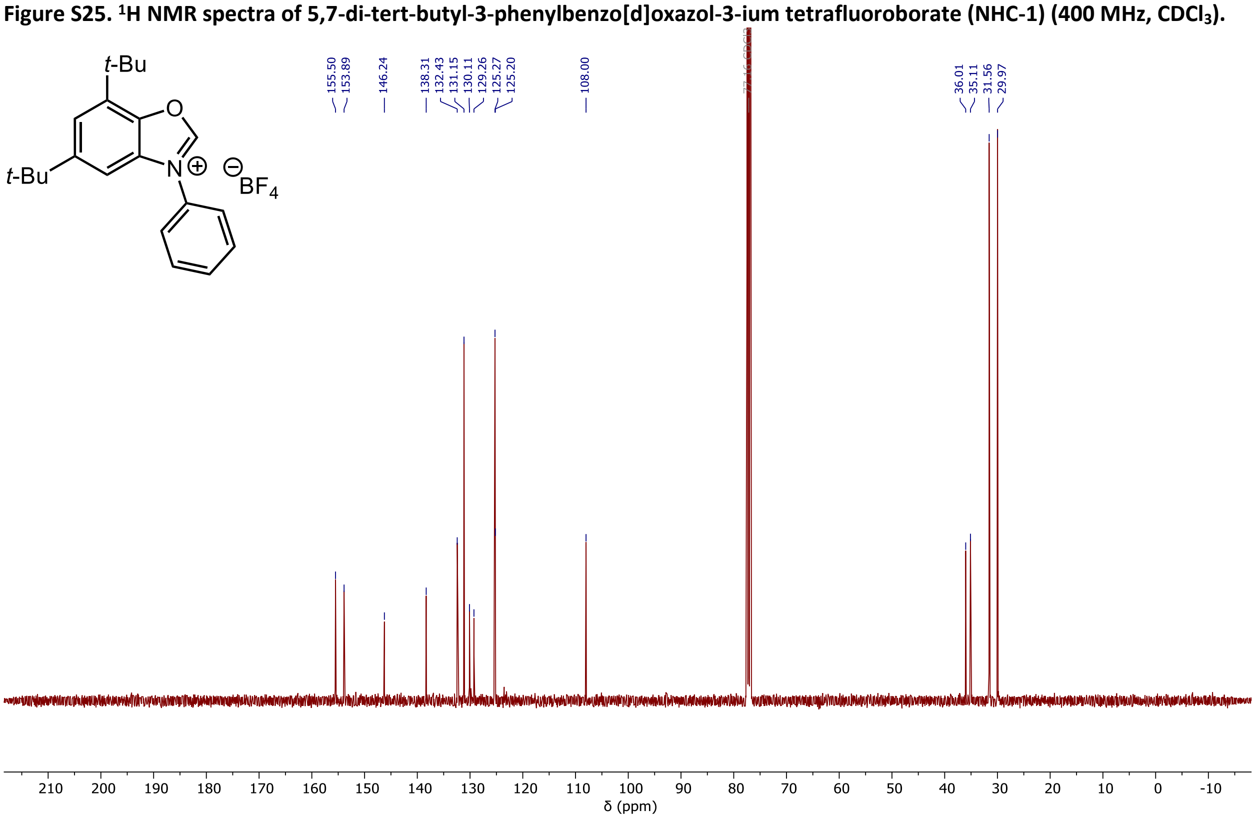

**Figure S26.** **$^{13}C$ NMR spectra of 5,7-di-tert-butyl-3-phenylbenzo[d]oxazol-3-ium tetrafluoroborate (NHC-1) (101 MHz, $CDCl_3$).**

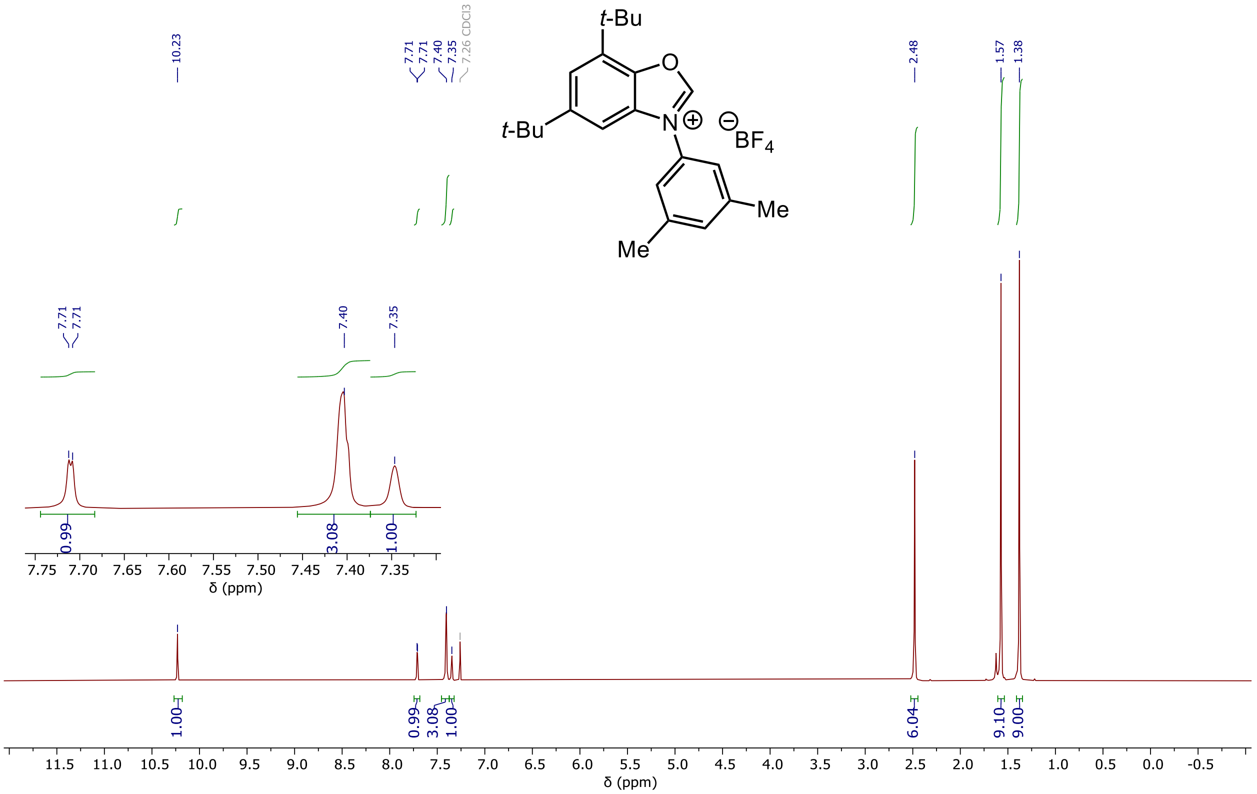


**Figure S27.** $^{1}H$ **NMR spectra of 5,7-di-tert-butyl-3-(3,5-dimethylphenyl)benzo[d]oxazol-3-ium tetrafluoroborate (NHC-2) (400 MHz,** $CDCl_3$**).**

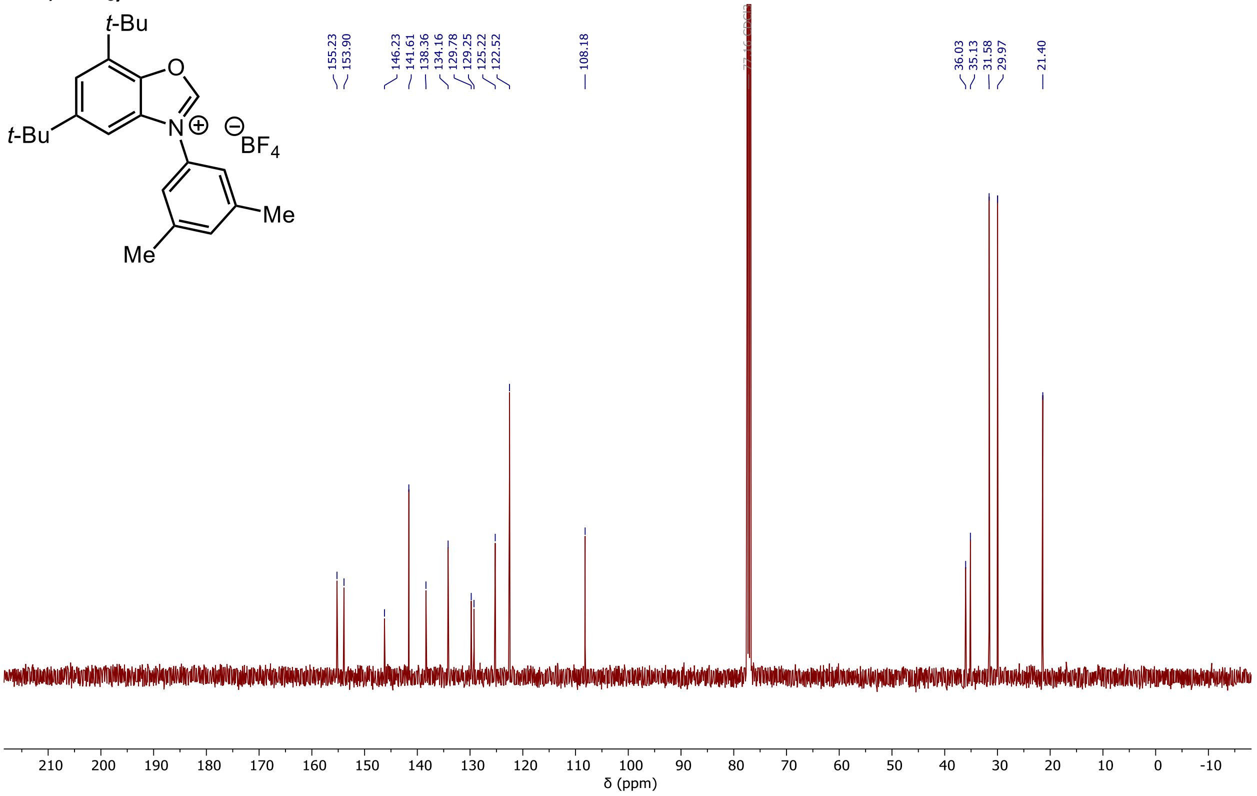


**Figure S28.** $^{13}C$ **NMR spectra of 5,7-di-tert-butyl-3-(3,5-dimethylphenyl)benzo[d]oxazol-3-ium tetrafluoroborate (NHC-2) (101 MHz,** $CDCl_3$**).**

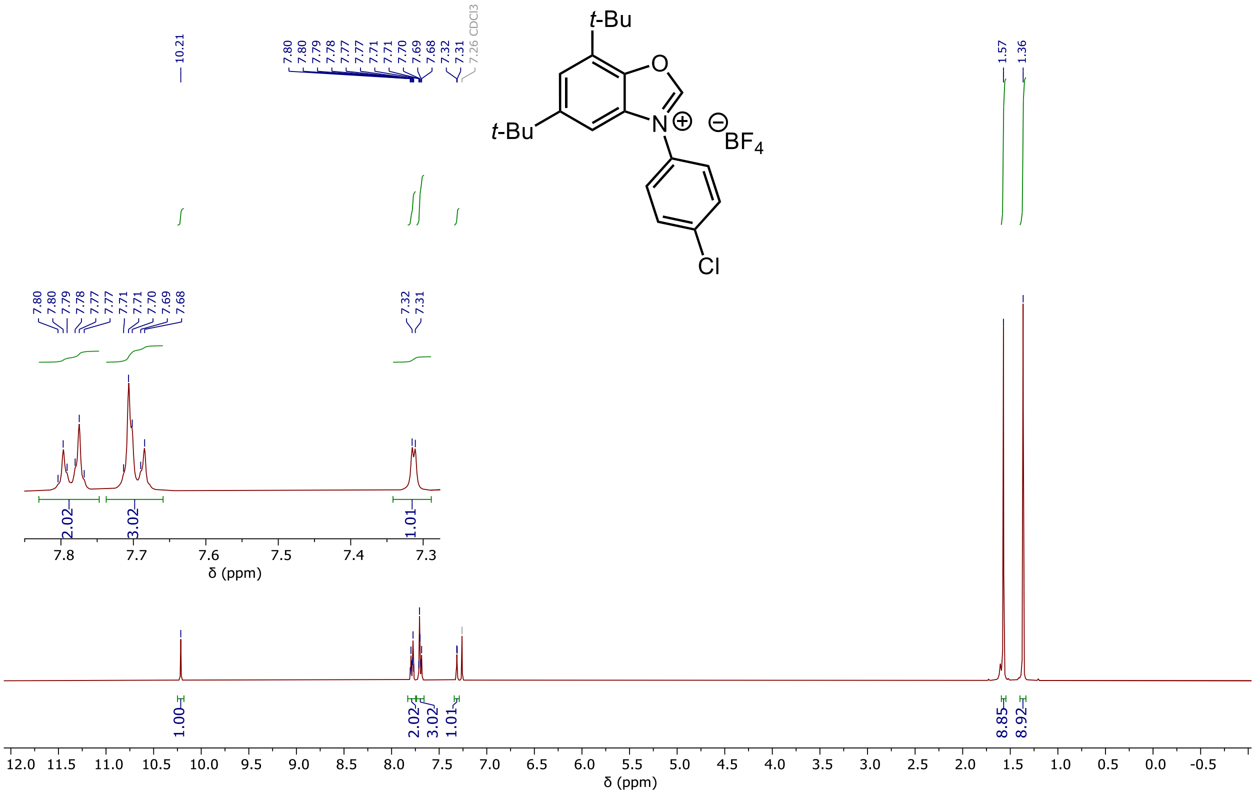


**Figure S29.** $^{1}H$ **NMR spectra of 5,7-di-tert-butyl-3-(4-chlorophenyl)benzo[d]oxazol-3-ium tetrafluoroborate (NHC-3) (400 MHz,** $CDCl_3$**).**

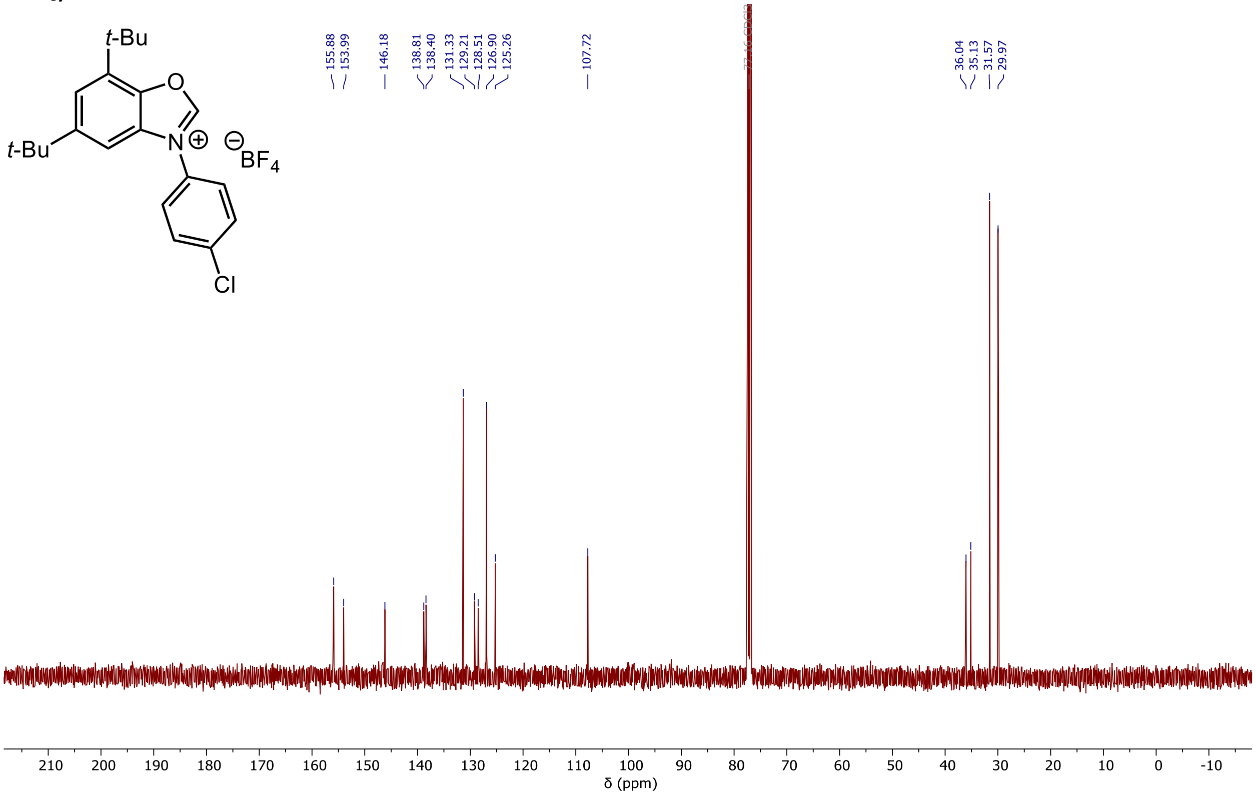


**Figure S30.** $^{13}C$ **NMR spectra of 5,7-di-tert-butyl-3-(4-chlorophenyl)benzo[d]oxazol-3-ium tetrafluoroborate (NHC-3) (101 MHz,** $CDCl_3$**).**

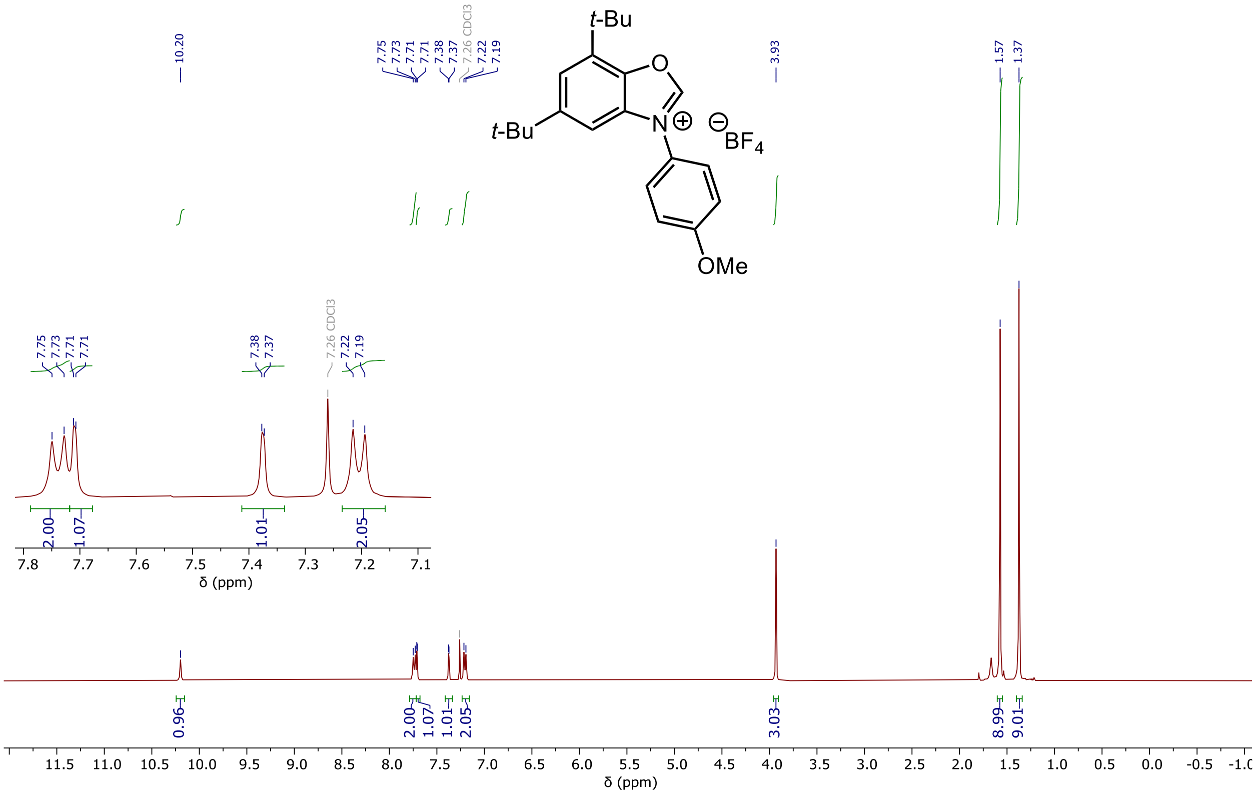

**Figure S31.** **$^{1}H$ NMR spectra of 5,7-di-tert-butyl-3-(4-methoxyphenyl)benzo[d]oxazol-3-ium tetrafluoroborate (NHC-4) (400 MHz, $CDCl_3$).**

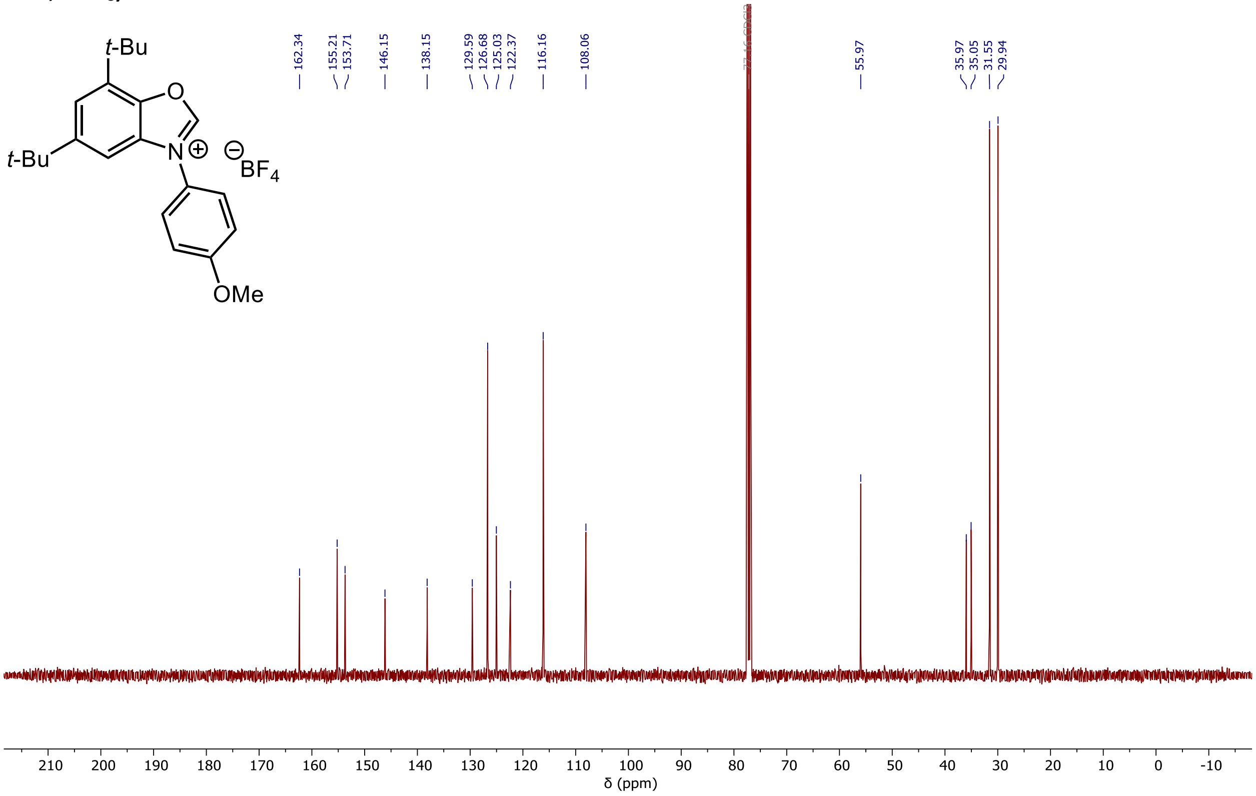

**Figure S32.** **$^{13}C$ NMR spectra of 5,7-di-tert-butyl-3-(4-methoxyphenyl)benzo[d]oxazol-3-ium tetrafluoroborate (NHC-4) (101 MHz, $CDCl_3$).**

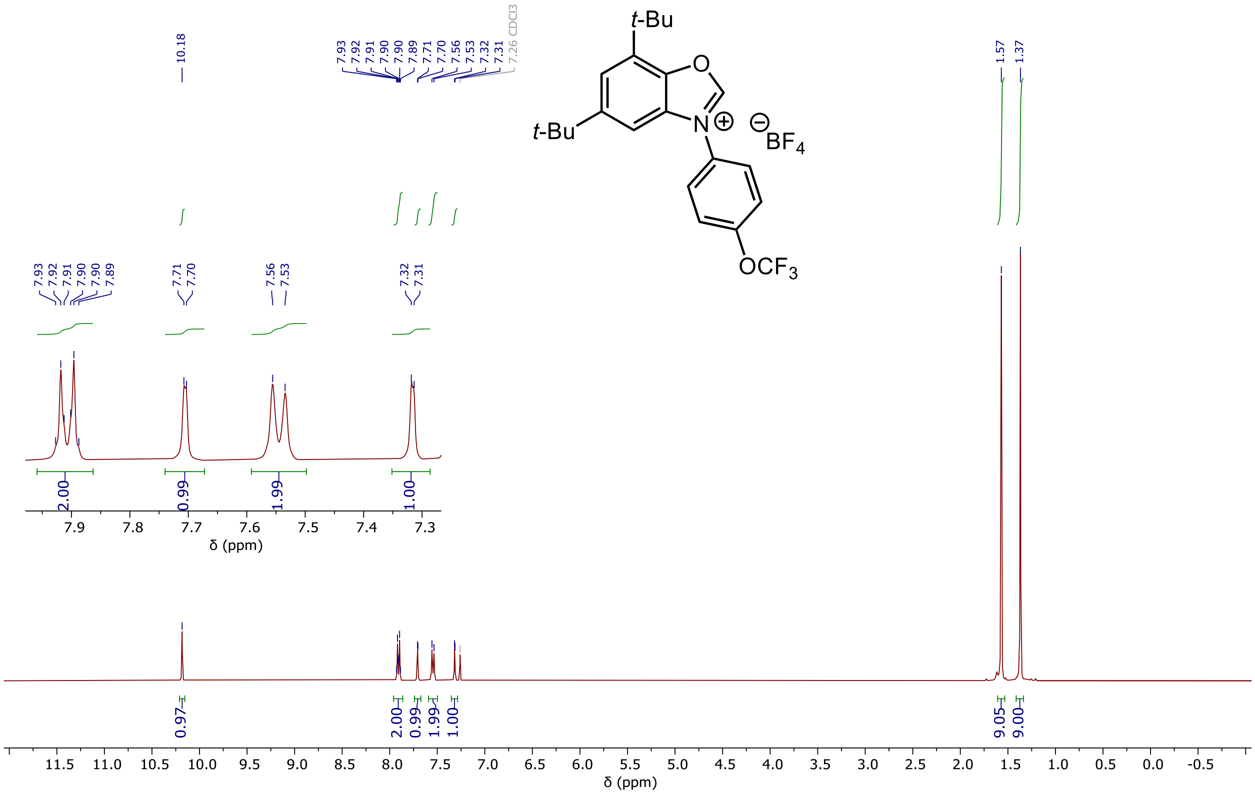

**Figure S33.** **$^{1}$H NMR spectra of 5,7-di-tert-butyl-3-(4-(trifluoromethoxy)phenyl)benzo[d]oxazol-3-ium tetrafluoroborate (NHC-5) (400 MHz, CDCl$_3$).**

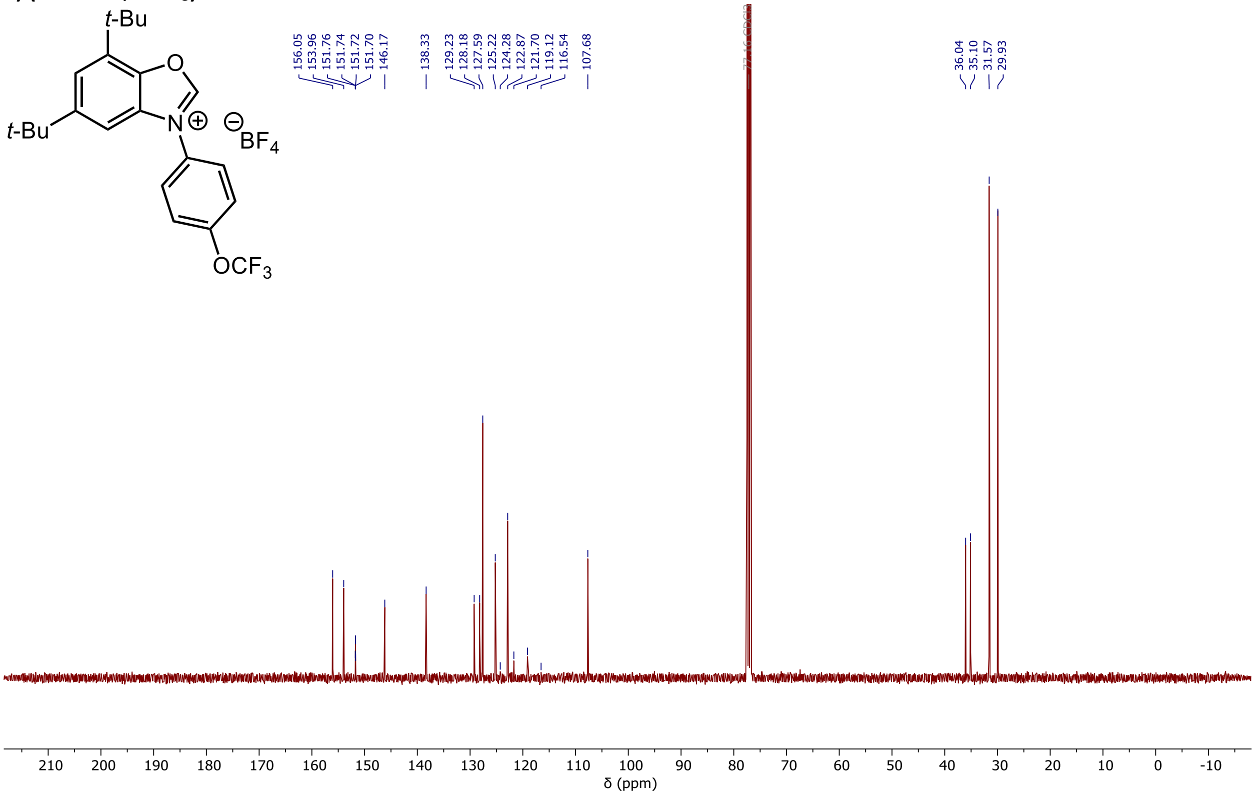

**Figure S34.** **$^{13}$C NMR spectra of 5,7-di-tert-butyl-3-(4-(trifluoromethoxy)phenyl)benzo[d]oxazol-3-ium tetrafluoroborate (NHC-5) (101 MHz, CDCl$_3$).**

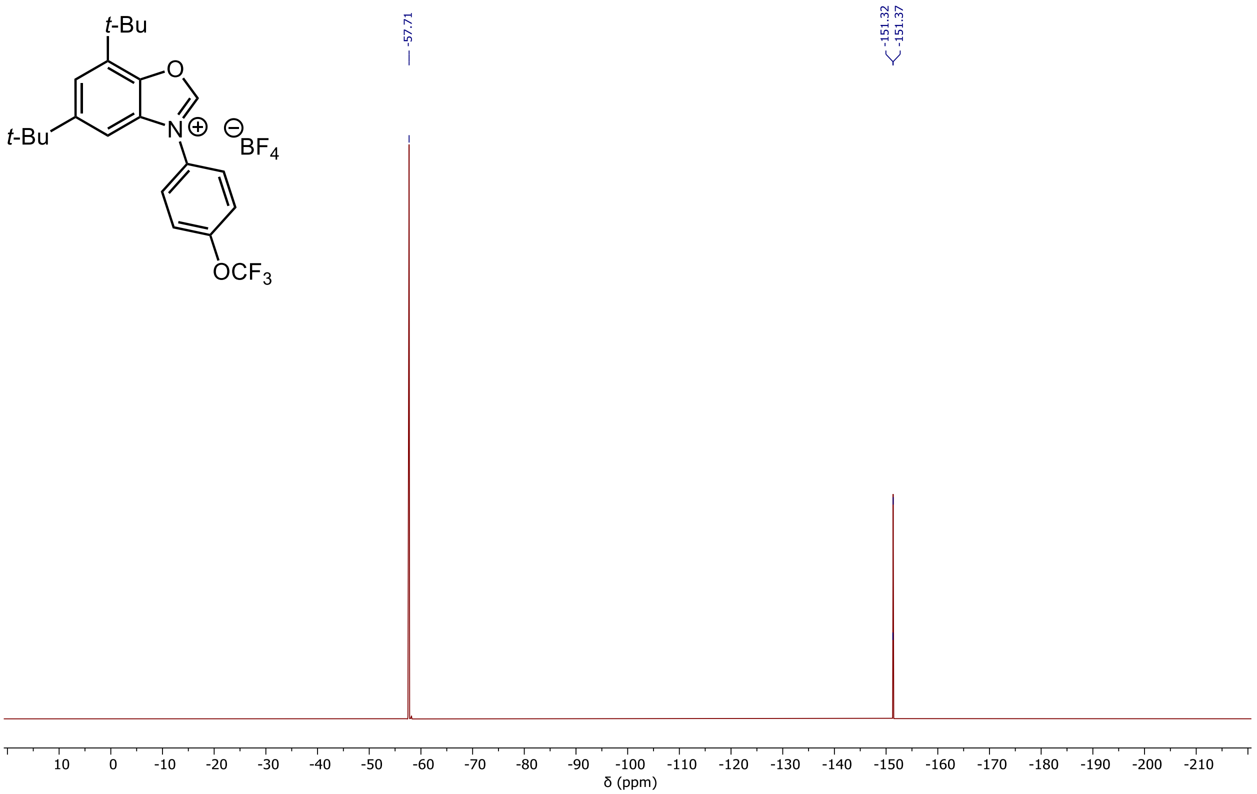

**Figure S35. $^{19}$F NMR spectra of 5,7-di-tert-butyl-3-(4-(trifluoromethoxy)phenyl)benzo[d]oxazol-3-ium tetrafluoroborate (NHC-5) (376 MHz, $CDCl_3$).**

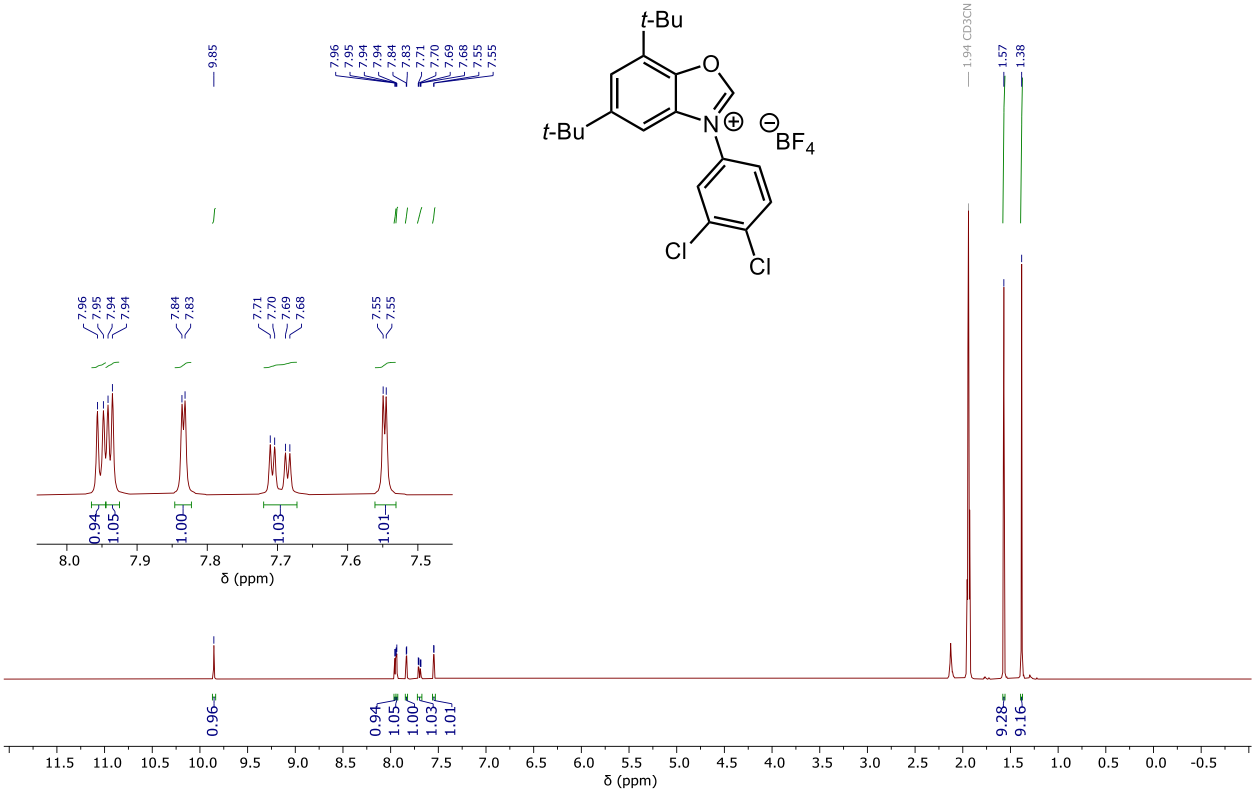

**Figure S36. $^{1}$H NMR spectra of 5,7-di-tert-butyl-3-(3,4-dichlorophenyl)benzo[d]oxazol-3-ium tetrafluoroborate (NHC-6) (400 MHz, $CD_3CN$).**

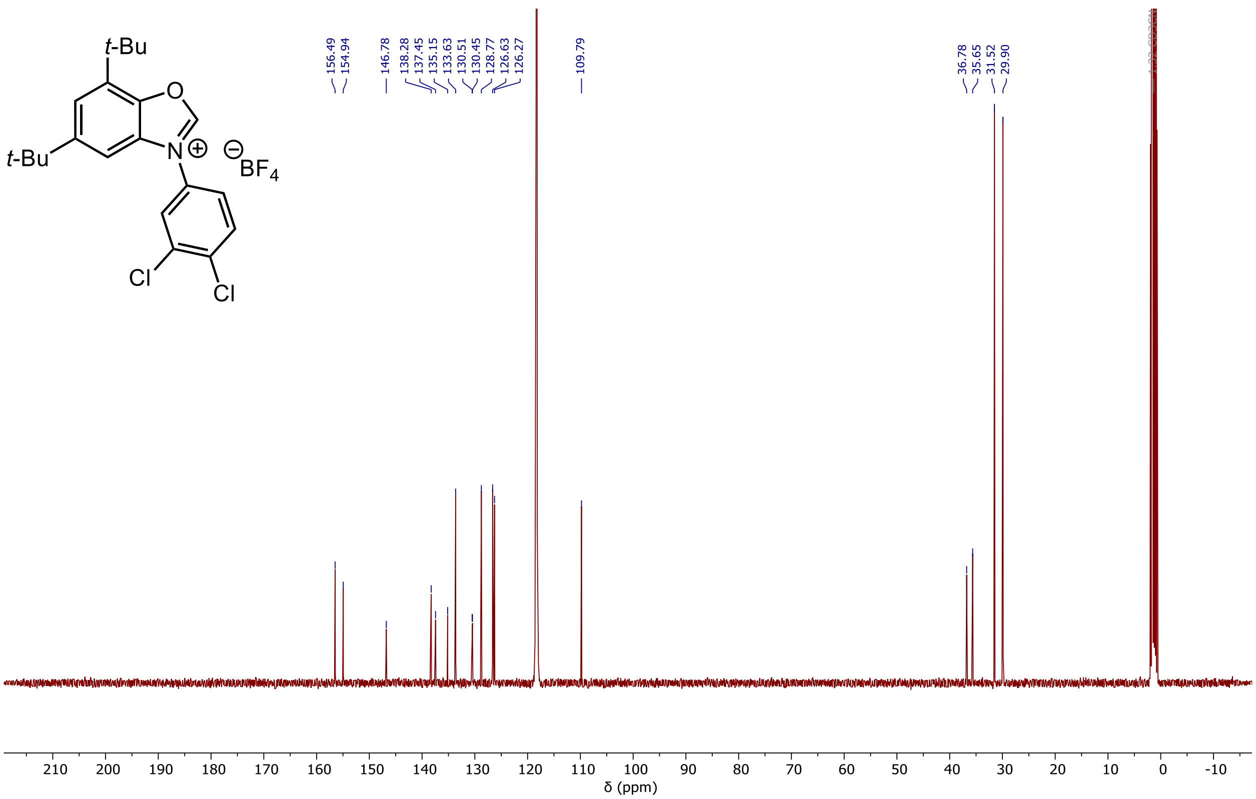

**Figure S37.** **$^{13}$C NMR spectra of 5,7-di-tert-butyl-3-(3,4-dichlorophenyl)benzo[d]oxazol-3-ium tetrafluoroborate (NHC-6) (101 MHz, $CD_3CN$).**

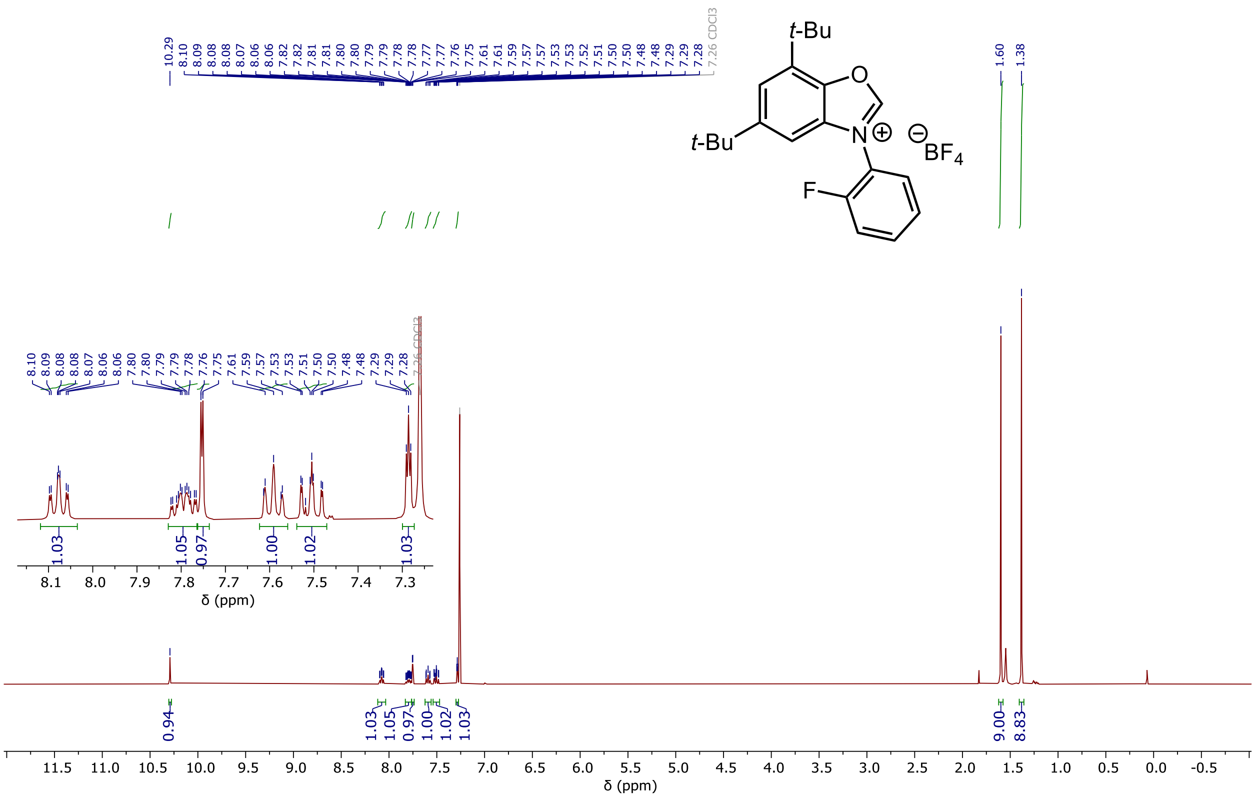

**Figure S38.** **$^{1}$H NMR spectra of 5,7-di-tert-butyl-3-(2-fluorophenyl)benzo[d]oxazol-3-ium tetrafluoroborate (NHC-7) (400 MHz, $CDCl_3$).**

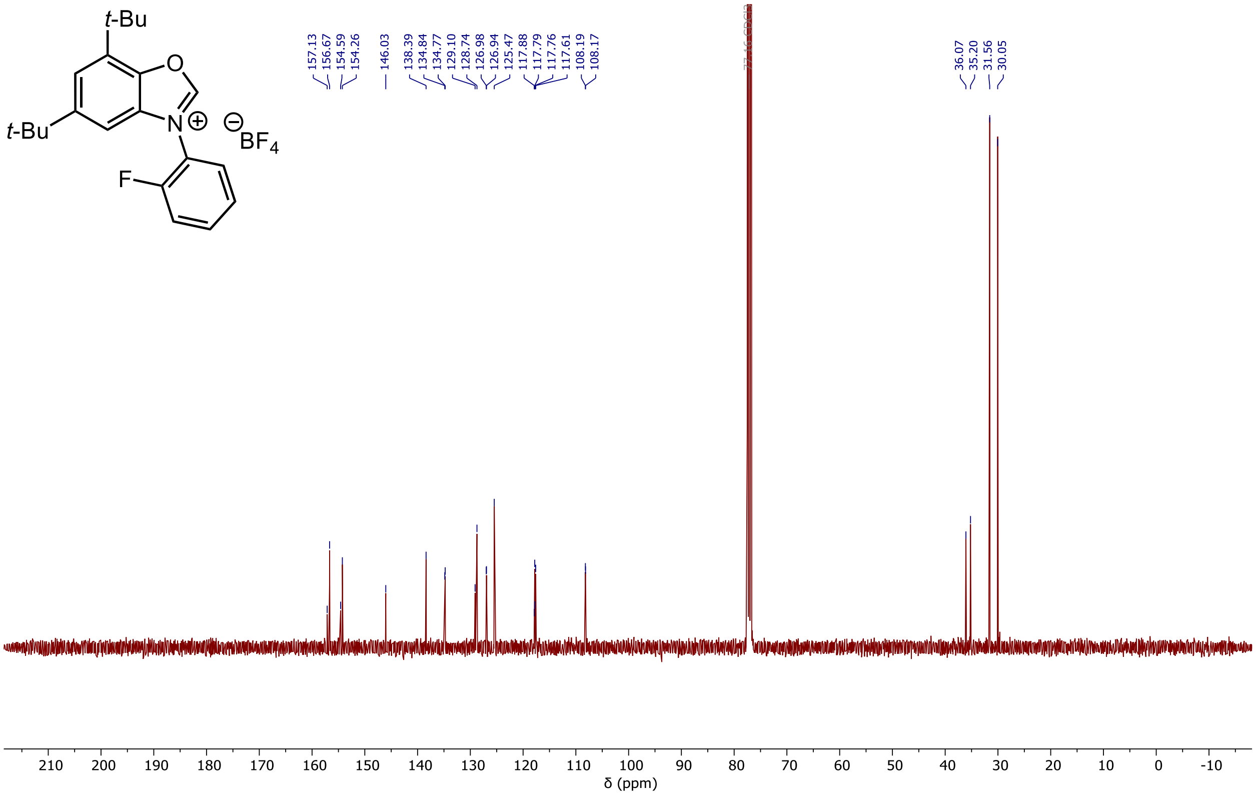

**Figure S39.** $^{13}$**C NMR spectra of 5,7-di-tert-butyl-3-(2-fluorophenyl)benzo[d]oxazol-3-ium tetrafluoroborate (NHC-7) (101 MHz, CDCl$_3$).**

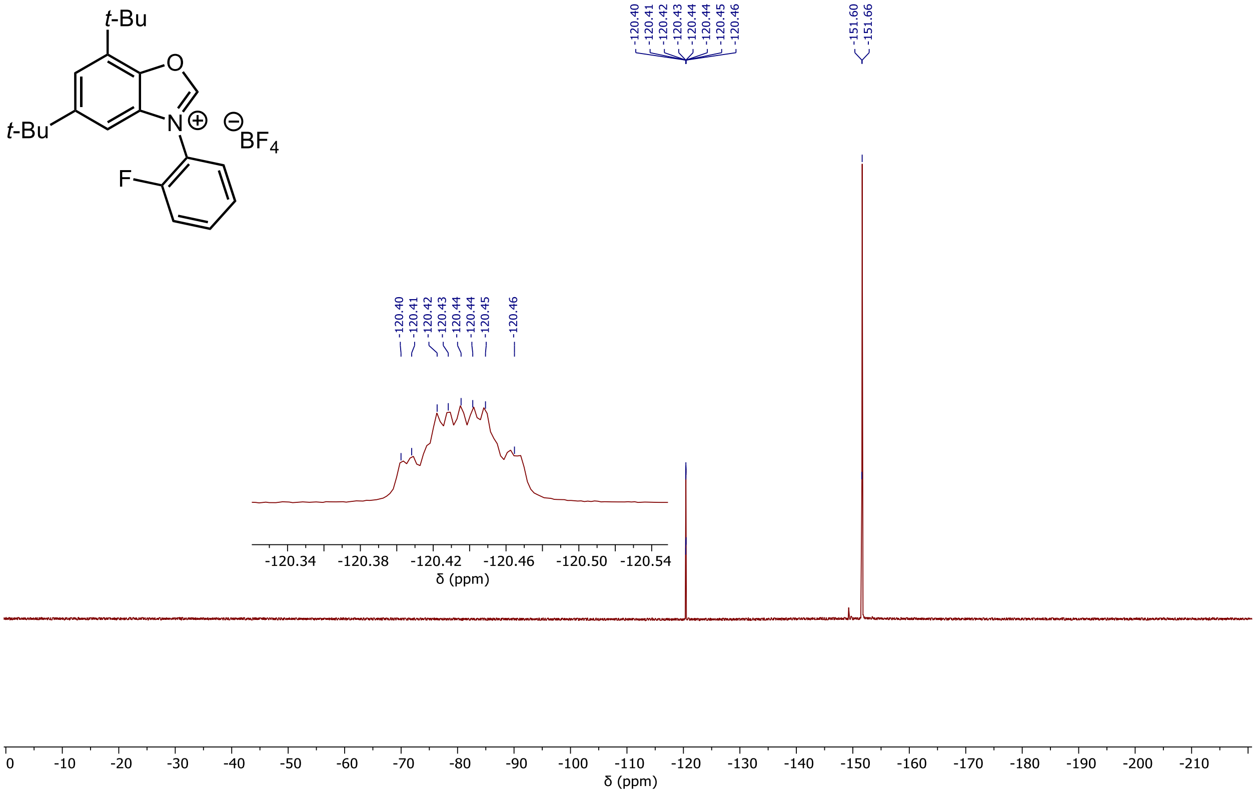

**Figure S40.** $^{19}$**F NMR spectra of 5,7-di-tert-butyl-3-(2-fluorophenyl)benzo[d]oxazol-3-ium tetrafluoroborate (NHC-7) (376 MHz, CDCl$_3$).**

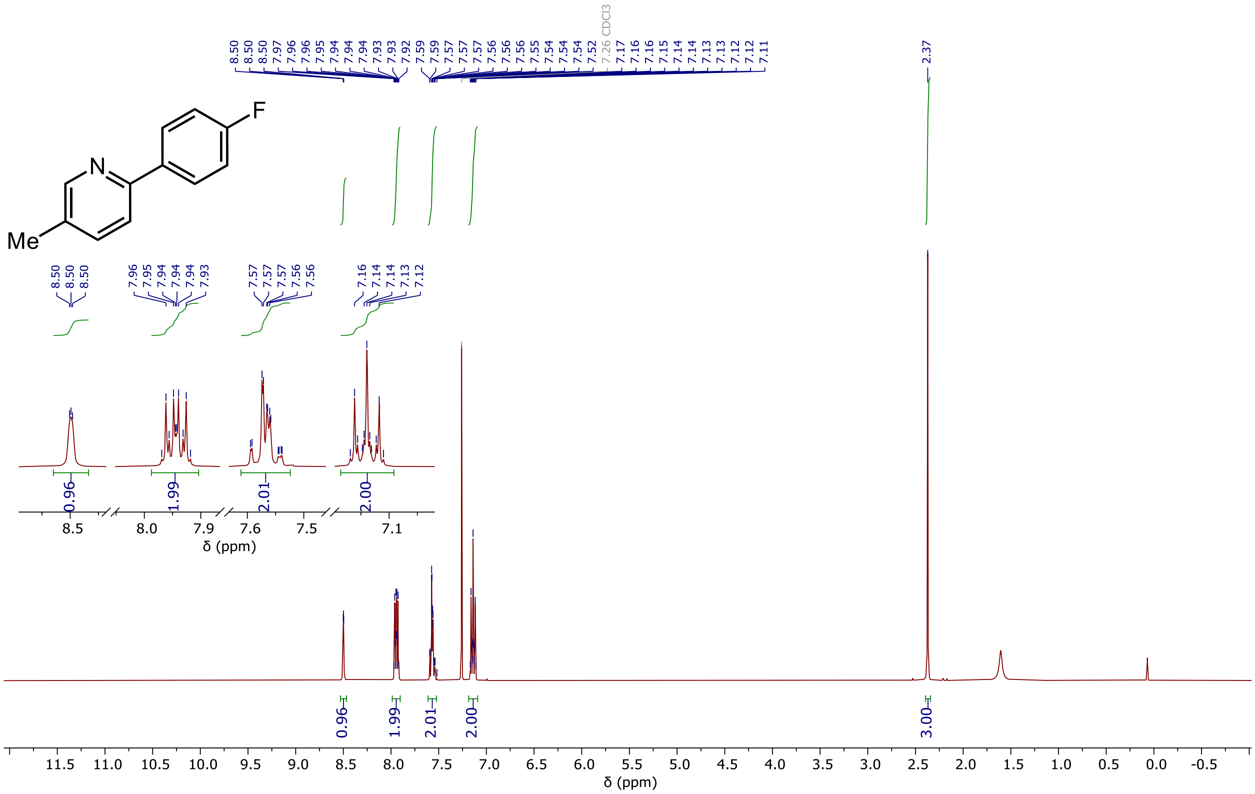

**Figure S41.** **$^{1}H$ NMR spectra of 2-(4-fluorophenyl)-5-methylpyridine (400 MHz, $CDCl_3$).**

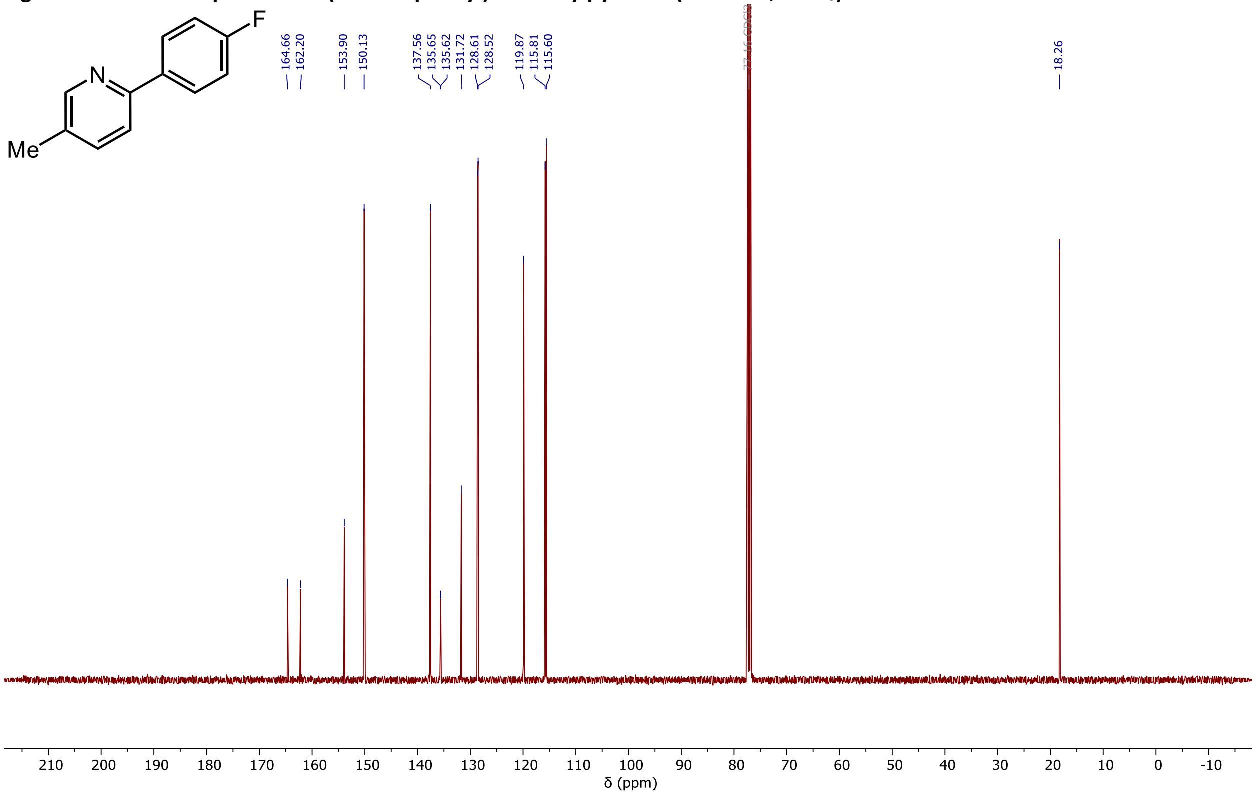

**Figure S42.** **$^{13}C$ NMR spectra of 2-(4-fluorophenyl)-5-methylpyridine (101 MHz, $CDCl_3$).**

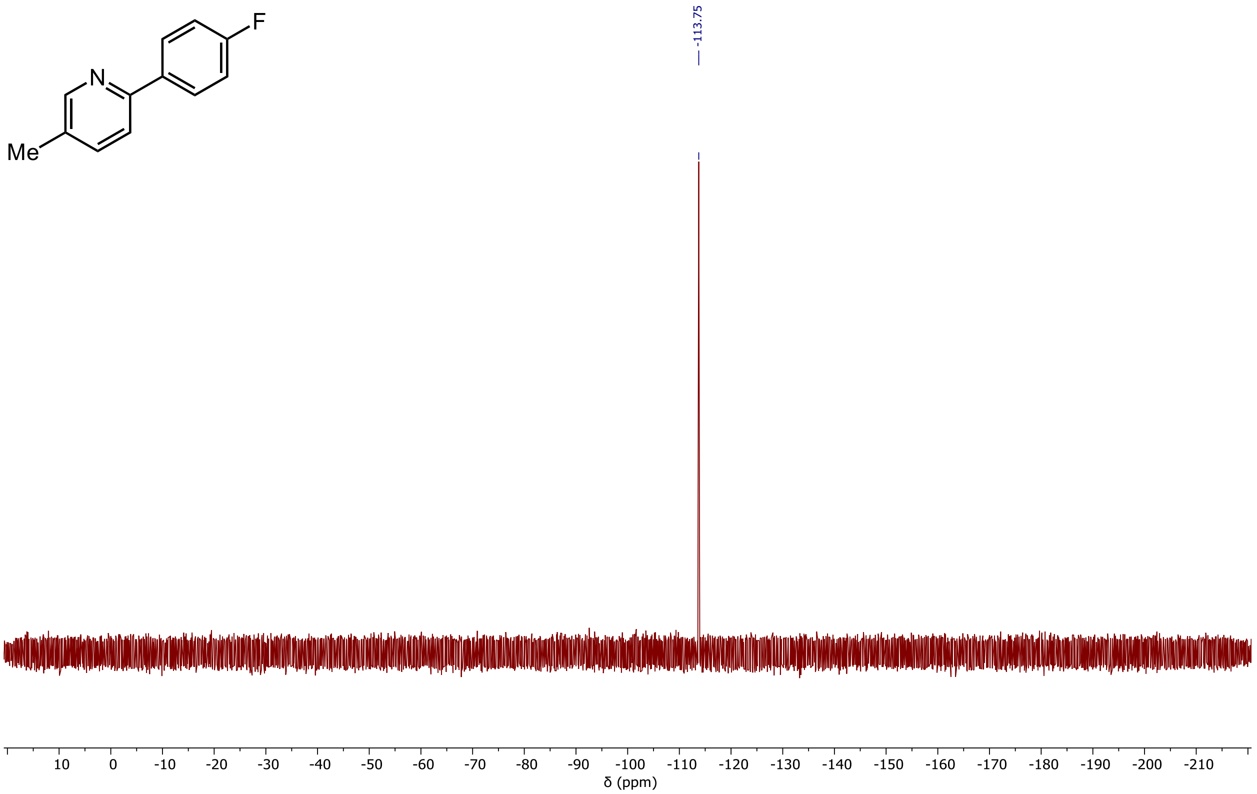

**Figure S43. $^{19}F$ NMR spectra of 2-(4-fluorophenyl)-5-methylpyridine (376 MHz, $CDCl_3$).**

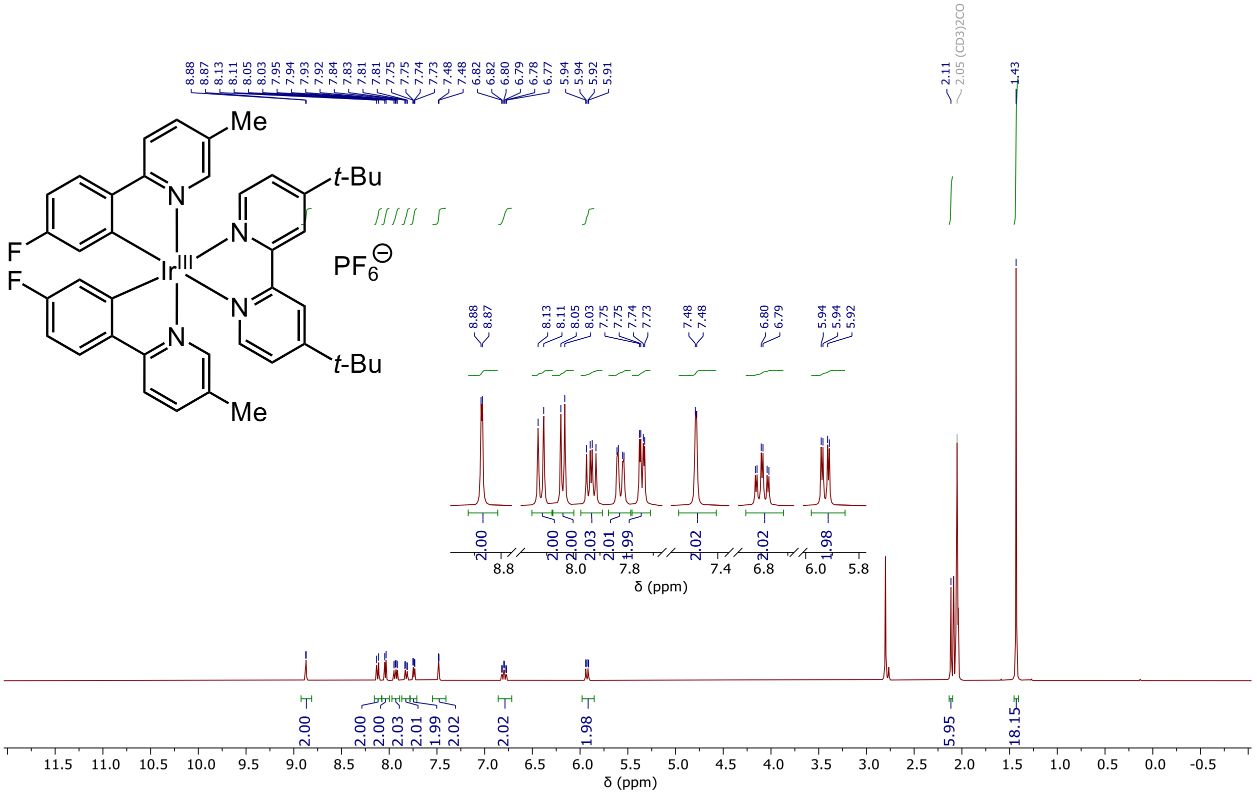

**Figure S44. $^{1}H$ NMR spectra of $Ir(F(Me)ppy)_2(dtbbpy)PF_6$ (400 MHz, Acetone-$d_6$).**

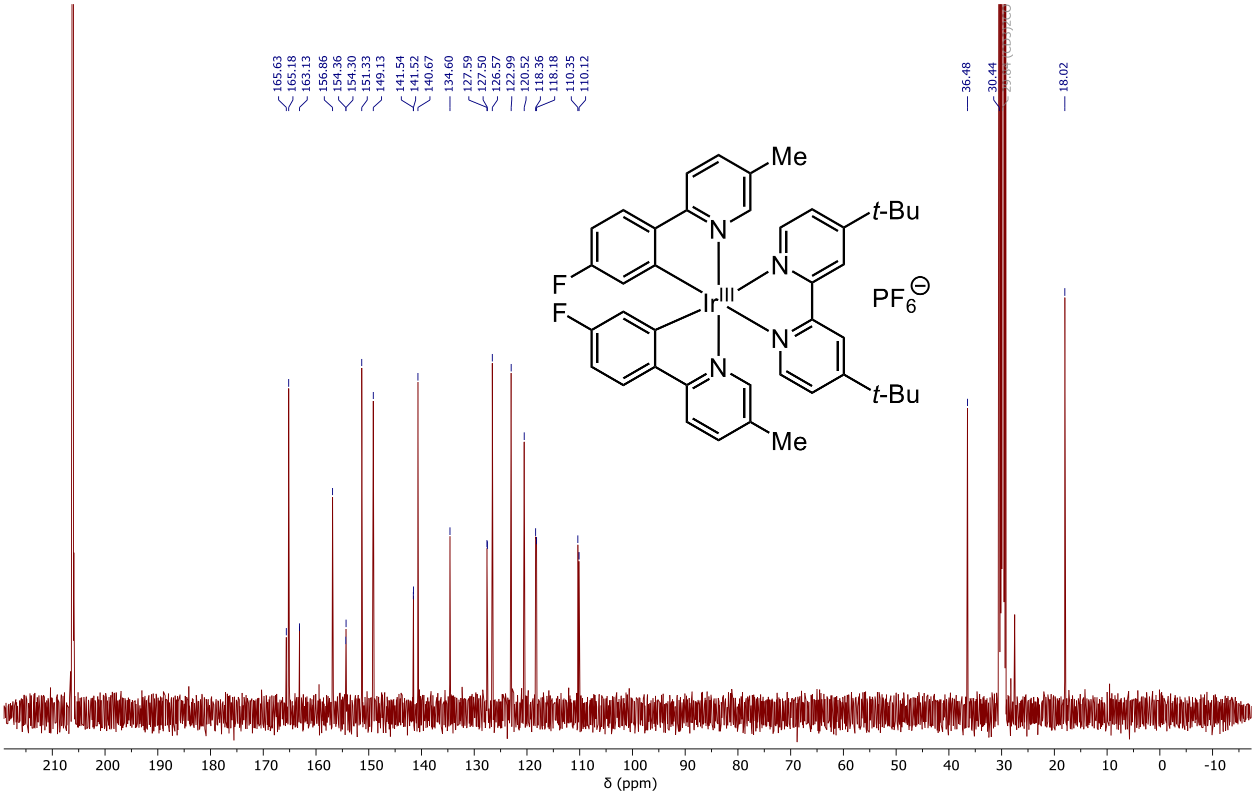


**Figure S45.** $^{13}C$ **NMR spectra of Ir(F(Me)ppy)$_2$(dtbbpy)PF$_6$ (101 MHz, Acetone-d$_6$).**

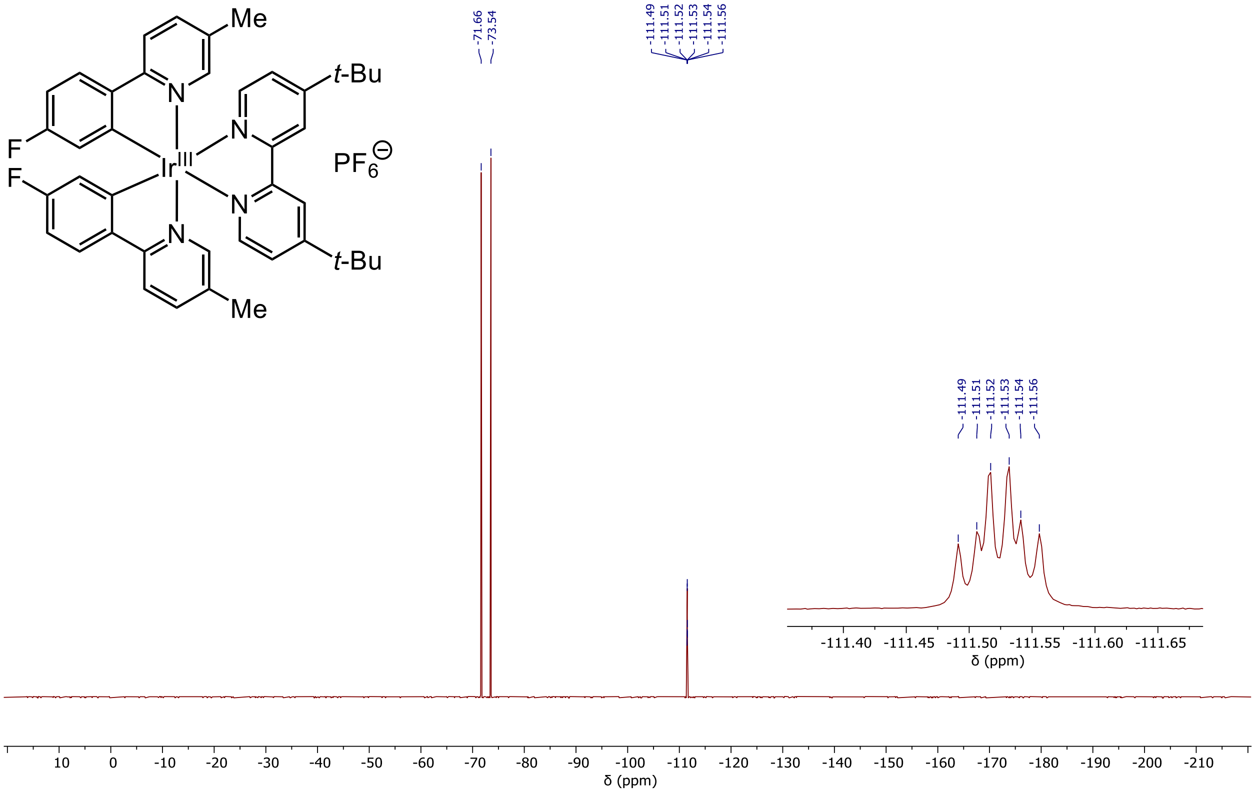


**Figure S46.** $^{19}F$ **NMR spectra of Ir(F(Me)ppy)$_2$(dtbbpy)PF$_6$ (376 MHz, Acetone-d$_6$).**

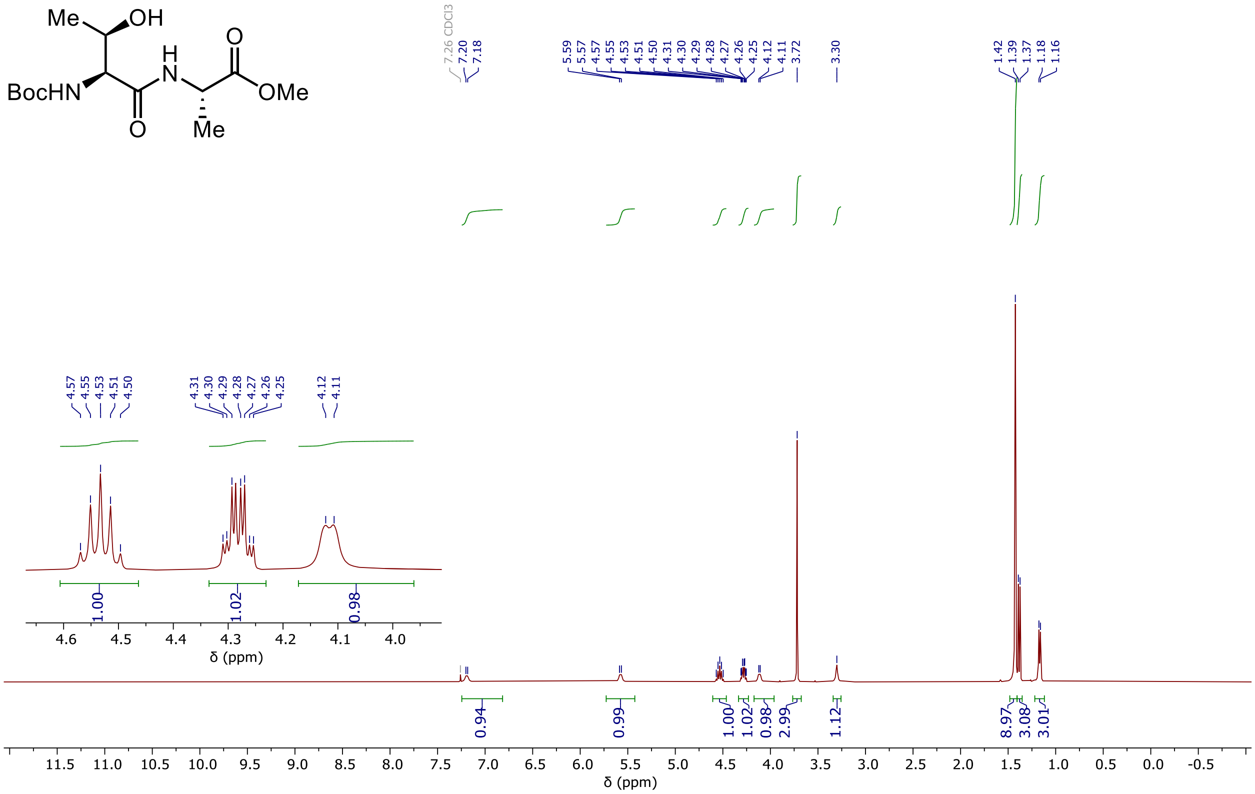

Figure S47. $^{1}H$ NMR spectra of Boc-Thr-Ala-OMe (400 MHz, $CDCl_3$).

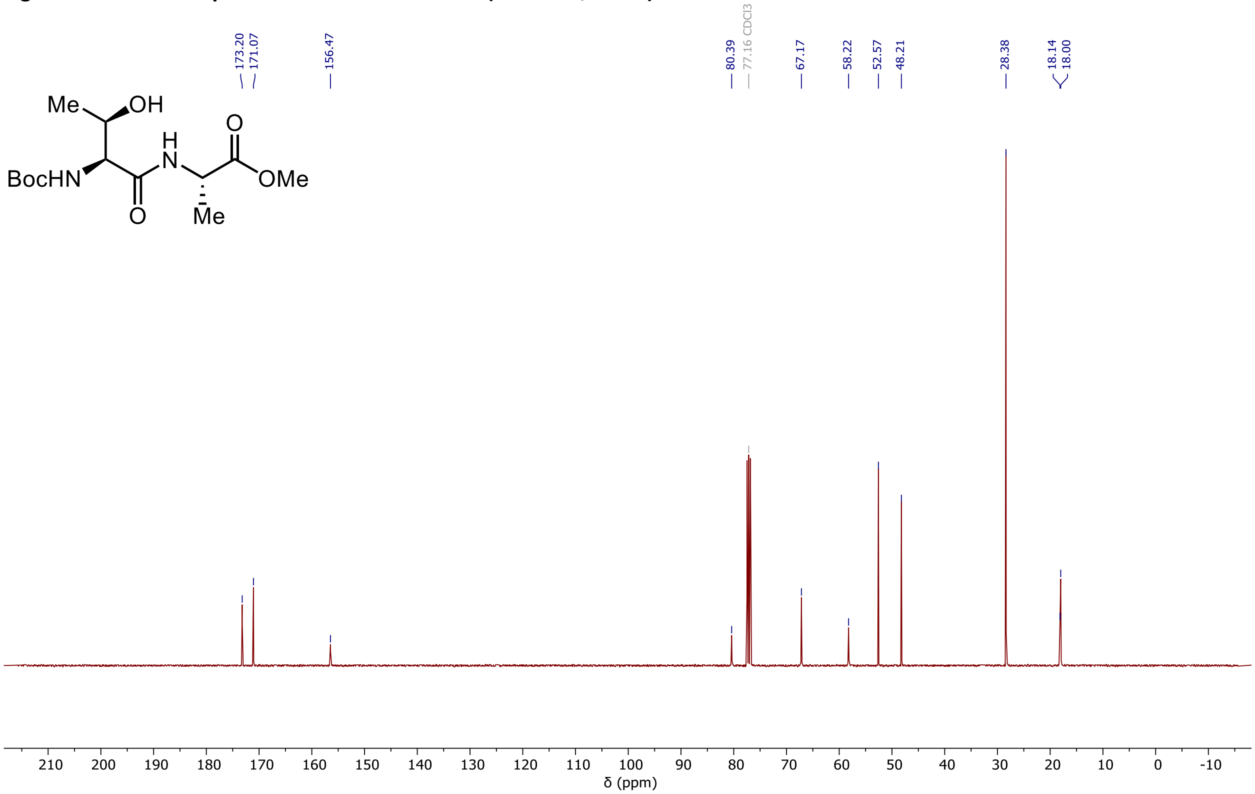

Figure S48. $^{13}C$ NMR spectra of Boc-Thr-Ala-OMe (101 MHz, $CDCl_3$).

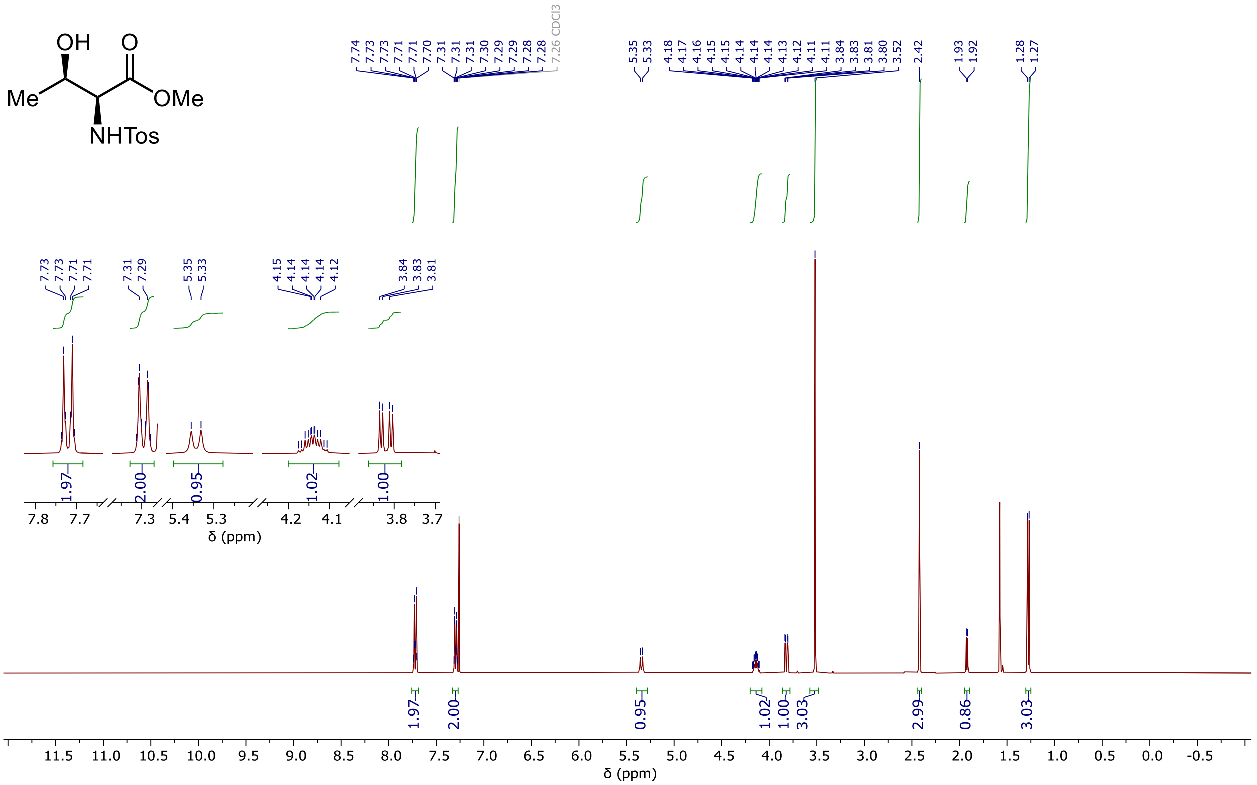


**Figure S49. $^{1}$H NMR spectra of Tos-Thr-OMe (400 MHz, $CDCl_3$).**

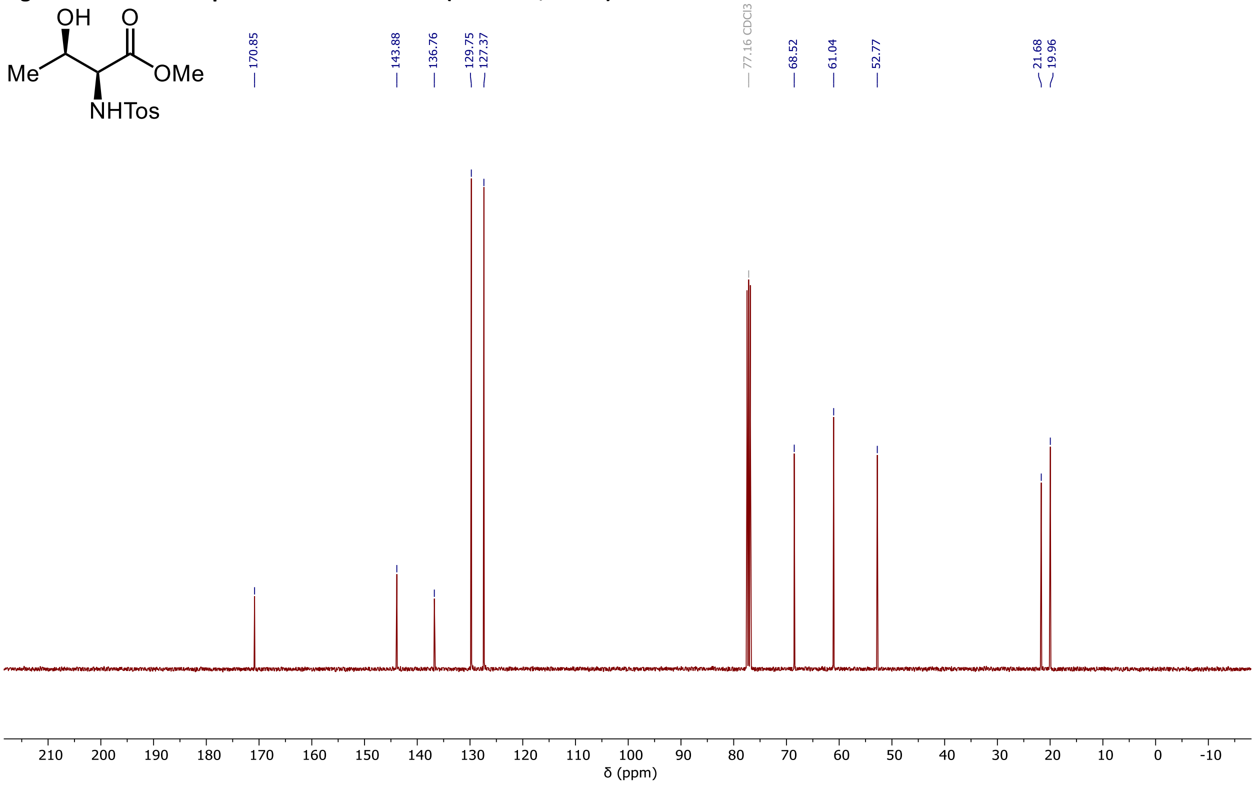


**Figure S50. $^{13}$C NMR spectra of Tos-Thr-OMe (101 MHz, $CDCl_3$).**

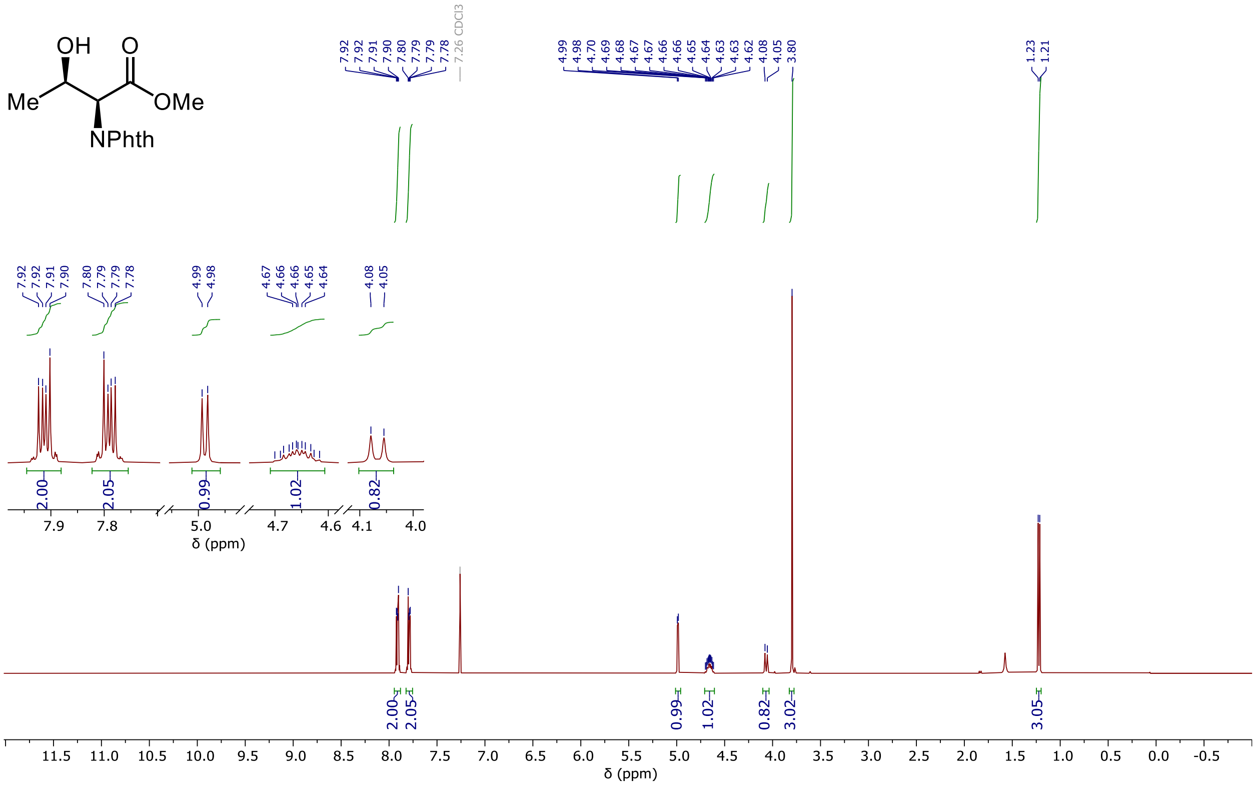


**Figure S51.** **$^{1}$H NMR spectra of Phth-Thr-OMe (400 MHz, $CDCl_3$).**

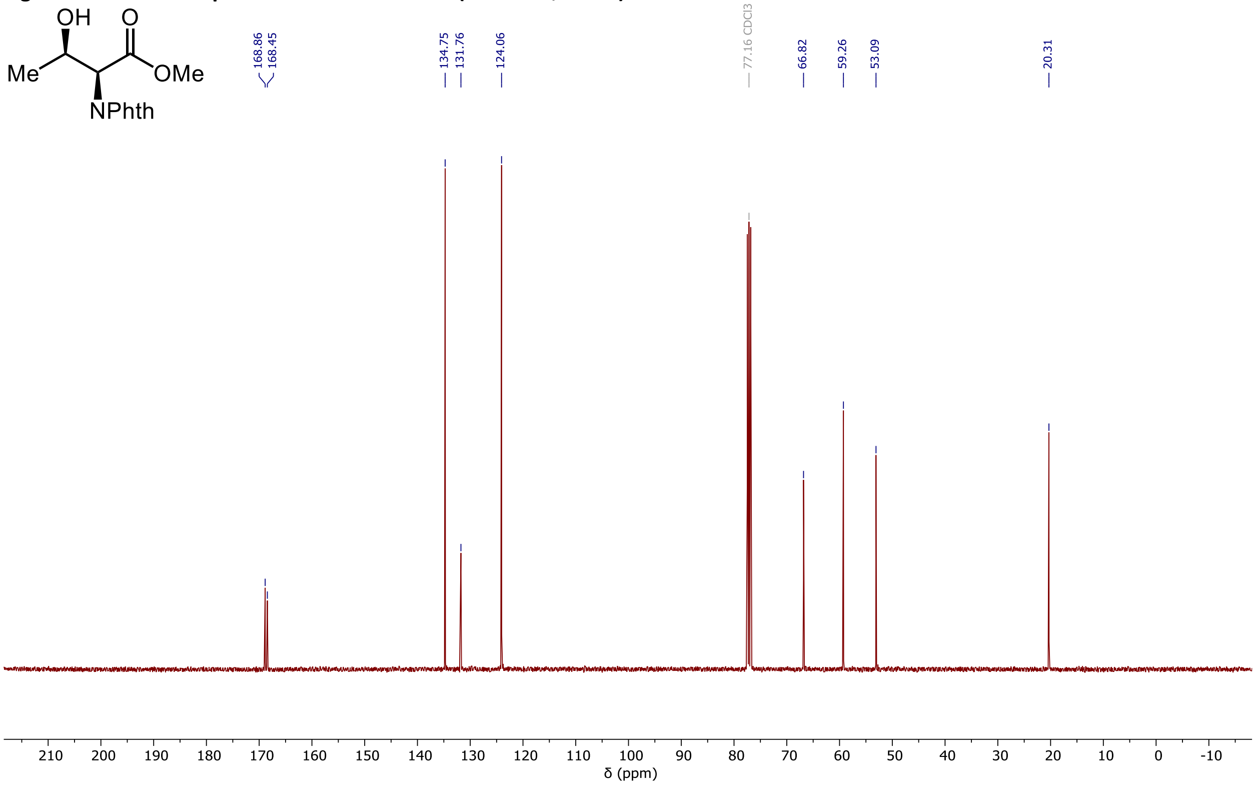


**Figure S52.** **$^{13}$C NMR spectra of Phth-Thr-OMe (101 MHz, $CDCl_3$).**

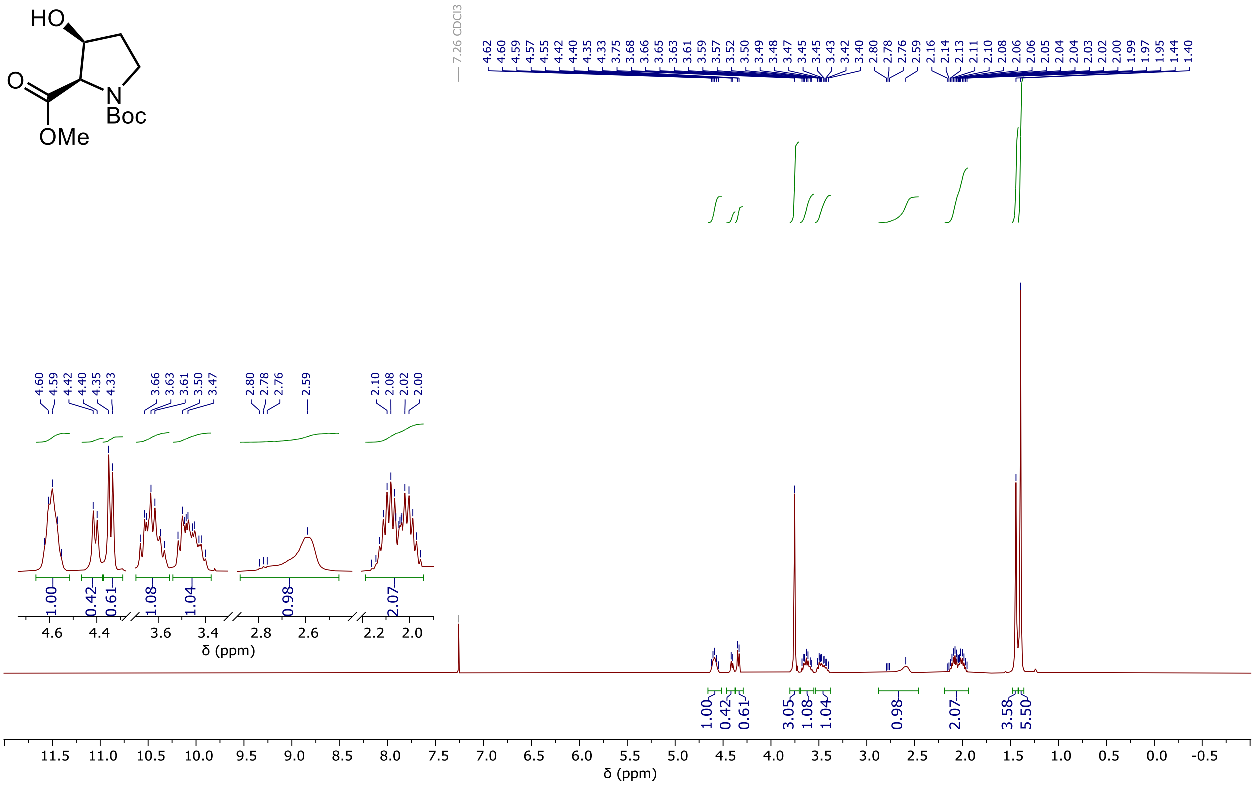

**Figure S53.** **$^{1}$H NMR spectra of Boc-3-Hyp-OMe (400 MHz, $CDCl_3$).**

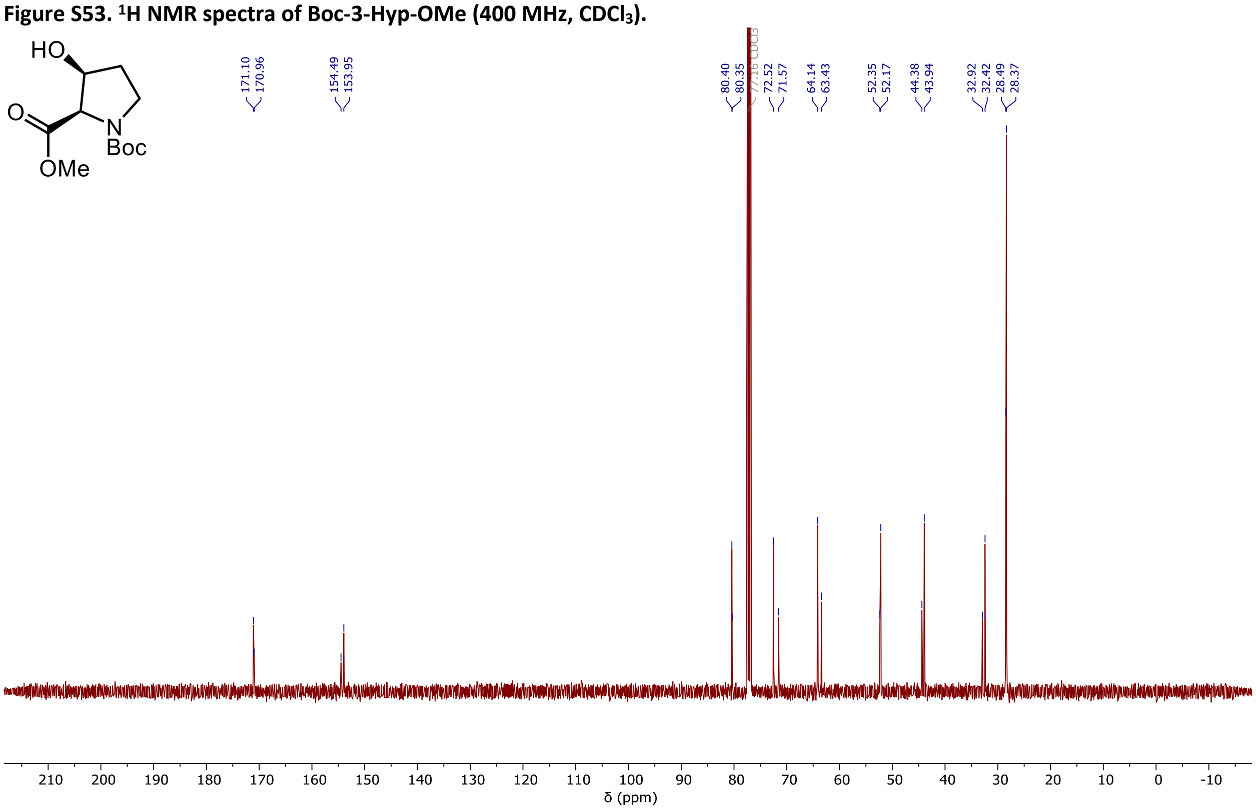

**Figure S54.** **$^{13}$C NMR spectra of Boc-3-Hyp-OMe (101 MHz, $CDCl_3$).**

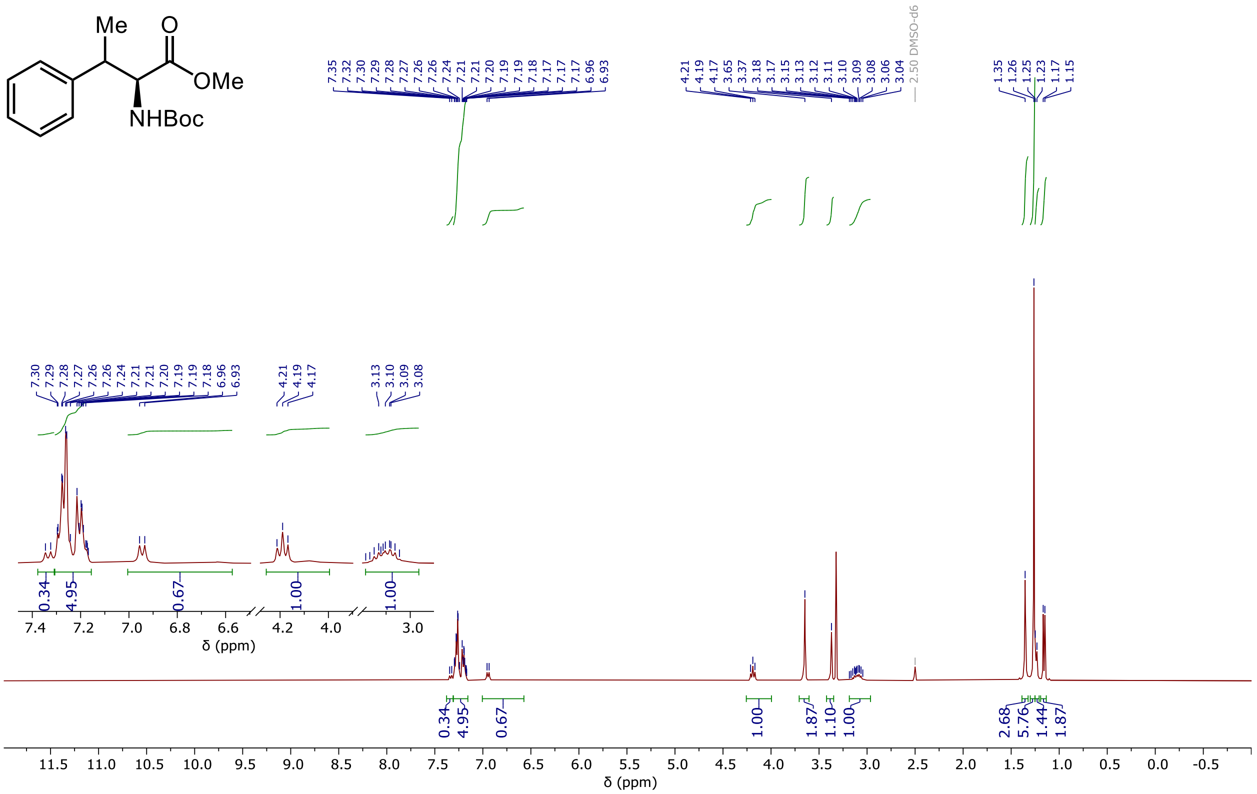

**Figure S55.** $^{1}$**H NMR spectra of (3) (400 MHz, DMSO-d$_6$).**

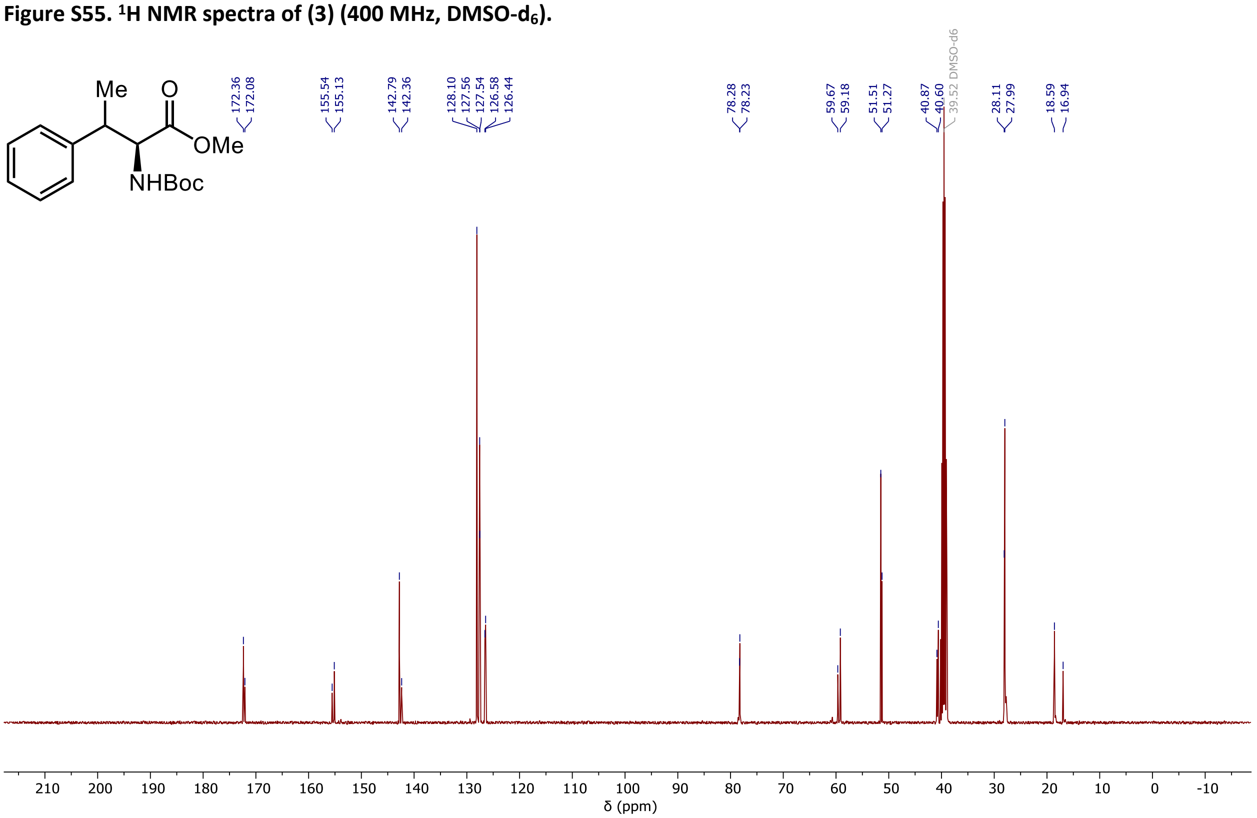

**Figure S56.** $^{13}$**C NMR spectra of (3) (101 MHz, DMSO-d$_6$).**

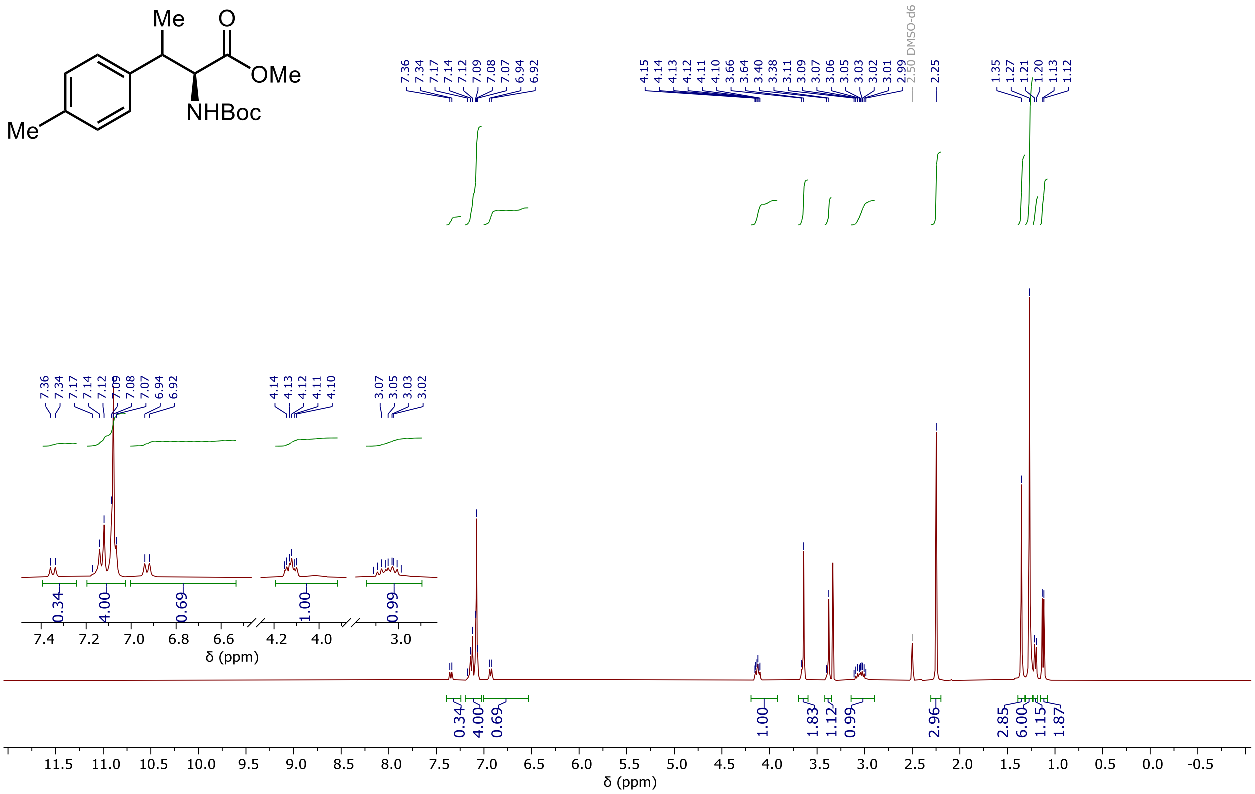

**Figure S57.** $^{1}H$ **NMR spectra of (4) (400 MHz, DMSO-d$_6$).**

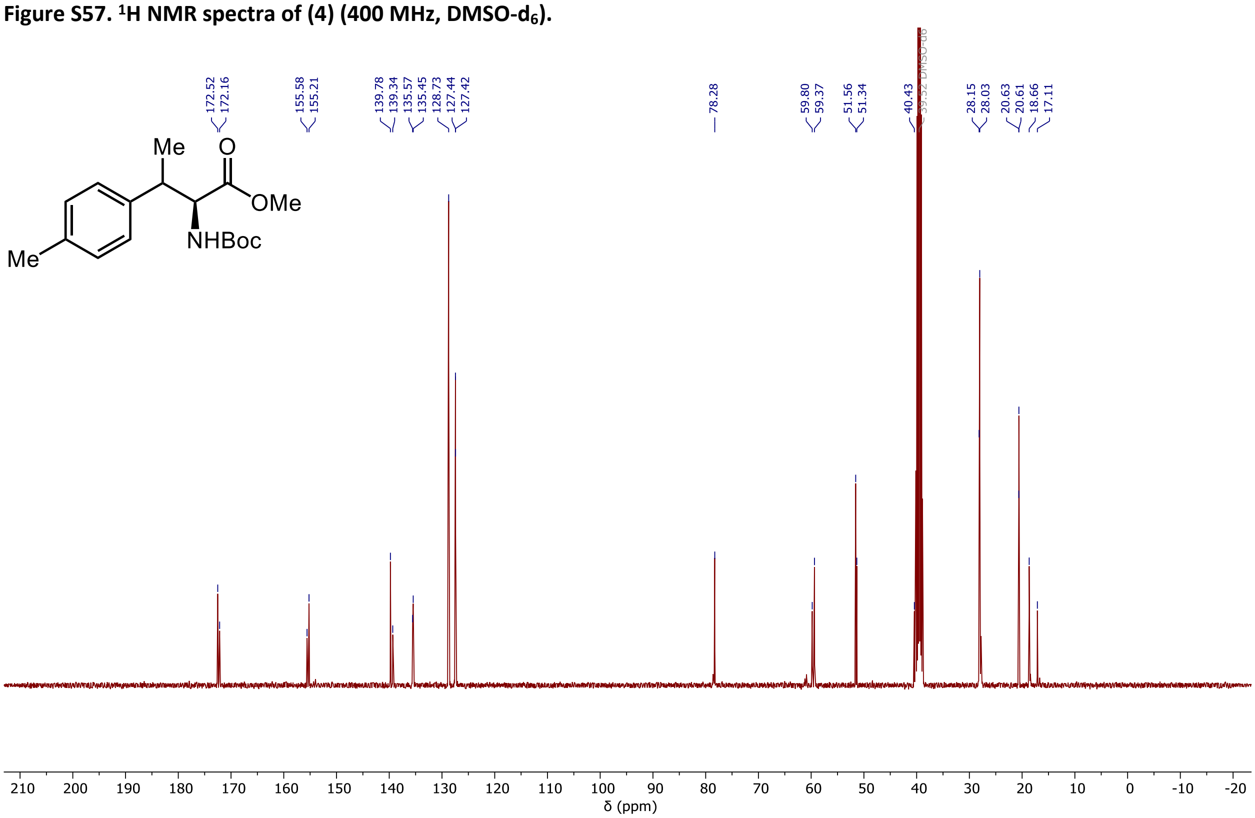

**Figure S58.** $^{13}C$ **NMR spectra of (4) (101 MHz, DMSO-d$_6$).**

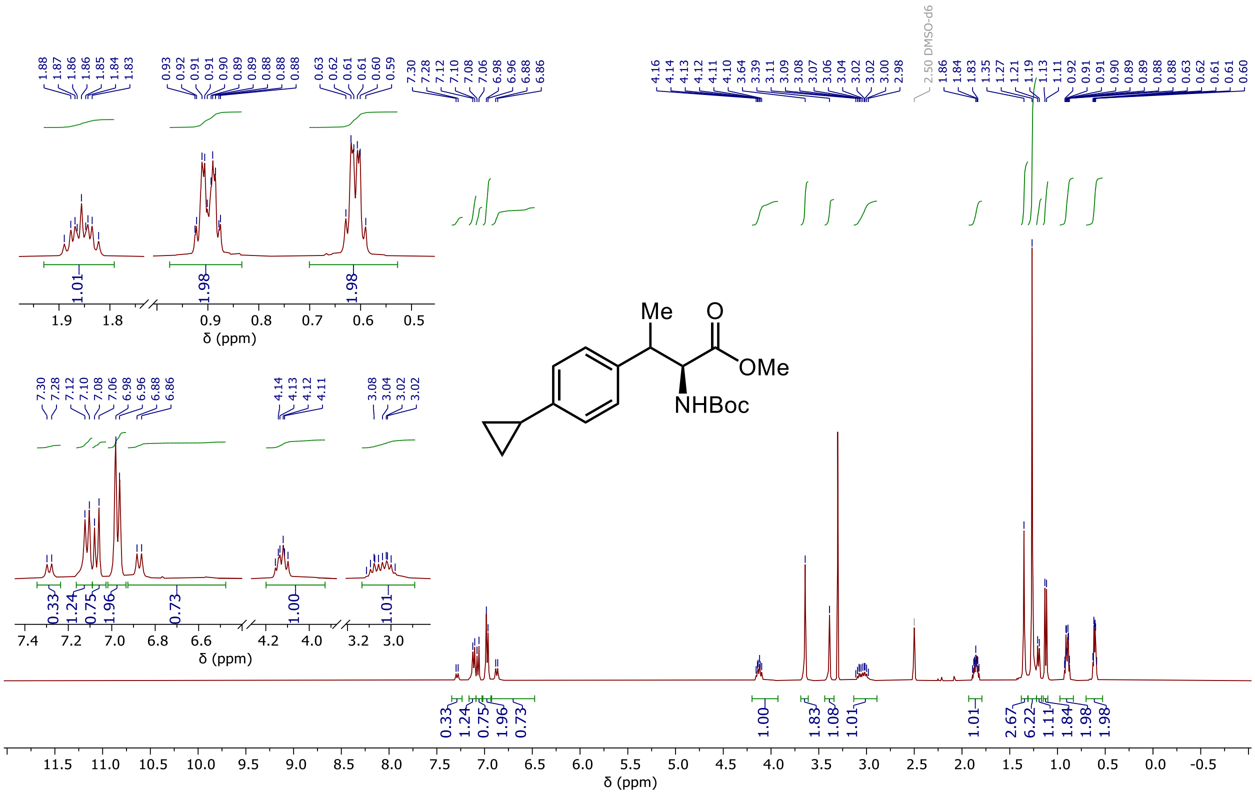

**Figure S59.** **$^{1}H$ NMR spectra of (5) (400 MHz, DMSO-$d_6$).**

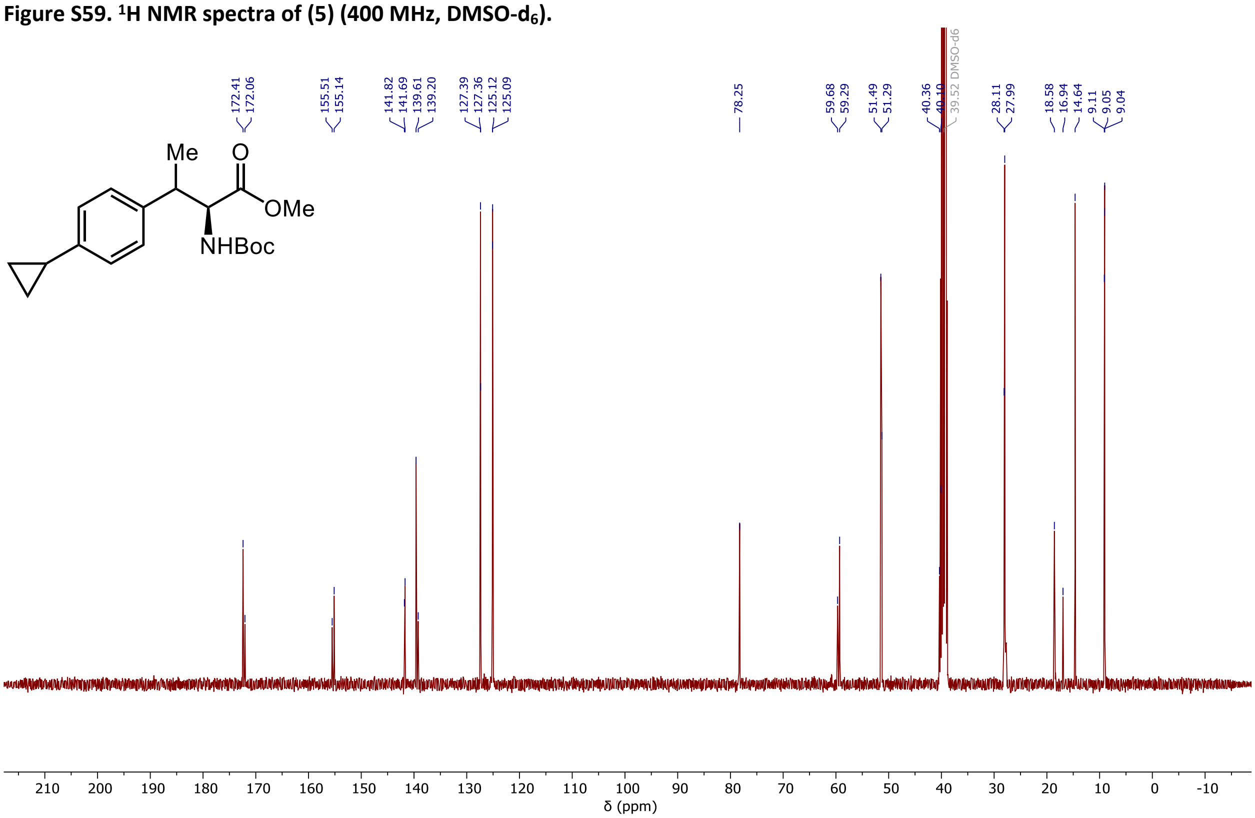

**Figure S60.** **$^{13}C$ NMR spectra of (5) (101 MHz, DMSO-$d_6$).**

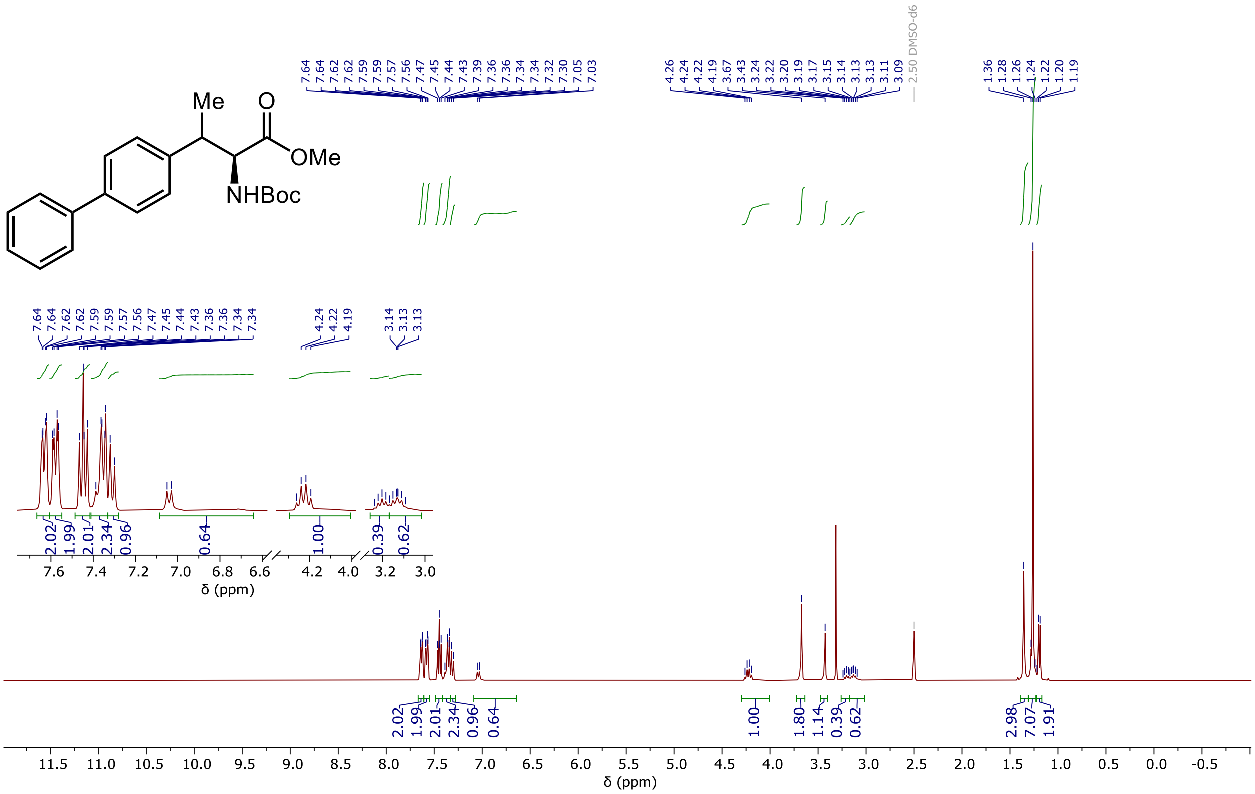

**Figure S61.** **$^{1}$H NMR spectra of (6) (400 MHz, DMSO-$d_6$).**

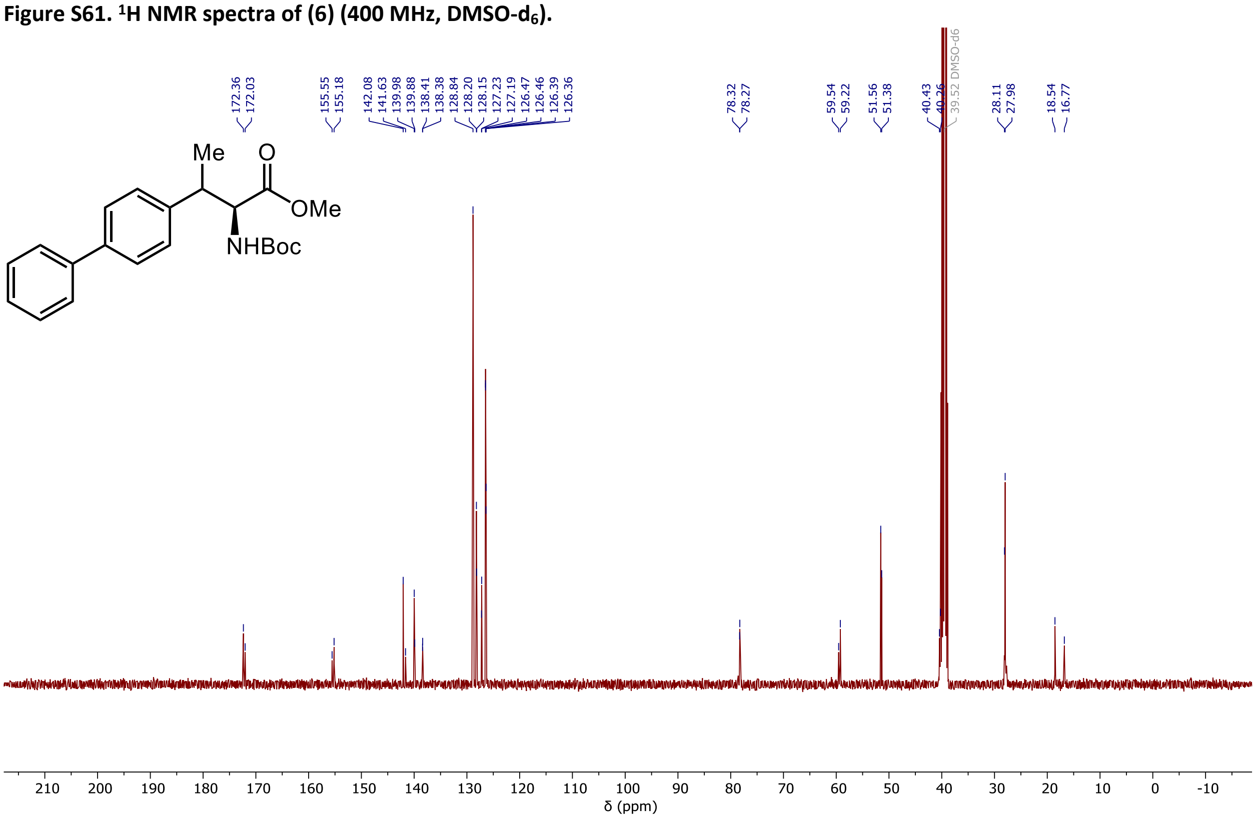

**Figure S62.** **$^{13}$C NMR spectra of (6) (101 MHz, DMSO-$d_6$).**

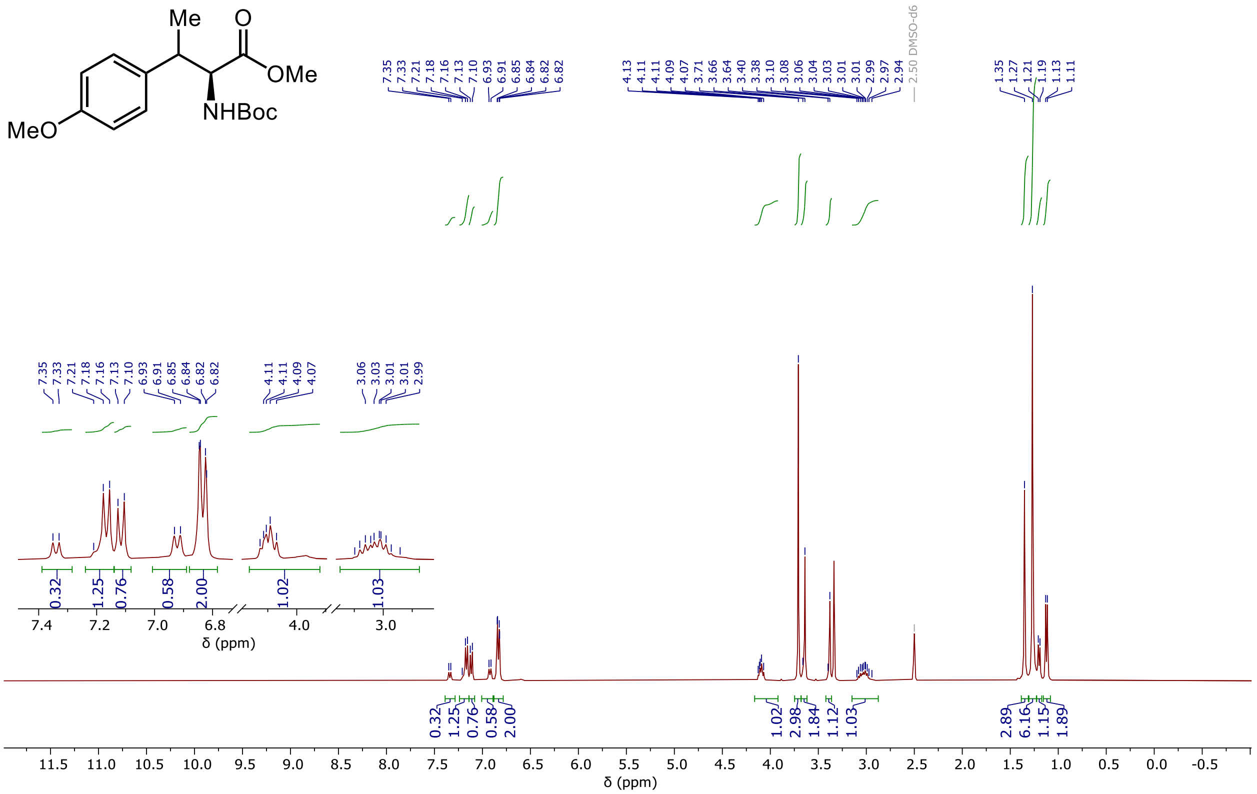

**Figure S63. $^{1}$H NMR spectra of (7) (400 MHz, DMSO-d$_6$).**

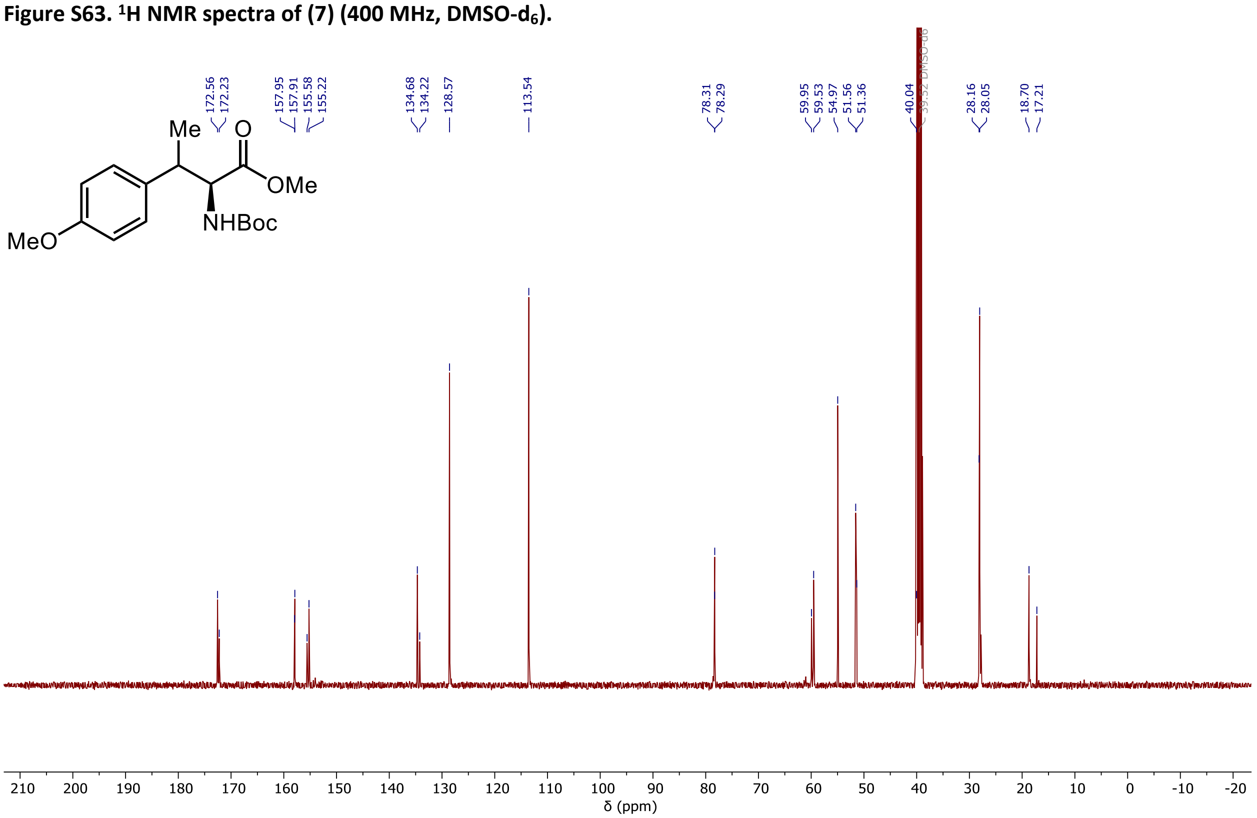

**Figure S64. $^{13}$C NMR spectra of (7) (101 MHz, DMSO-d$_6$).**

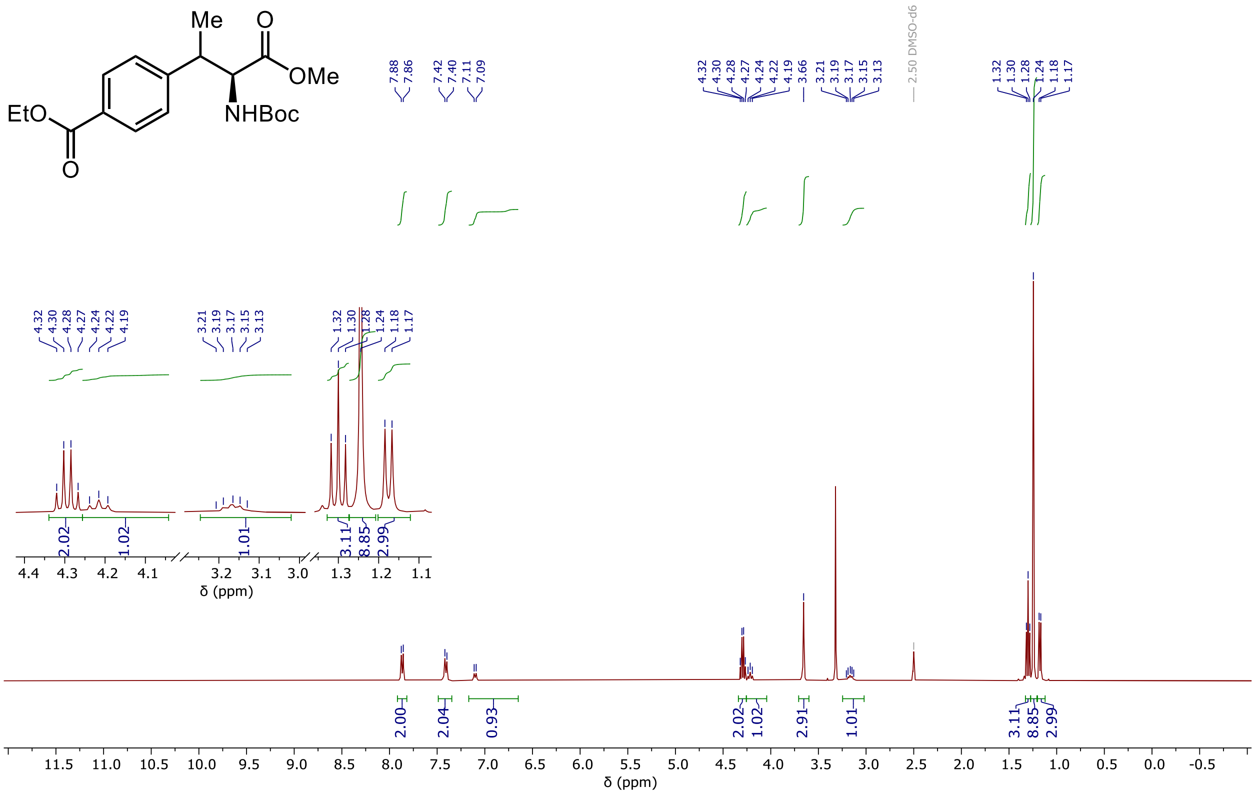

**Figure S65. $^{1}$H NMR spectra of (8a) (400 MHz, DMSO-$d_6$).**

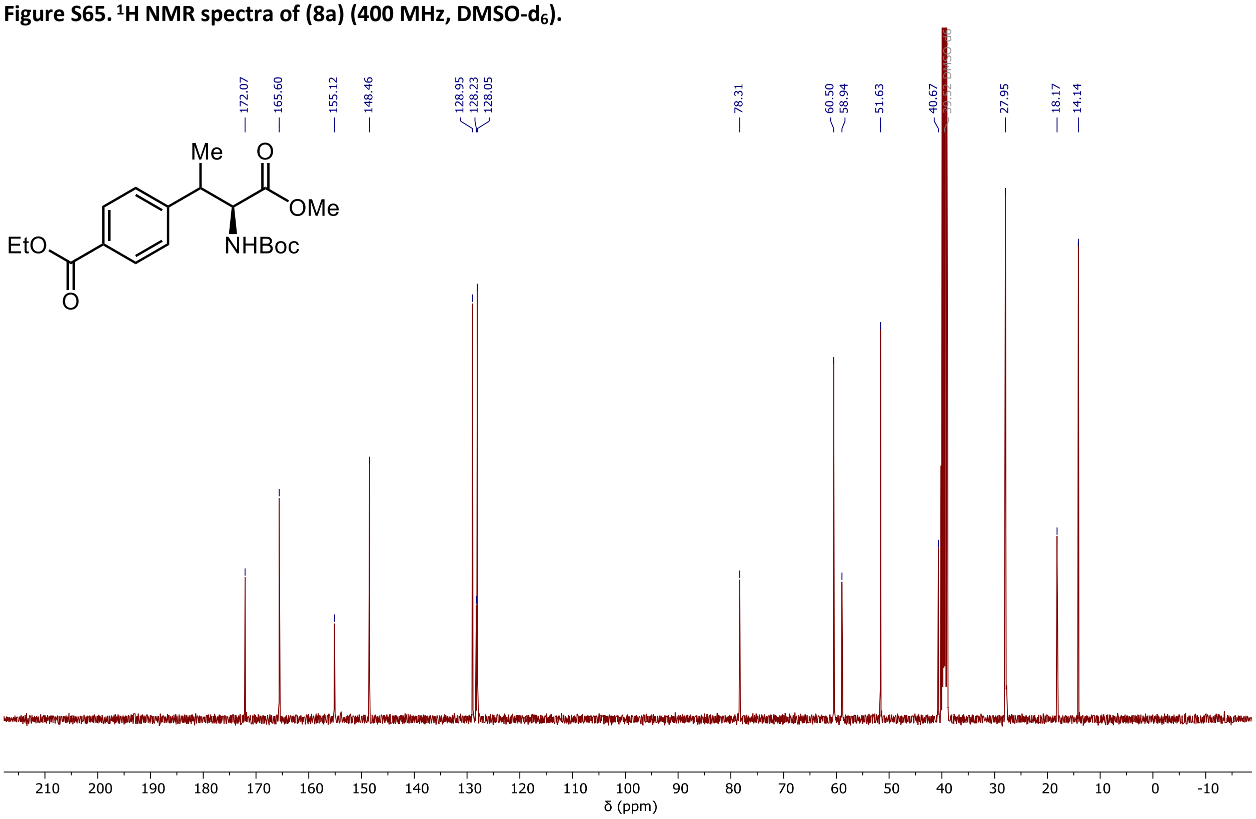

**Figure S66. $^{13}$C NMR spectra of (8a) (101 MHz, DMSO-$d_6$).**

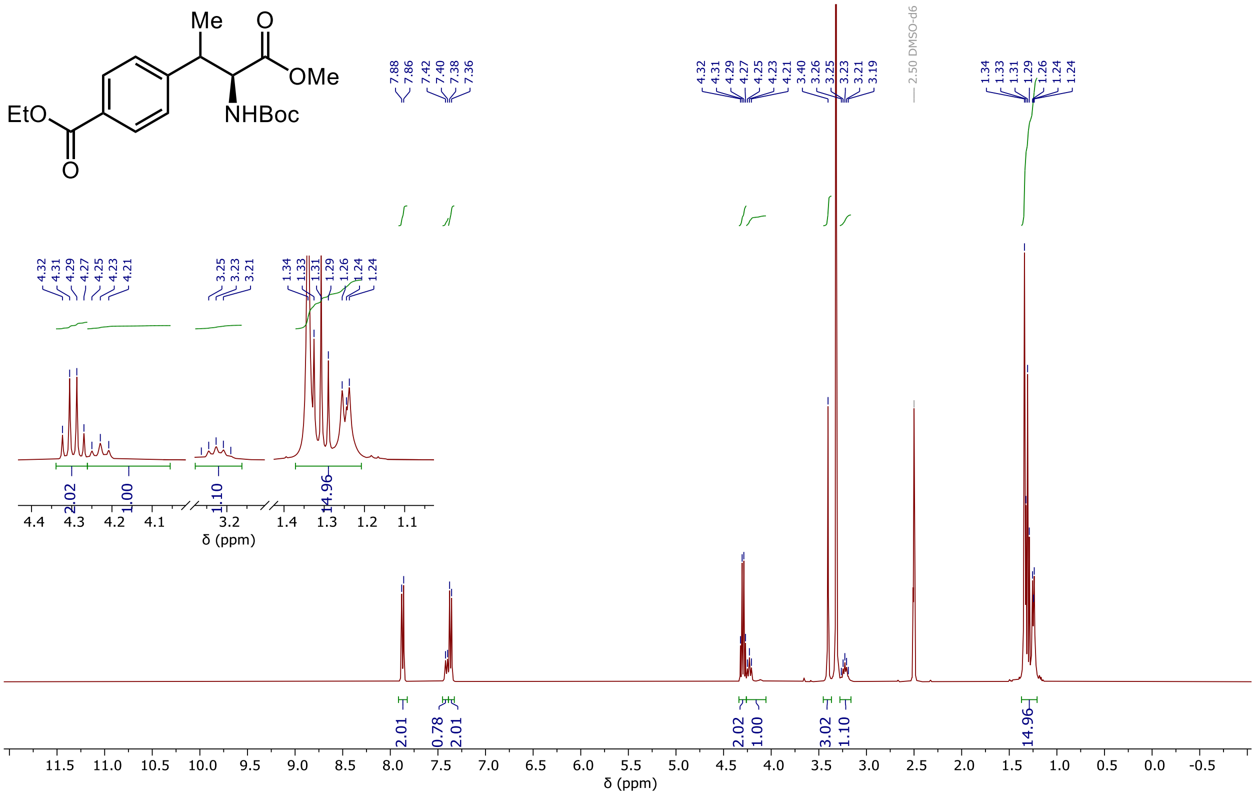


**Figure S67.** $^{1}H$ **NMR spectra of (8b) (400 MHz, DMSO-** $d_6$**).**

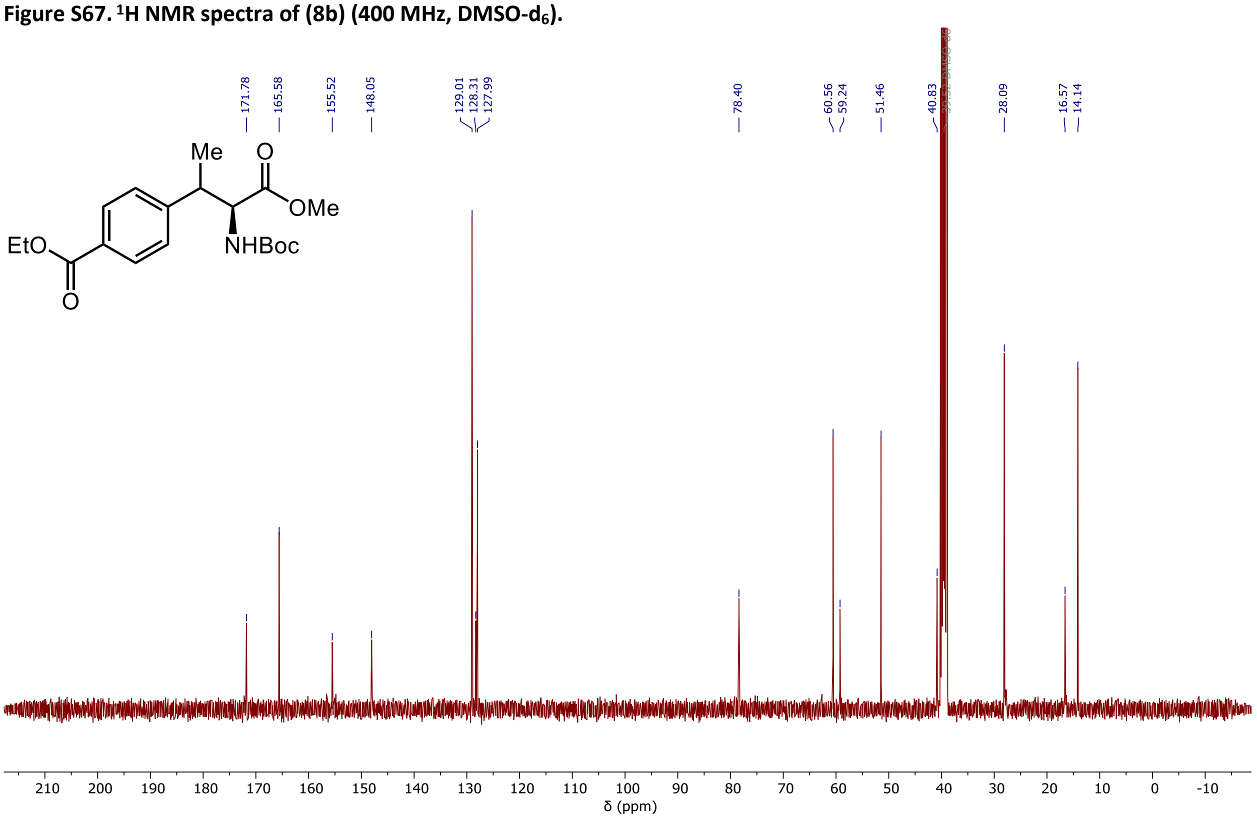


**Figure S68.** $^{13}C$ **NMR spectra of (8b) (101 MHz, DMSO-** $d_6$**).**

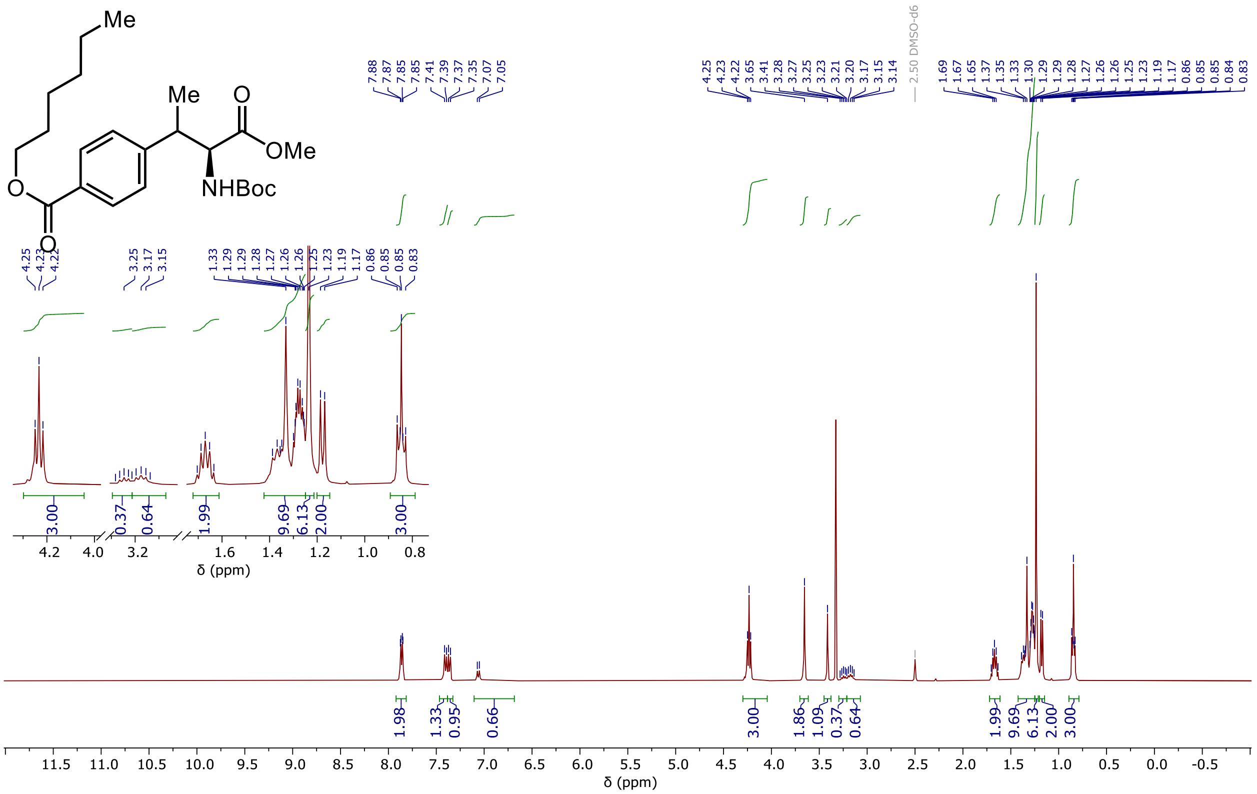


**Figure S69.** $^{1}$**H NMR spectra of (9) (400 MHz, DMSO-d$_6$).**

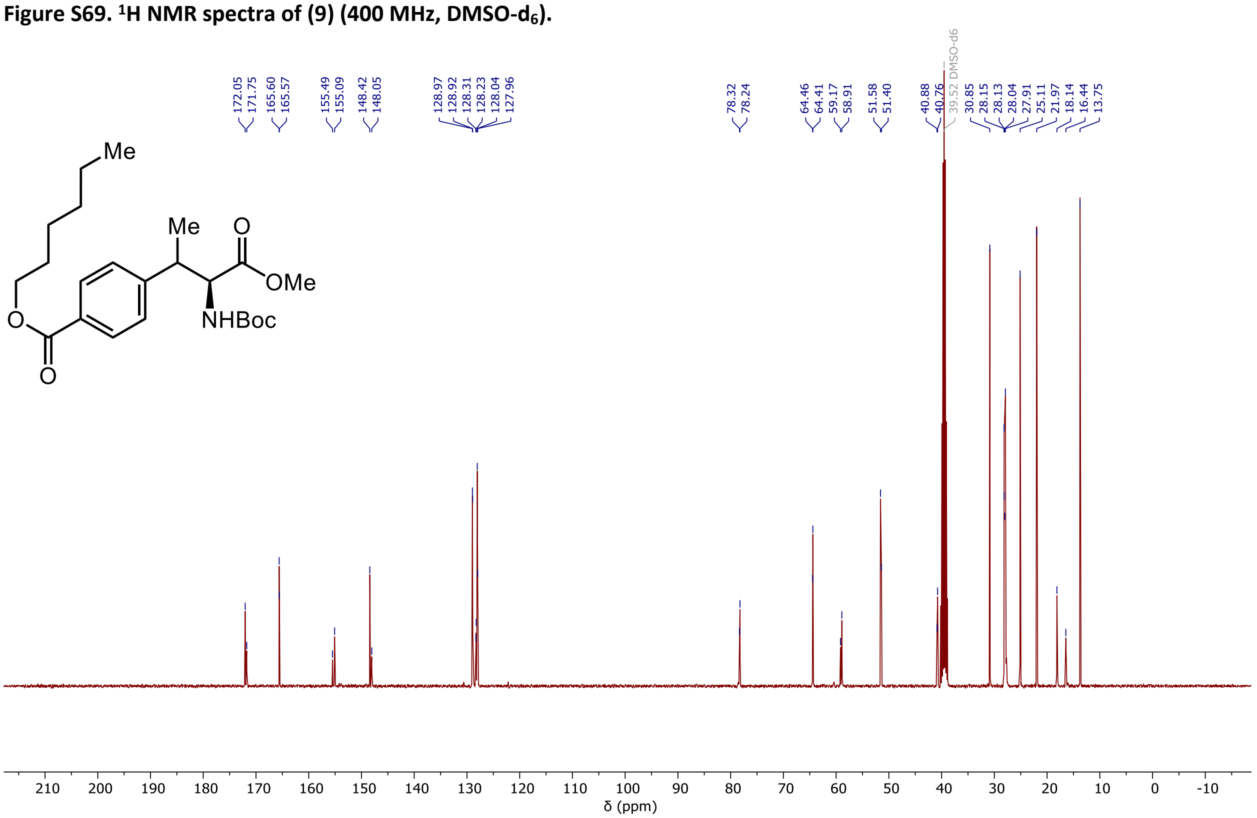


**Figure S70.** $^{13}$**C NMR spectra of (9) (101 MHz, DMSO-d$_6$).**

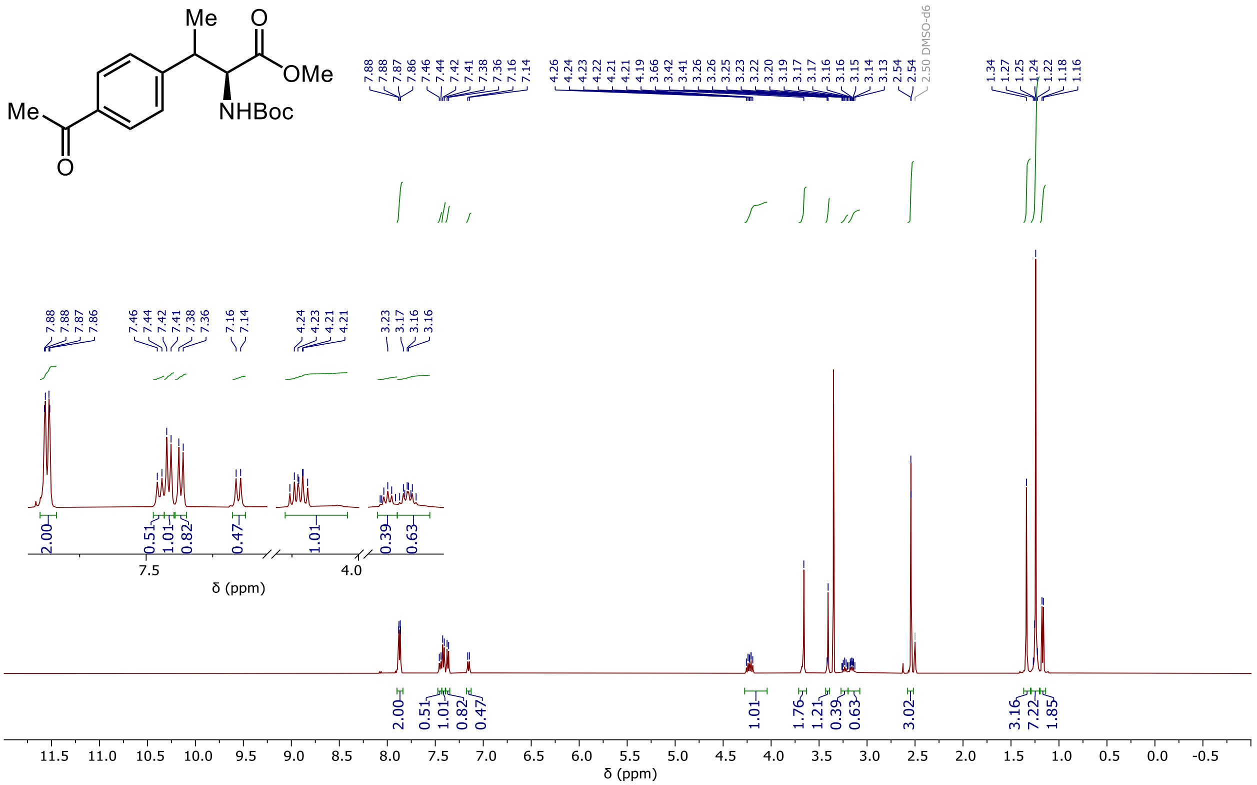


**Figure S71. $^{1}H$ NMR spectra of (10) (500 MHz, DMSO-$d_6$).**

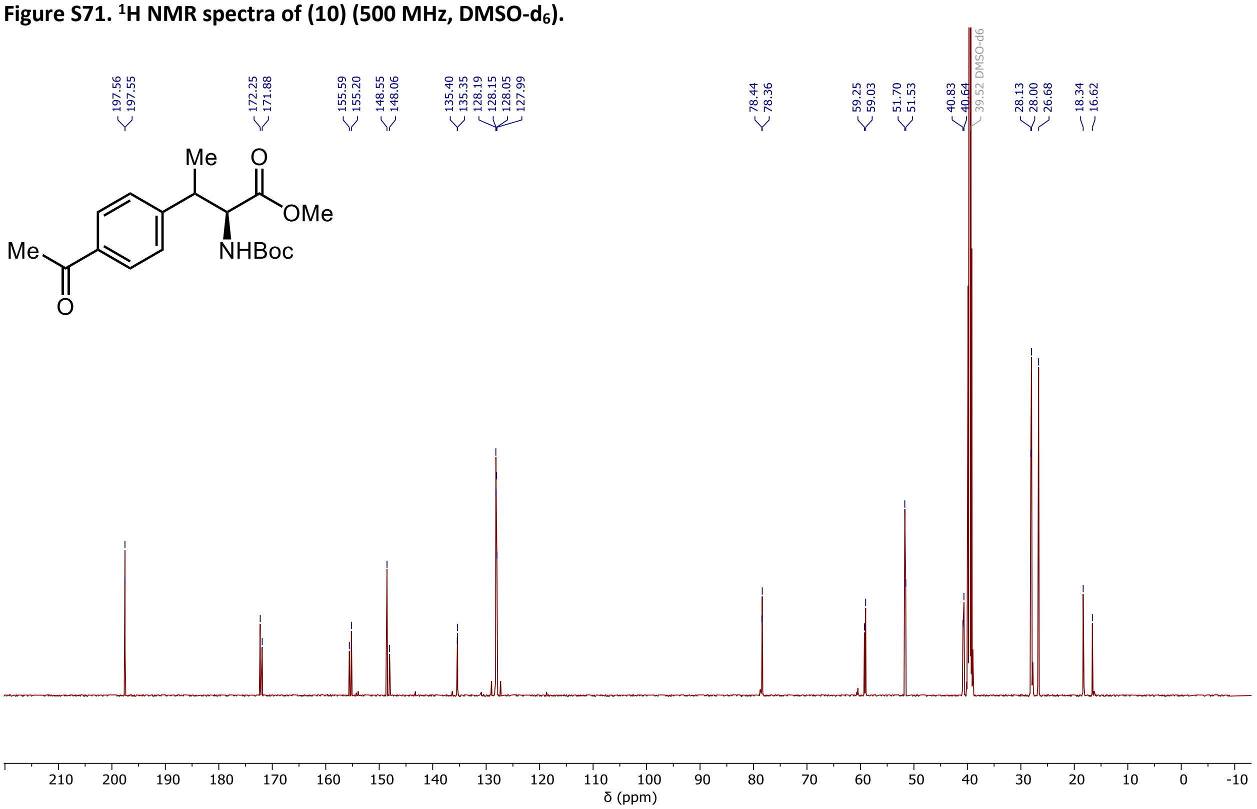


**Figure S72. $^{13}C$ NMR spectra of (10) (126 MHz, DMSO-$d_6$).**

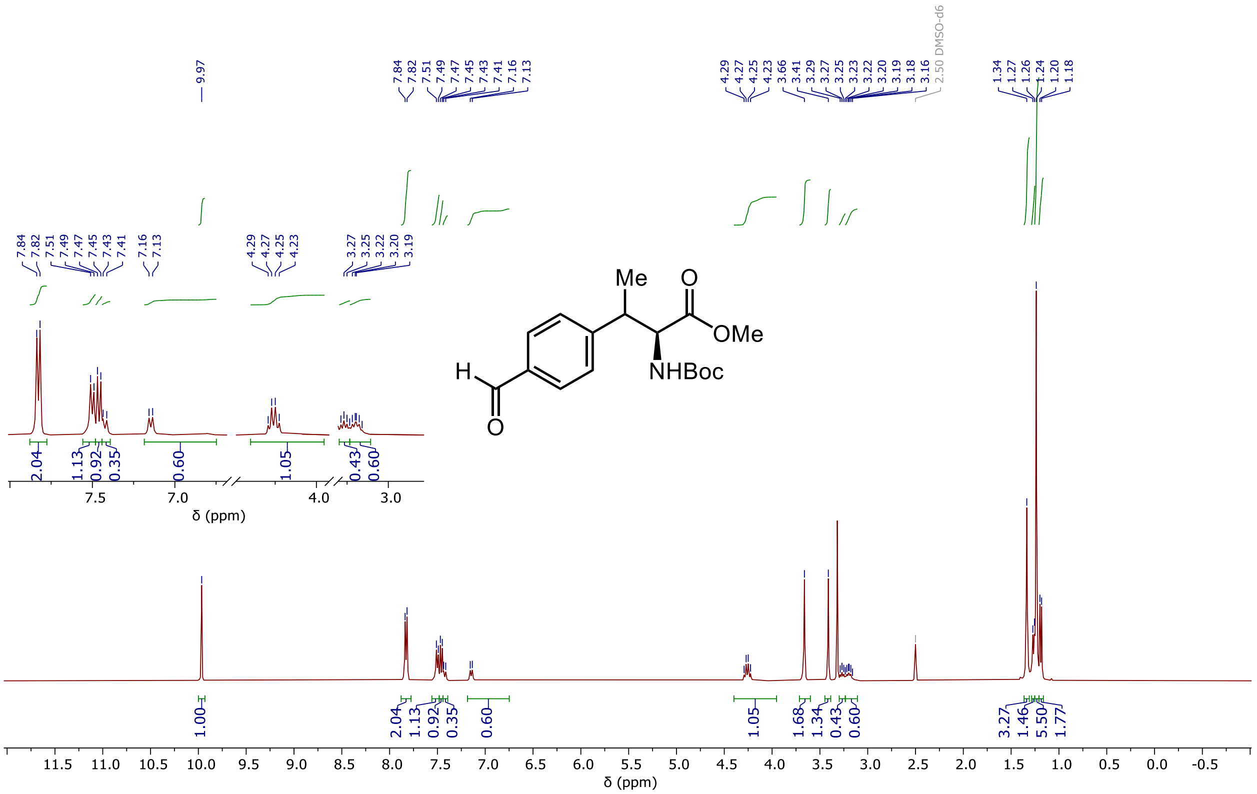

**Figure S73.** **$^{1}$H NMR spectra of (11) (400 MHz, DMSO-d$_6$).**

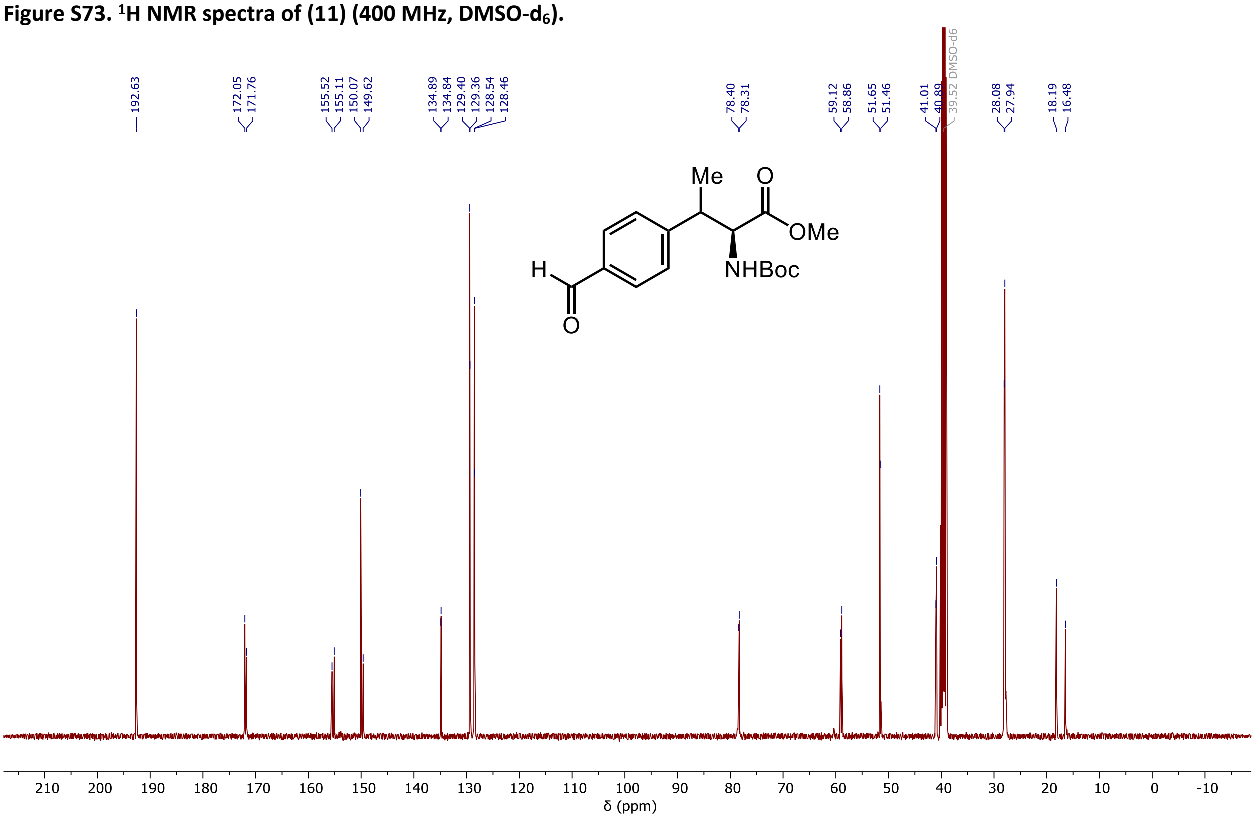

**Figure S74.** **$^{13}$C NMR spectra of (11) (101 MHz, DMSO-d$_6$).**

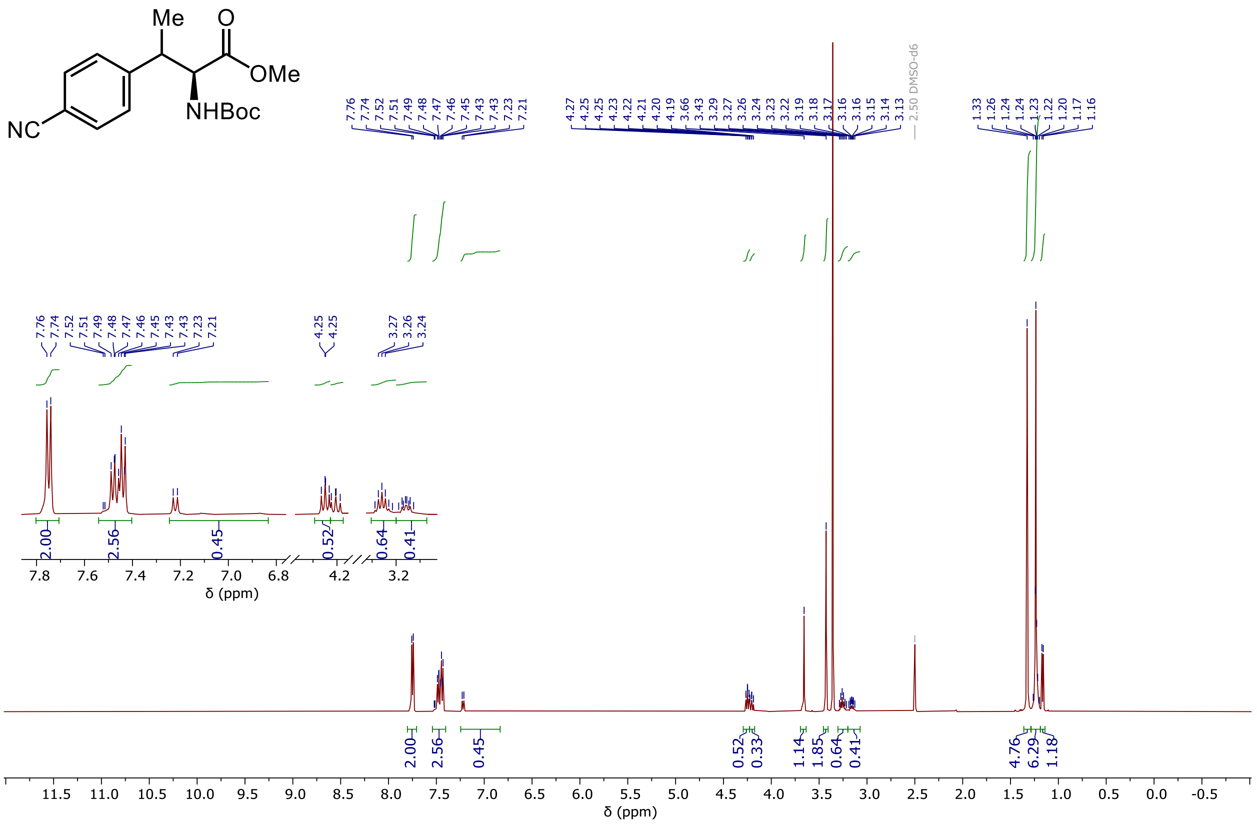

**Figure S75.** $^{1}H$ **NMR spectra of (12) (500 MHz, DMSO-d$_6$).**

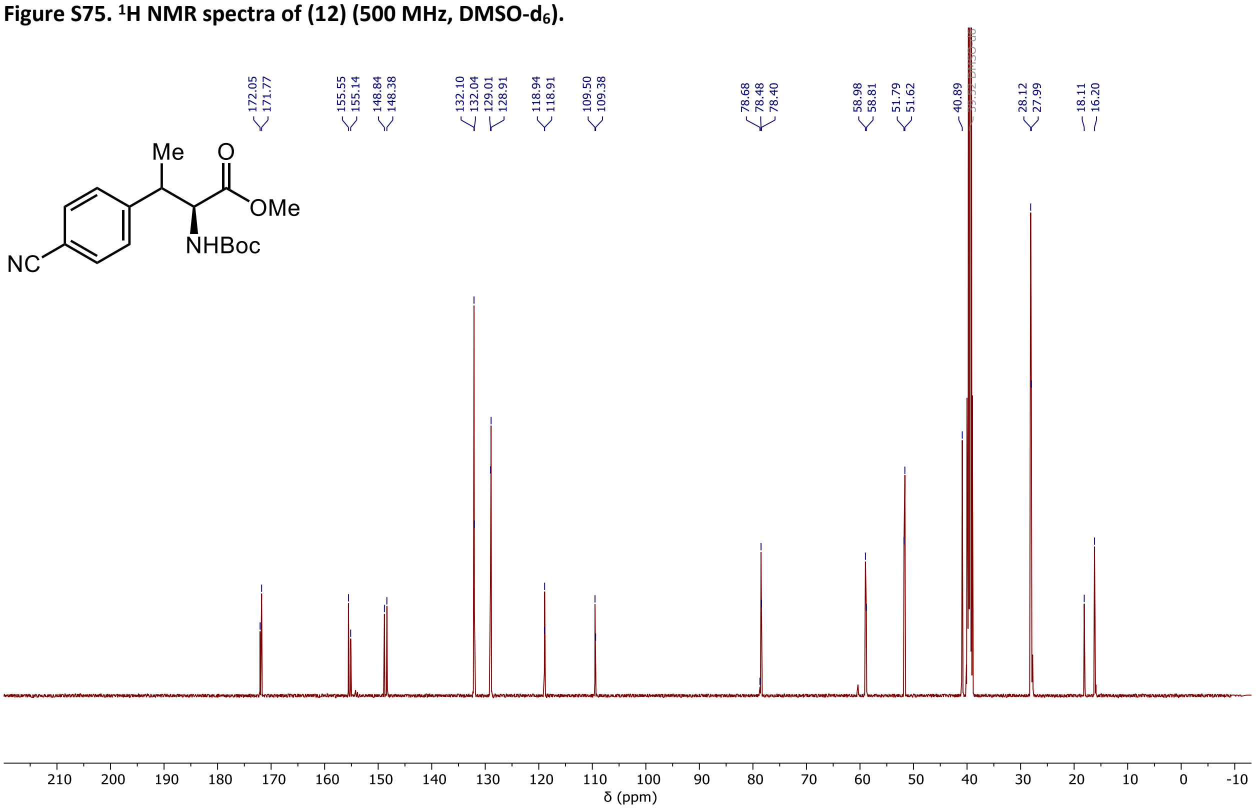

**Figure S76.** $^{13}C$ **NMR spectra of (12) (126 MHz, DMSO-d$_6$).**

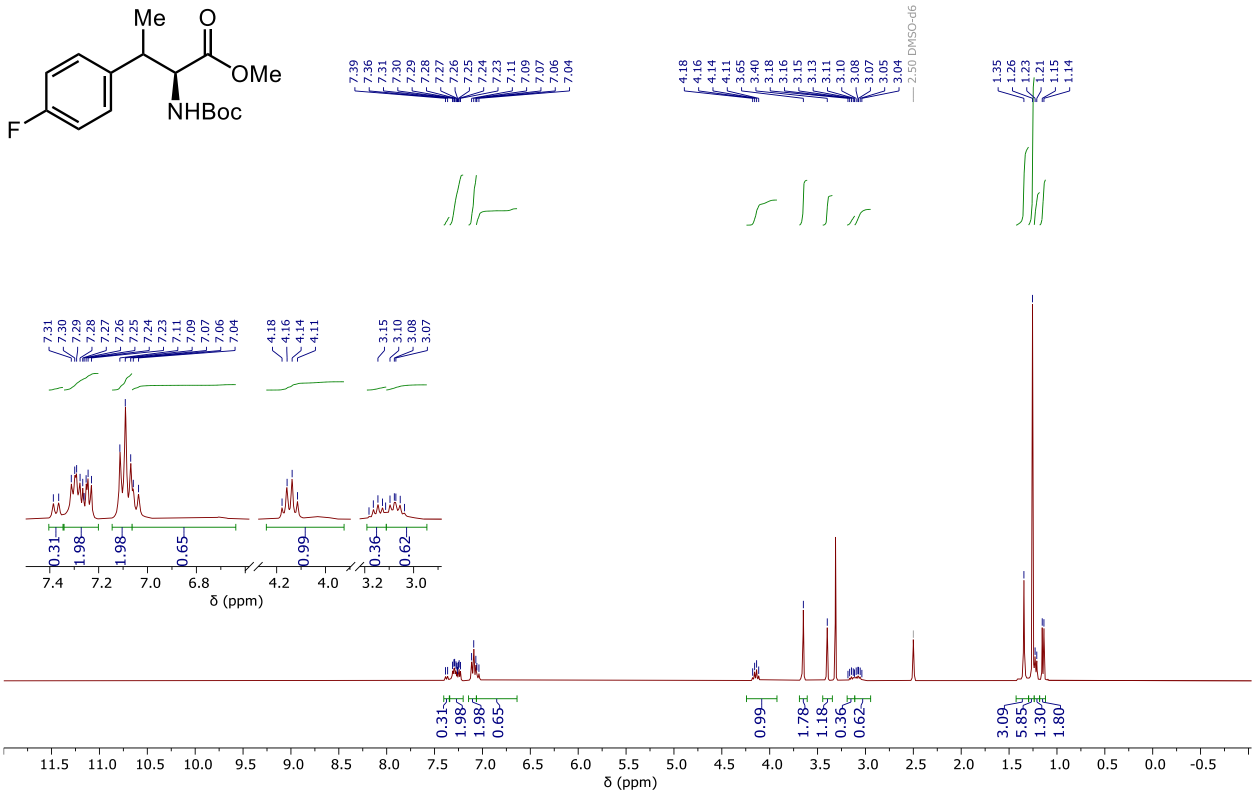

**Figure S77.** **$^{1}$H NMR spectra of (13) (400 MHz, DMSO-$d_6$).**

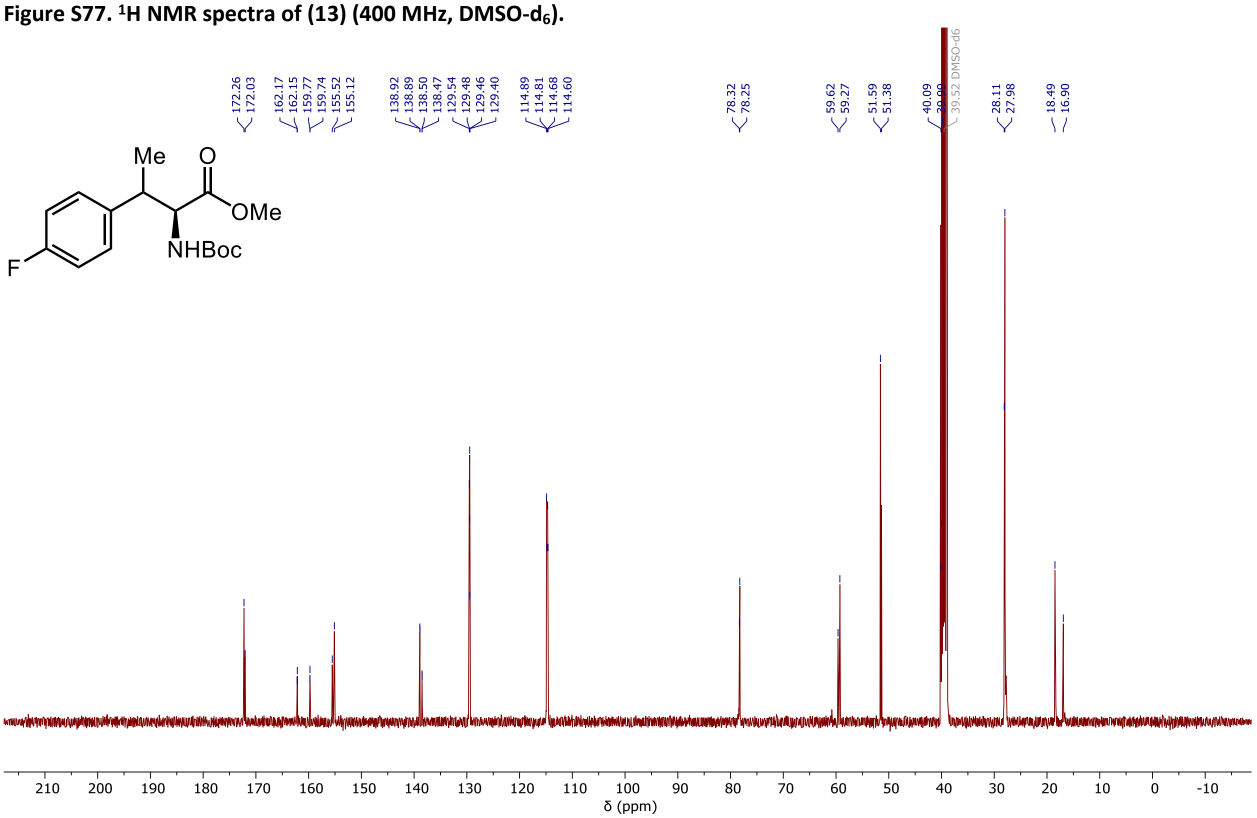

**Figure S78.** **$^{13}$C NMR spectra of (13) (101 MHz, DMSO-$d_6$).**

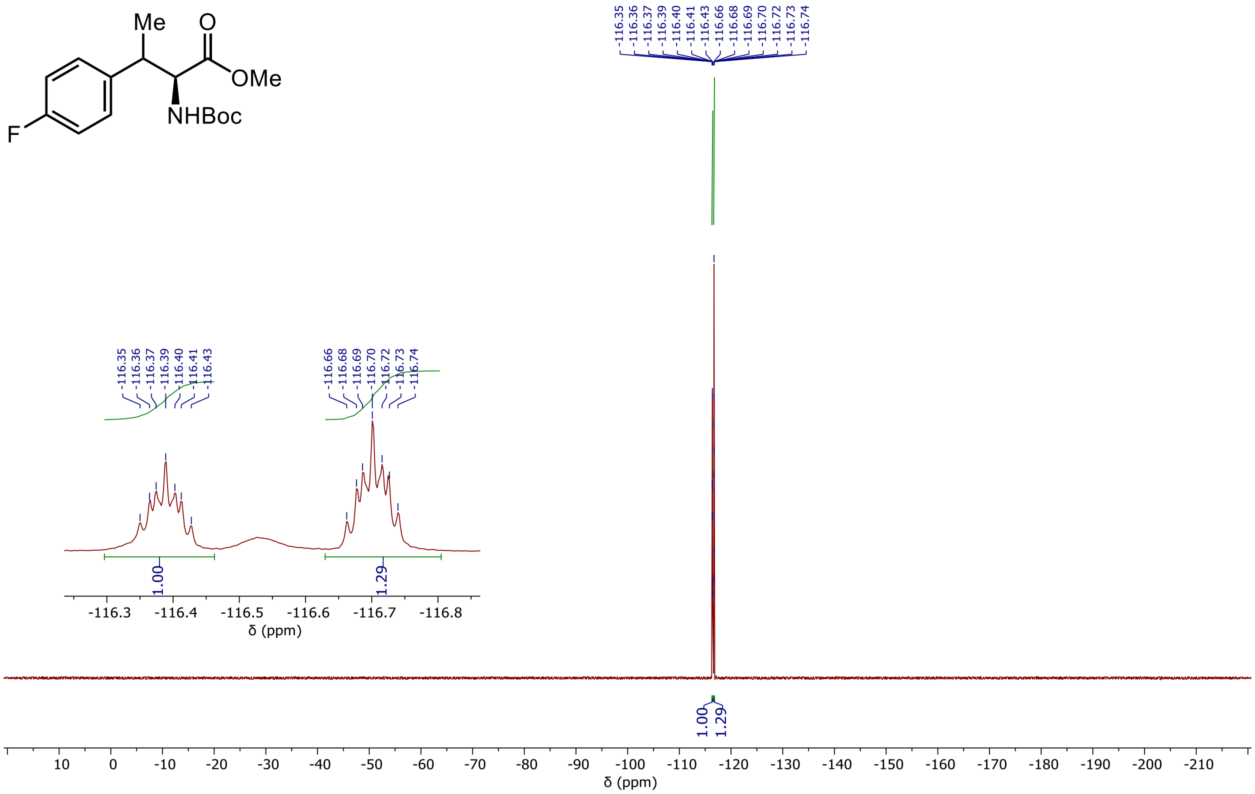

**Figure S79.** **$^{19}$F NMR spectra of (13) (376 MHz, DMSO-$d_6$).**

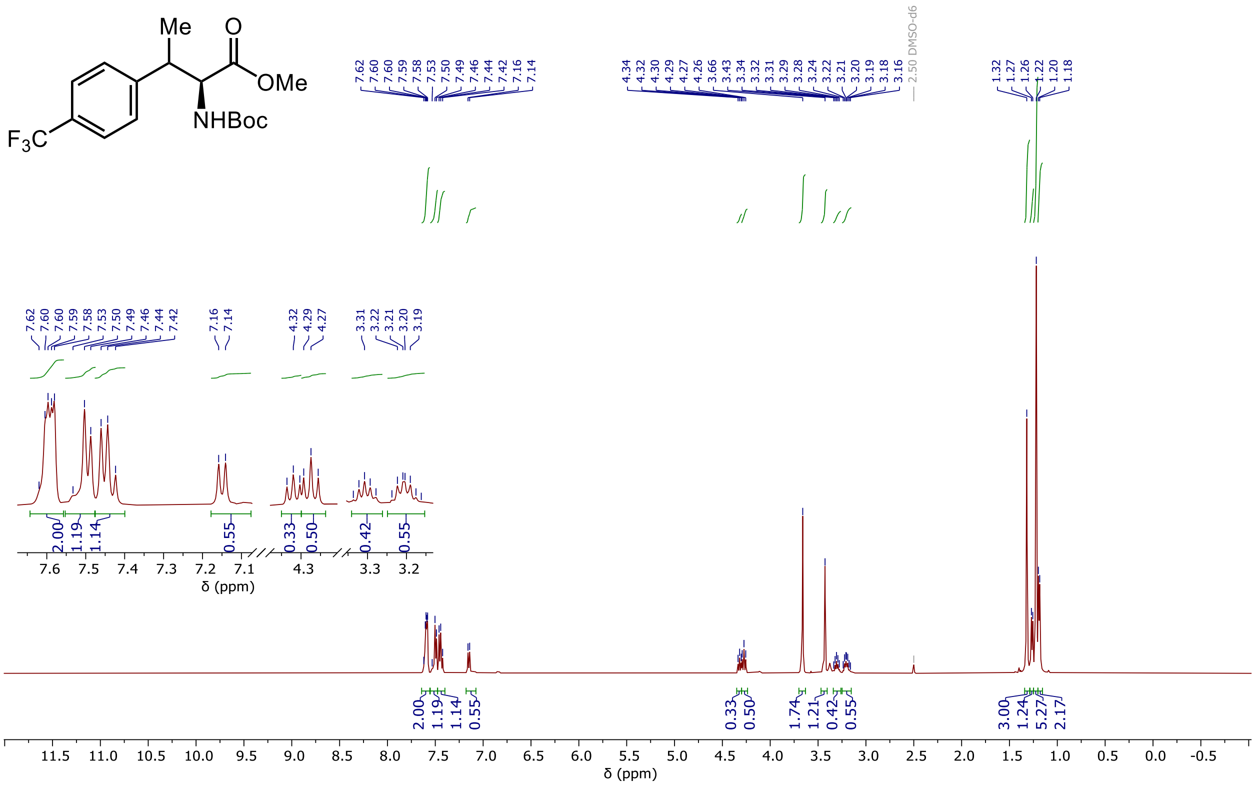

**Figure S80.** **$^{1}$H NMR spectra of (14) (500 MHz, DMSO-$d_6$).**

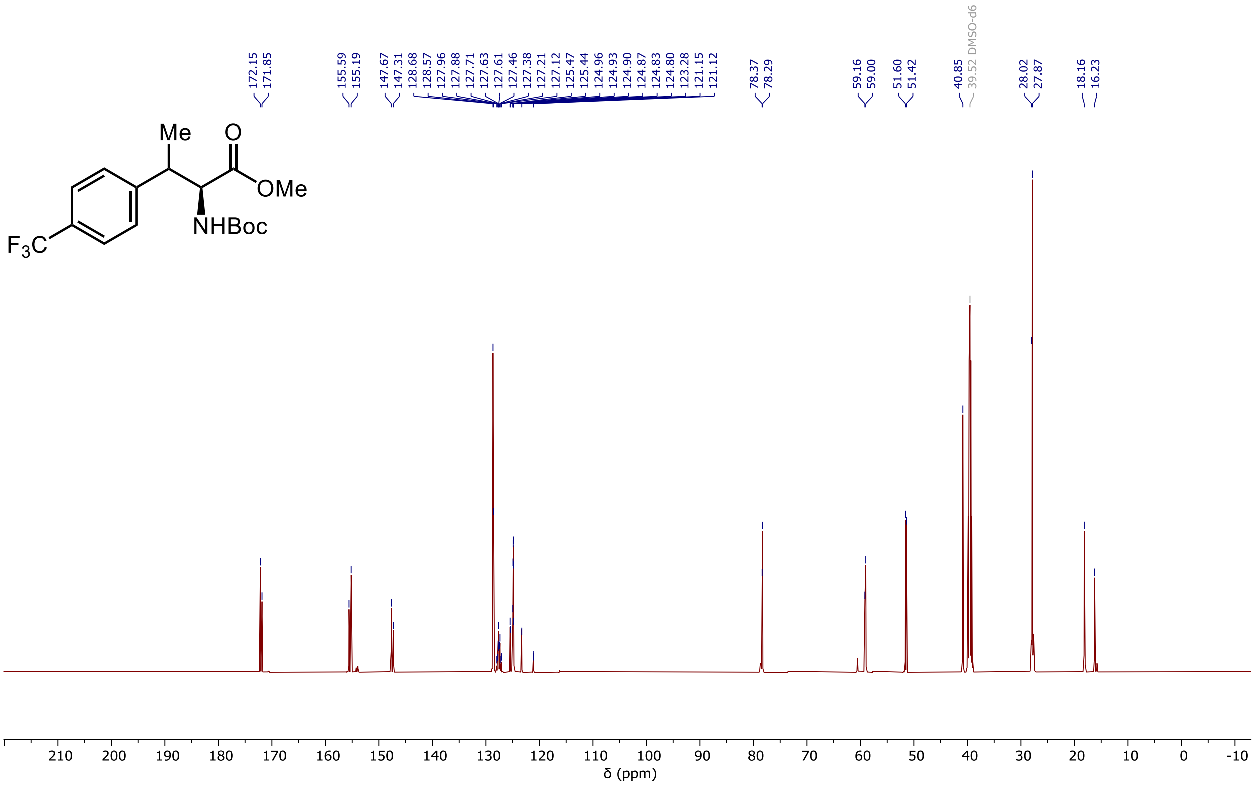

**Figure S81.** $^{13}$**C NMR spectra of (14) (126 MHz, DMSO-d$_6$).**

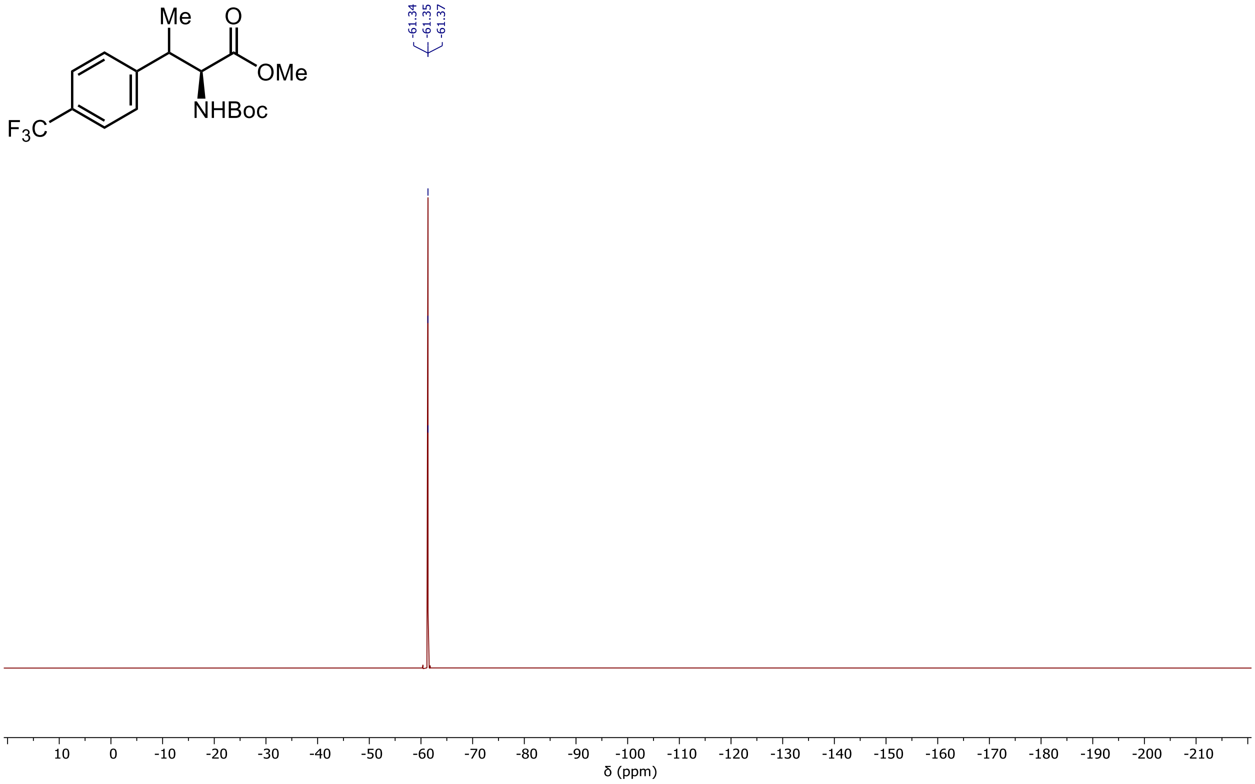

**Figure S82.** $^{19}$**F NMR spectra of (14) (470 MHz, DMSO-d$_6$).**

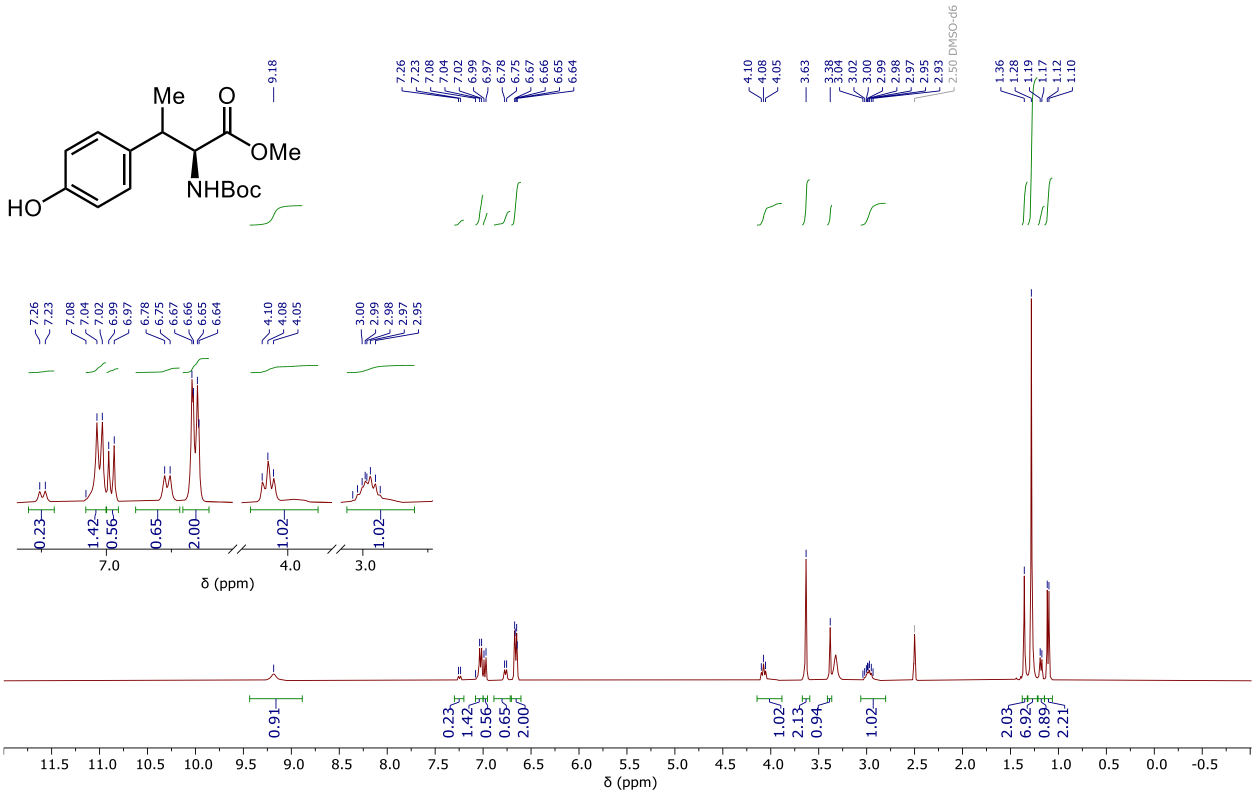

**Figure S83. $^{1}H$ NMR spectra of (15) (400 MHz, DMSO-$d_6$).**

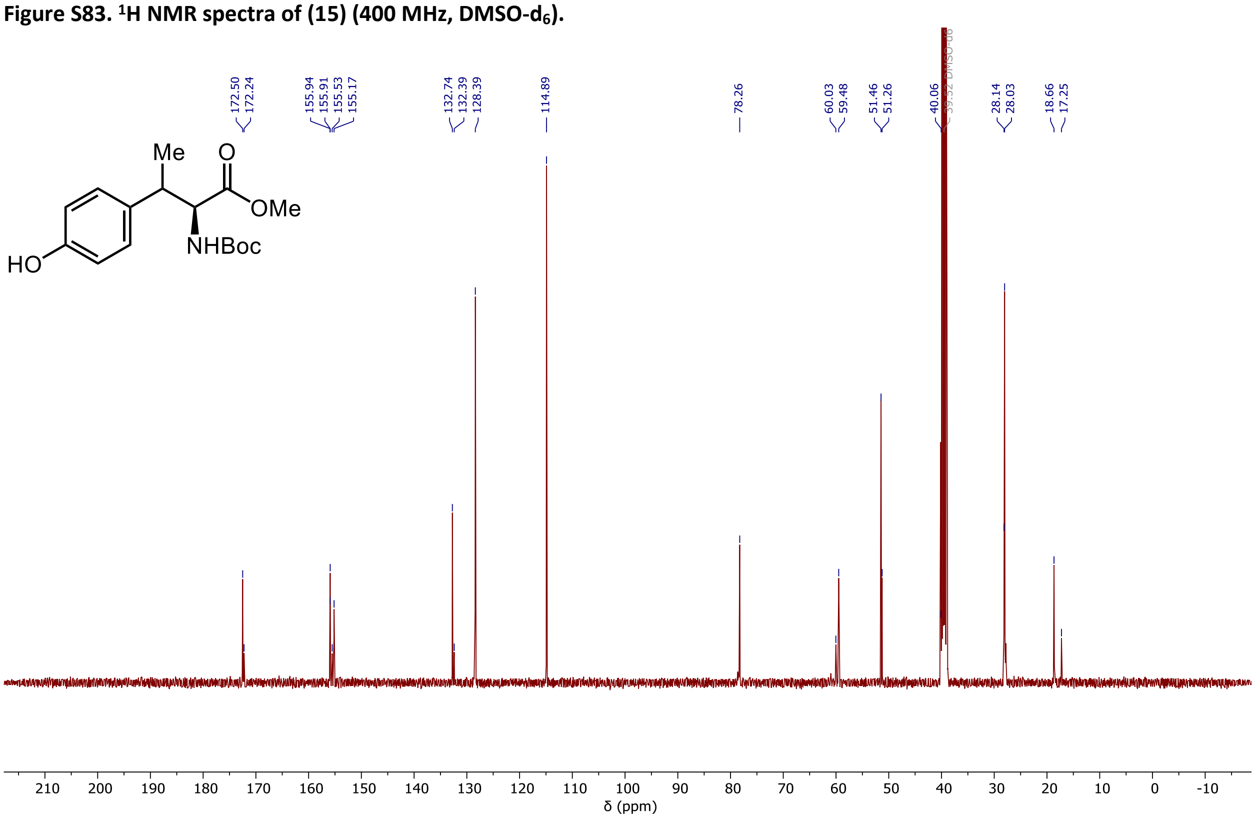

**Figure S84. $^{13}C$ NMR spectra of (15) (101 MHz, DMSO-$d_6$).**

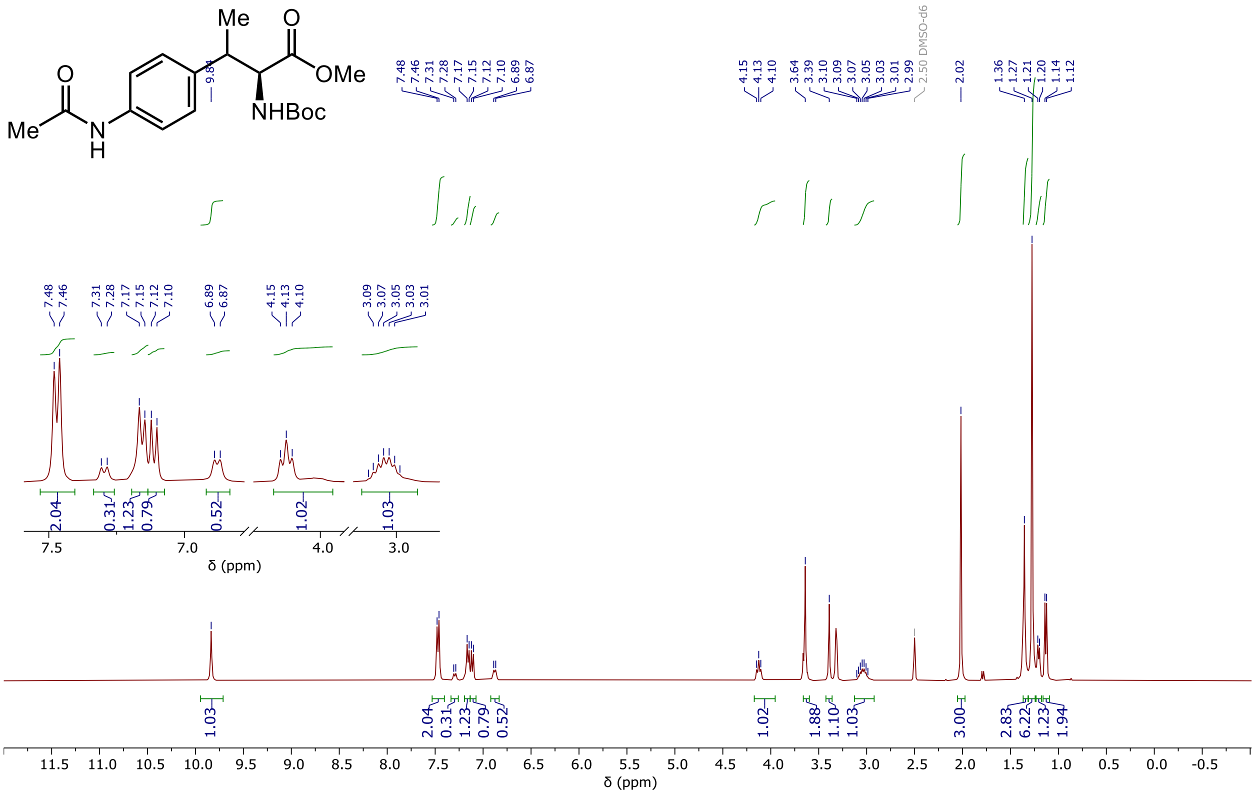

**Figure S85.** **$^{1}$H NMR spectra of (16) (400 MHz, DMSO-$d_6$).**

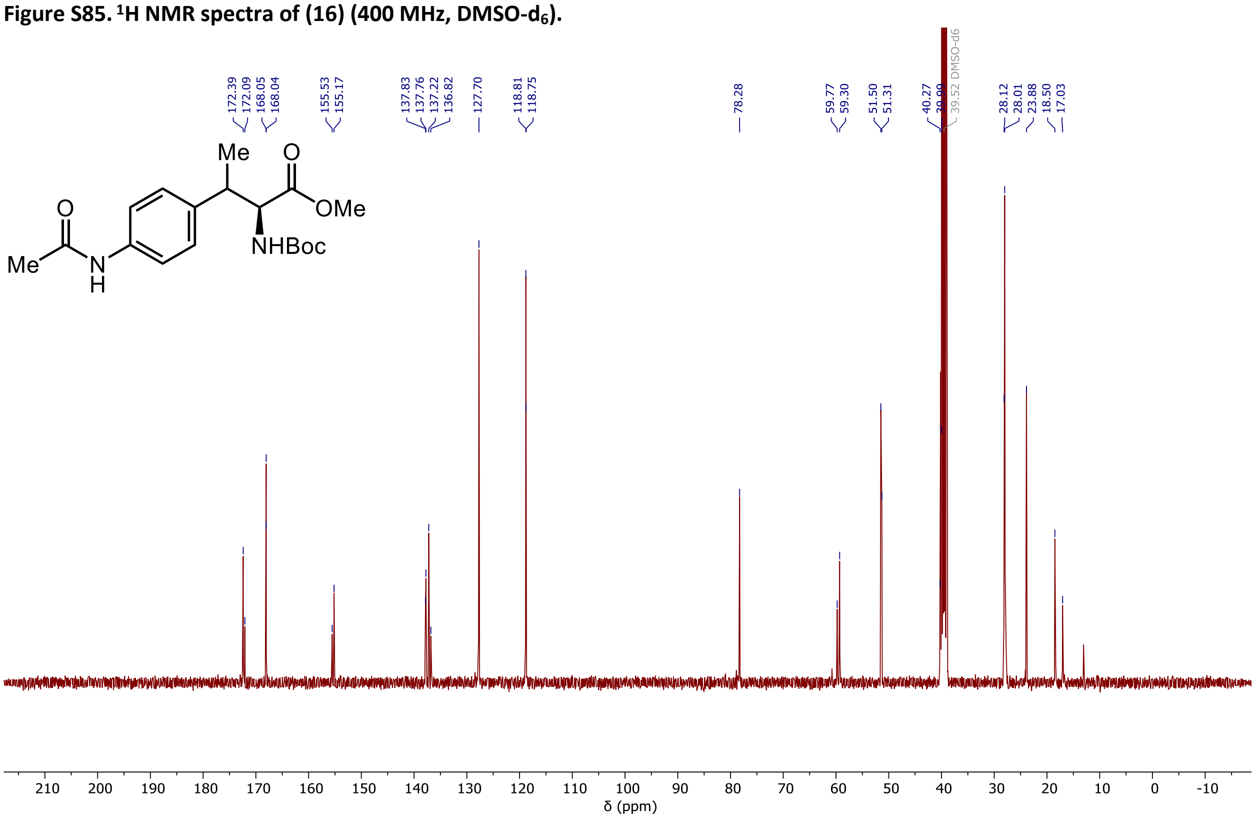

**Figure S86.** **$^{13}$C NMR spectra of (16) (101 MHz, DMSO-$d_6$).**

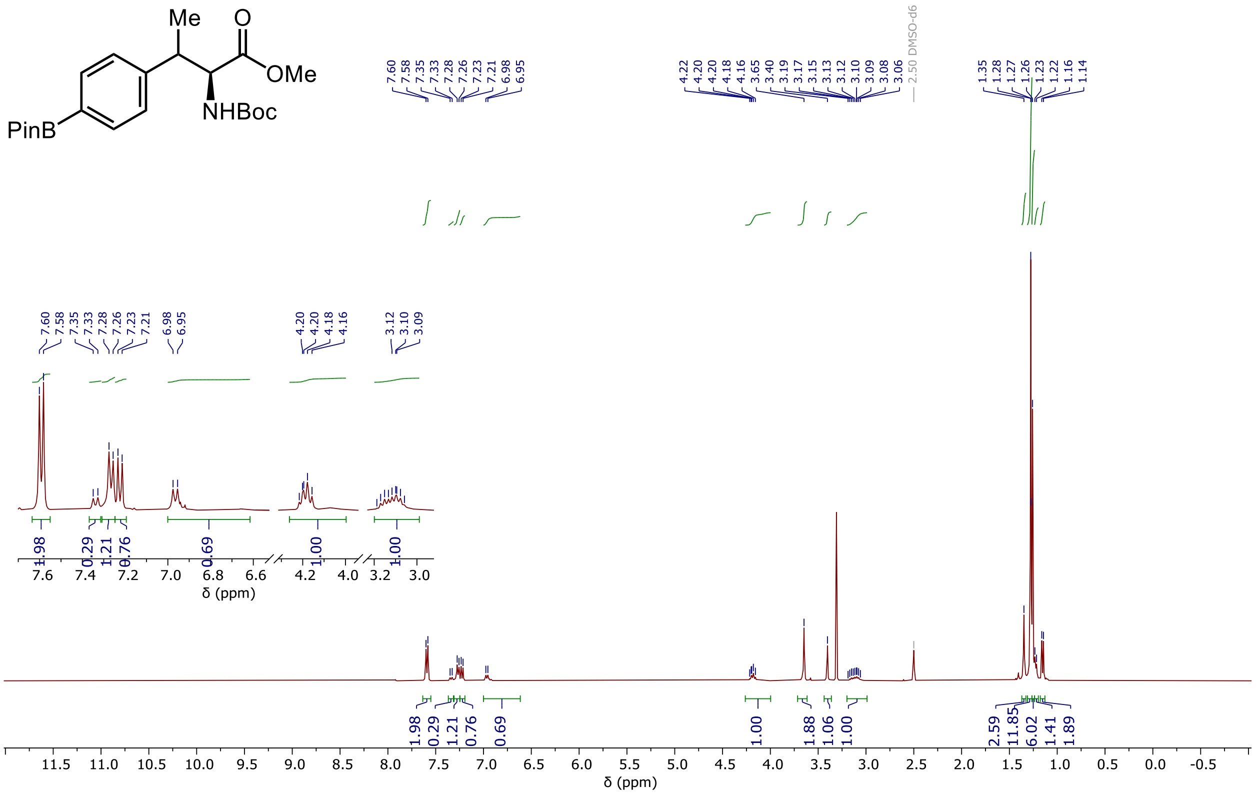

**Figure S87. $^{1}H$ NMR spectra of (17) (400 MHz, DMSO-$d_6$).**

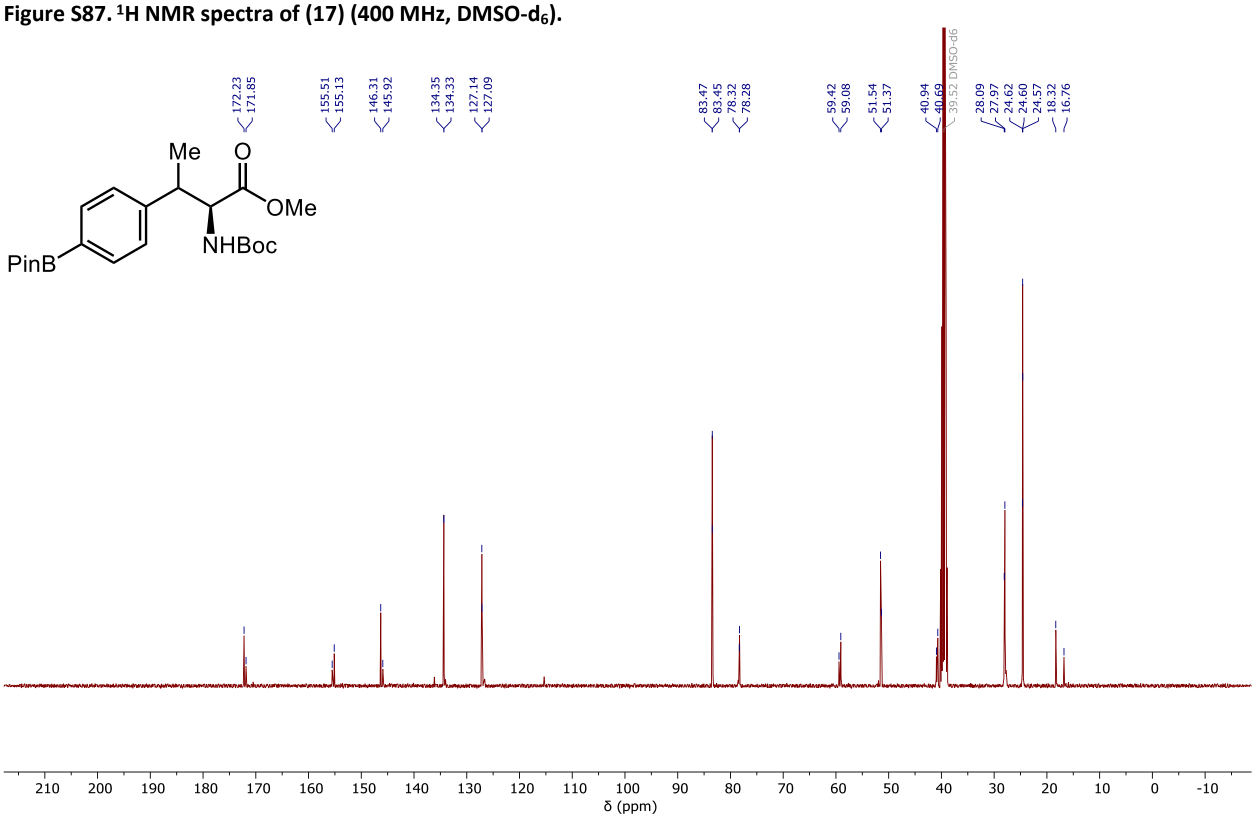

**Figure S88. $^{13}C$ NMR spectra of (17) (101 MHz, DMSO-$d_6$).**

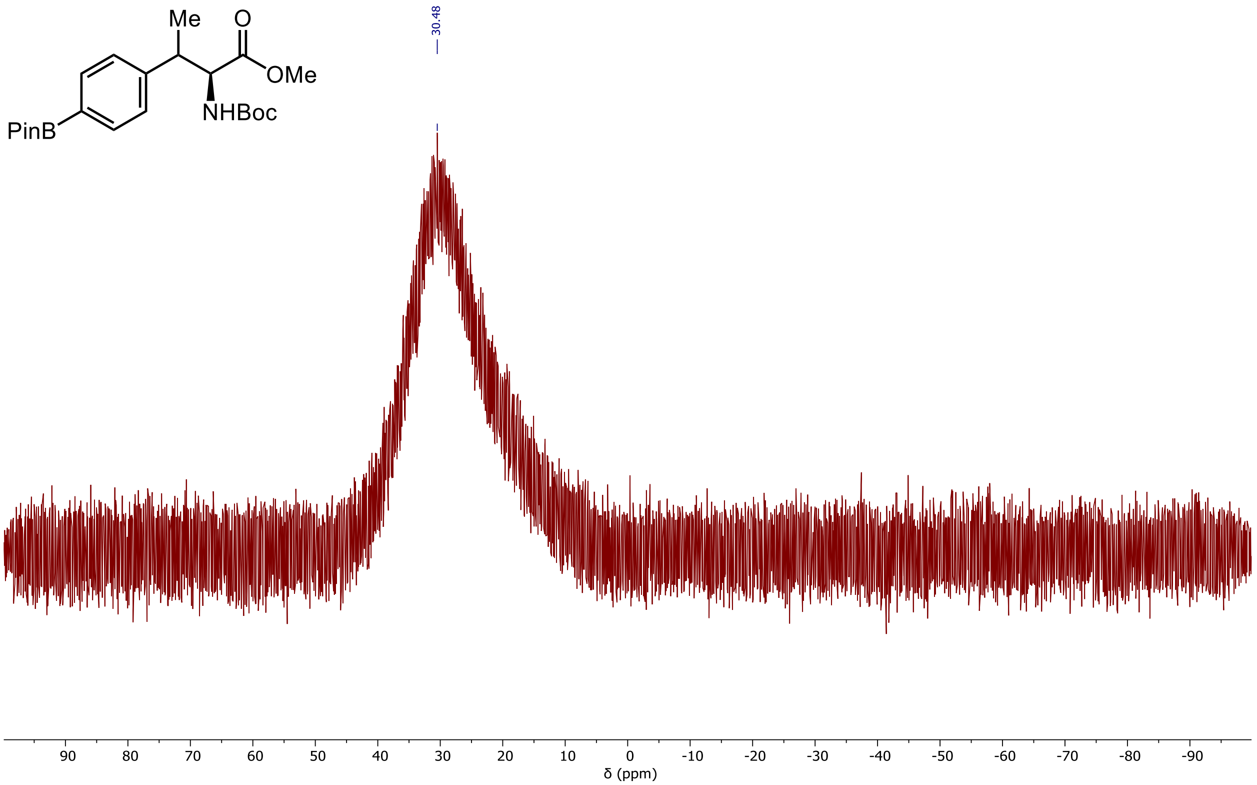


**Figure S89.** $^{11}$**B NMR spectra of (17) (160 MHz, DMSO-d$_6$).**

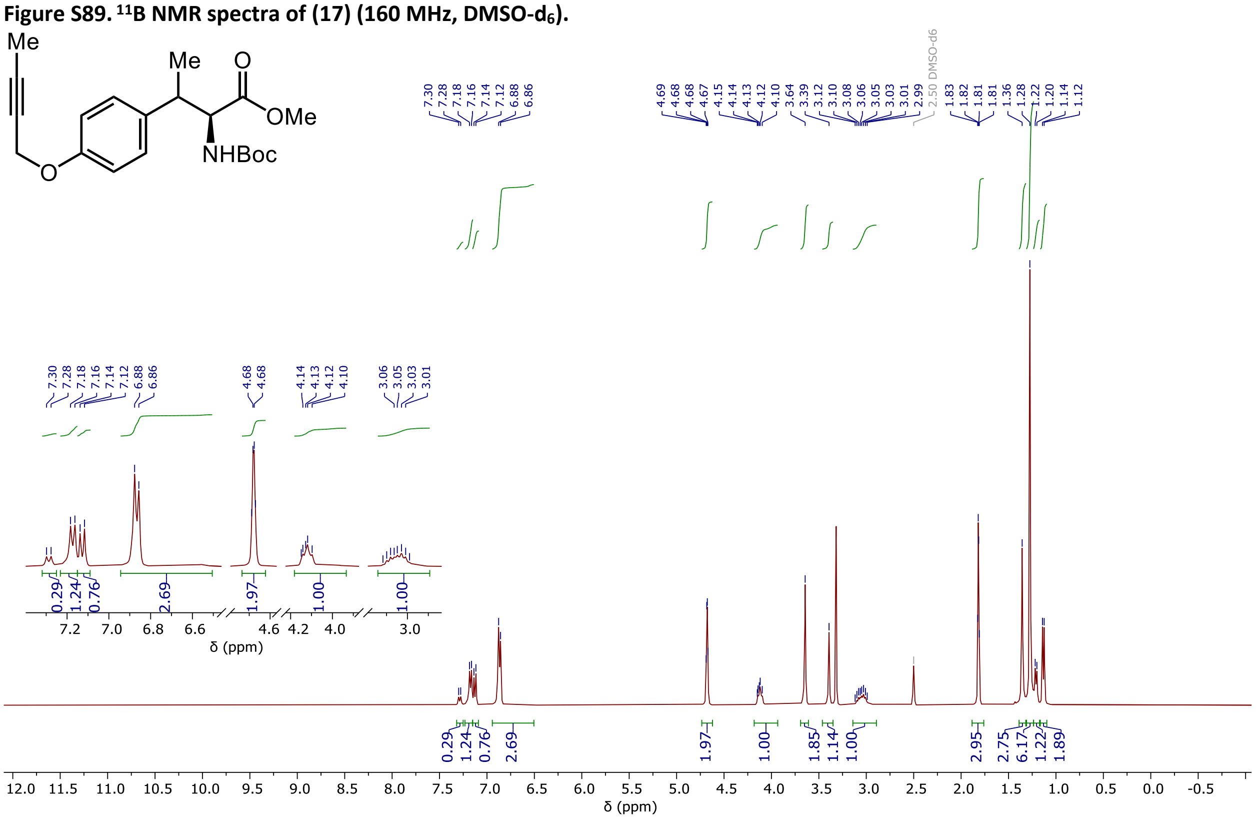


**Figure S90.** $^{1}$**H NMR spectra of (18) (400 MHz, DMSO-d$_6$).**

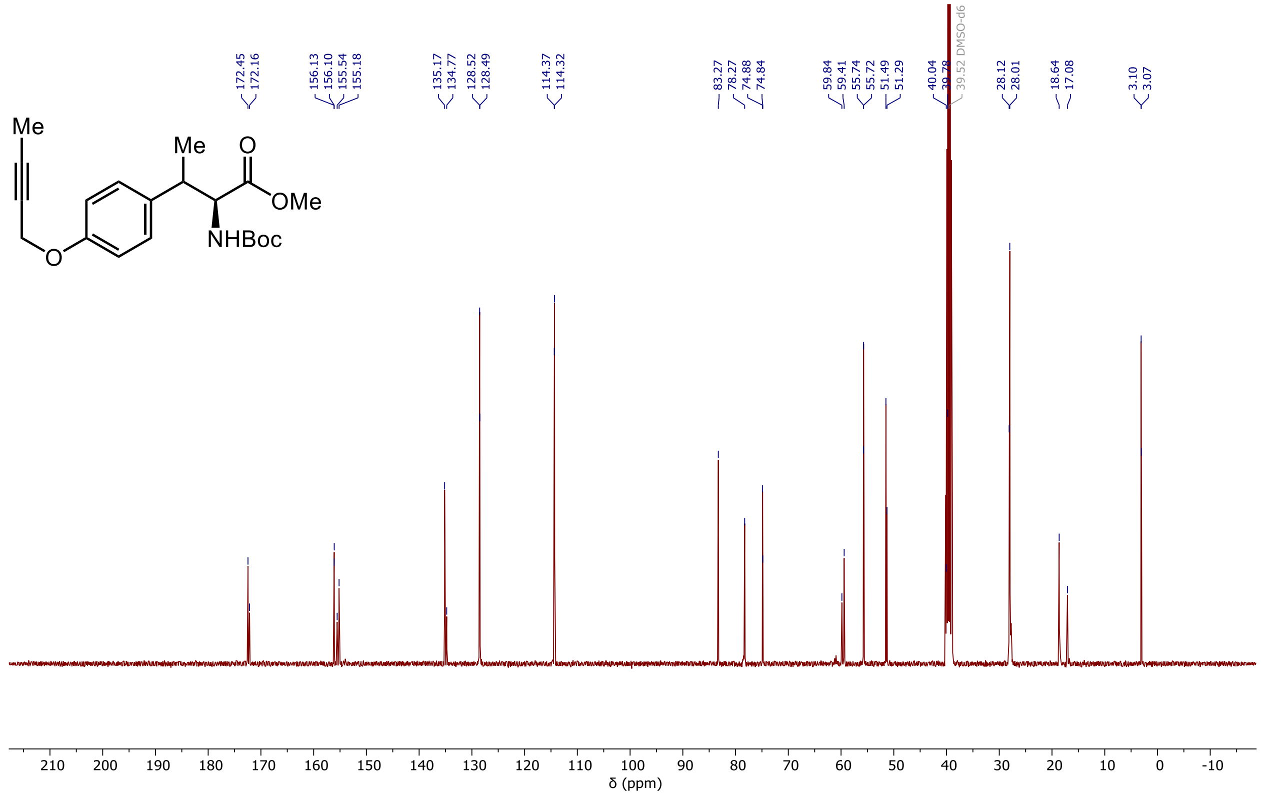

**Figure S91.** $^{13}$**C NMR spectra of (18) (101 MHz, DMSO-d$_6$).**

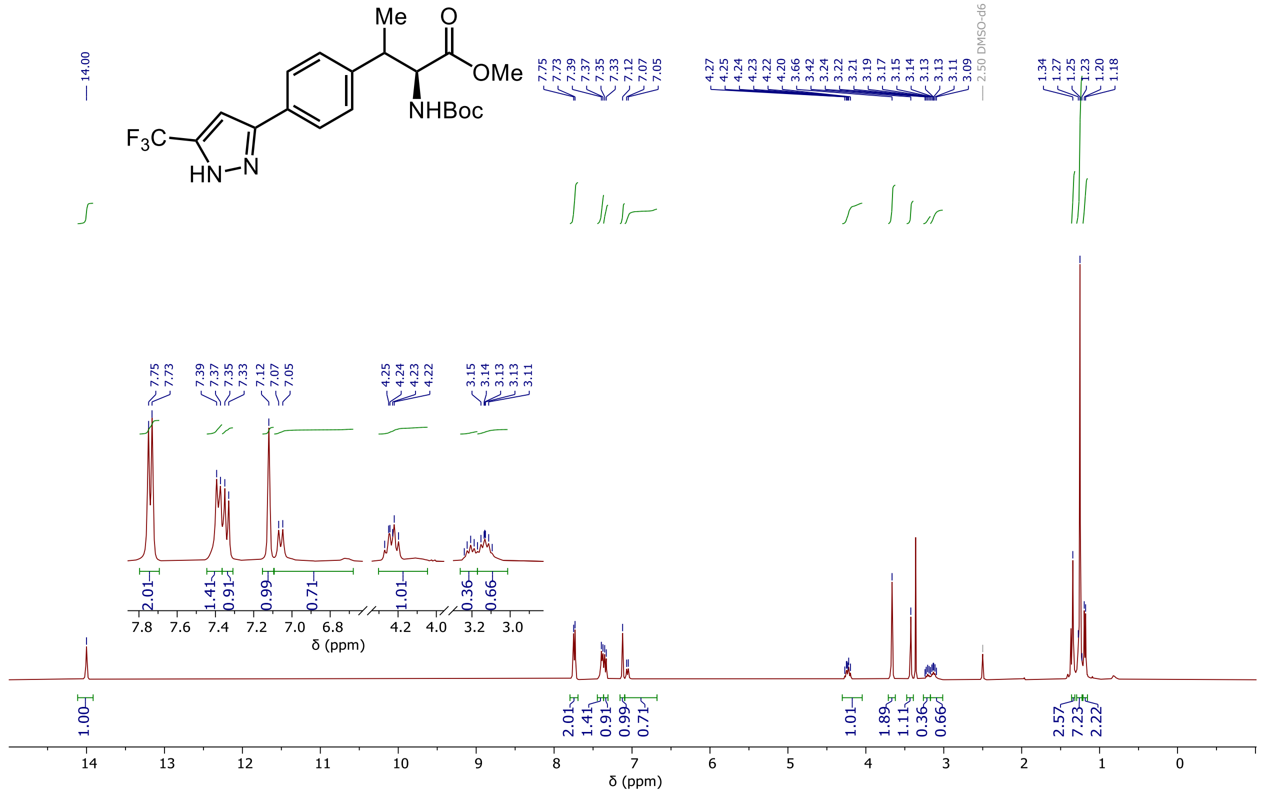

**Figure S92.** $^{1}$**H NMR spectra of (19) (400 MHz, DMSO-d$_6$).**

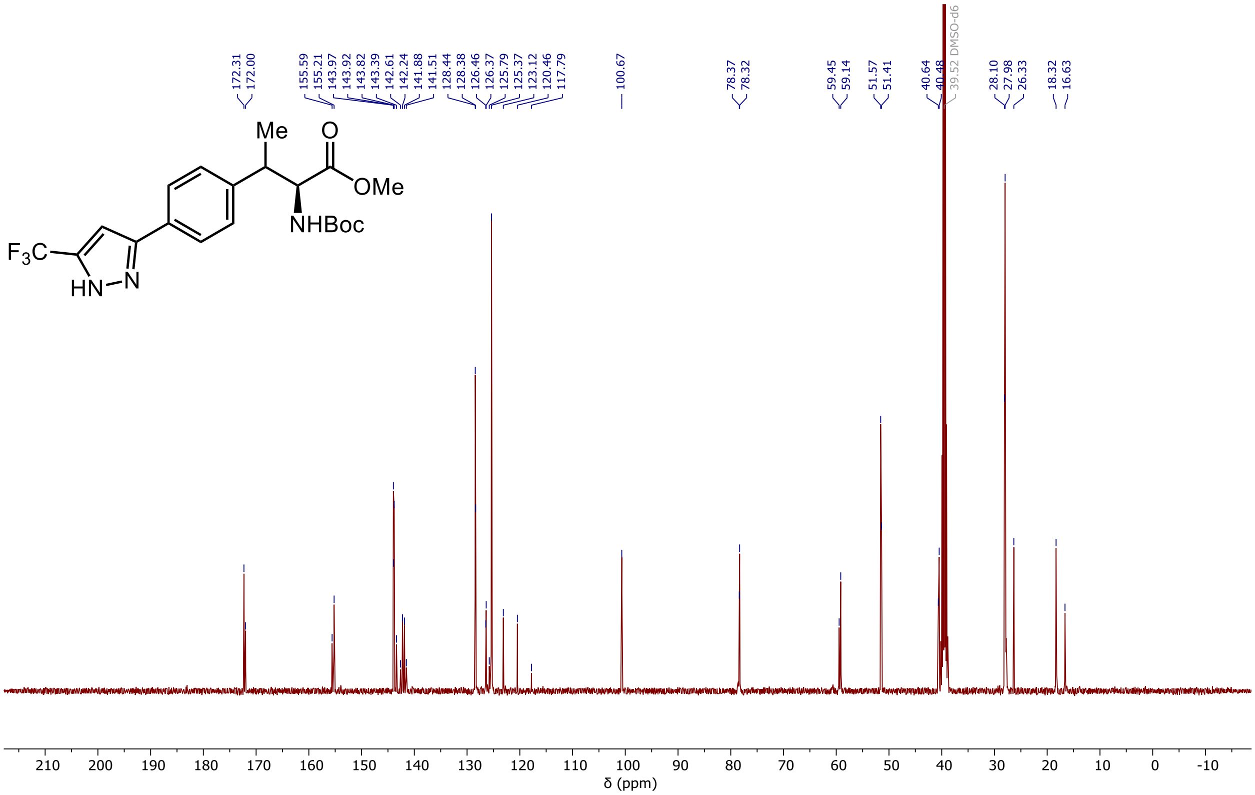


**Figure S93.** **$^{13}$C NMR spectra of (19) (101 MHz, DMSO-d$_6$).**

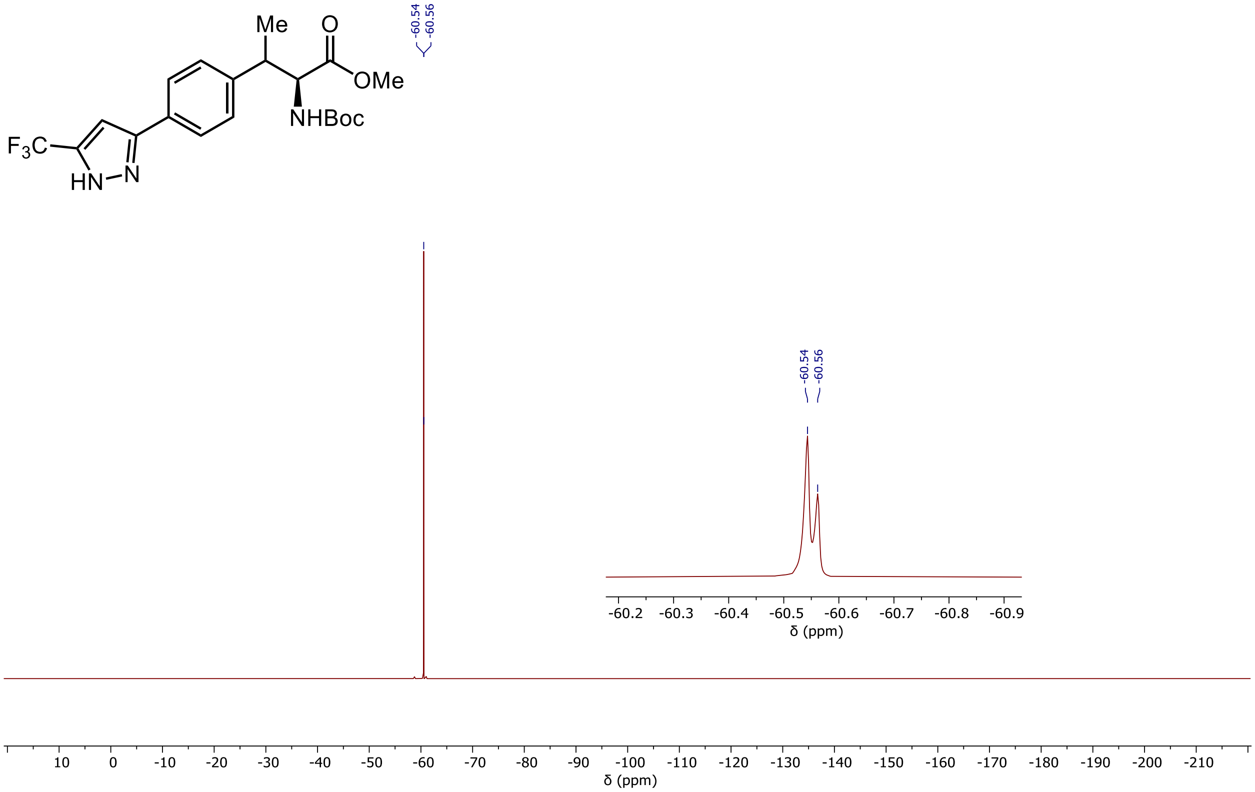


**Figure S94.** **$^{19}$F NMR spectra of (19) (376 MHz, DMSO-d$_6$).**

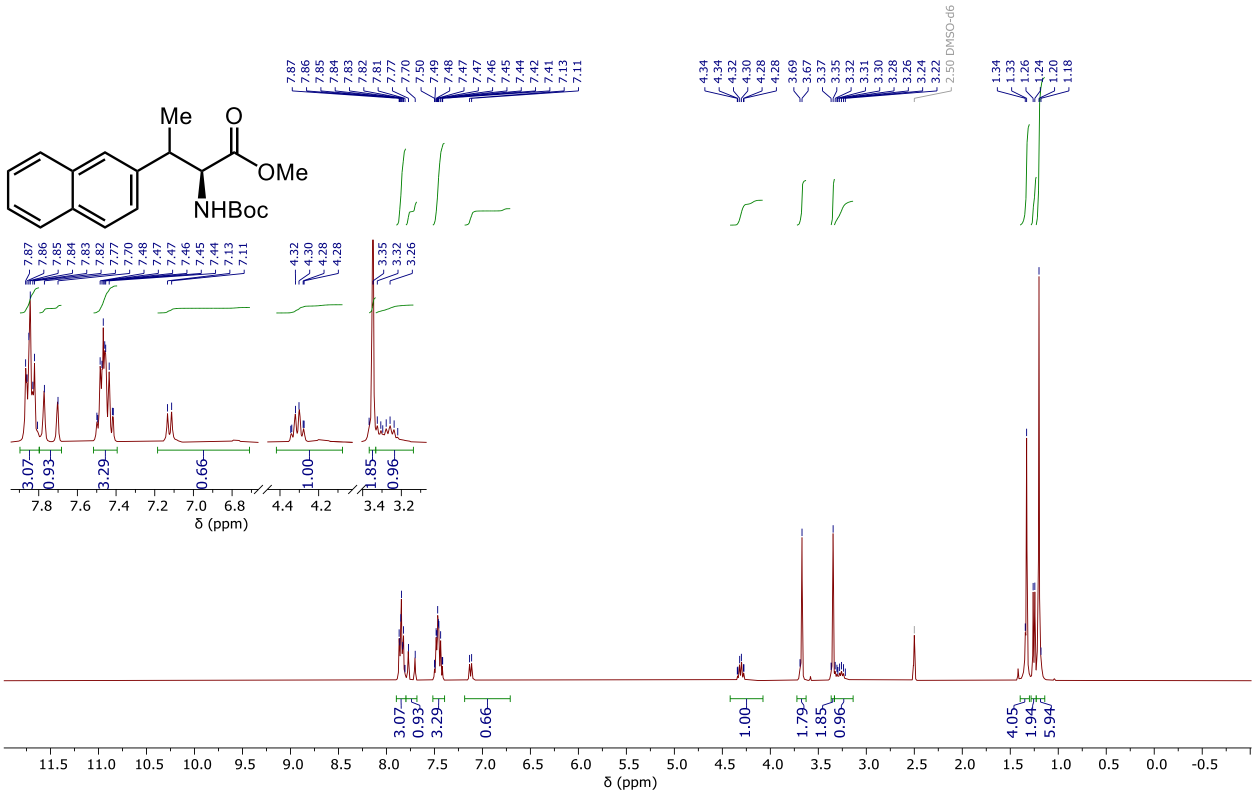

**Figure S95. $^{1}$H NMR spectra of (20) (400 MHz, DMSO-$d_6$).**

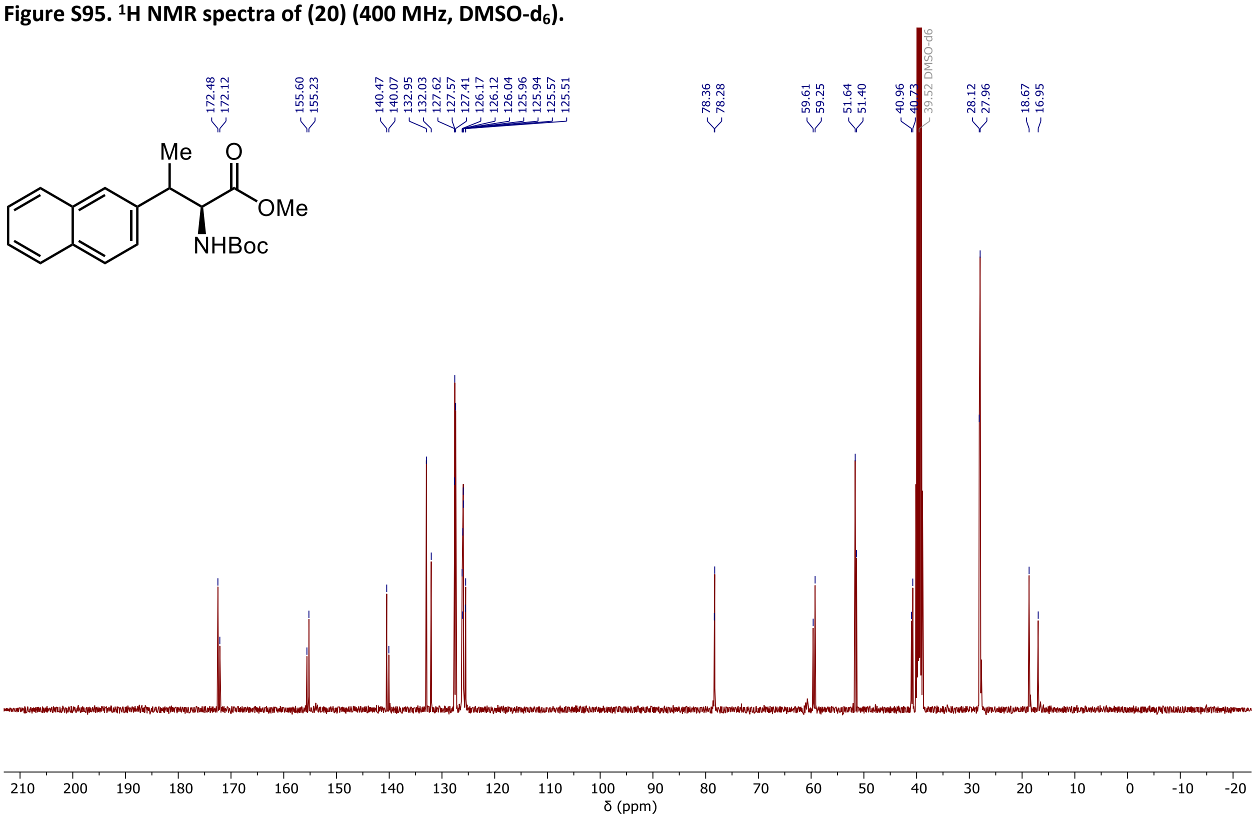

**Figure S96. $^{13}$C NMR spectra of (20) (101 MHz, DMSO-$d_6$).**

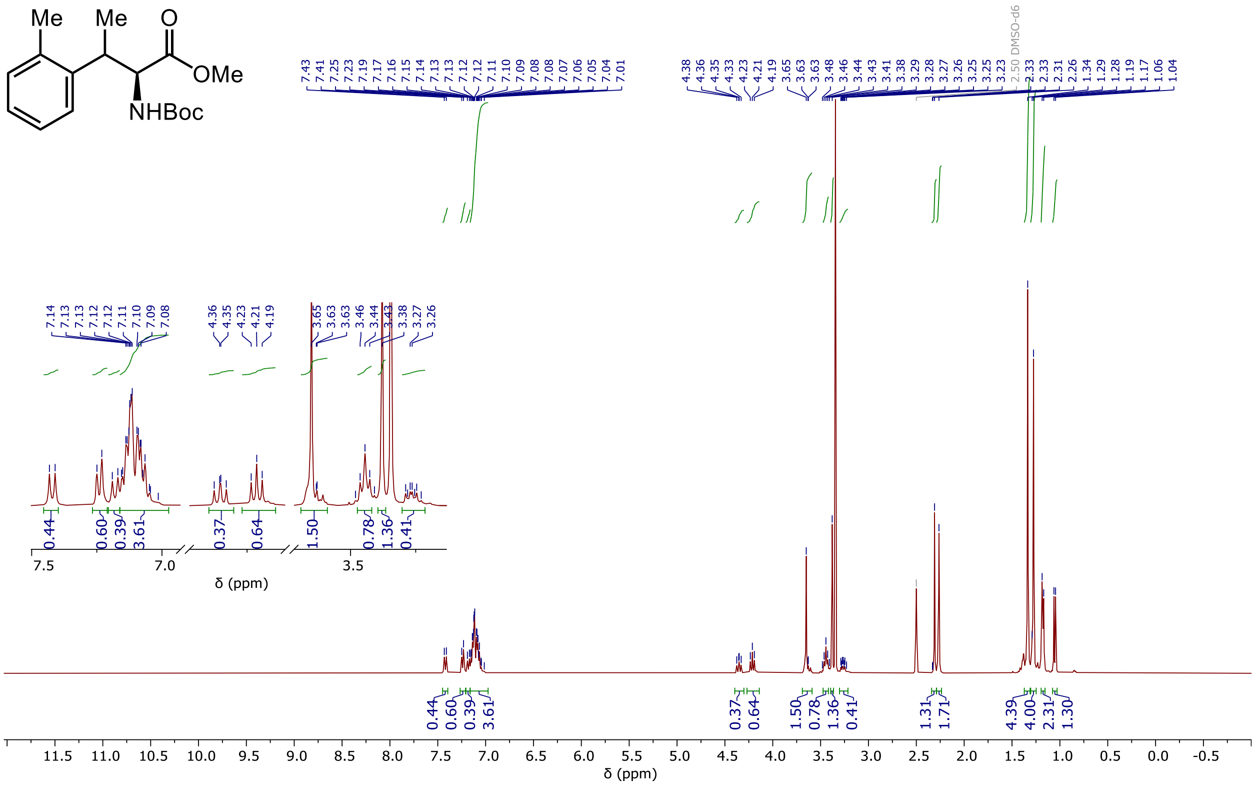

**Figure S97. $^{1}$H NMR spectra of (21) (400 MHz, DMSO-$d_6$).**

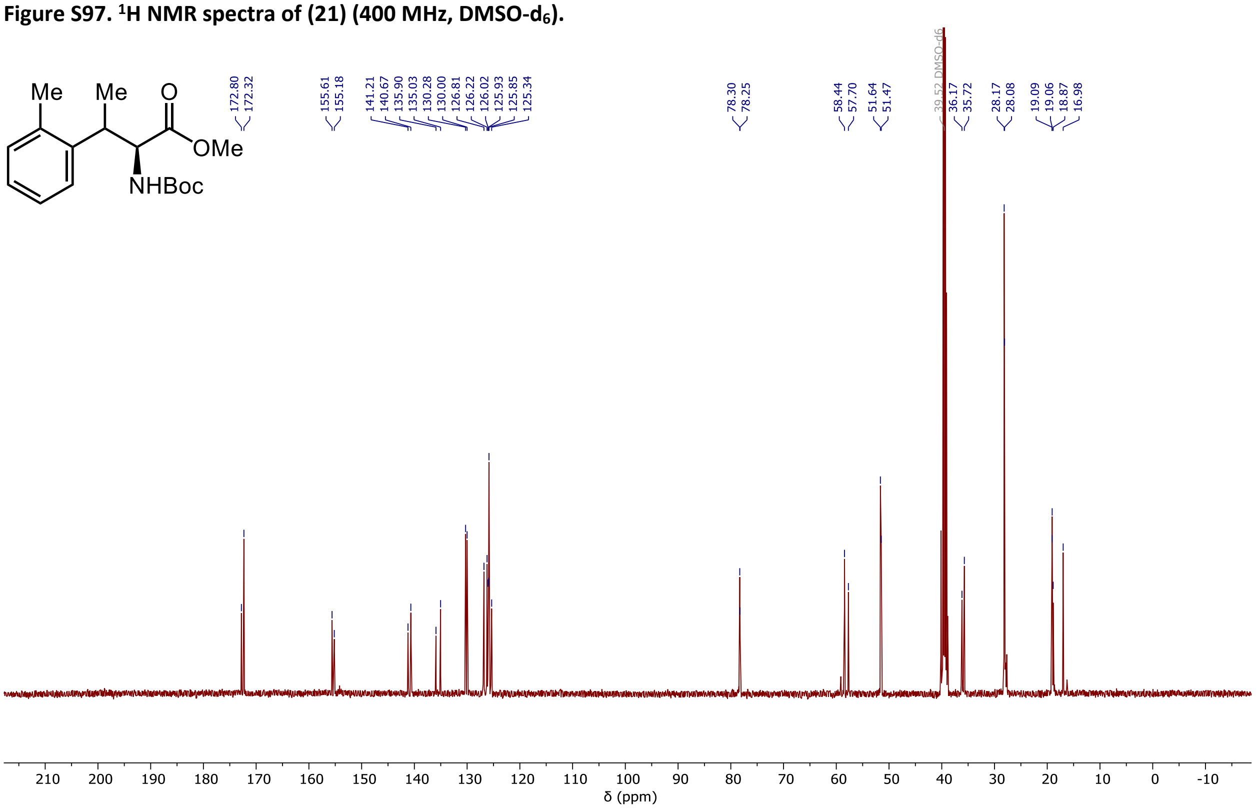

**Figure S98. $^{13}$C NMR spectra of (21) (101 MHz, DMSO-$d_6$).**

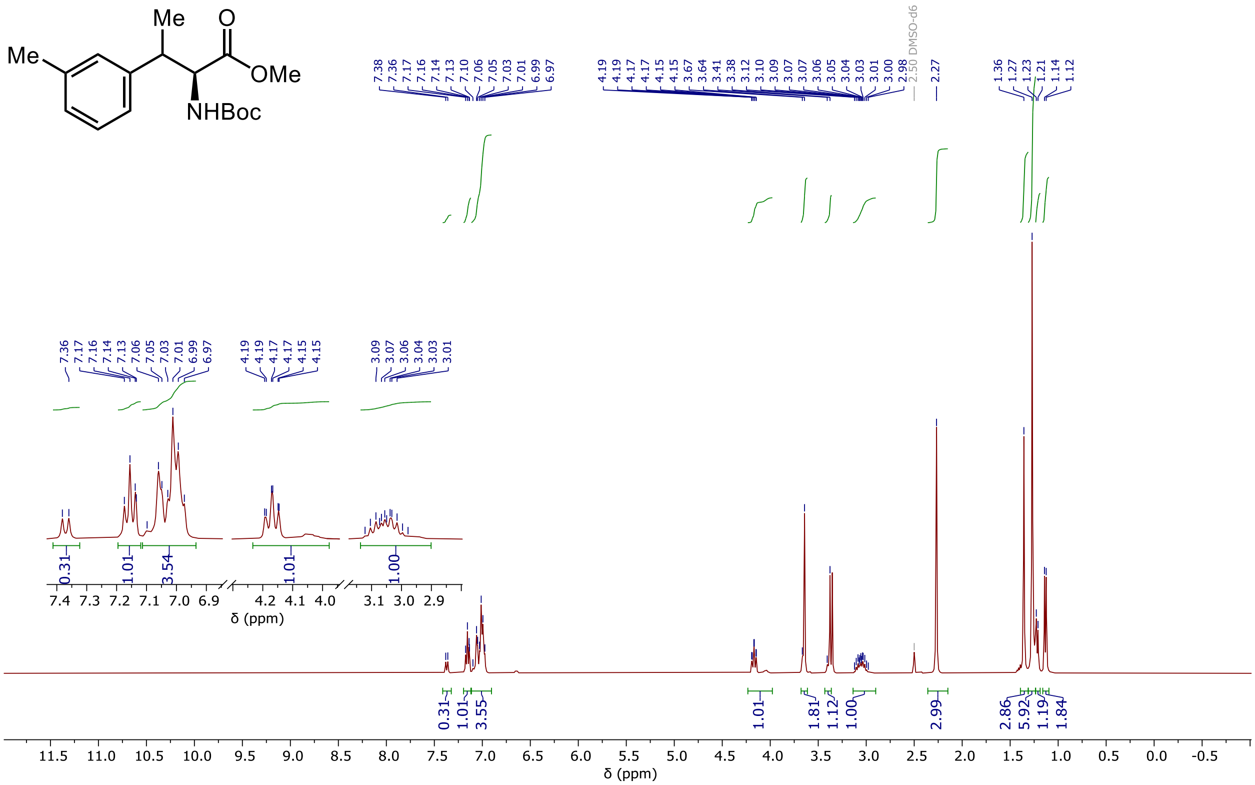

**Figure S99. $^{1}$H NMR spectra of (22) (400 MHz, DMSO-$d_6$).**

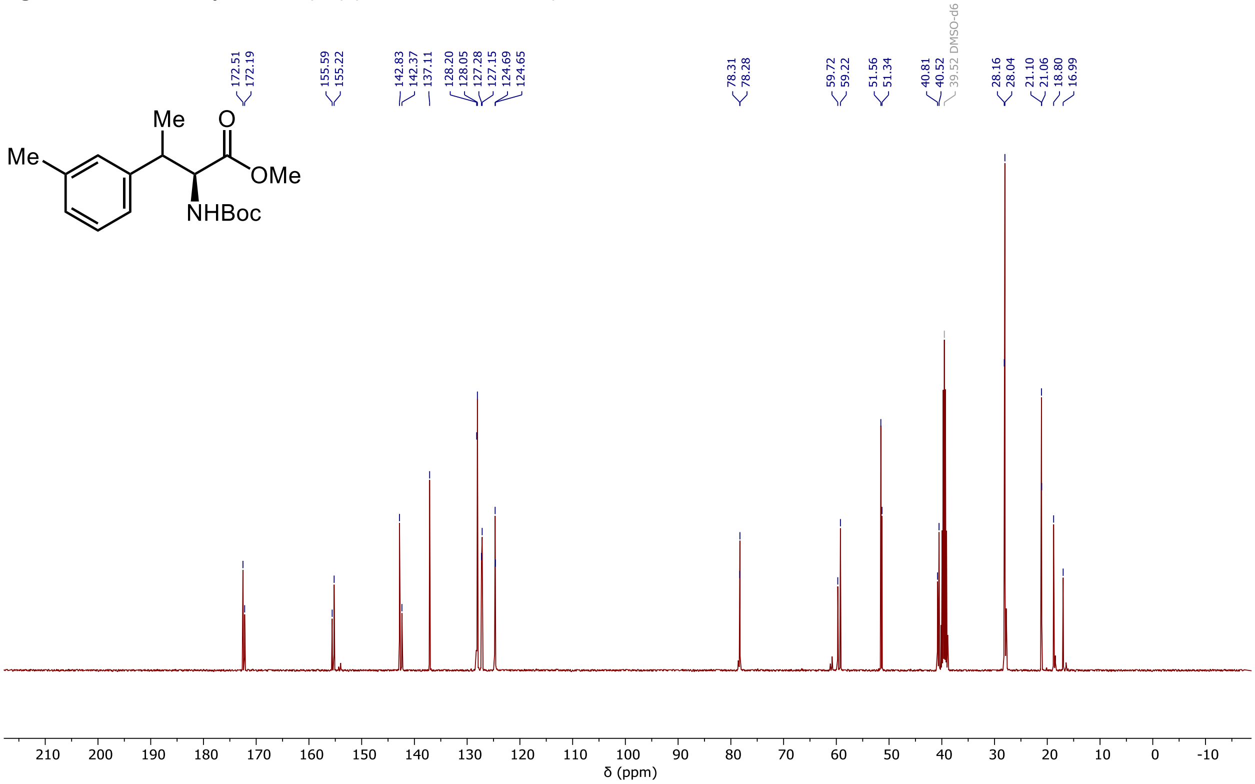

**Figure S100. $^{13}$C NMR spectra of (22) (101 MHz, DMSO-$d_6$).**

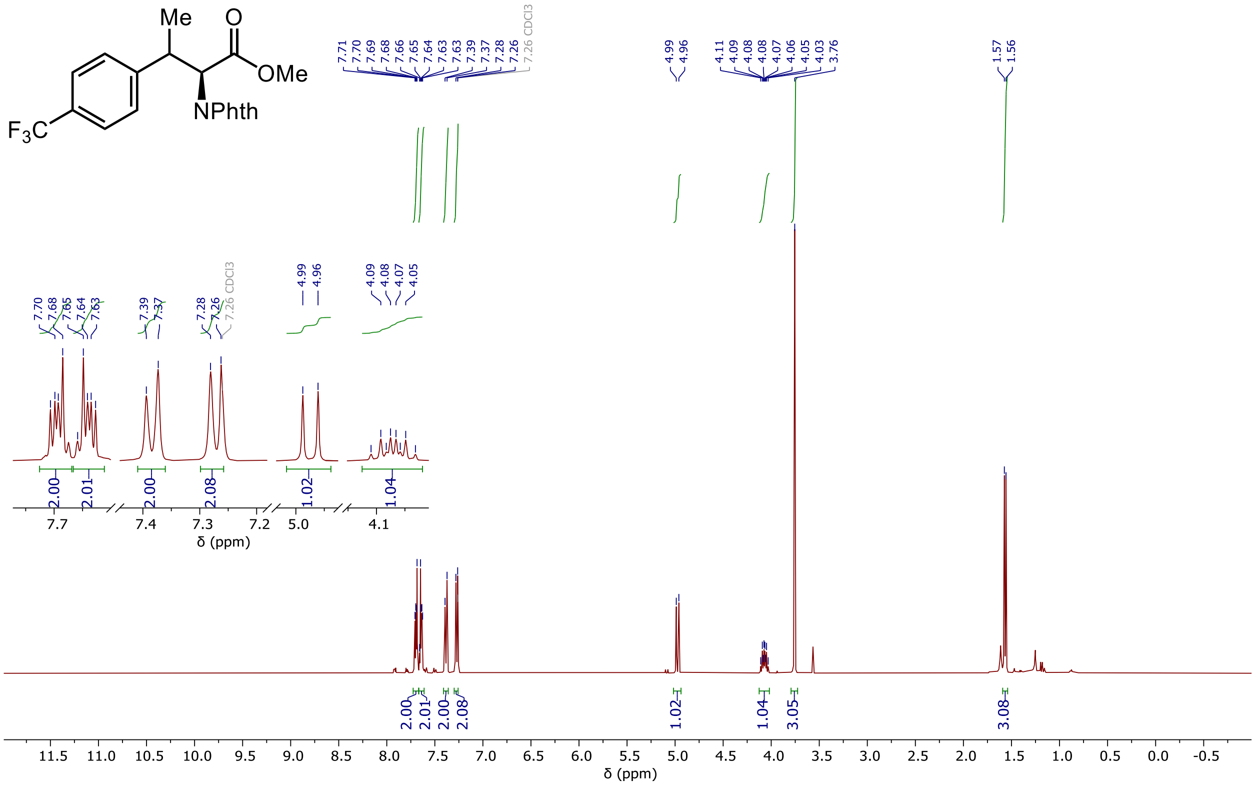


**Figure S101.** **$^{1}$H NMR spectra of (23a) (400 MHz, $CDCl_3$).**

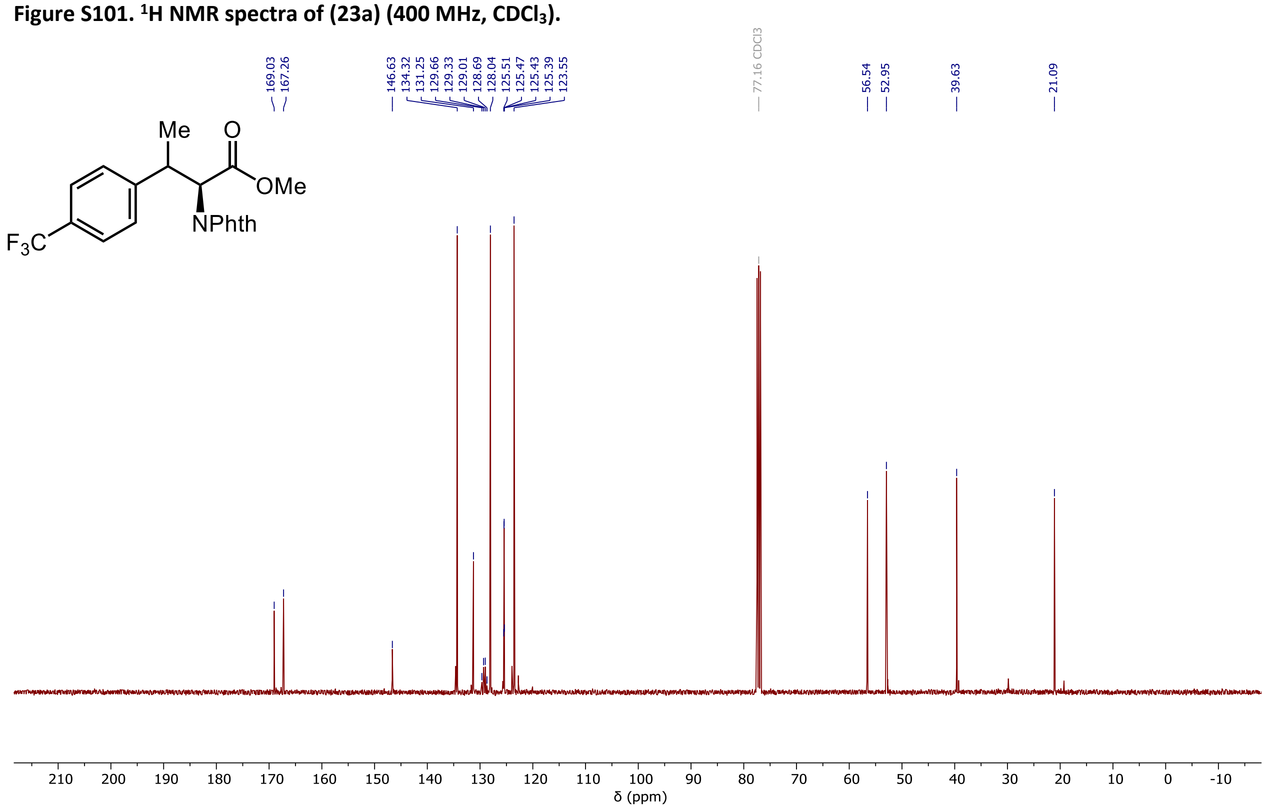


**Figure S102.** **$^{13}$C NMR spectra of (23a) (101 MHz, $CDCl_3$).**

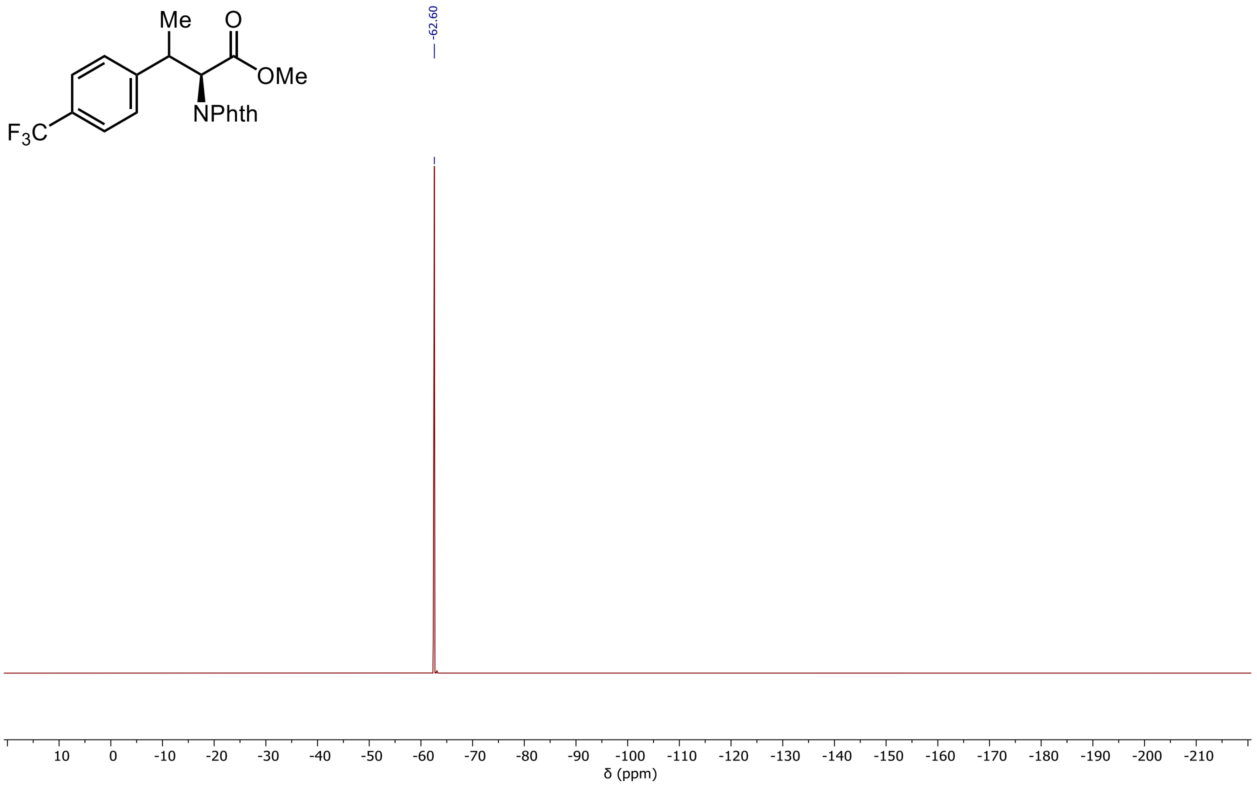


**Figure S103. $^{19}$F NMR spectra of (23a) (376 MHz, $CDCl_3$).**

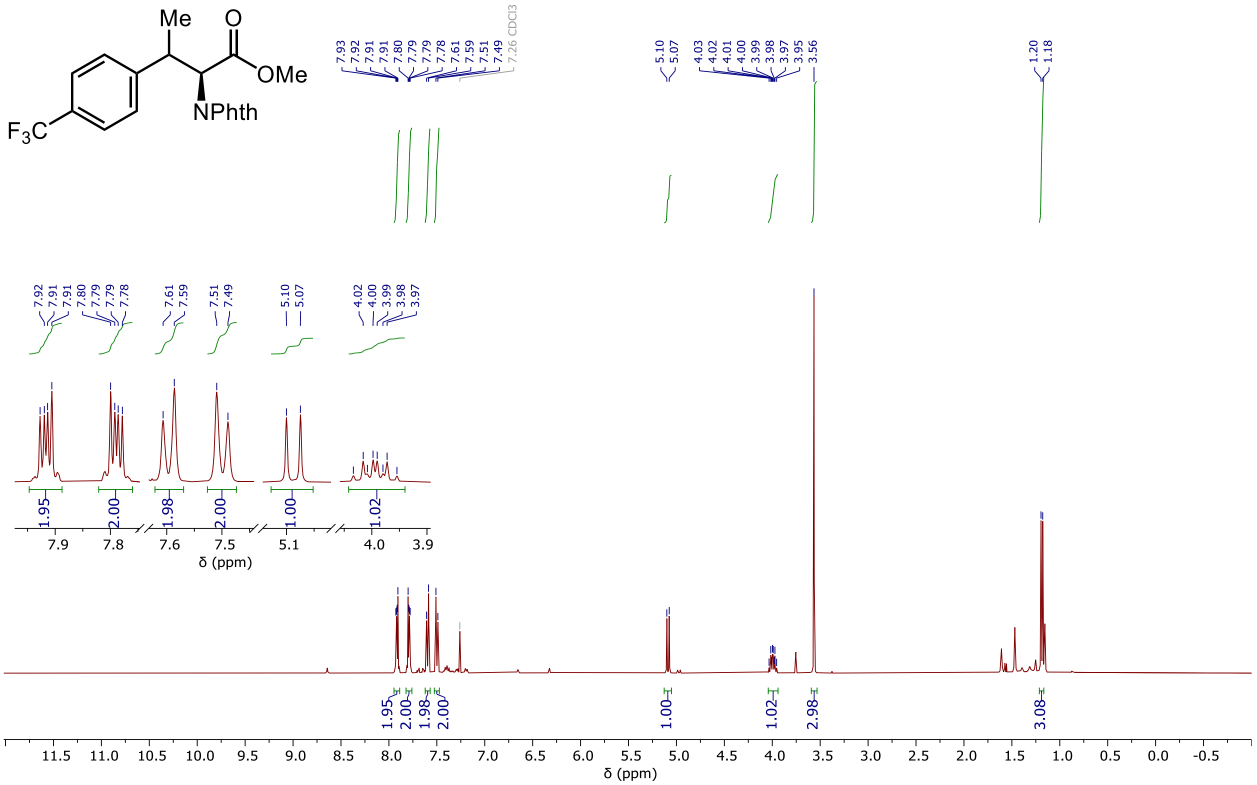


**Figure S104. $^{1}$H NMR spectra of (23b) (400 MHz, $CDCl_3$).**

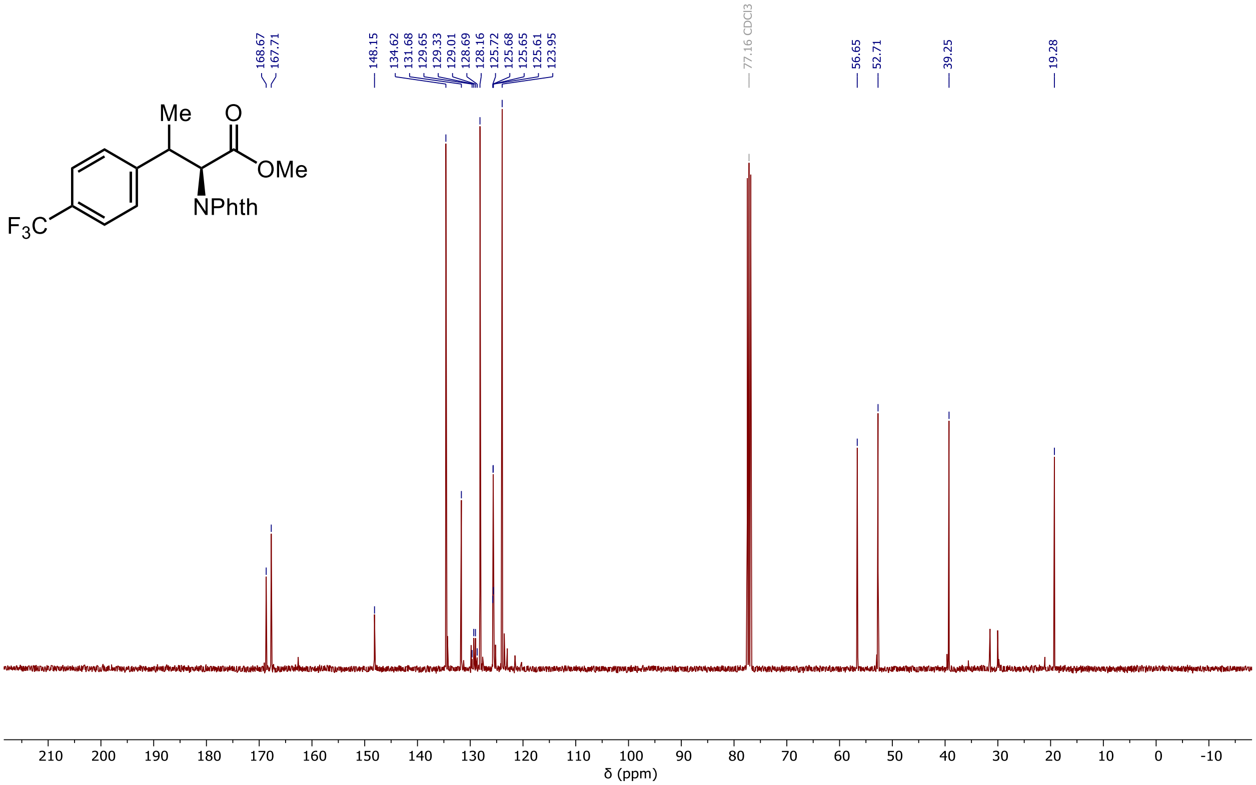


**Figure S105. $^{13}$C NMR spectra of (23b) (101 MHz, $CDCl_3$).**

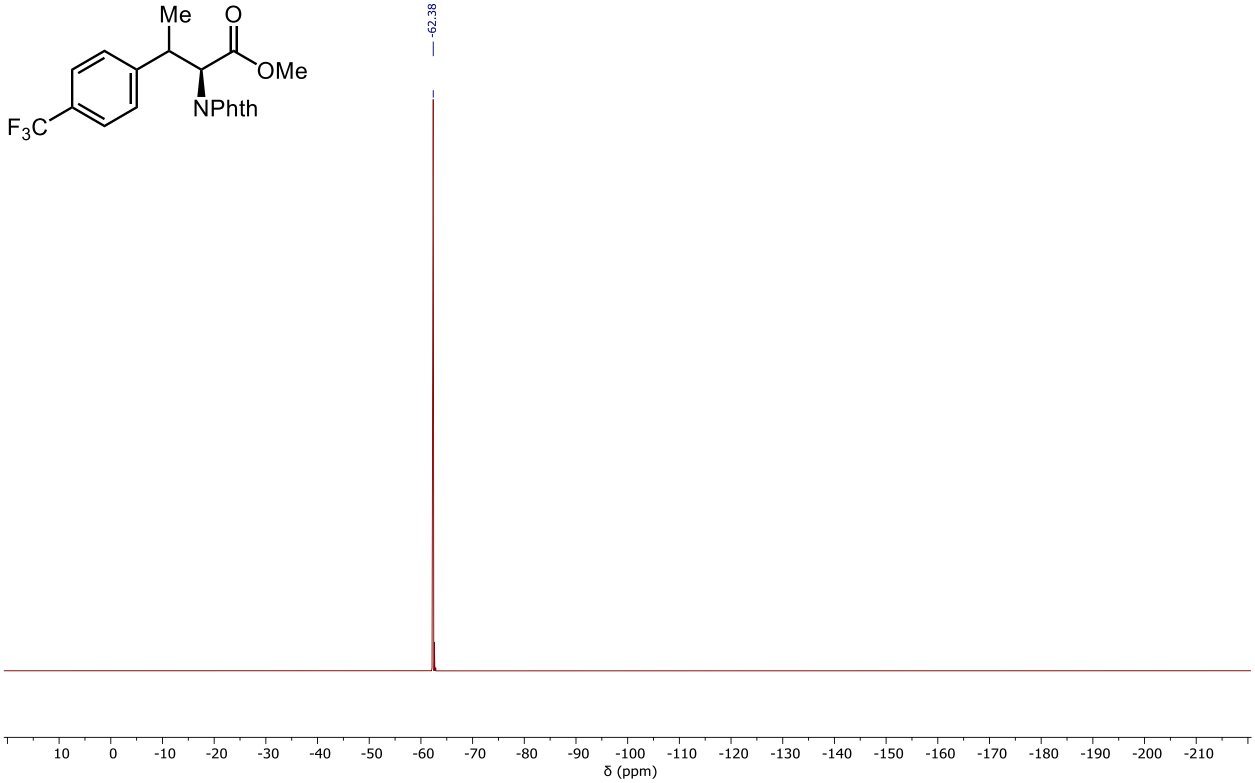


**Figure S106. $^{19}$F NMR spectra of (23b) (376 MHz, $CDCl_3$).**

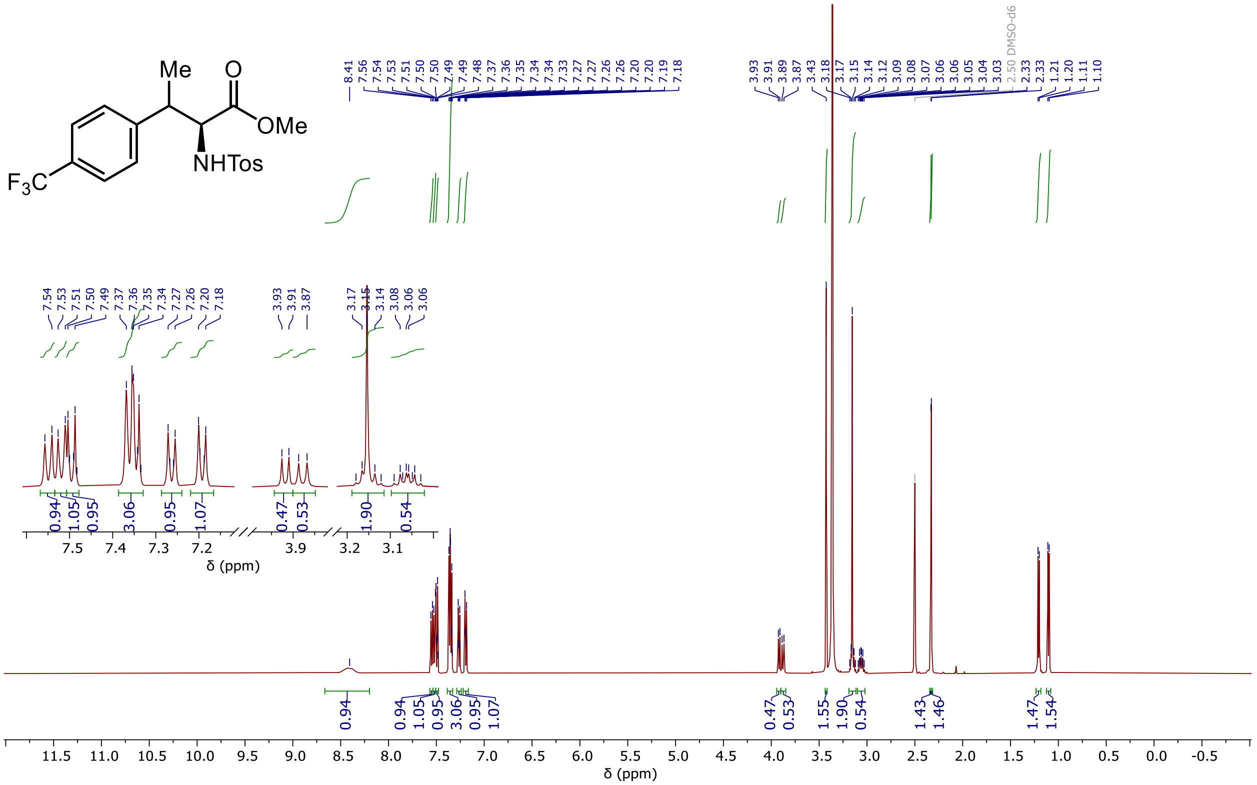


**Figure S107.** $^{1}H$ **NMR spectra of (24) (500 MHz, DMSO-** ***d*** $_{6}$ **).**

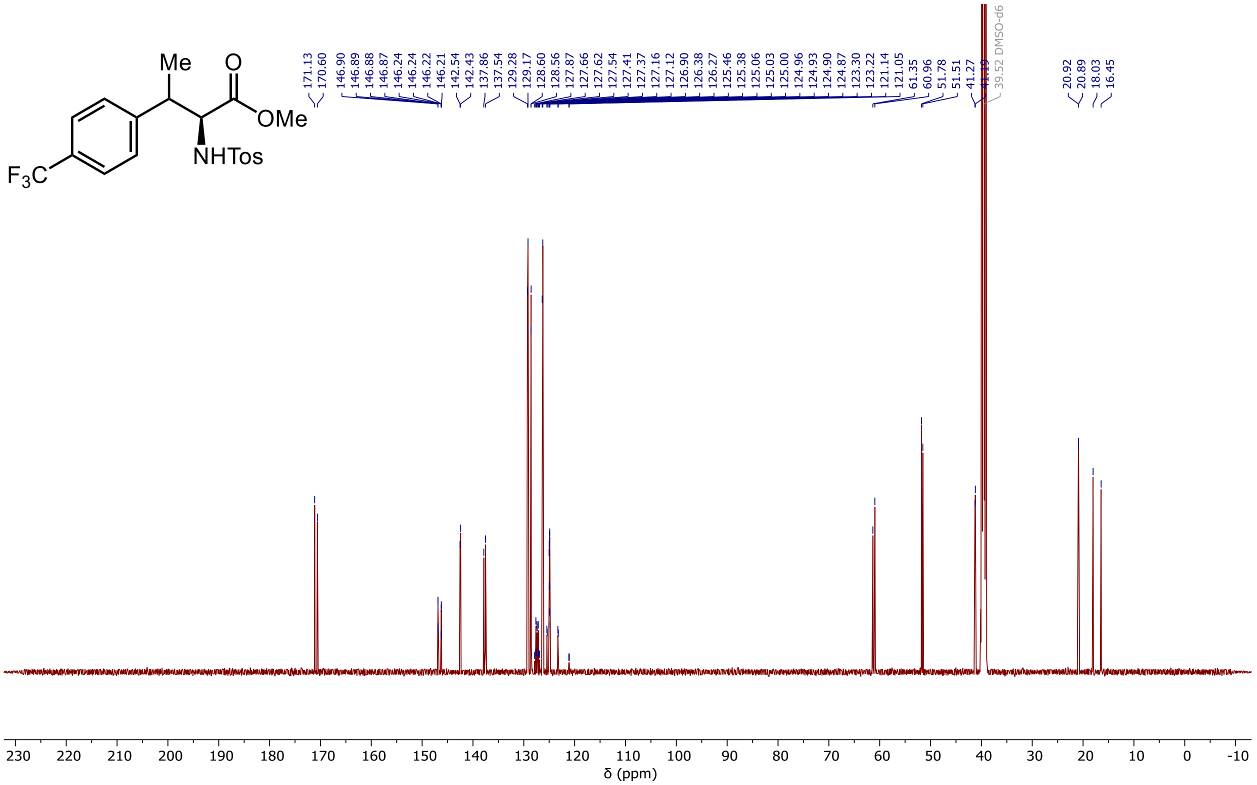


**Figure S108.** $^{13}C$ **NMR spectra of (24) (126 MHz, DMSO-** ***d*** $_{6}$ **).**

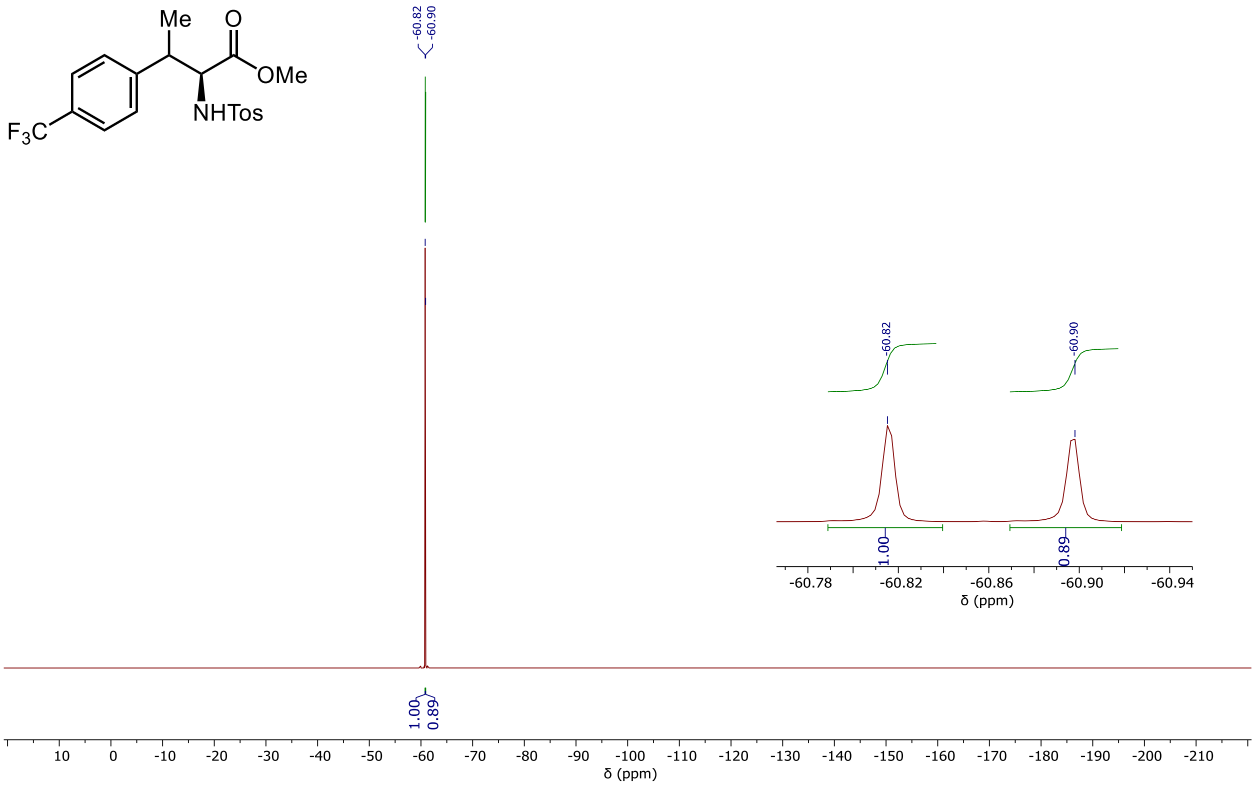

**Figure S109. $^{19}$F NMR spectra of (24) (470 MHz, DMSO-$d_6$).**

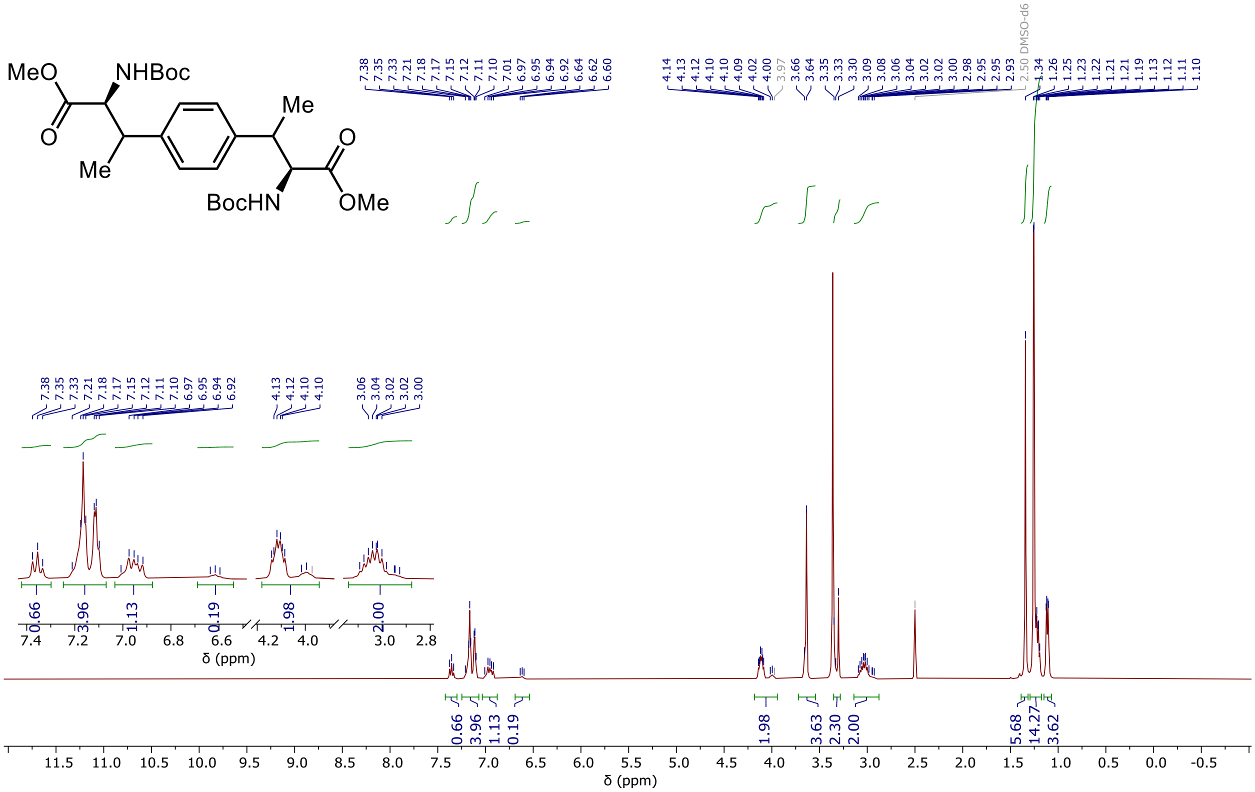

**Figure S110. $^{1}$H NMR spectra of (25) (400 MHz, DMSO-$d_6$).**

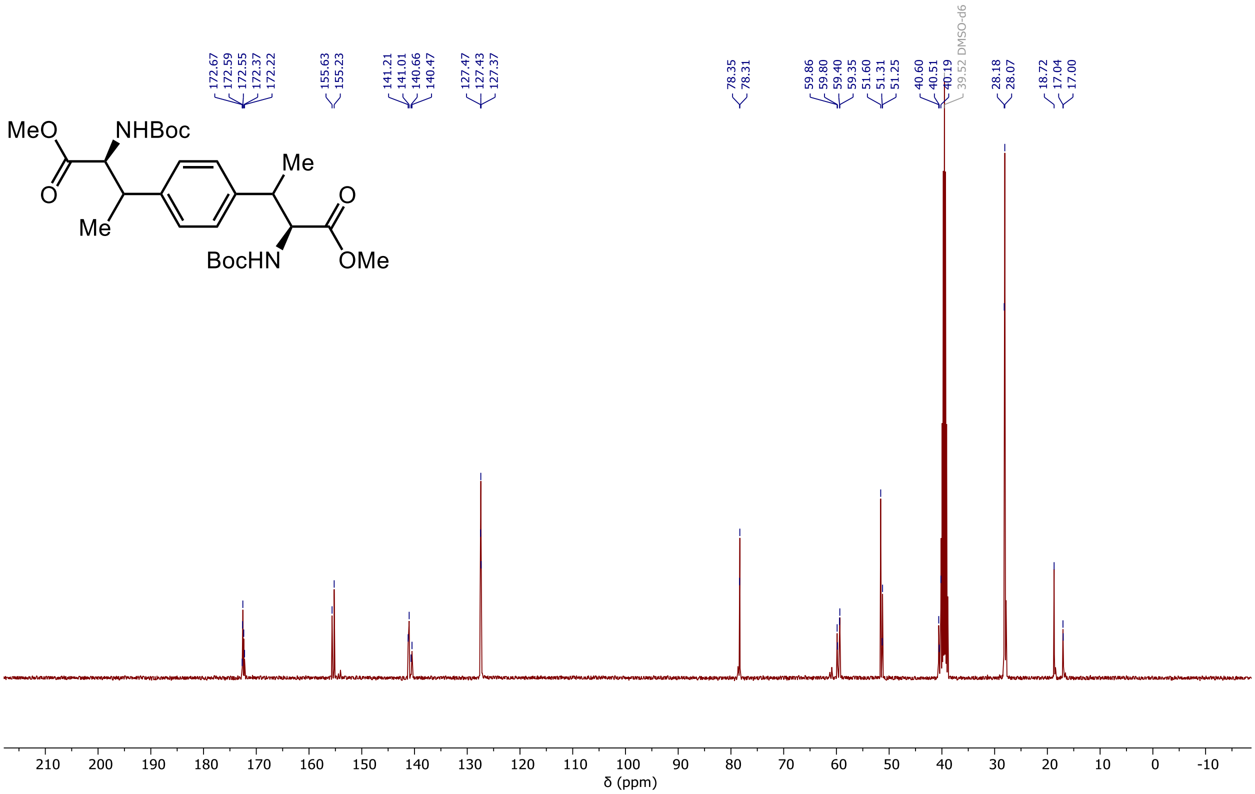

**Figure S111.** $^{13}$**C NMR spectra of (25) (101 MHz, DMSO-d**$_6$**).**

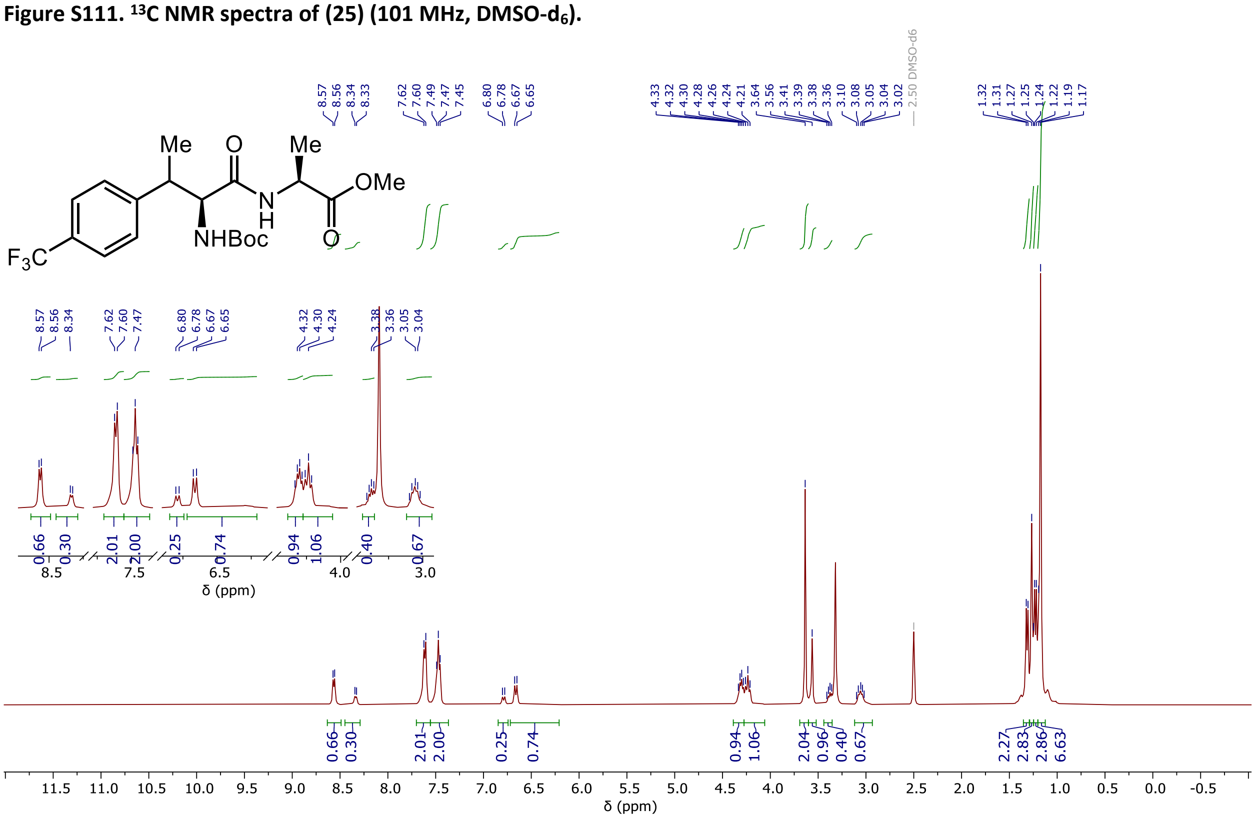

**Figure S112.** $^{1}$**H NMR spectra of (26) (400 MHz, DMSO-d**$_6$**).**

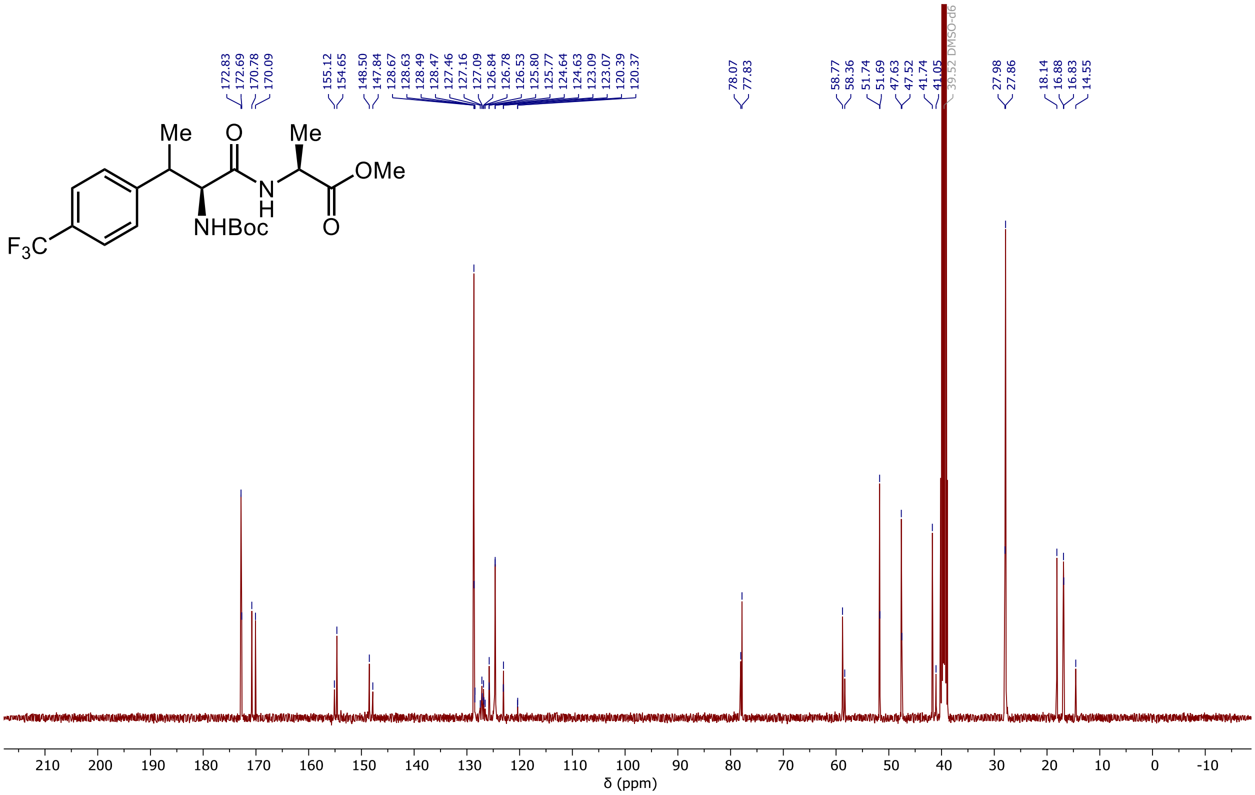


**Figure S113.** $^{13}$C **NMR spectra of (26) (101 MHz, DMSO-d$_6$).**

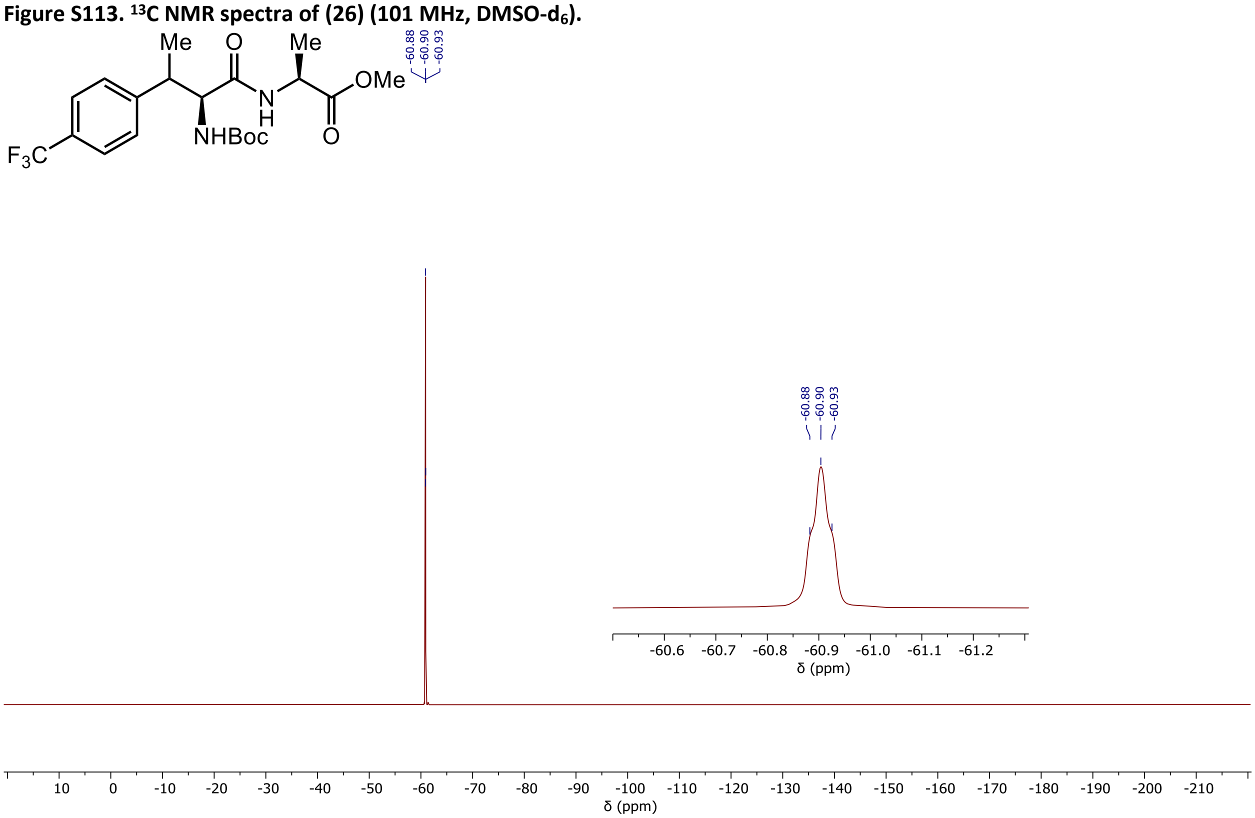


**Figure S114.** $^{19}$F **NMR spectra of (26) (376 MHz, DMSO-d$_6$).**

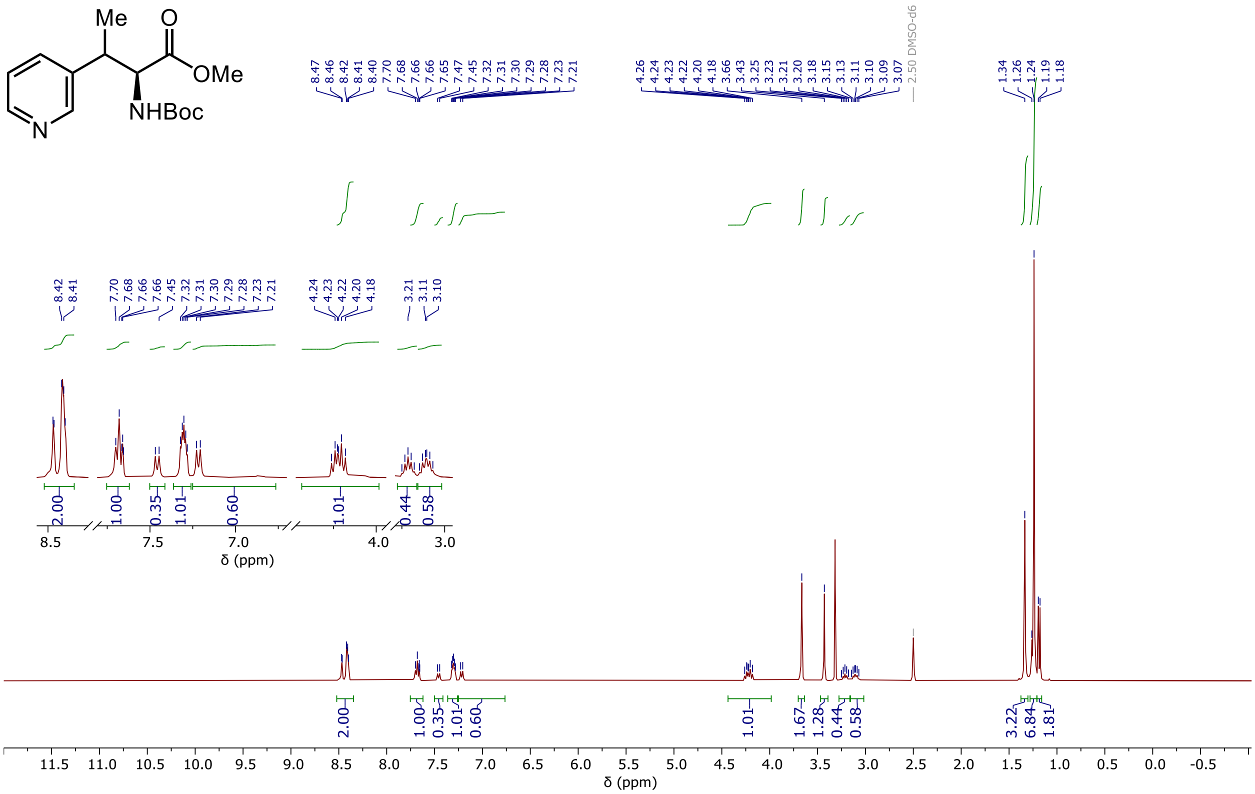

**Figure S115. $^{1}$H NMR spectra of (27) (400 MHz, DMSO-$d_6$).**

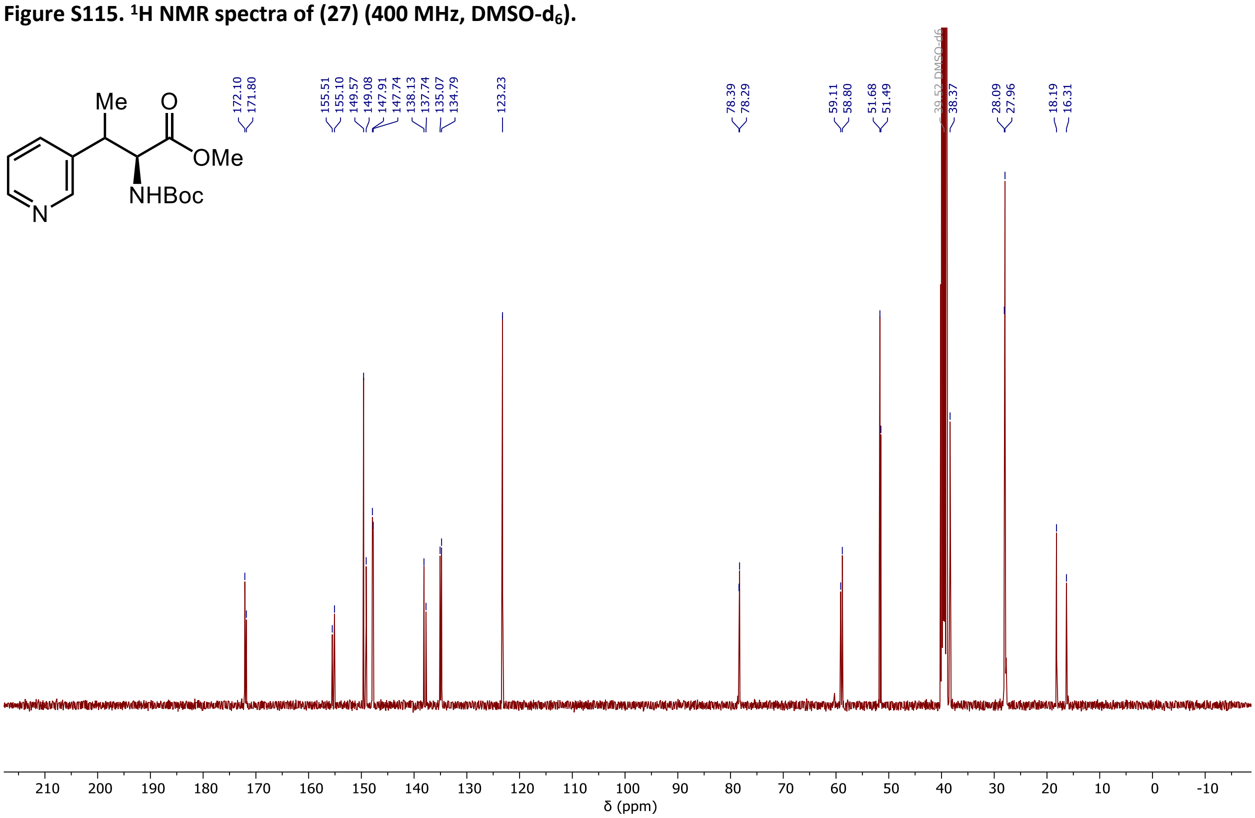

**Figure S116. $^{13}$C NMR spectra of (27) (101 MHz, DMSO-$d_6$).**

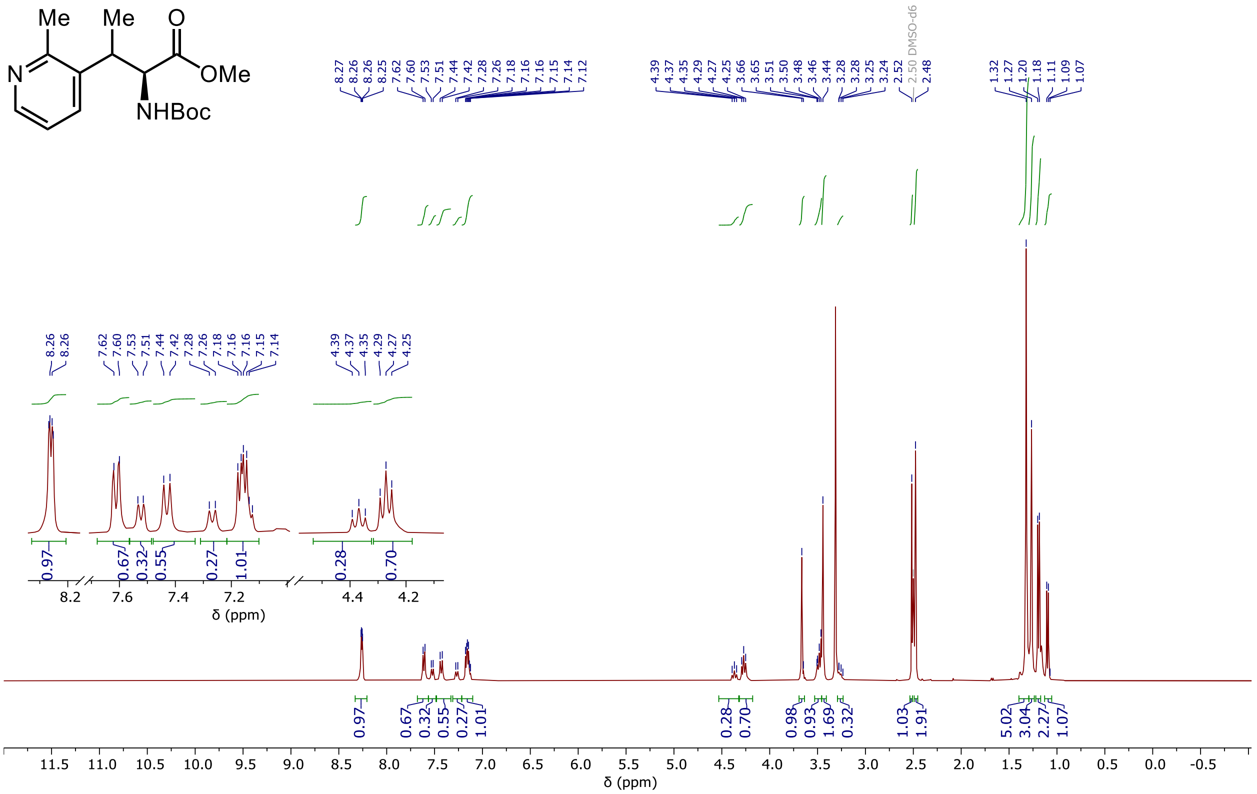


**Figure S117.** **$^{1}$H NMR spectra of (28) (400 MHz, DMSO-d$_6$).**

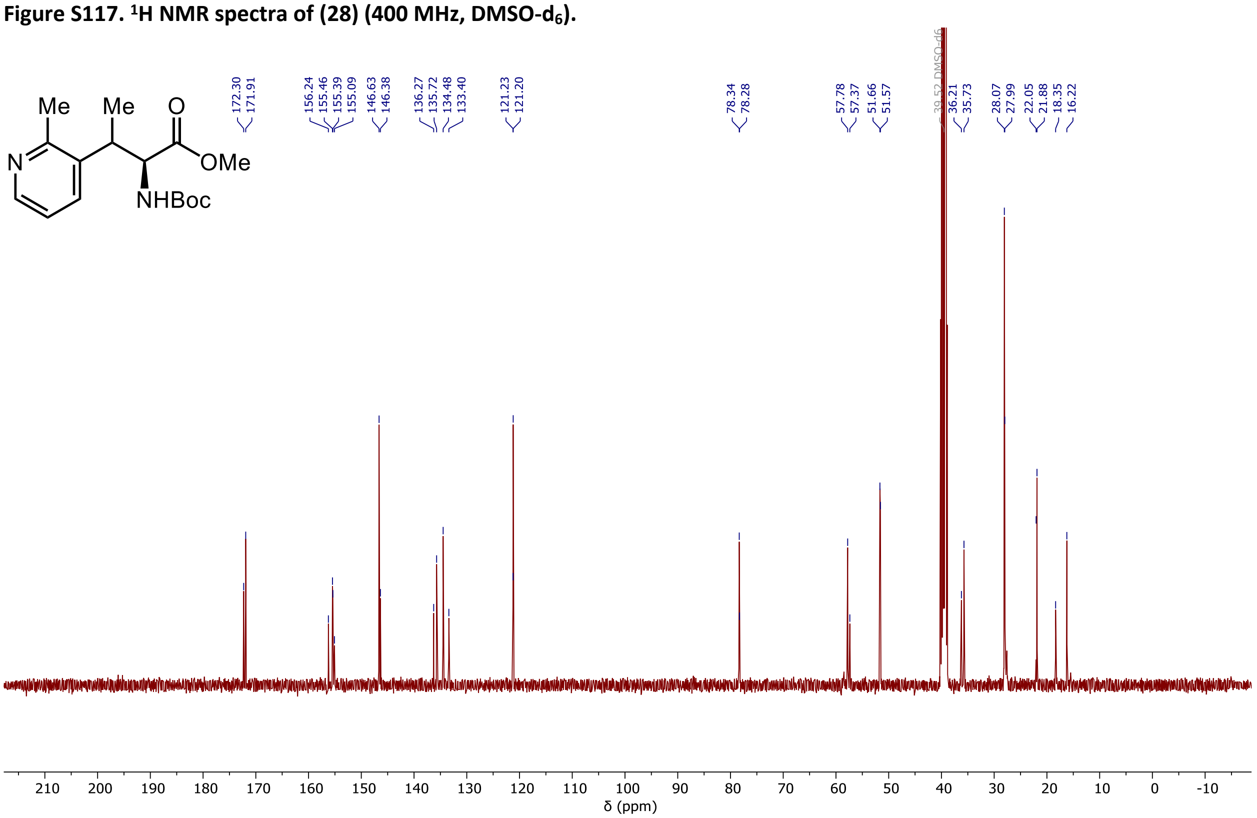


**Figure S118.** **$^{13}$C NMR spectra of (28) (101 MHz, DMSO-d$_6$).**

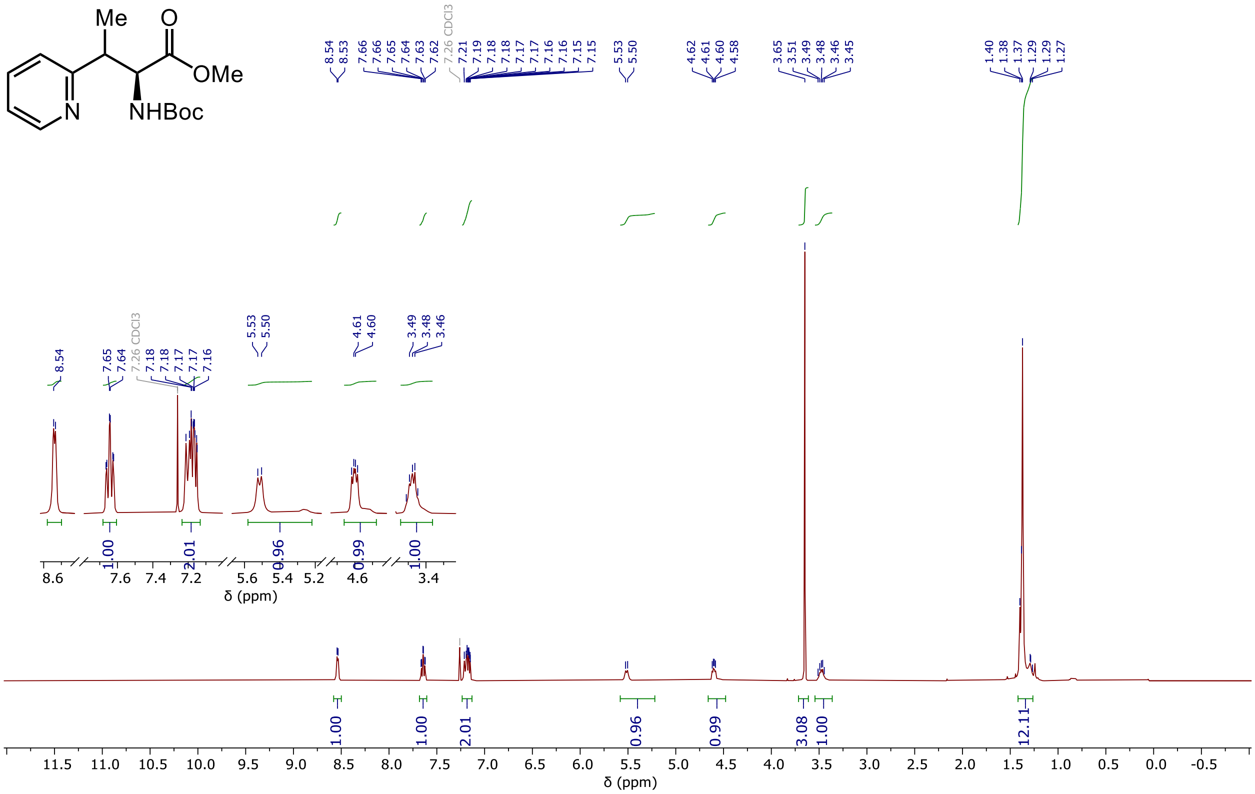

**Figure S119. $^{1}$H NMR spectra of (29a) (400 MHz, $CDCl_3$).**

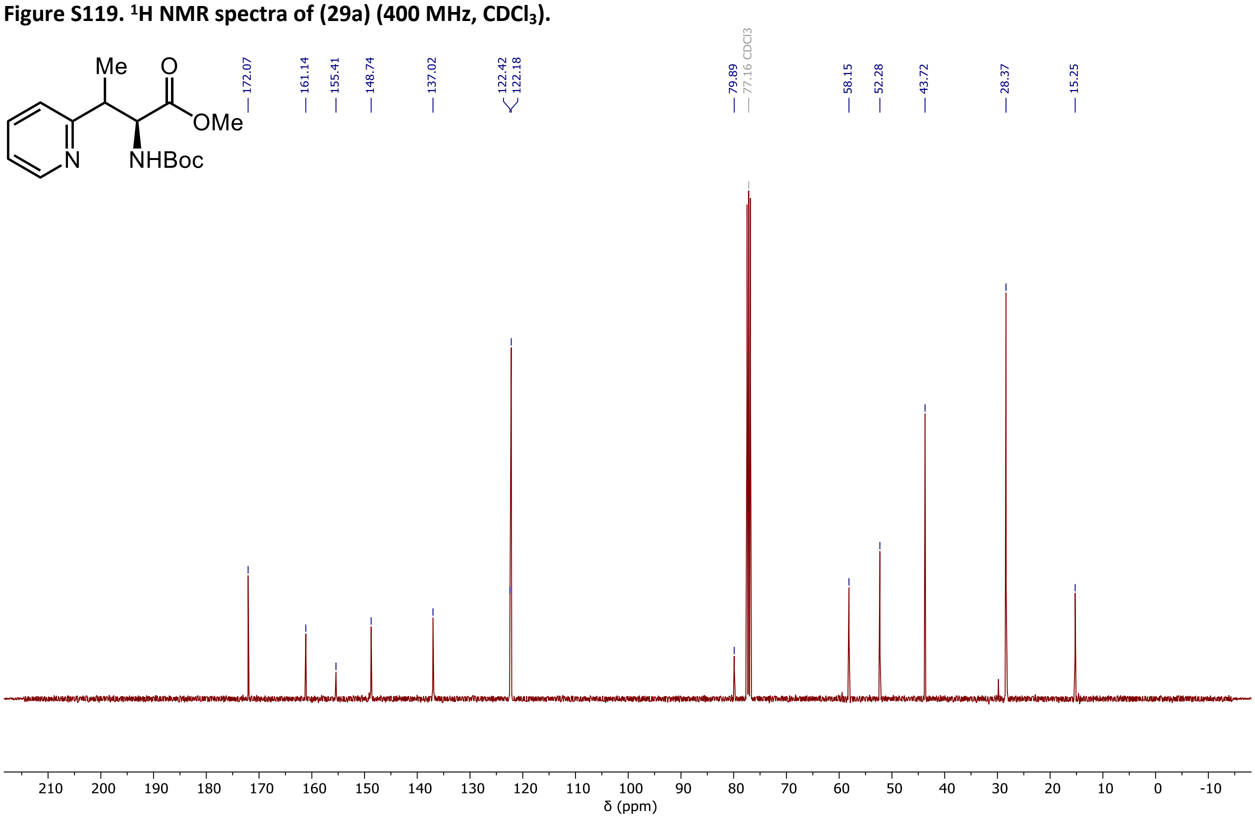

**Figure S120. $^{13}$C NMR spectra of (29a) (101 MHz, $CDCl_3$).**

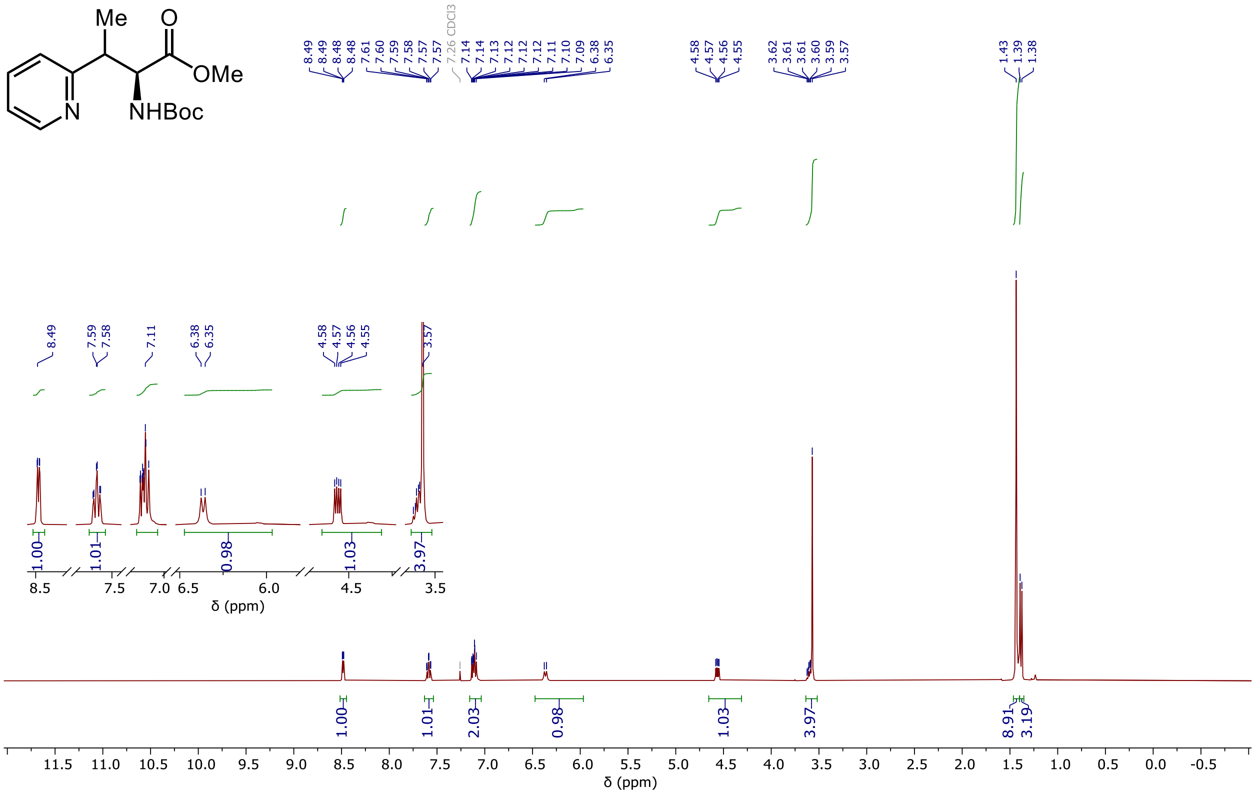


**Figure S121.** $^{1}$**H NMR spectra of (29b) (400 MHz, $CDCl_3$).**

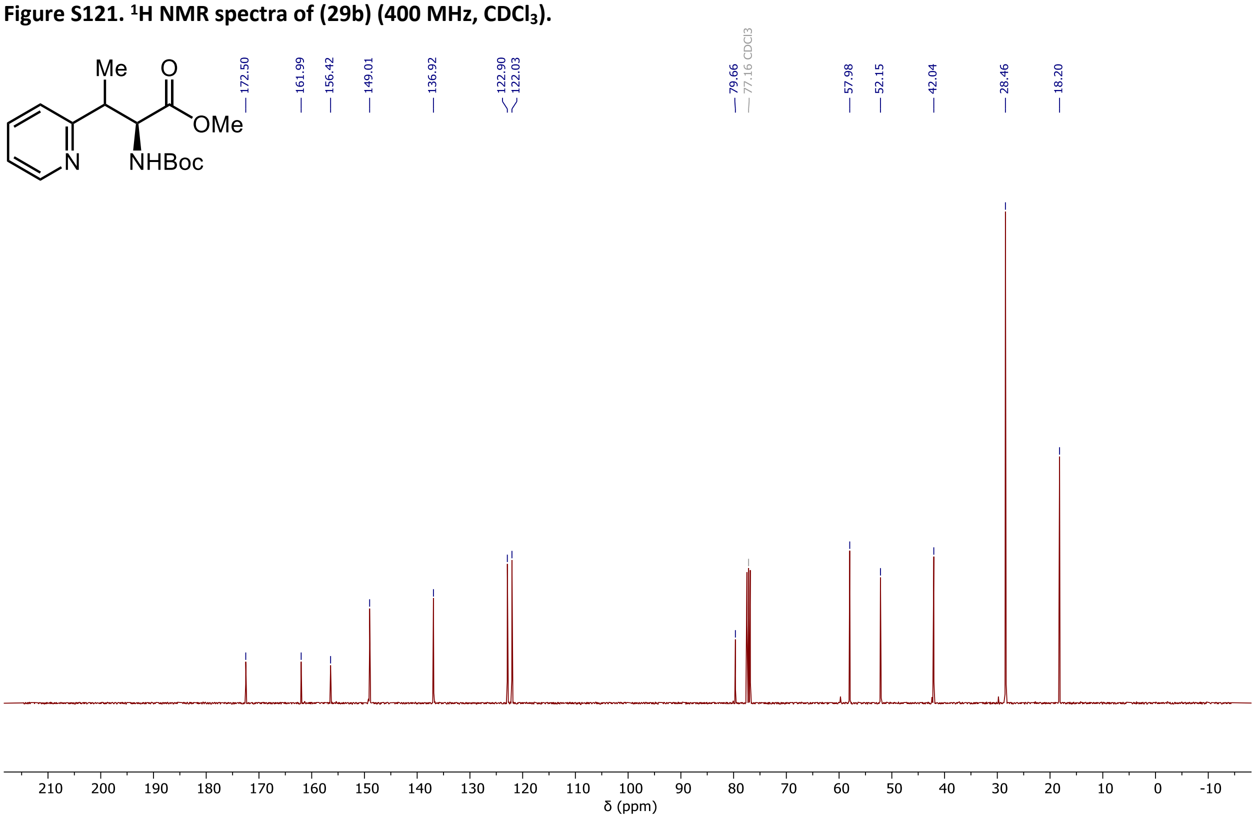


**Figure S122.** $^{13}$**C NMR spectra of (29b) (101 MHz, $CDCl_3$).**

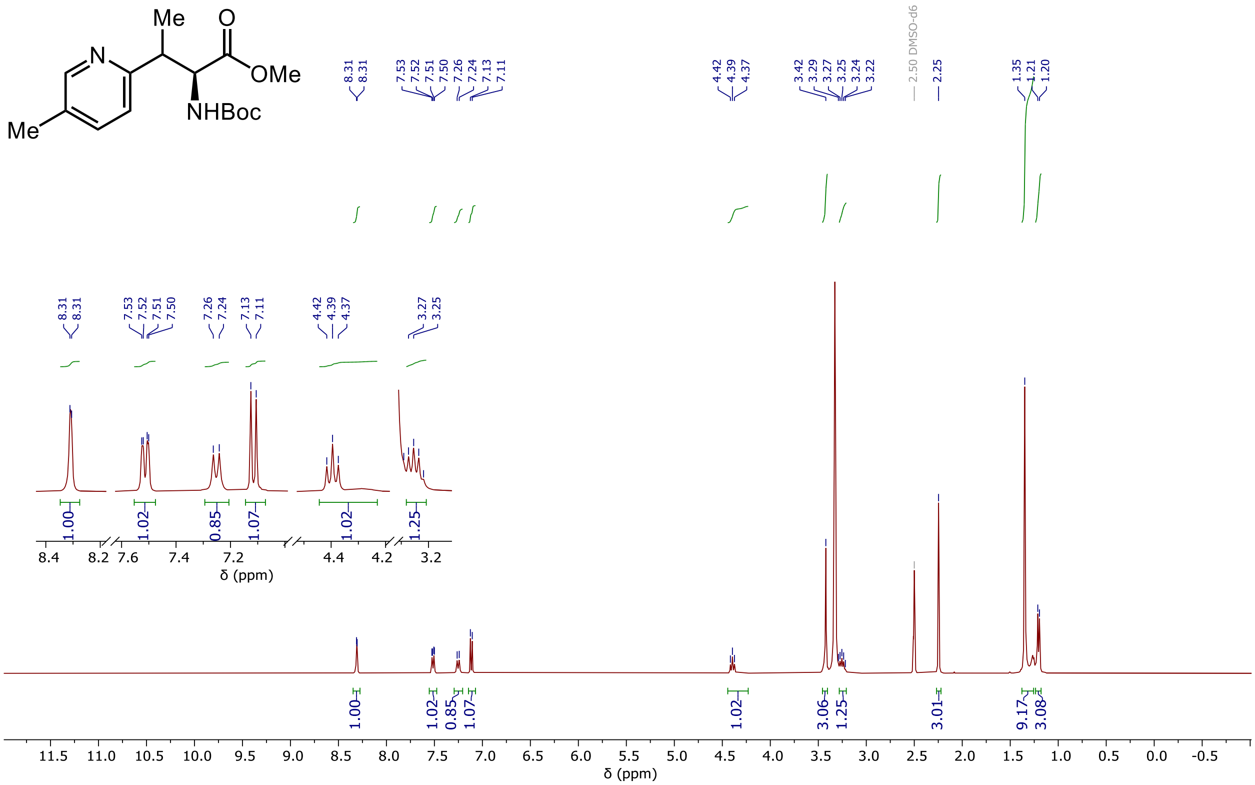

**Figure S123. $^{1}H$ NMR spectra of (30a) (400 MHz, DMSO-d$_6$).**

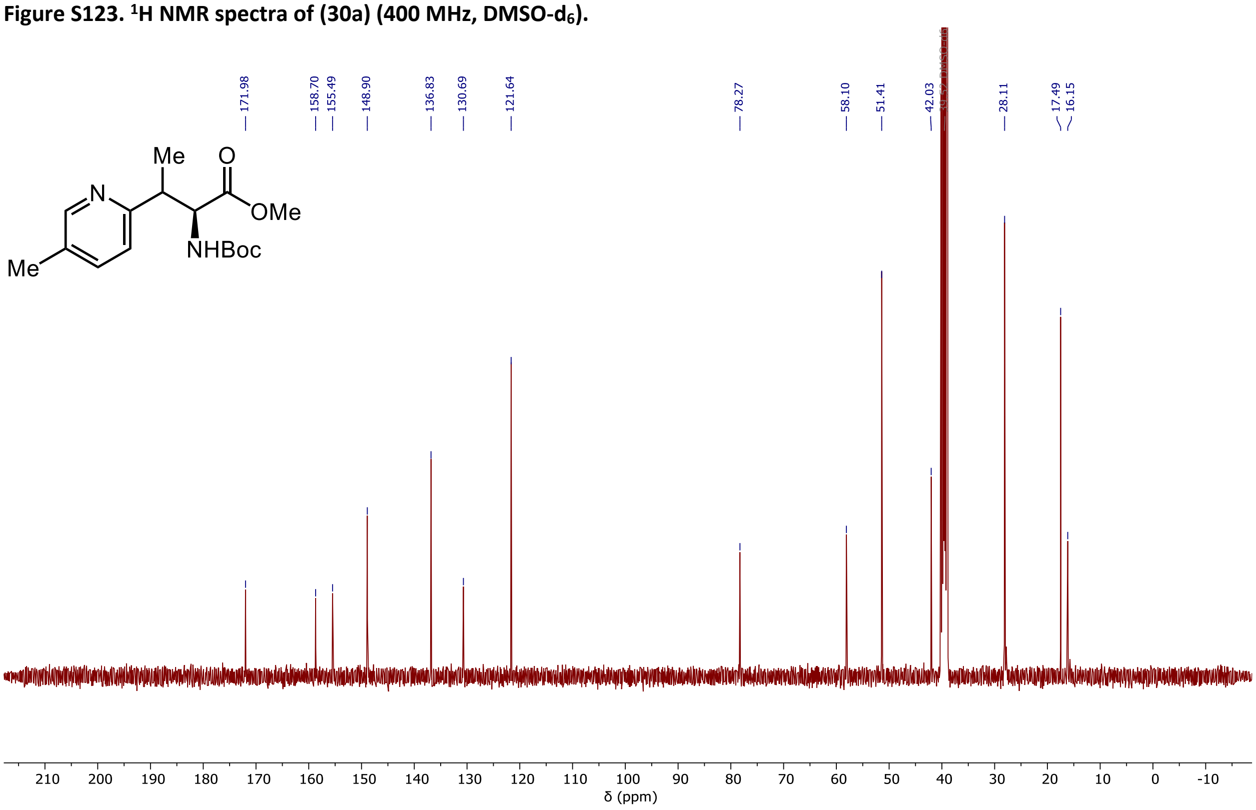

**Figure S124. $^{13}C$ NMR spectra of (30a) (101 MHz, DMSO-d$_6$).**

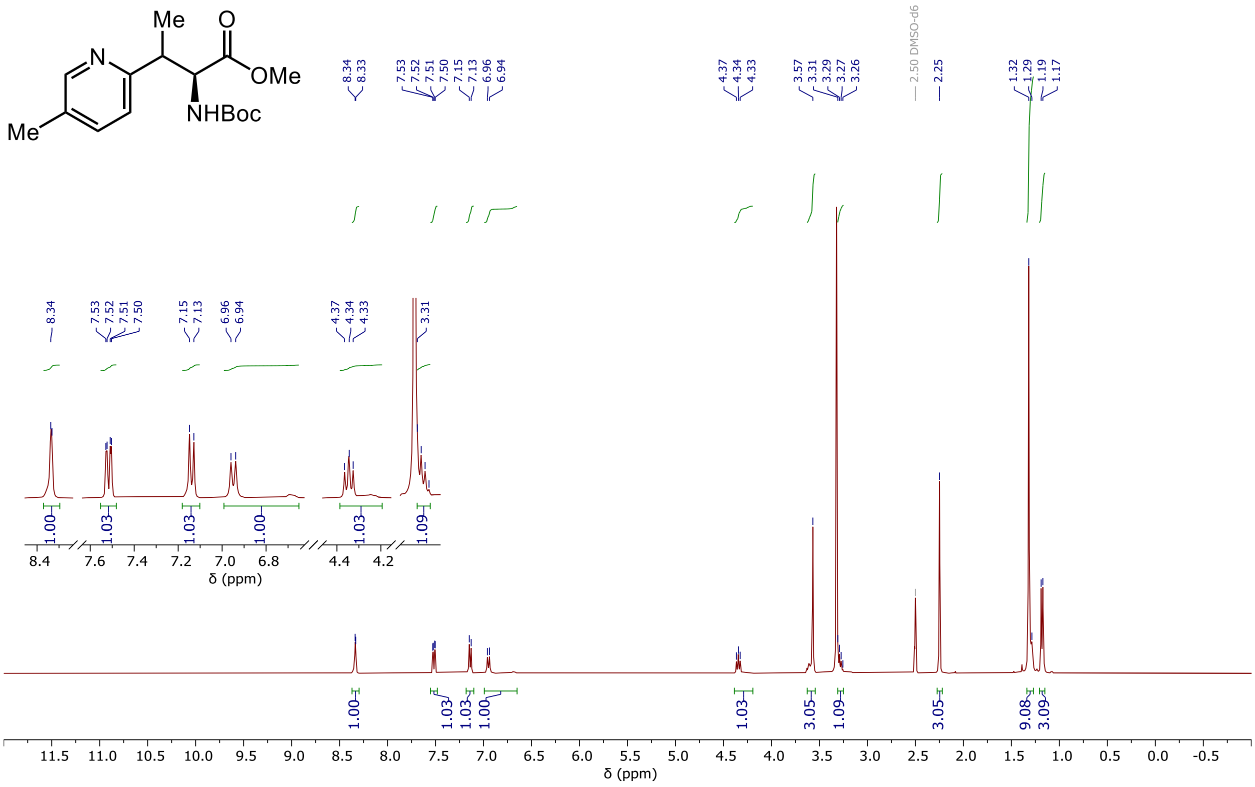

**Figure S125. $^{1}$H NMR spectra of (30b) (400 MHz, DMSO-d$_6$).**

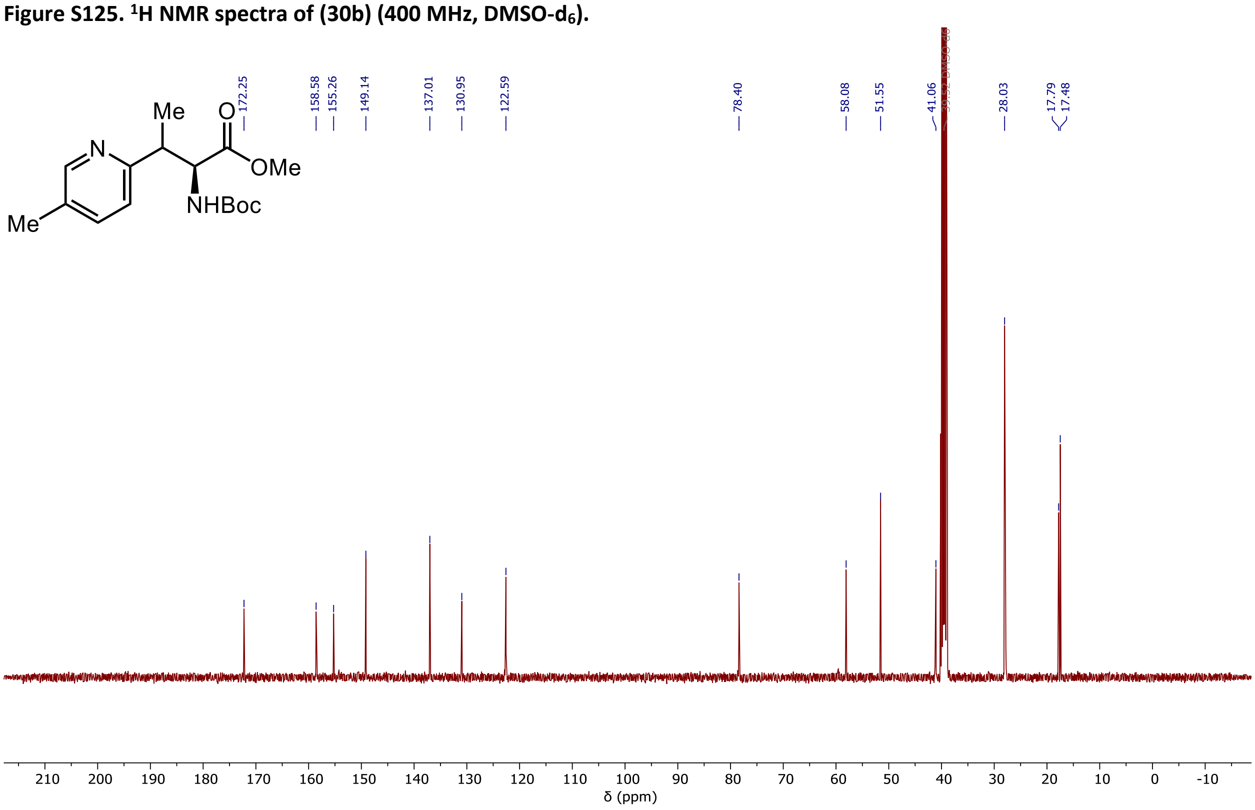

**Figure S126. $^{13}$C NMR spectra of (30b) (101 MHz, DMSO-d$_6$).**

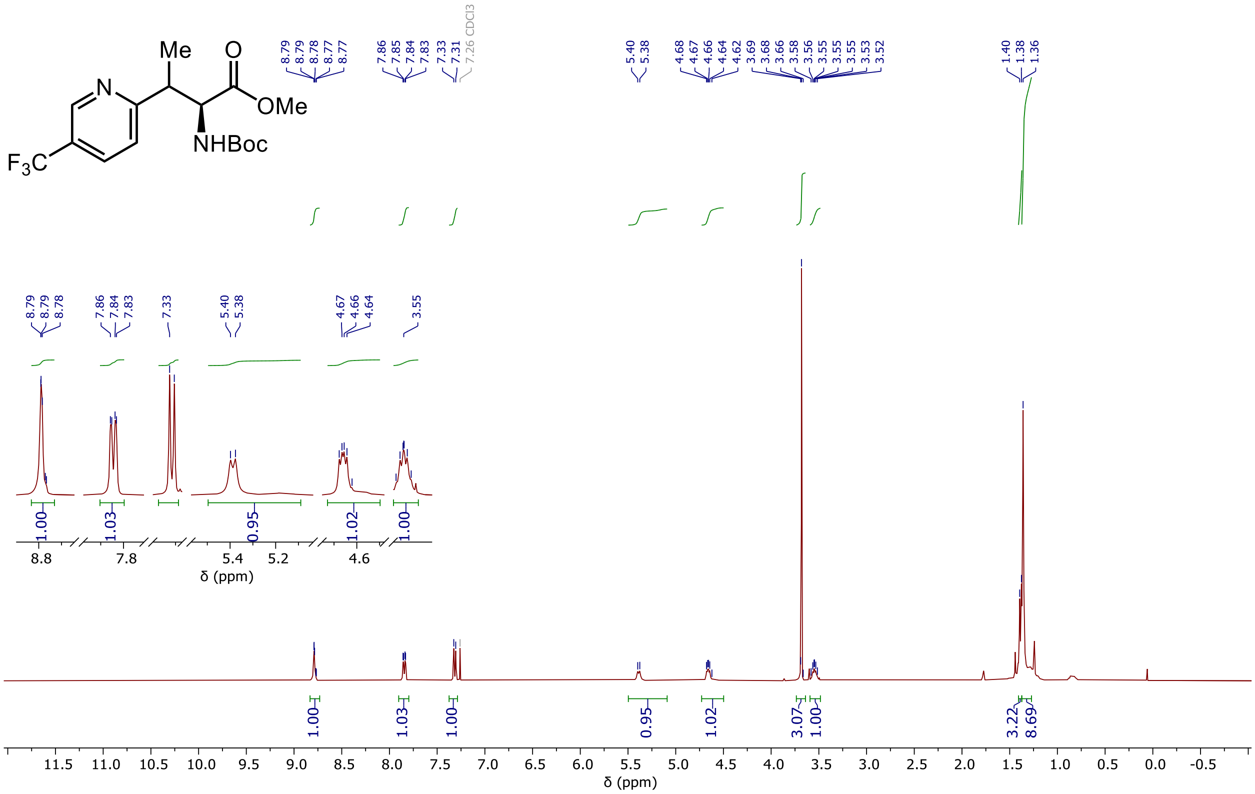

**Figure S127.** **$^{1}H$ NMR spectra of (31a) (400 MHz, $CDCl_3$).**

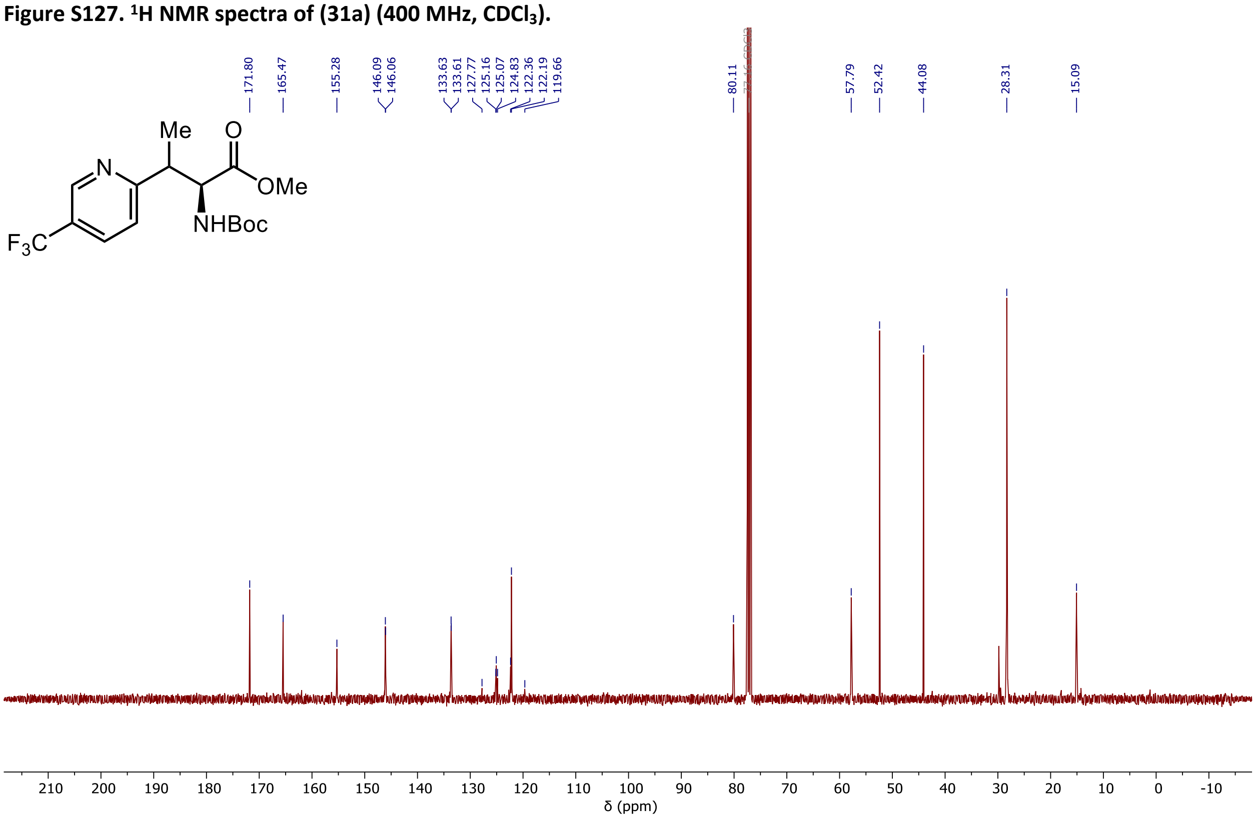

**Figure S128.** **$^{13}C$ NMR spectra of (31a) (101 MHz, $CDCl_3$).**

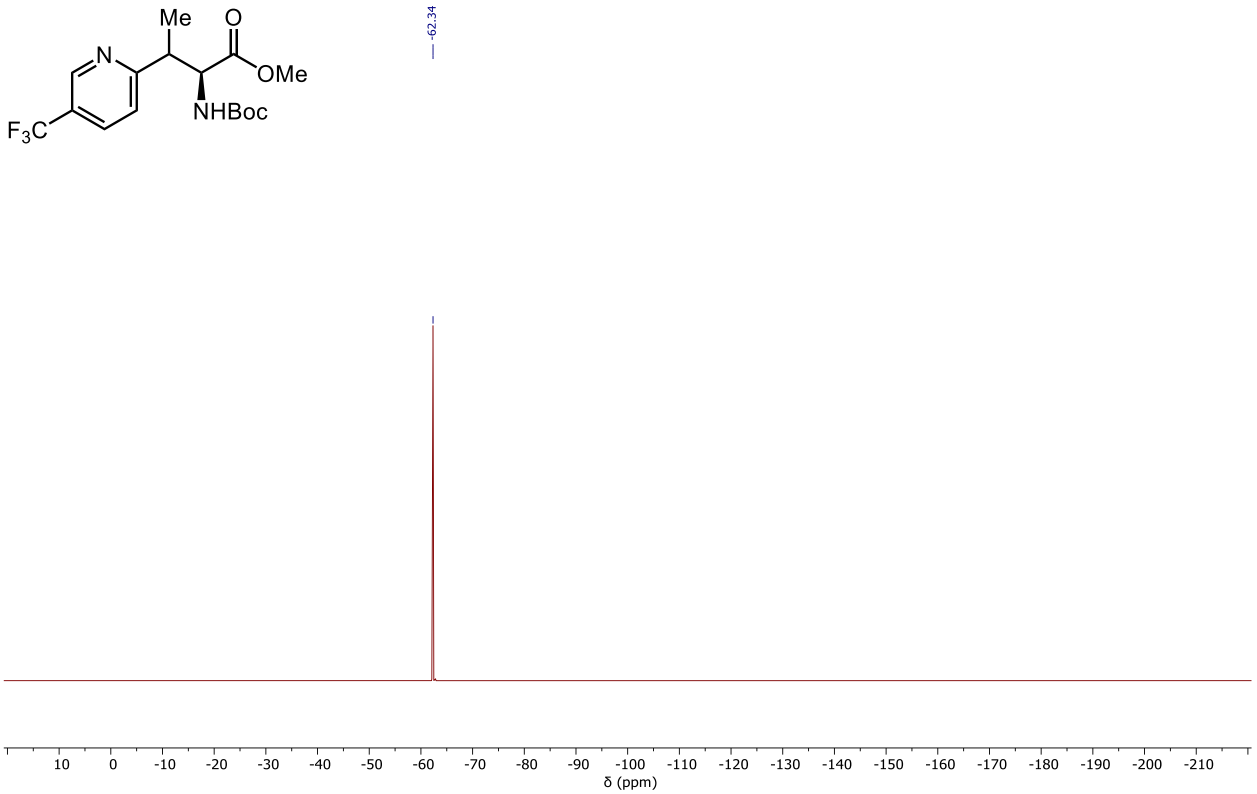

Figure S129. $^{19}F$ NMR spectra of (31a) (376 MHz, $CDCl_3$).

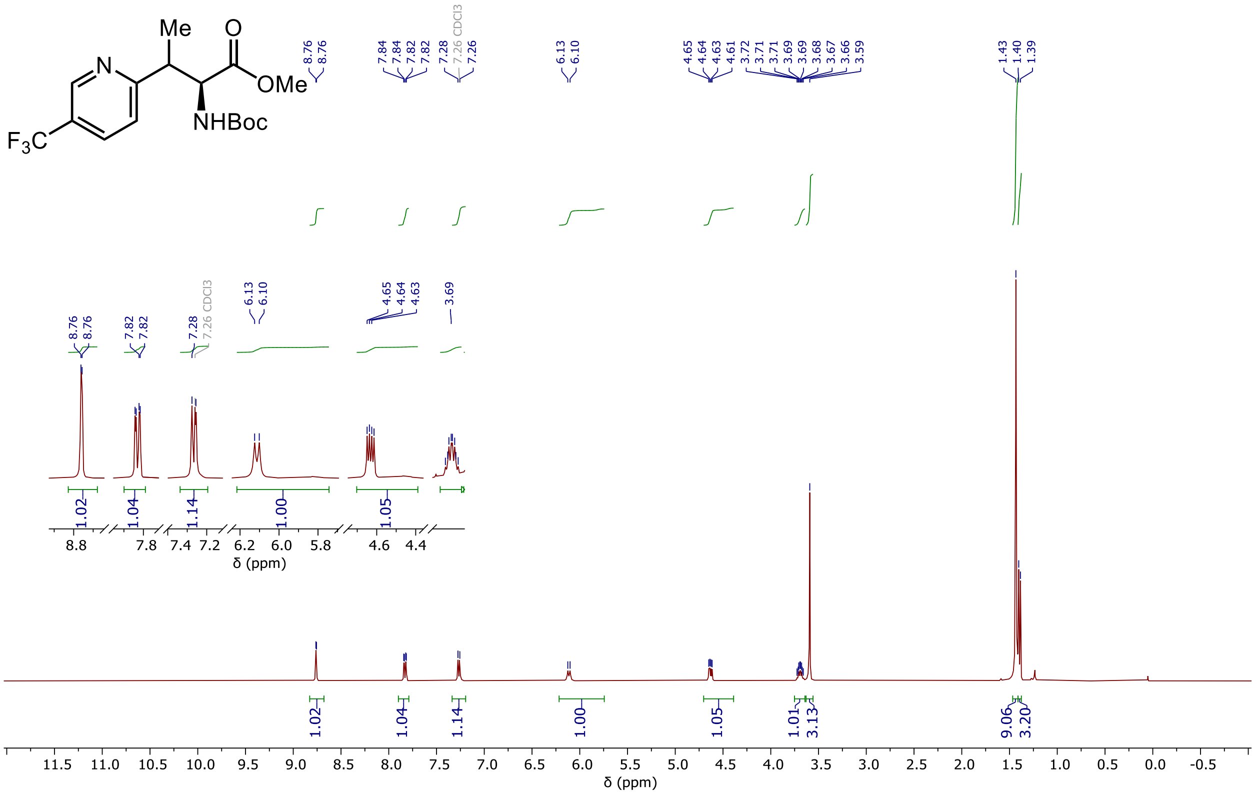

Figure S130. $^{1}H$ NMR spectra of (31b) (400 MHz, $CDCl_3$).

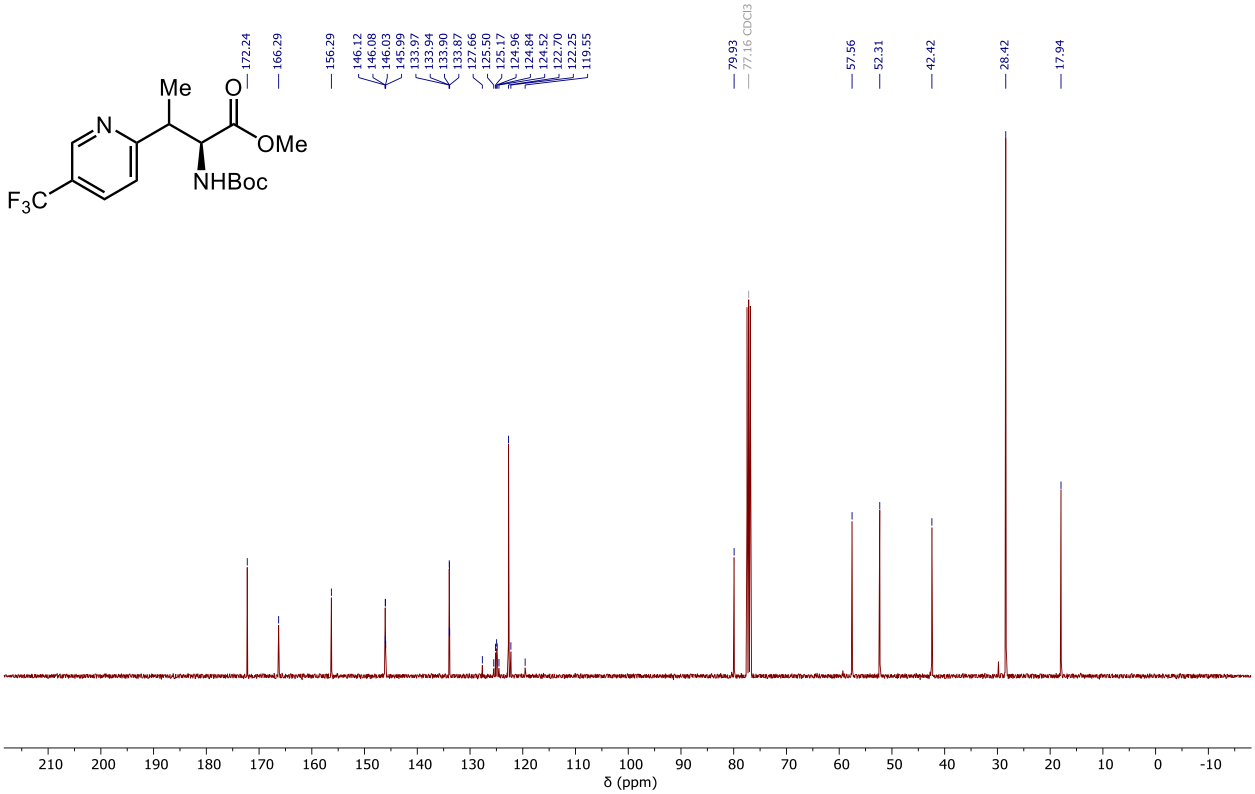


**Figure S131. $^{13}C$ NMR spectra of (31b) (101 MHz, $CDCl_3$).**

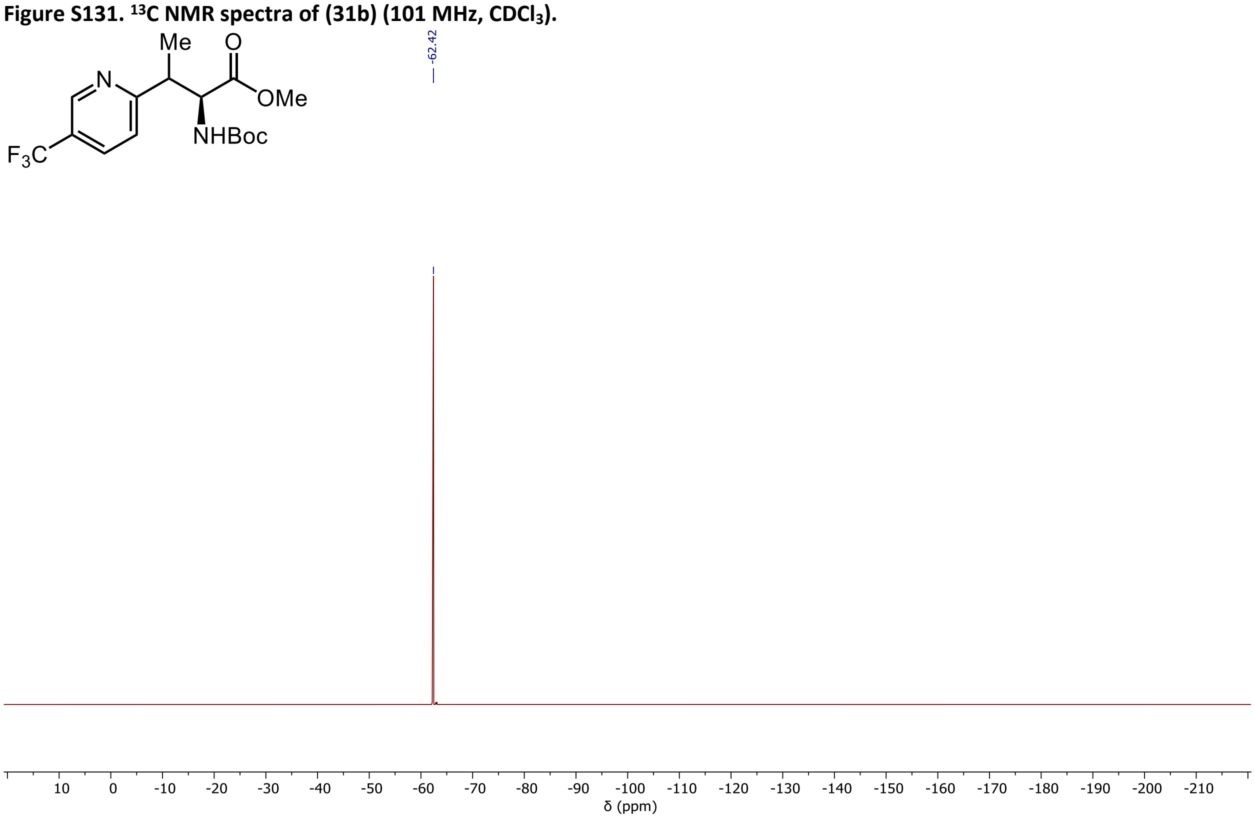


**Figure S132. $^{19}F$ NMR spectra of (31b) (376 MHz, $CDCl_3$).**

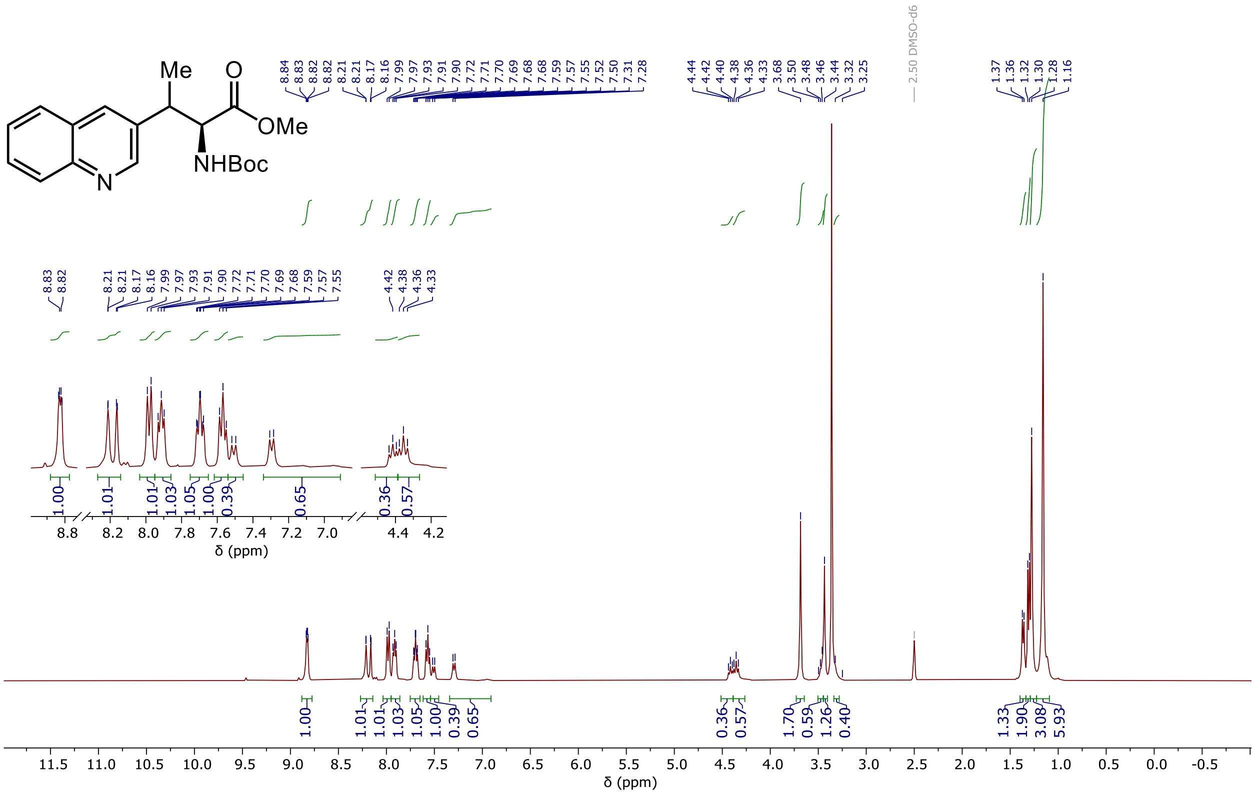


**Figure S133. $^{1}$H NMR spectra of (32) (400 MHz, DMSO-d$_6$).**

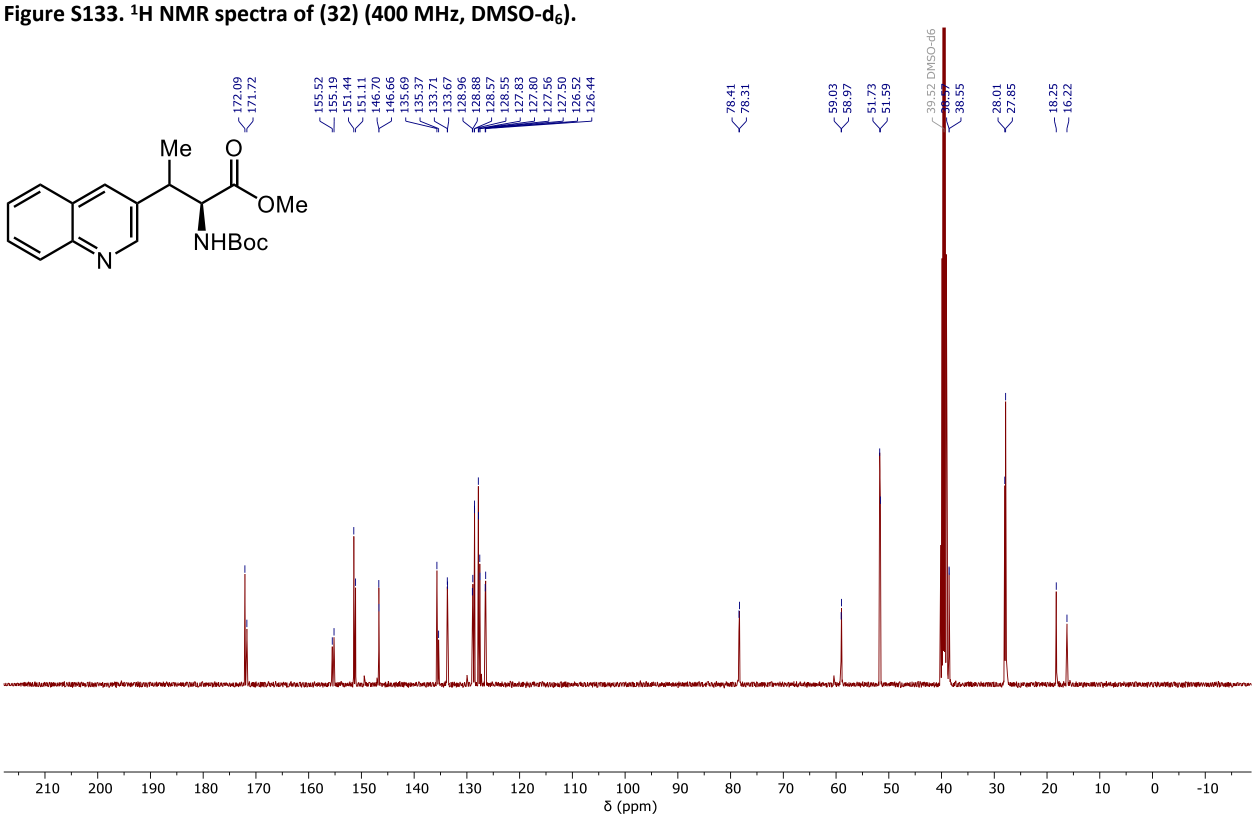


**Figure S134. $^{13}$C NMR spectra of (32) (101 MHz, DMSO-d$_6$).**

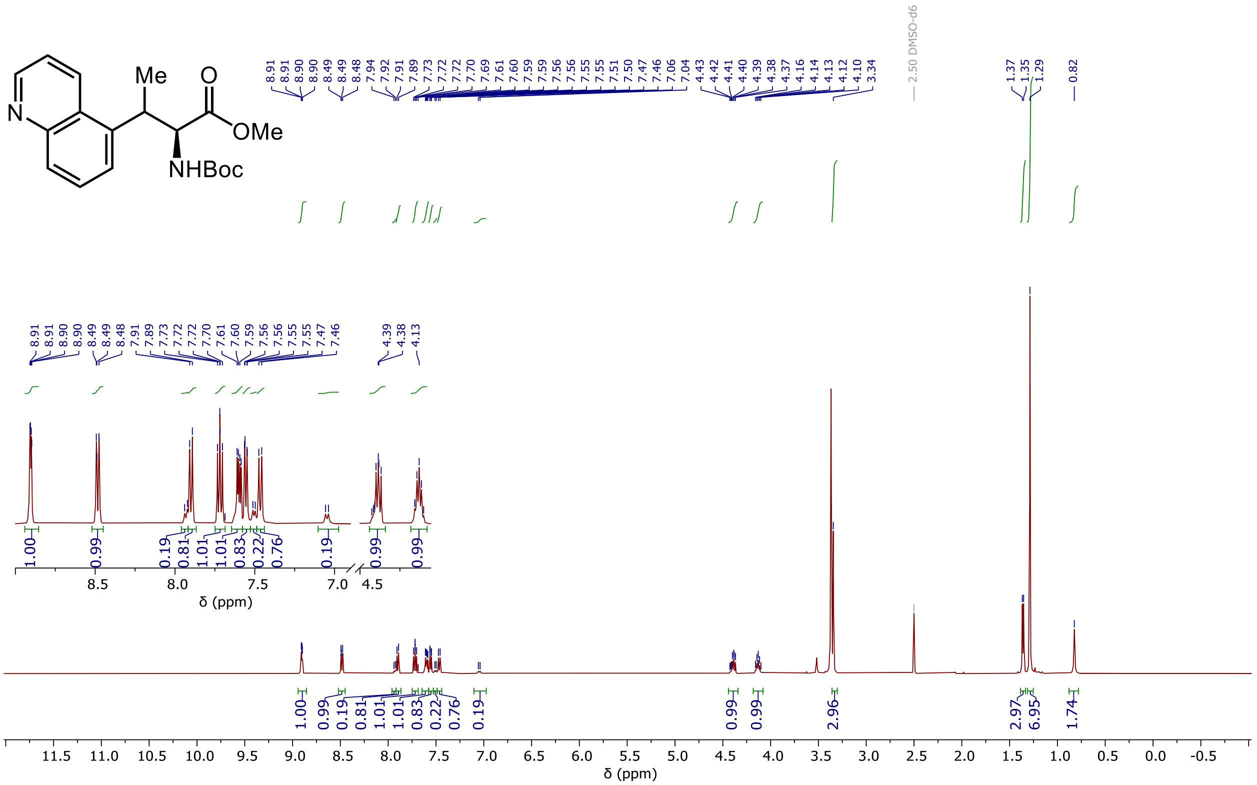

**Figure S135. $^{1}$H NMR spectra of (33a) (500 MHz, DMSO-$d_6$).**

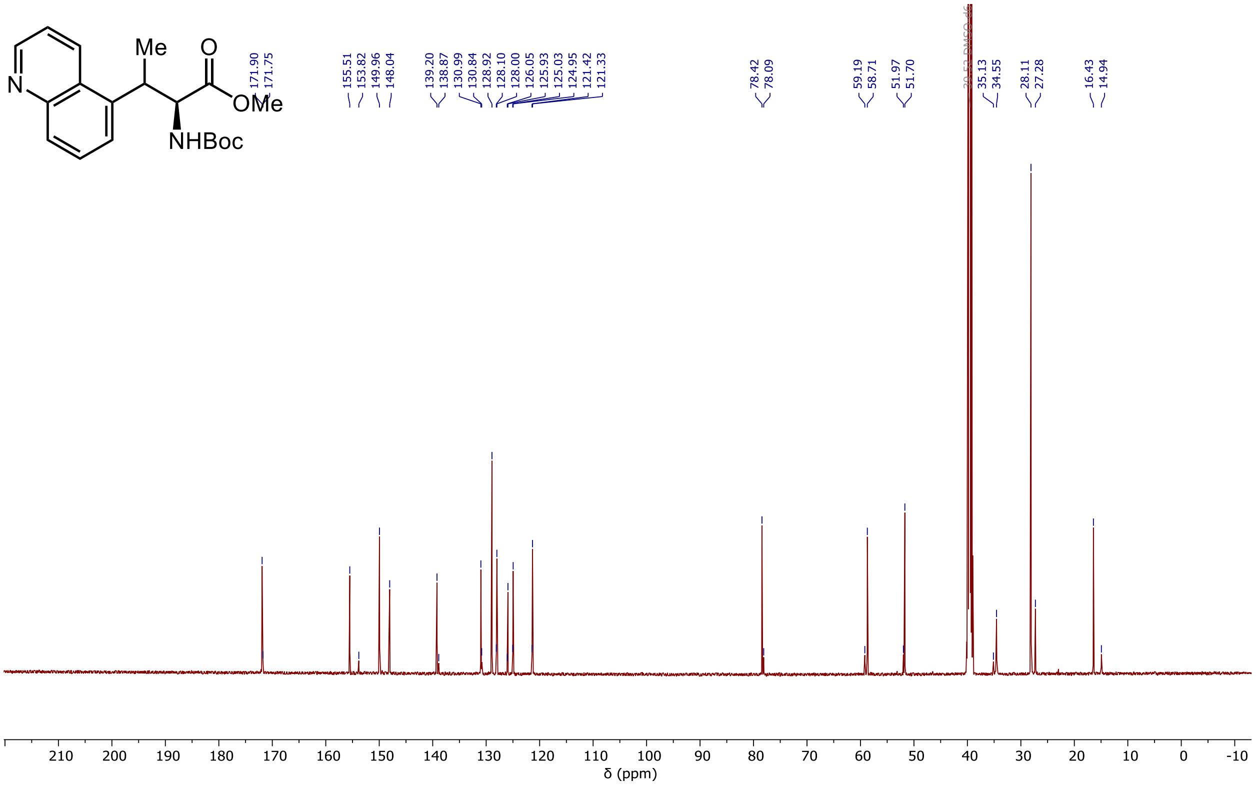

**Figure S136. $^{13}$C NMR spectra of (33a) (126 MHz, DMSO-$d_6$).**

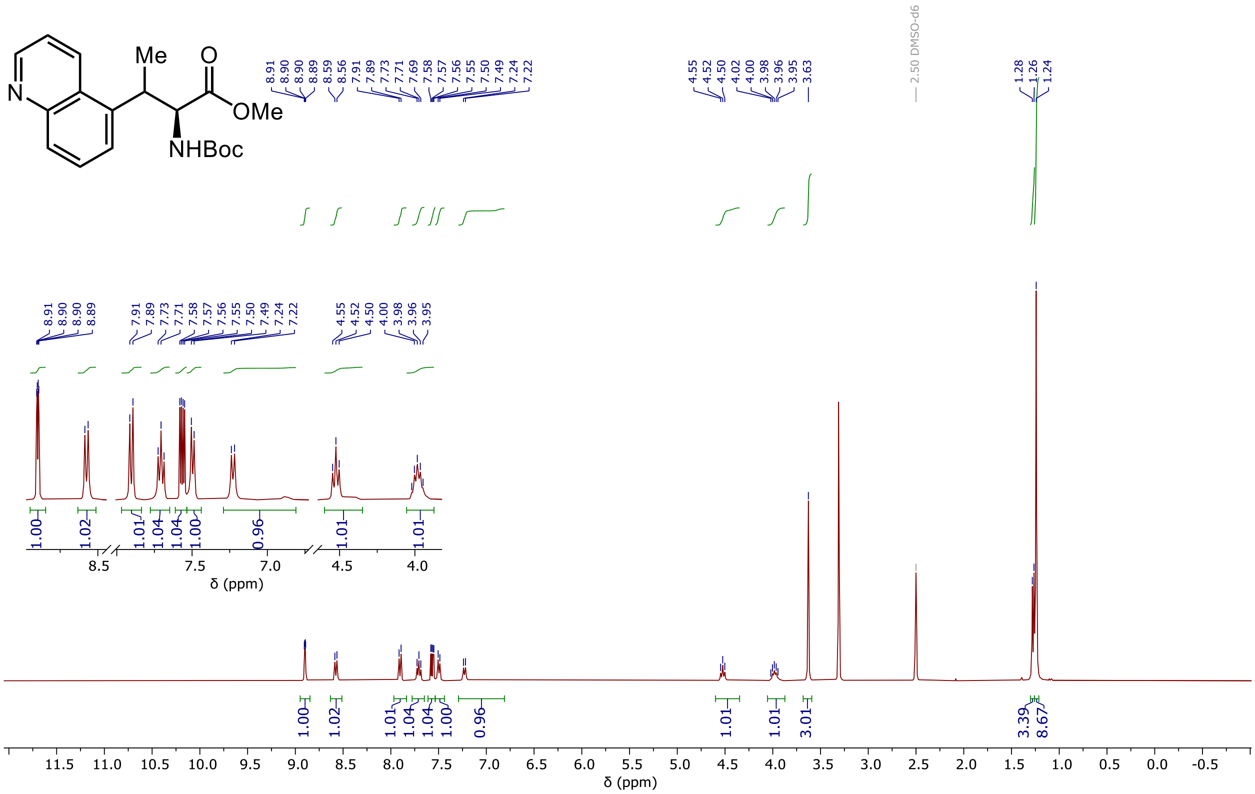

**Figure S137.** $^{1}$**H NMR spectra of (33b) (400 MHz, DMSO-d$_6$).**

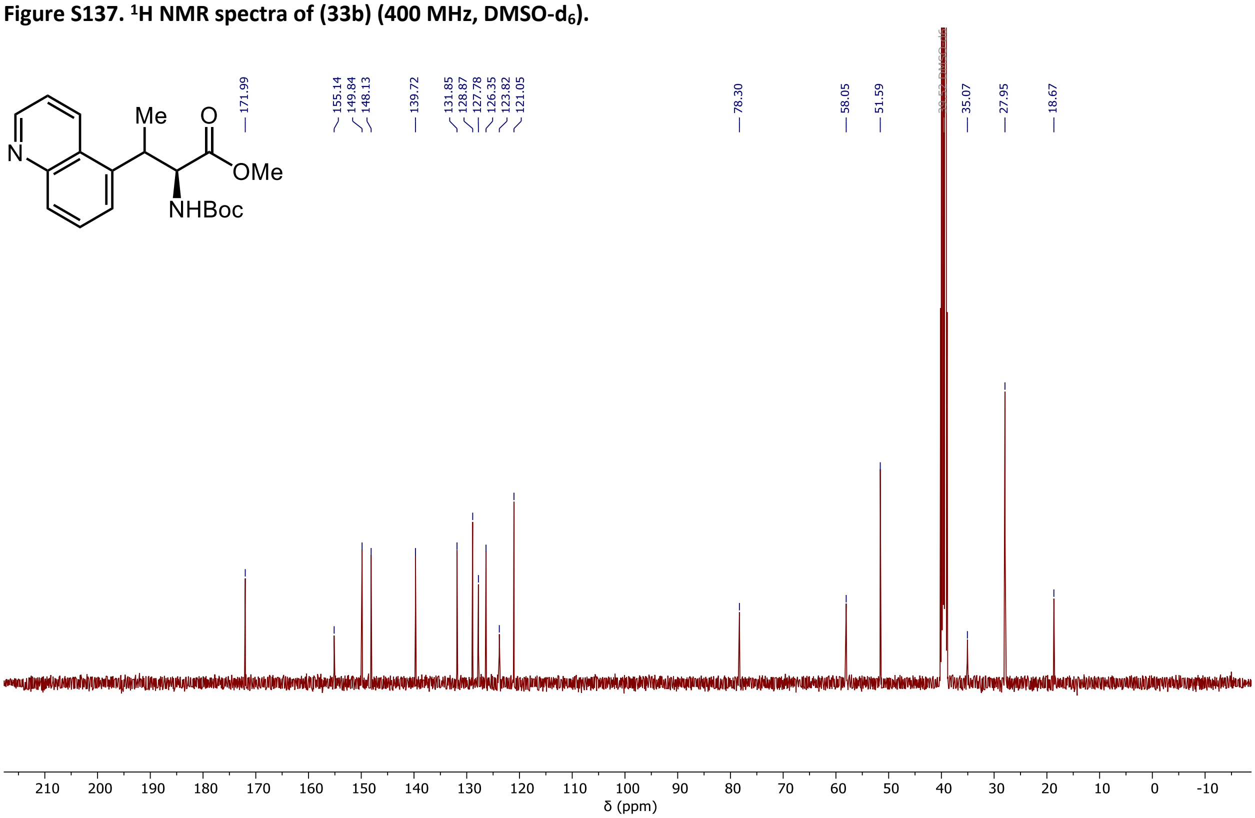

**Figure S138.** $^{13}$**C NMR spectra of (33b) (101 MHz, DMSO-d$_6$).**

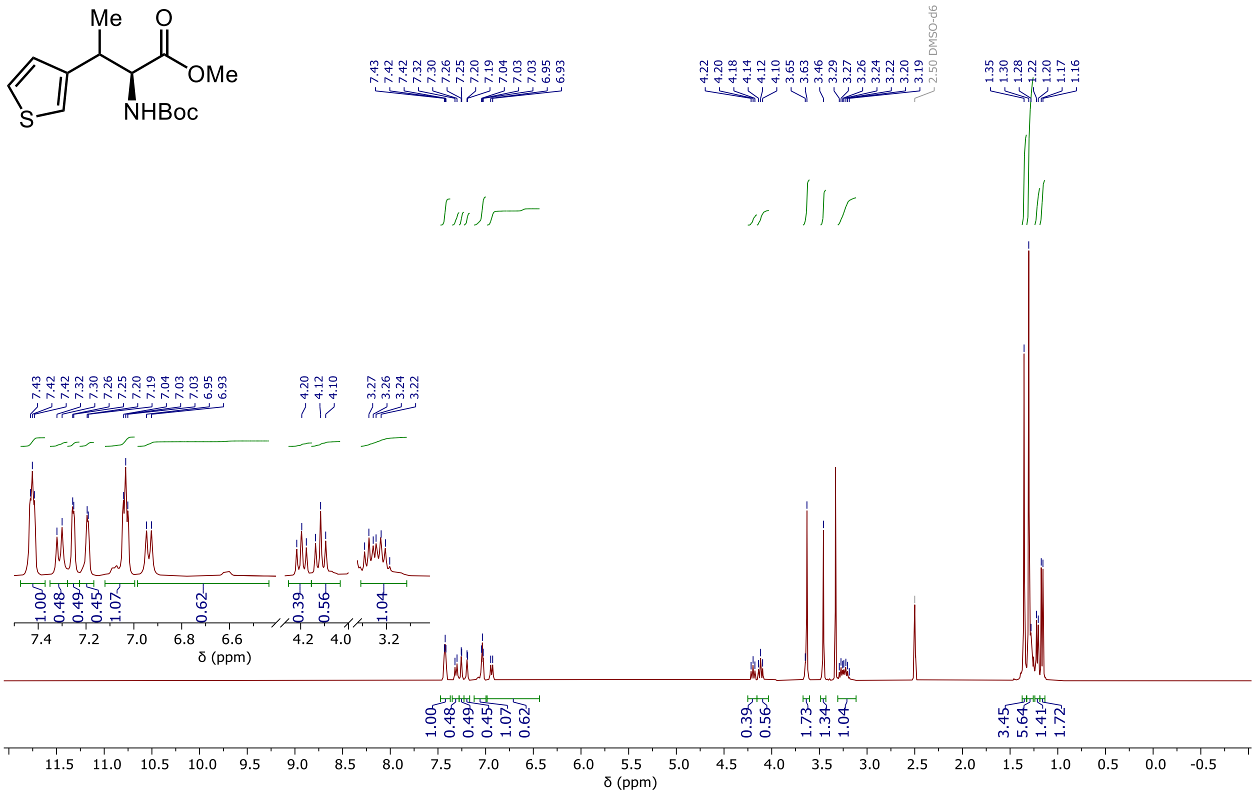

**Figure S139.** $^{1}H$ **NMR spectra of (34) (400 MHz, DMSO-d$_6$).**

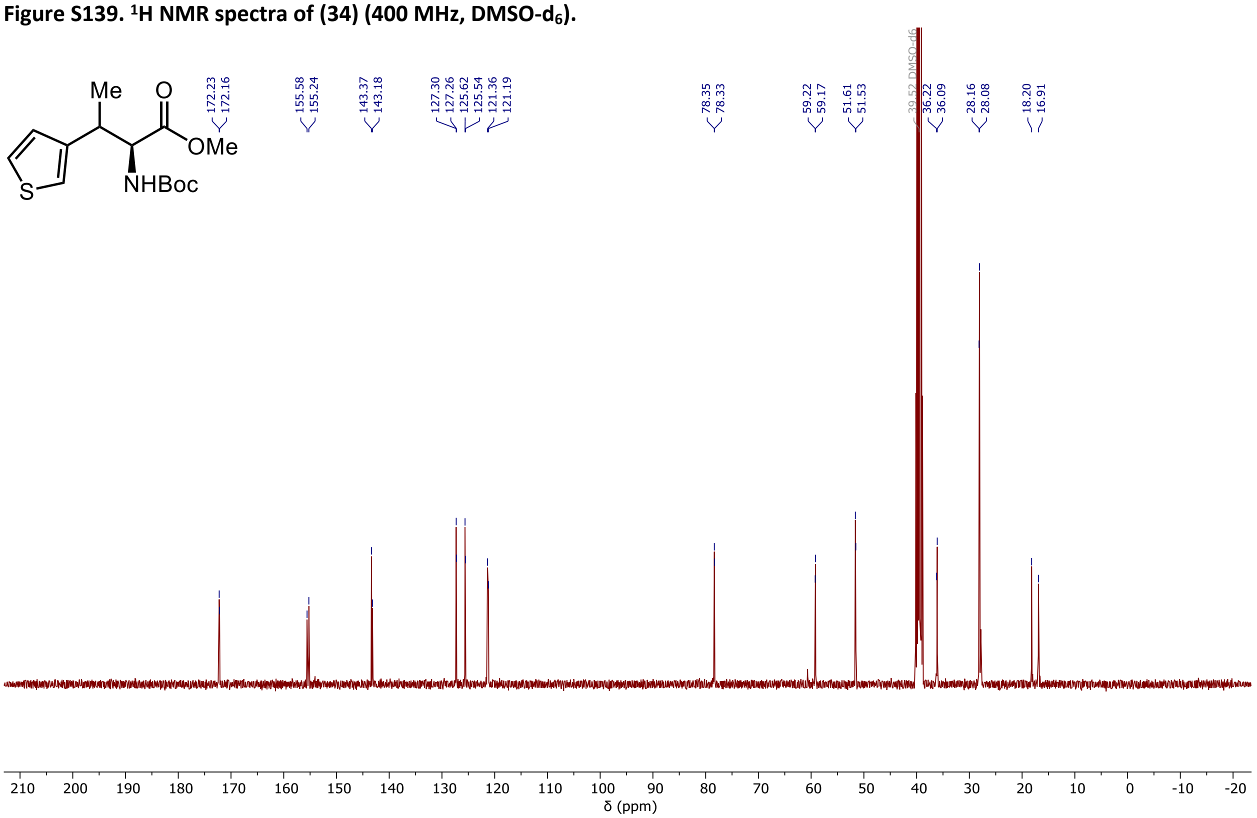

**Figure S140.** $^{13}C$ **NMR spectra of (34) (101 MHz, DMSO-d$_6$).**

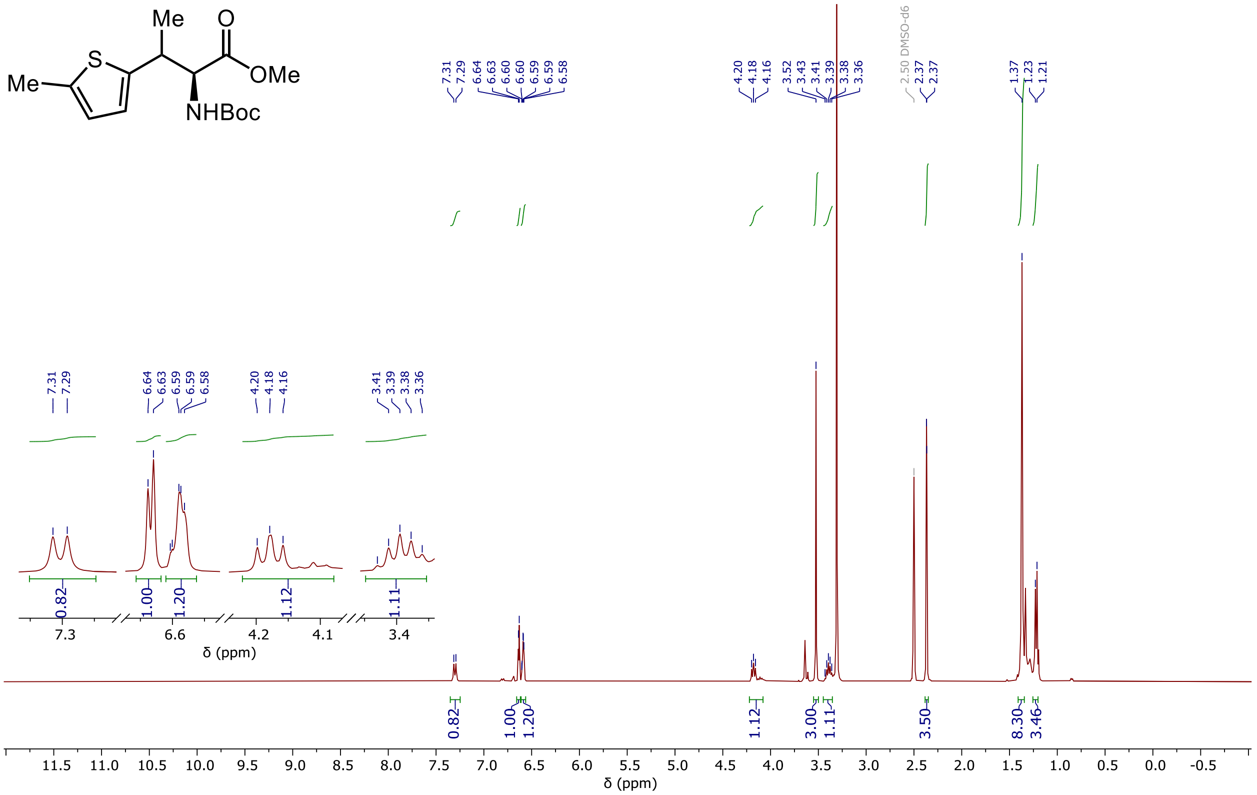

**Figure S141.** **$^1$H NMR spectra of (35a) (400 MHz, DMSO-$d_6$).**

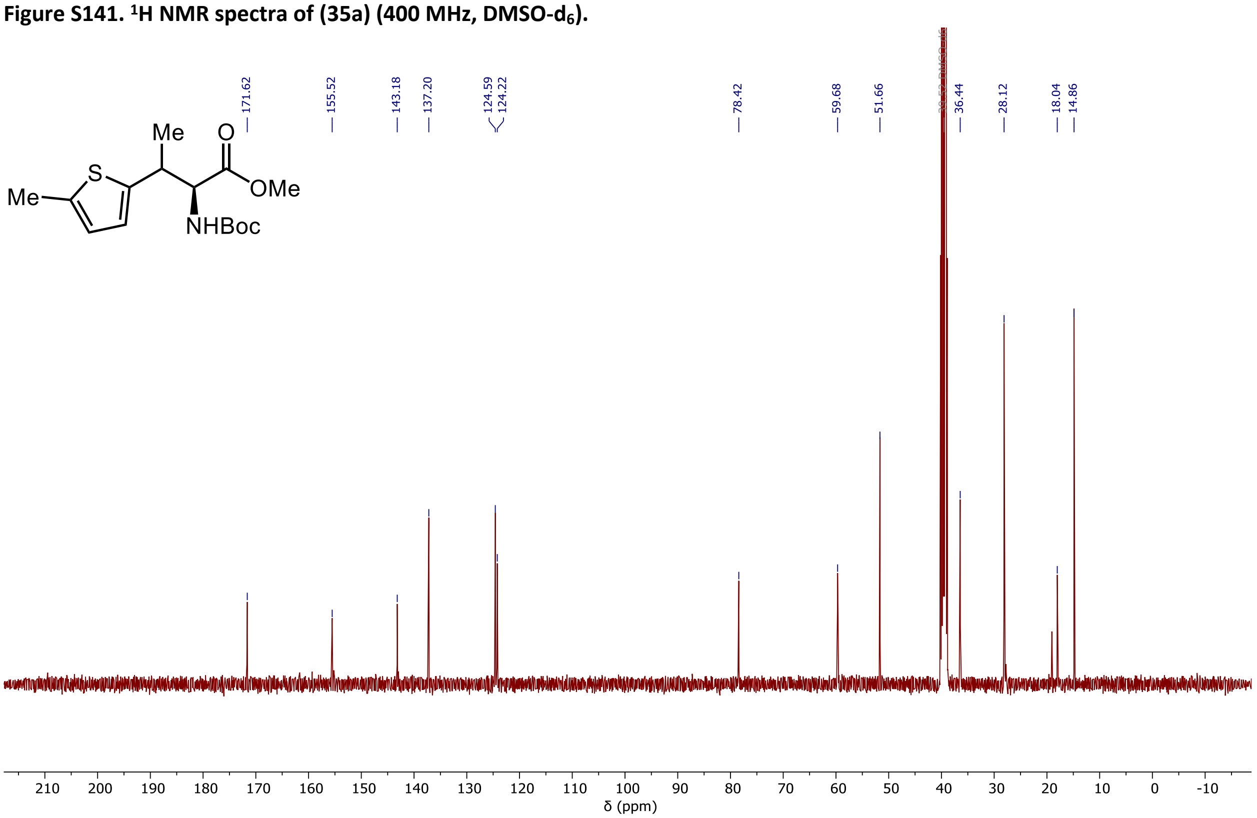

**Figure S142.** **$^{13}$C NMR spectra of (35a) (101 MHz, DMSO-$d_6$).**

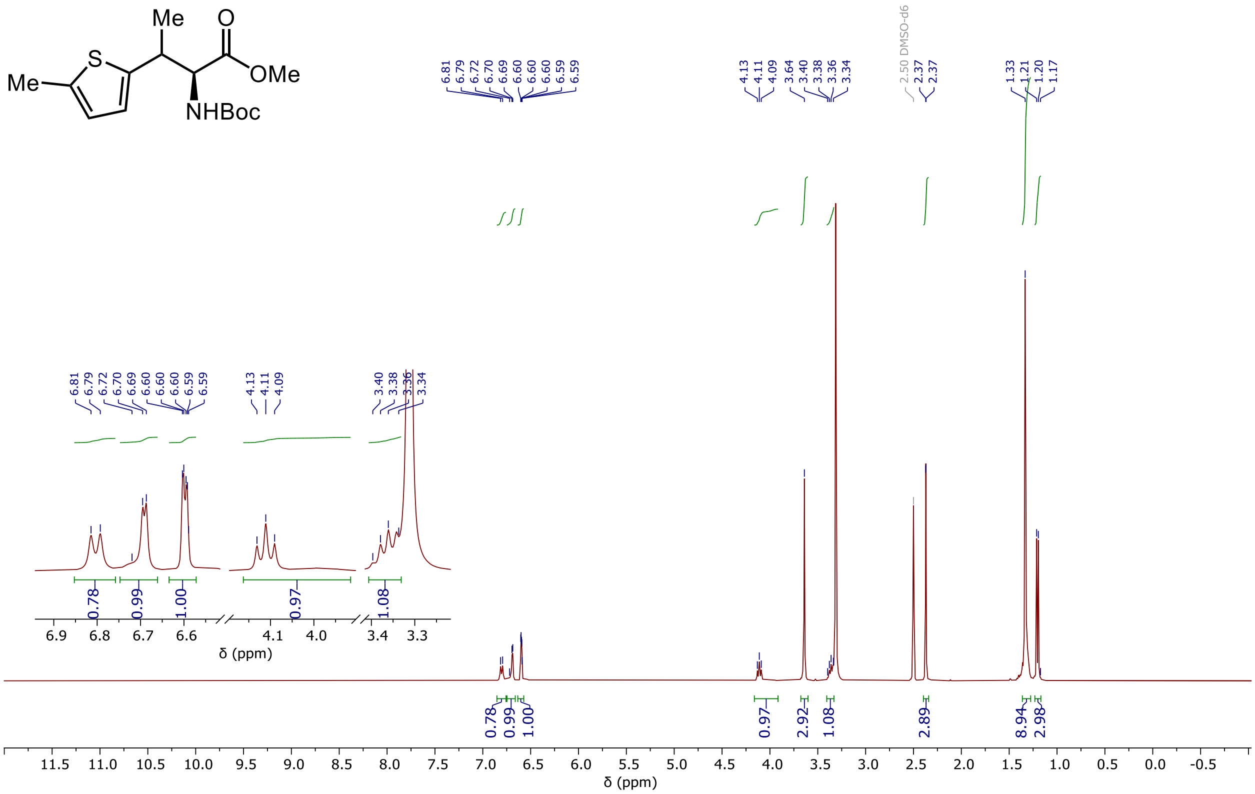

**Figure S143. $^{1}$H NMR spectra of (35b) (400 MHz, DMSO-$d_6$).**

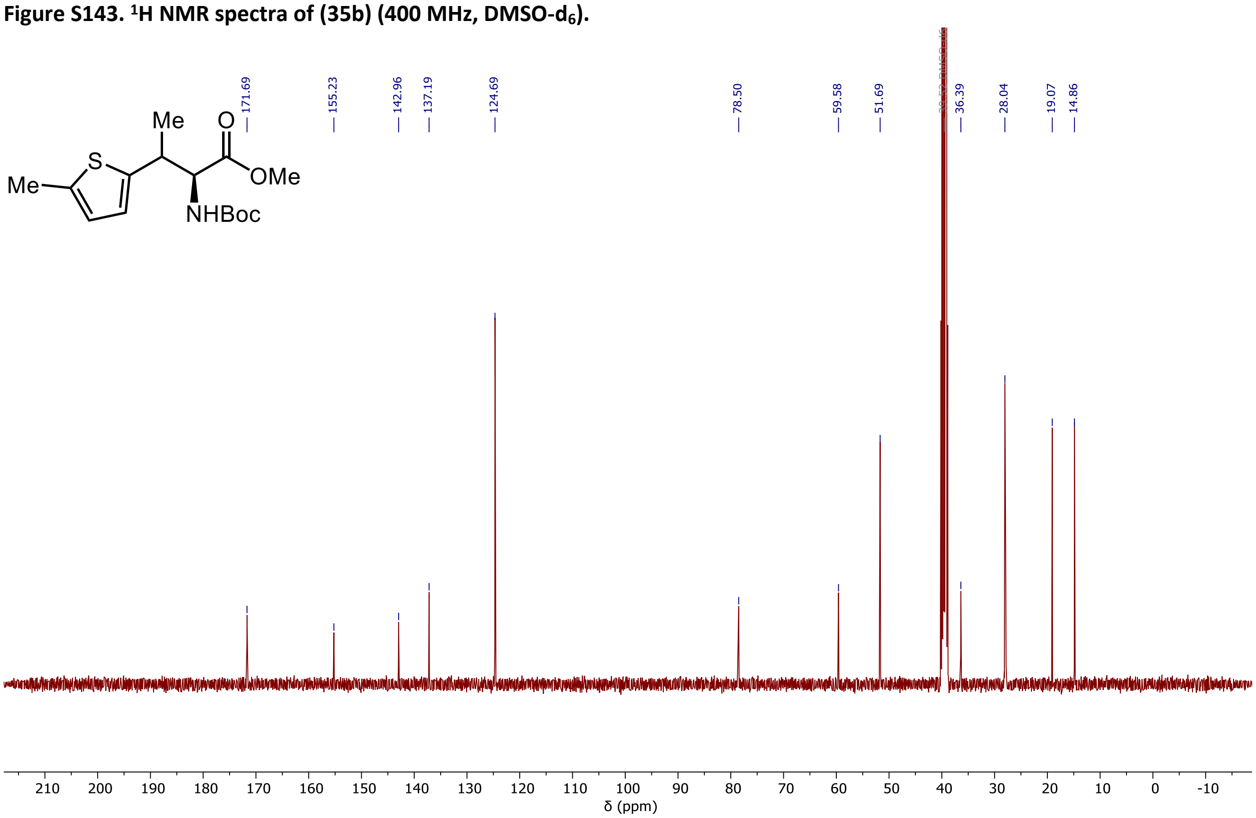

**Figure S144. $^{13}$C NMR spectra of (35b) (101 MHz, DMSO-$d_6$).**

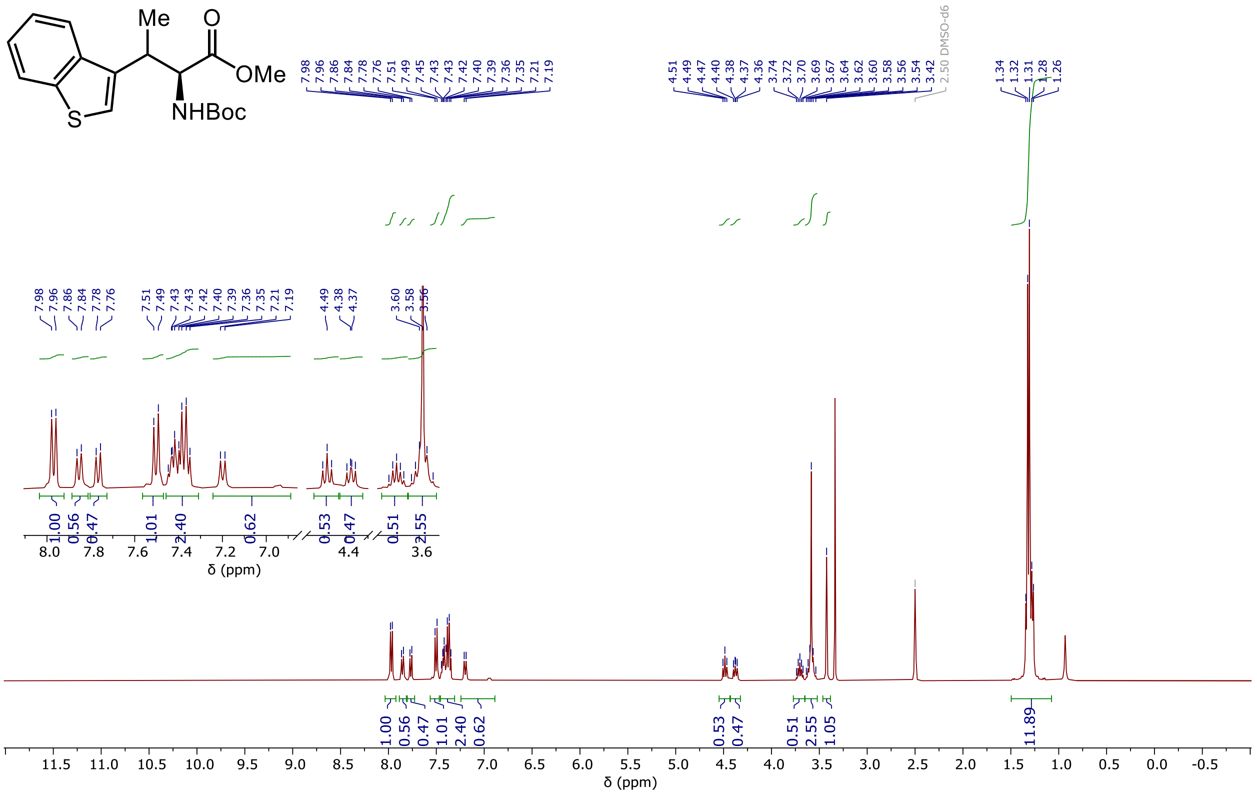

**Figure S145.** $^{1}$**H NMR spectra of (36) (400 MHz, DMSO-d$_6$).**

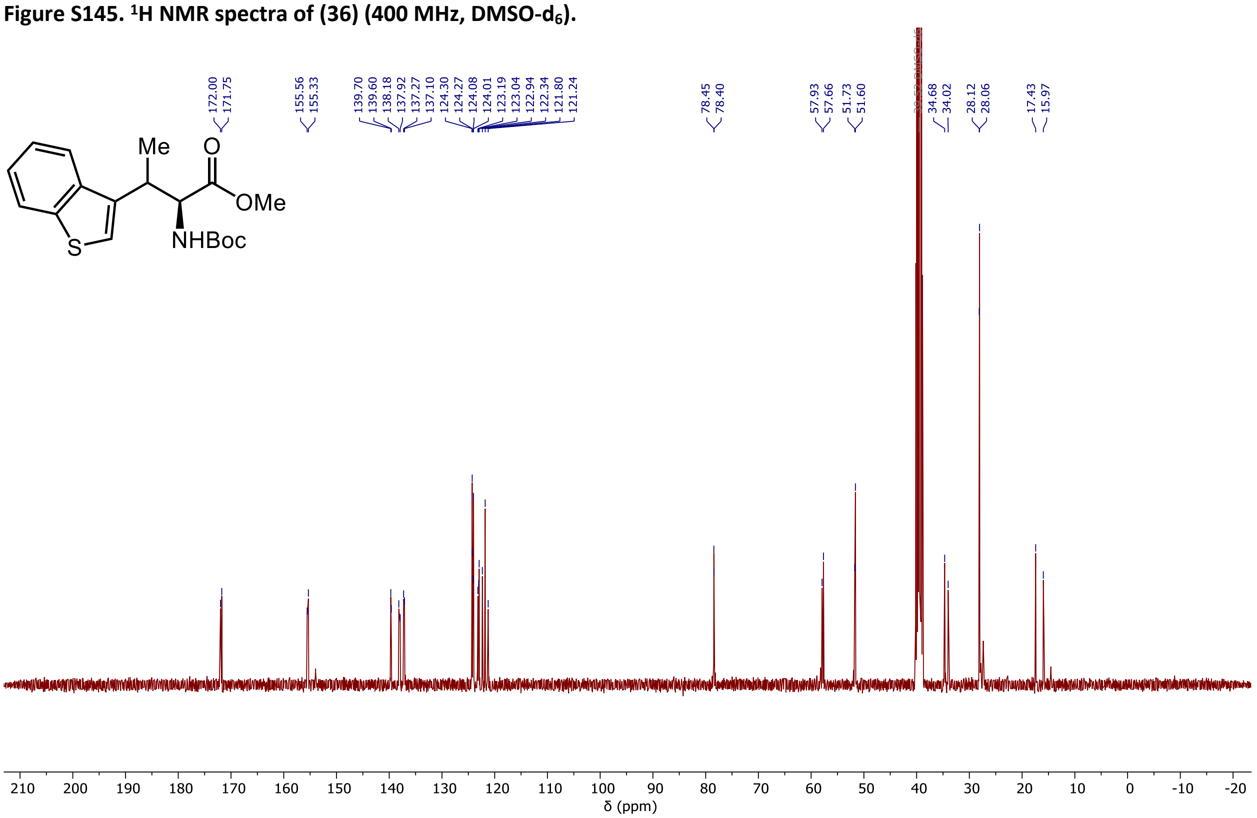

**Figure S146.** $^{13}$**C NMR spectra of (36) (101 MHz, DMSO-d$_6$).**

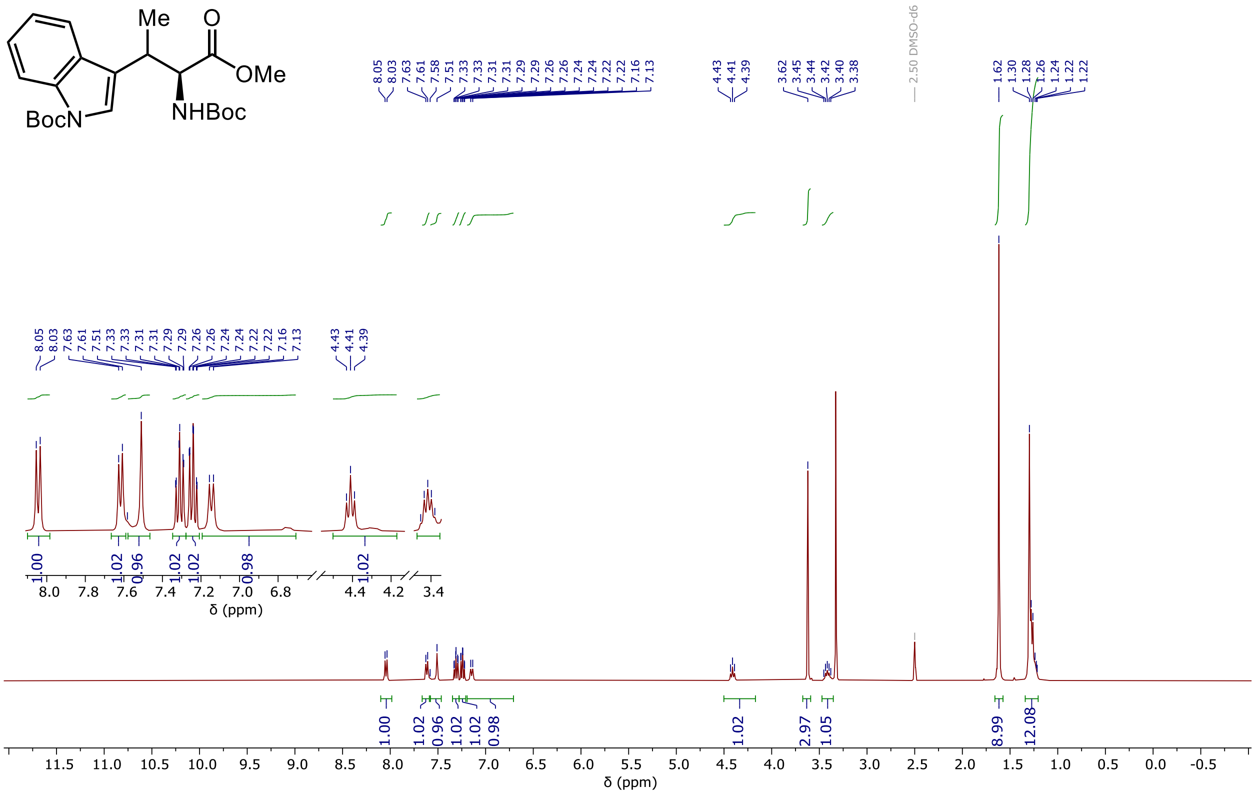


**Figure S147.** **$^{1}$H NMR spectra of (37a) (400 MHz, DMSO-d$_6$).**

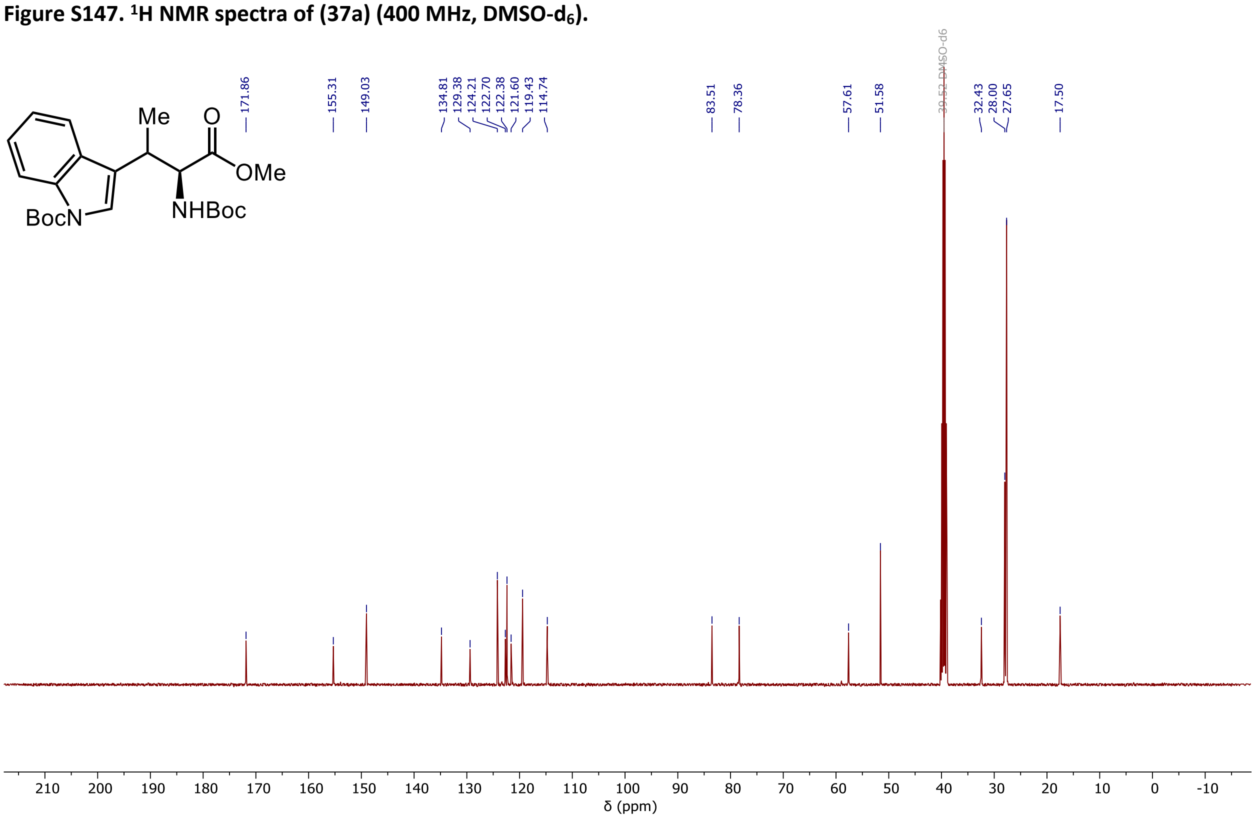


**Figure S148.** **$^{13}$C NMR spectra of (37a) (101 MHz, DMSO-d$_6$).**

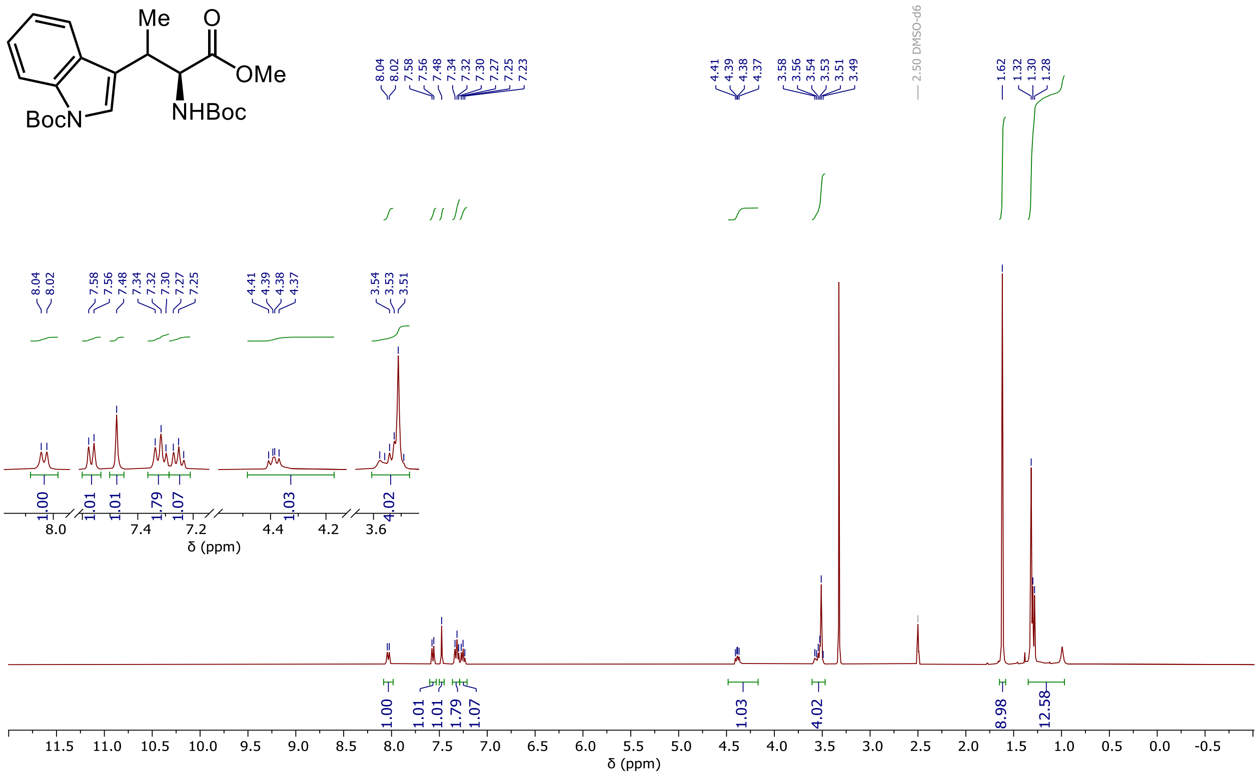

**Figure S149.** $^{1}$**H NMR spectra of (37b) (400 MHz, DMSO-d$_6$).**

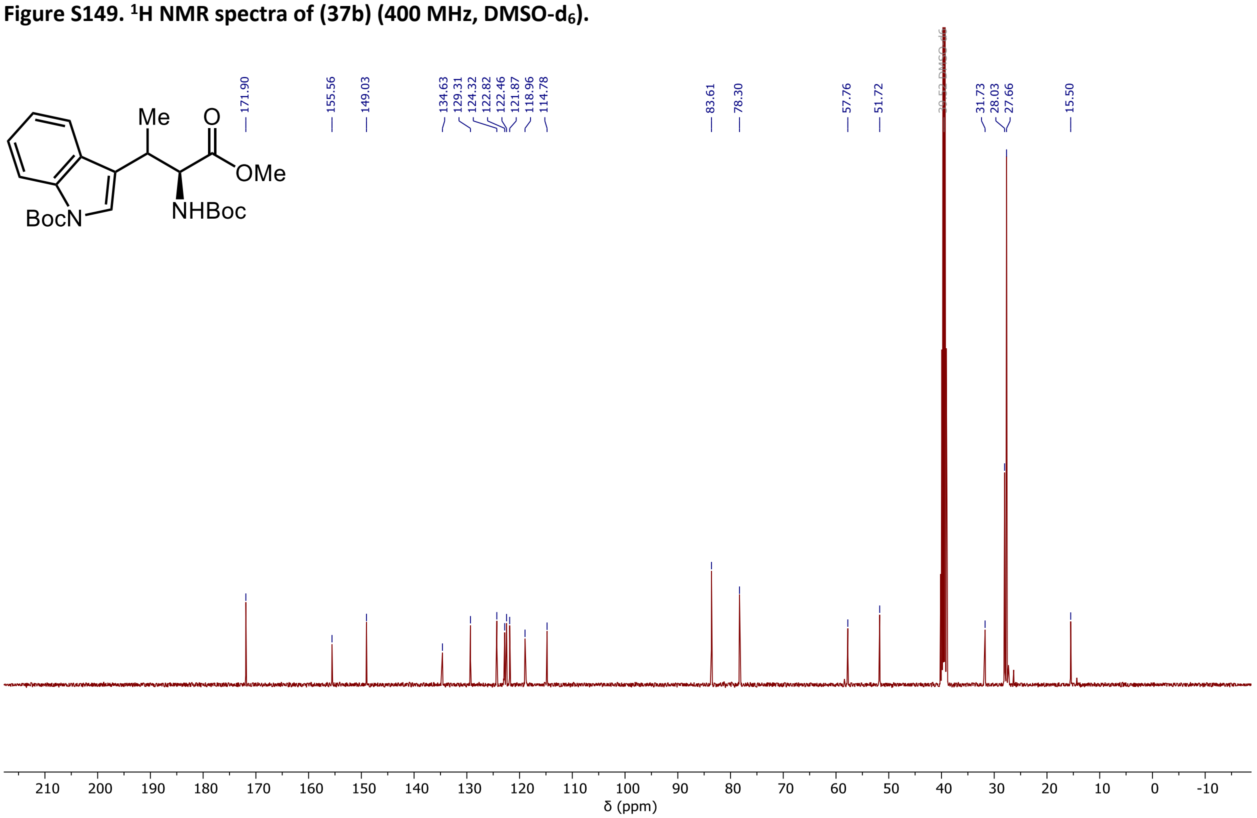

**Figure S150.** $^{13}$**C NMR spectra of (37b) (101 MHz, DMSO-d$_6$).**

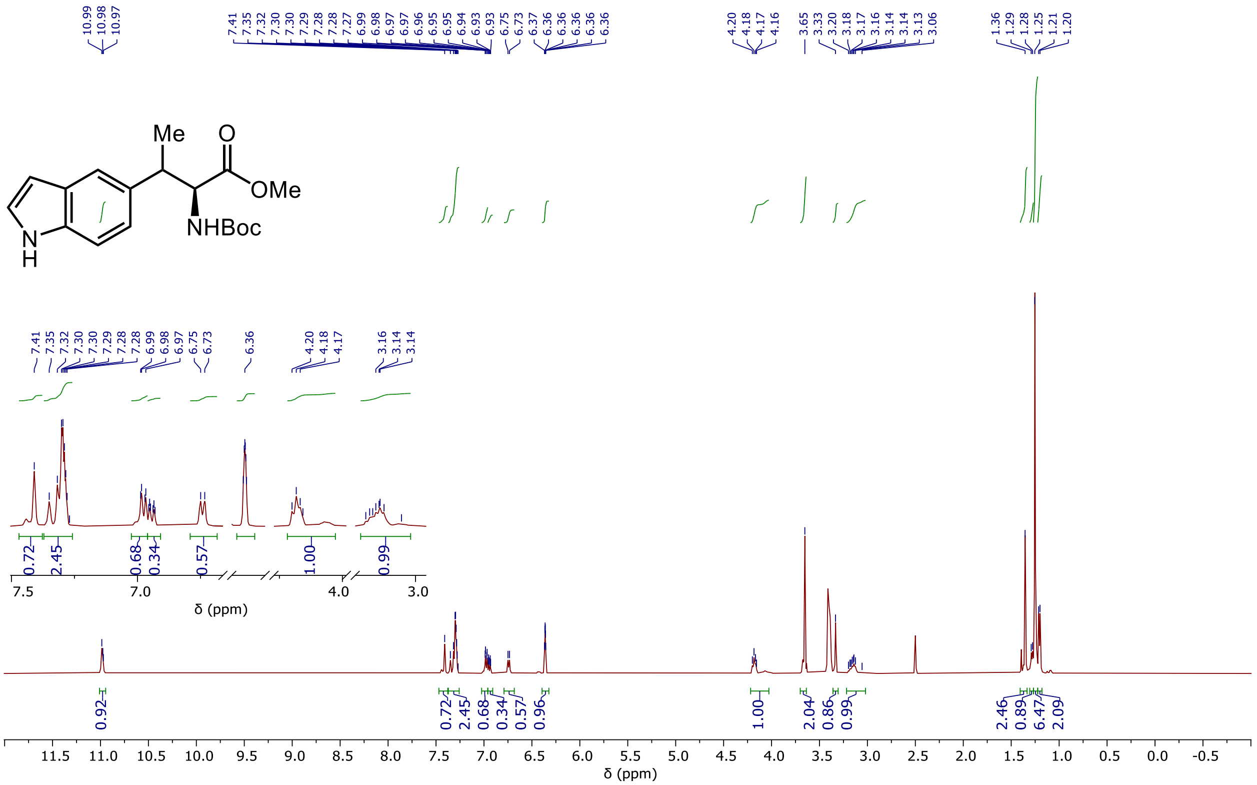

**Figure S151.** **$^{1}$H NMR spectra of (38) (500 MHz, DMSO-$d_6$).**

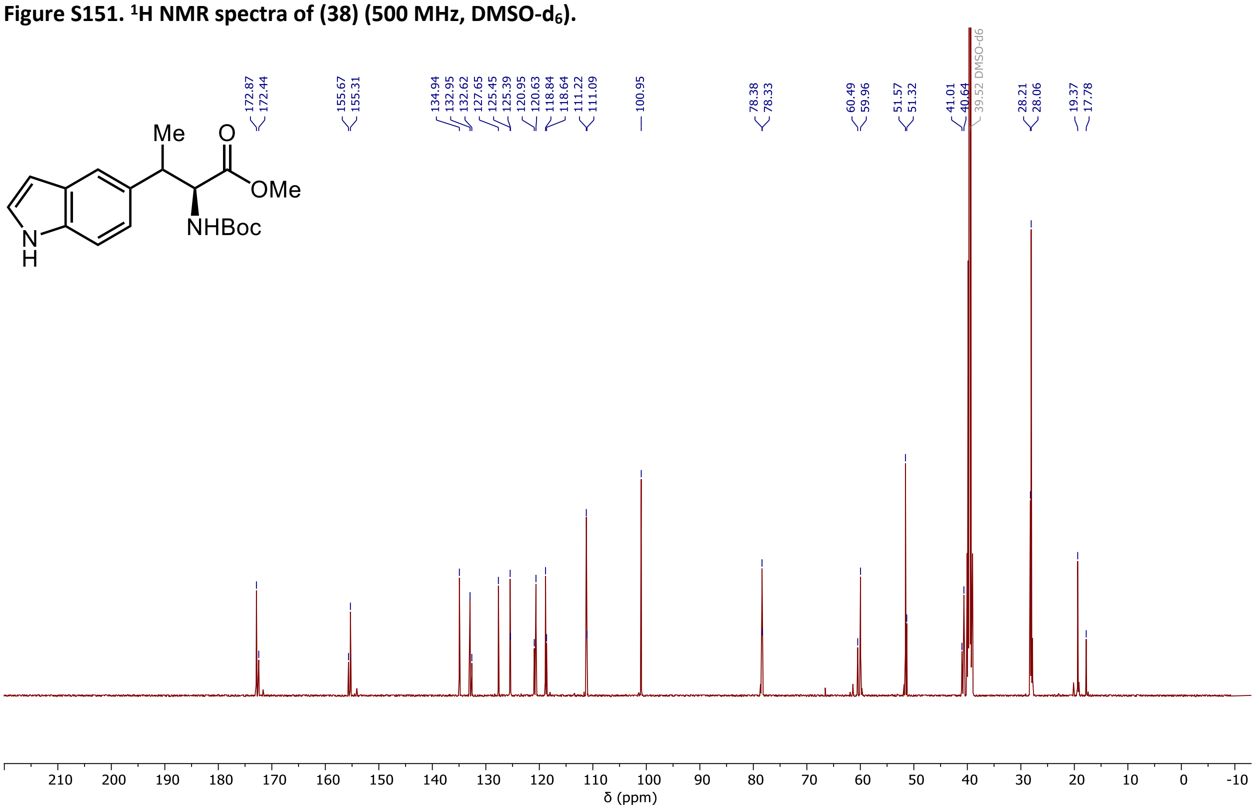

**Figure S152.** **$^{13}$C NMR spectra of (38) (126 MHz, DMSO-$d_6$).**

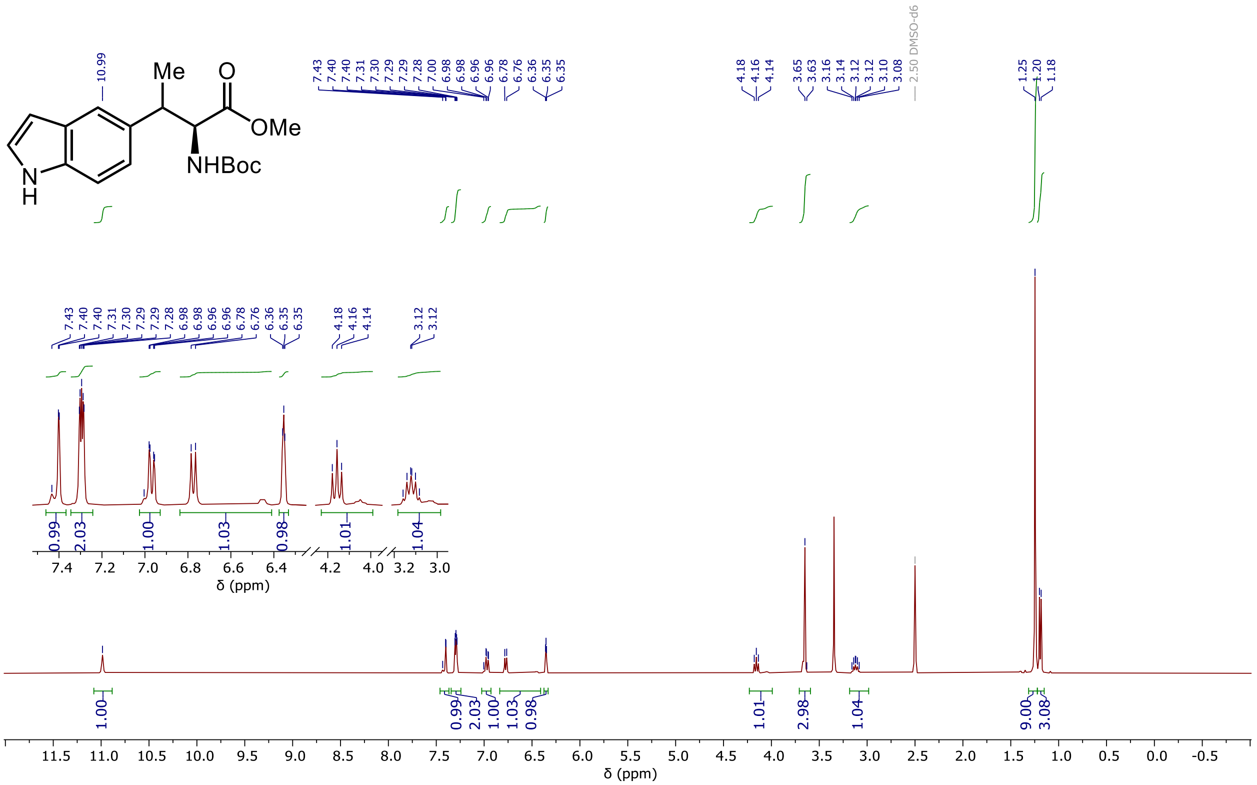


**Figure S153.** $^{1}$**H NMR spectra of (38’) (400 MHz, DMSO-d**$_{6}$**).**

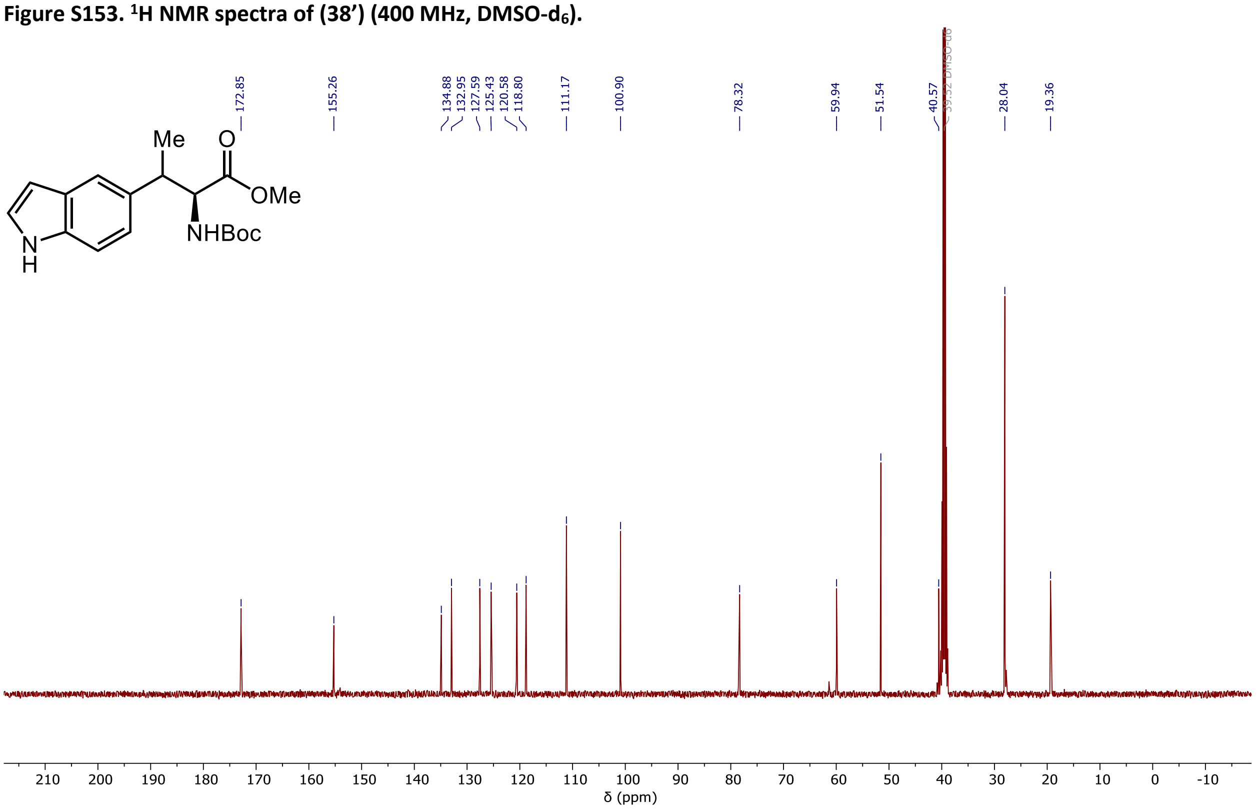


**Figure S154.** $^{13}$**C NMR spectra of (38’) (101 MHz, DMSO-d**$_{6}$**).**

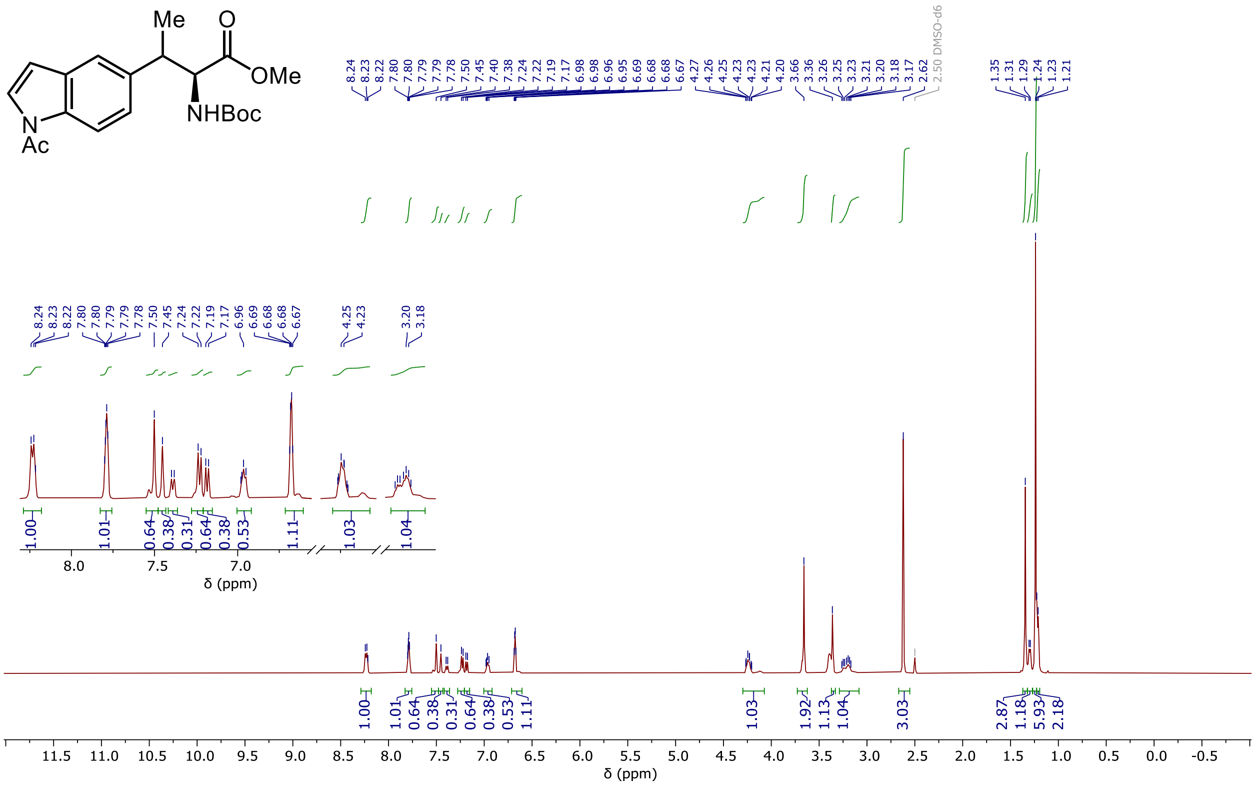

**Figure S155. $^{1}H$ NMR spectra of (39) (500 MHz, DMSO-$d_6$).**

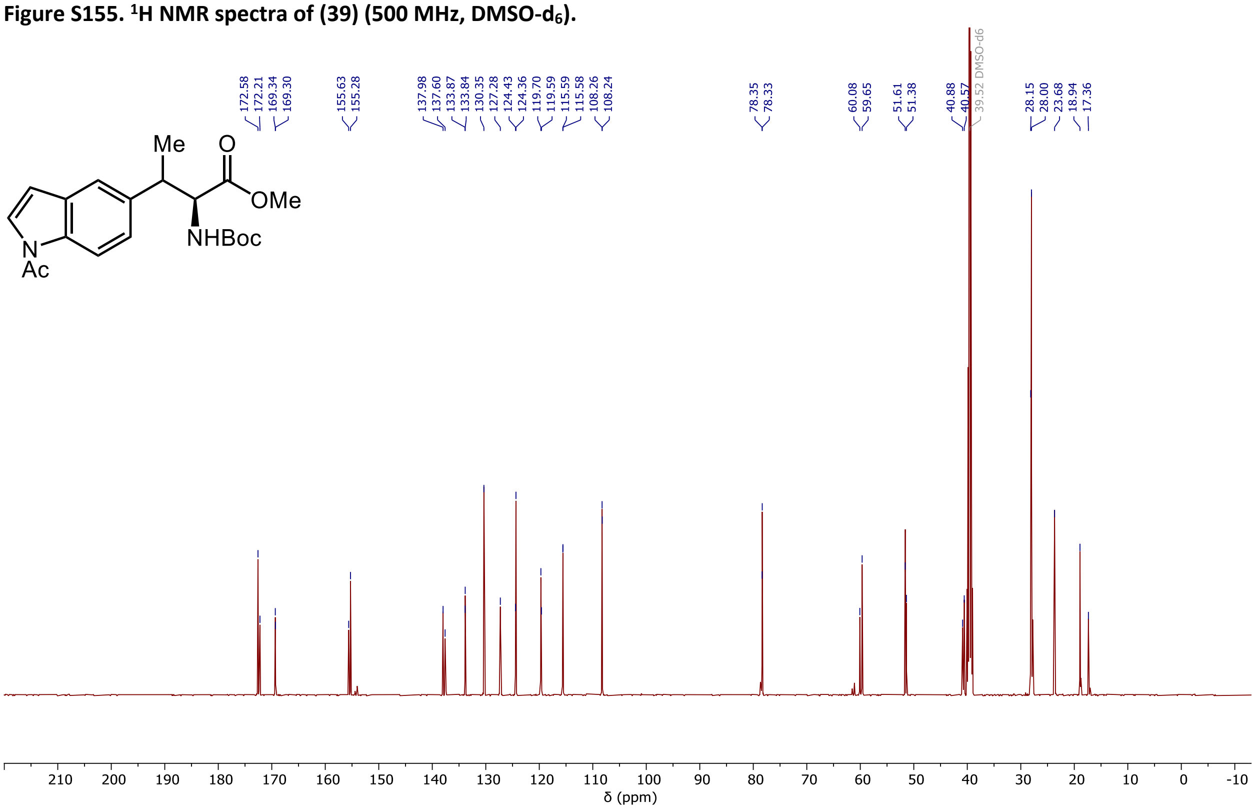

**Figure S156. $^{13}C$ NMR spectra of (39) (126 MHz, DMSO-$d_6$).**

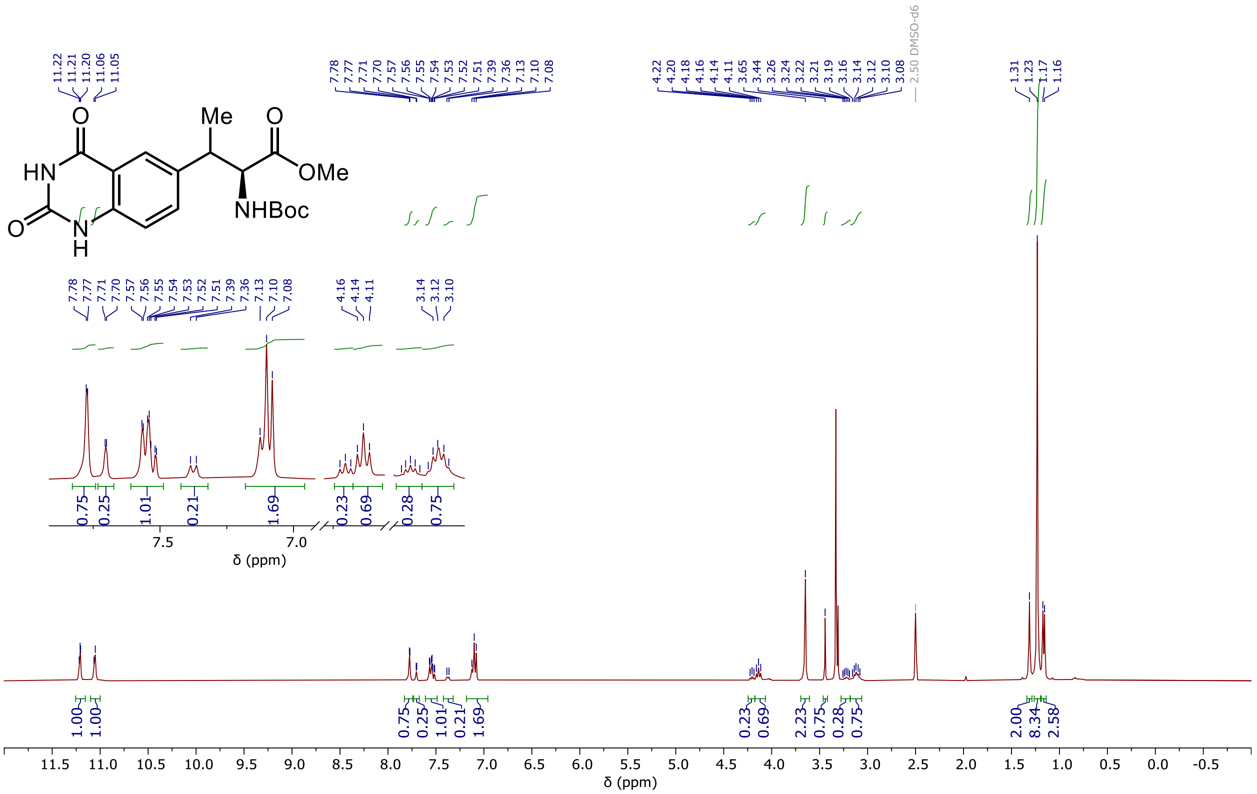

**Figure S157.** $^{1}$**H NMR spectra of (40) (400 MHz, DMSO-d$_6$).**

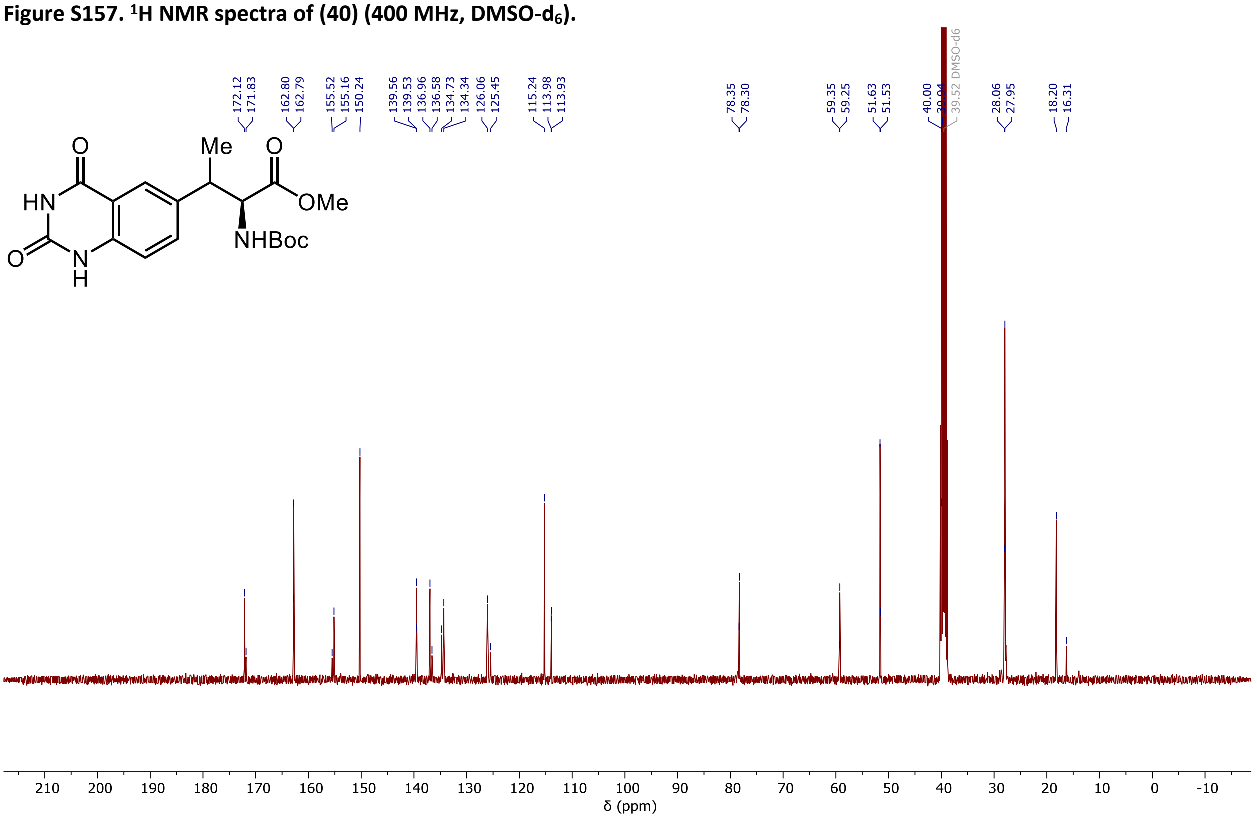

**Figure S158.** $^{13}$**C NMR spectra of (40) (101 MHz, DMSO-d$_6$).**

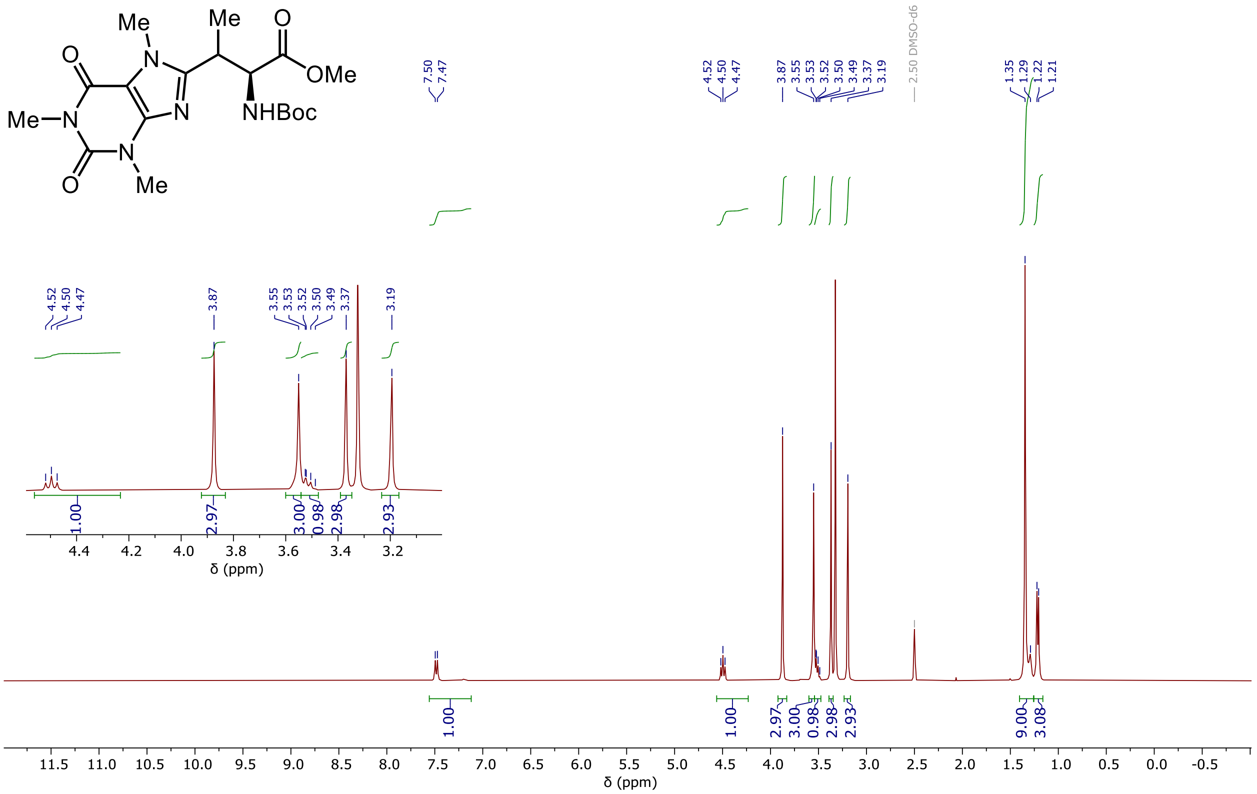

**Figure S159.** $^{1}$**H NMR spectra of (41) (400 MHz, DMSO-d$_6$).**

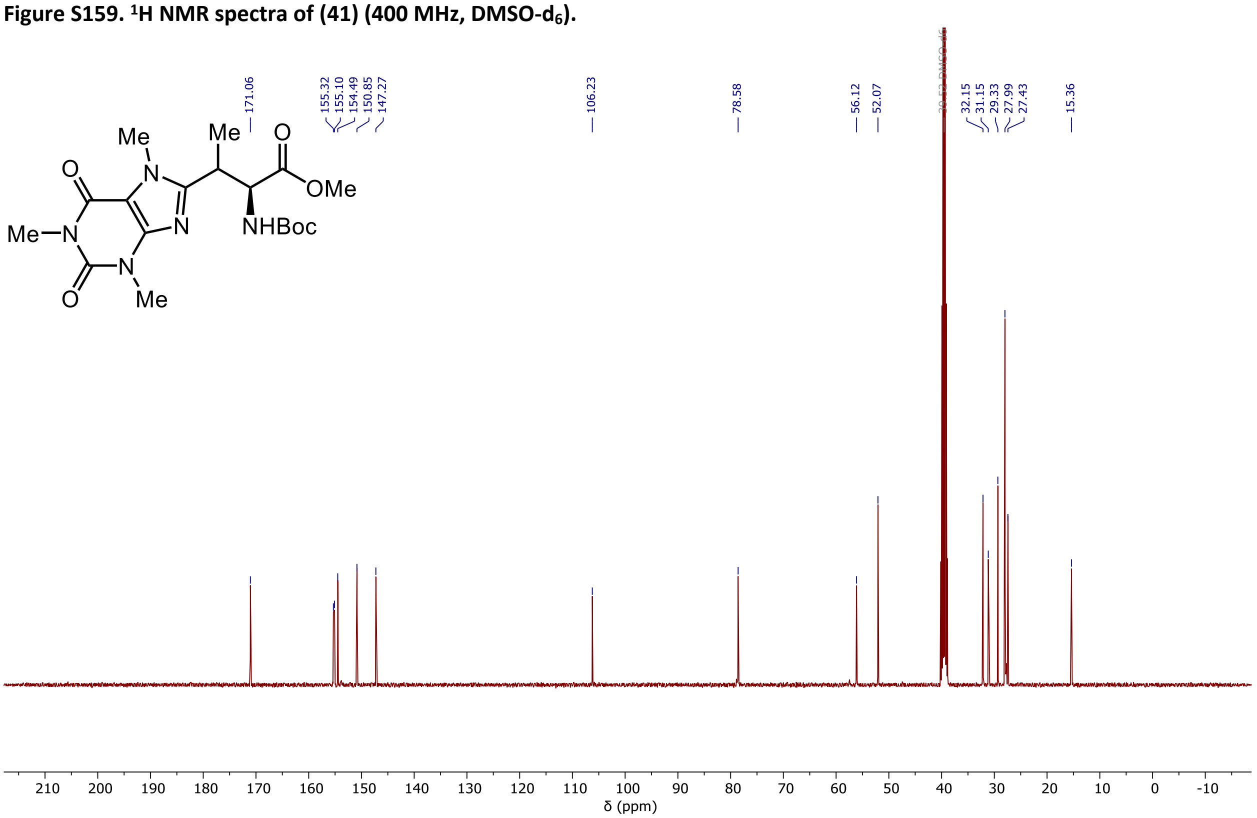

**Figure S160.** $^{13}$**C NMR spectra of (41) (101 MHz, DMSO-d$_6$).**

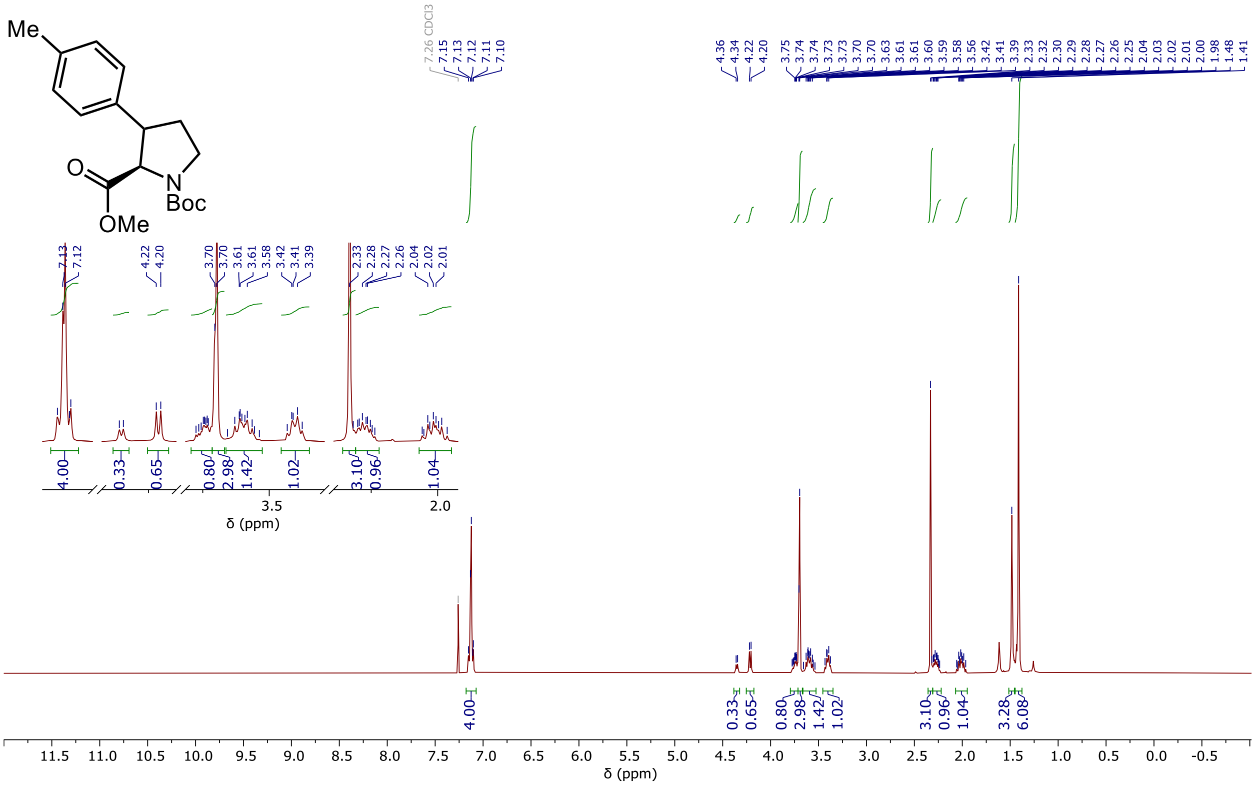


**Figure S161. $^{1}$H NMR spectra of (42) (400 MHz, $CDCl_3$).**

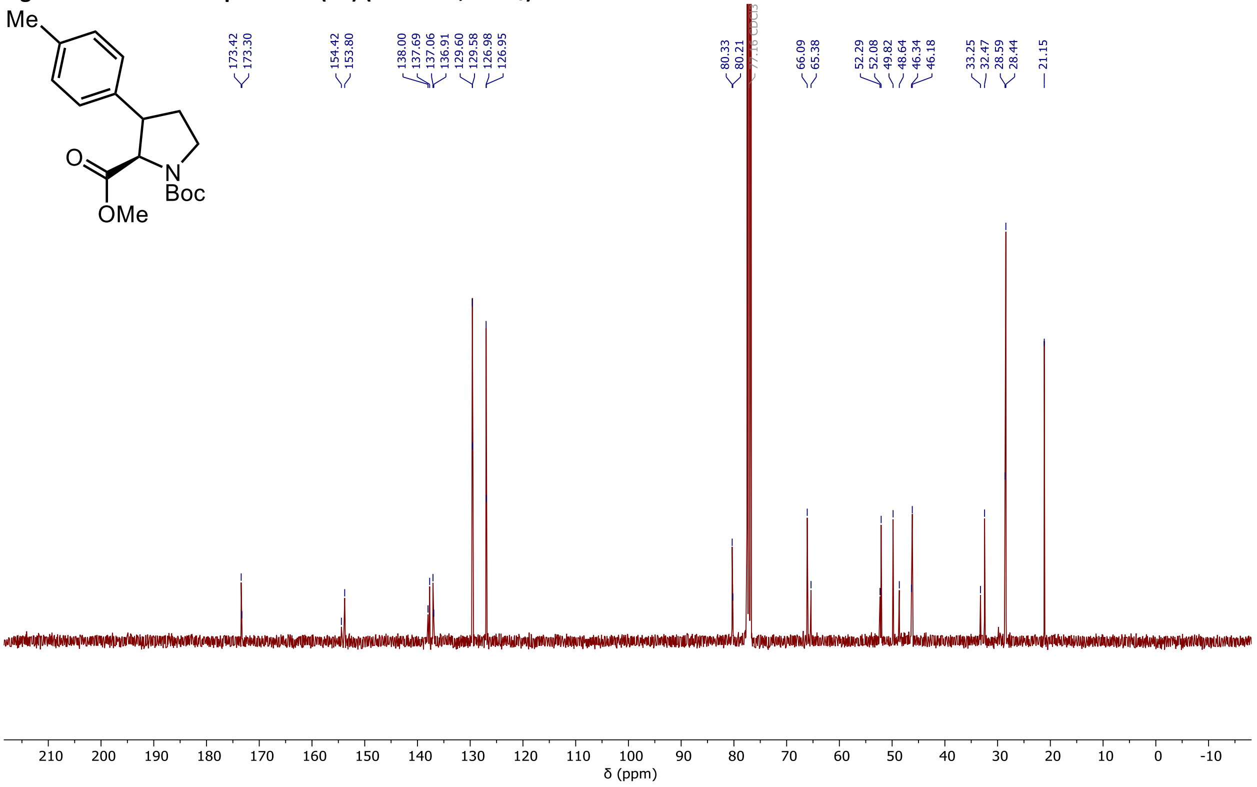


**Figure S162. $^{13}$C NMR spectra of (42) (101 MHz, $CDCl_3$).**

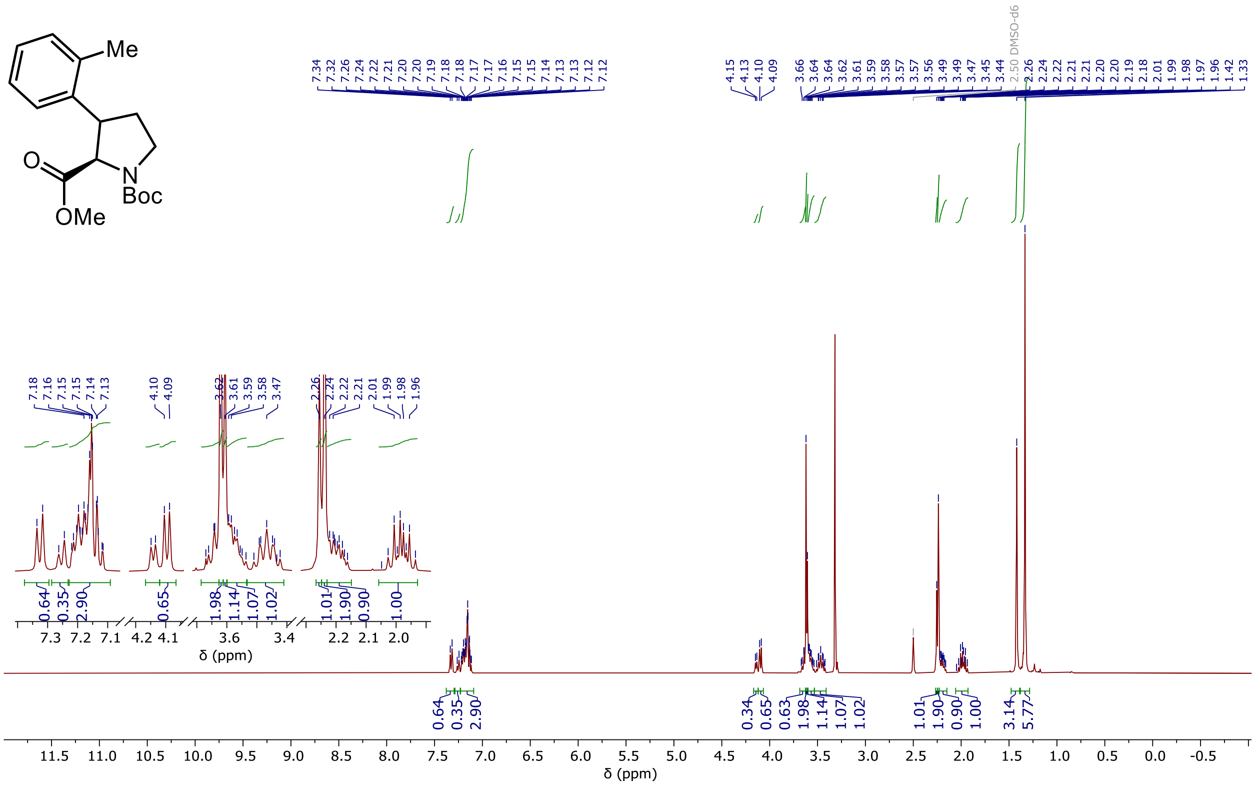


**Figure S163. $^{1}$H NMR spectra of (43) (400 MHz, $CDCl_3$).**

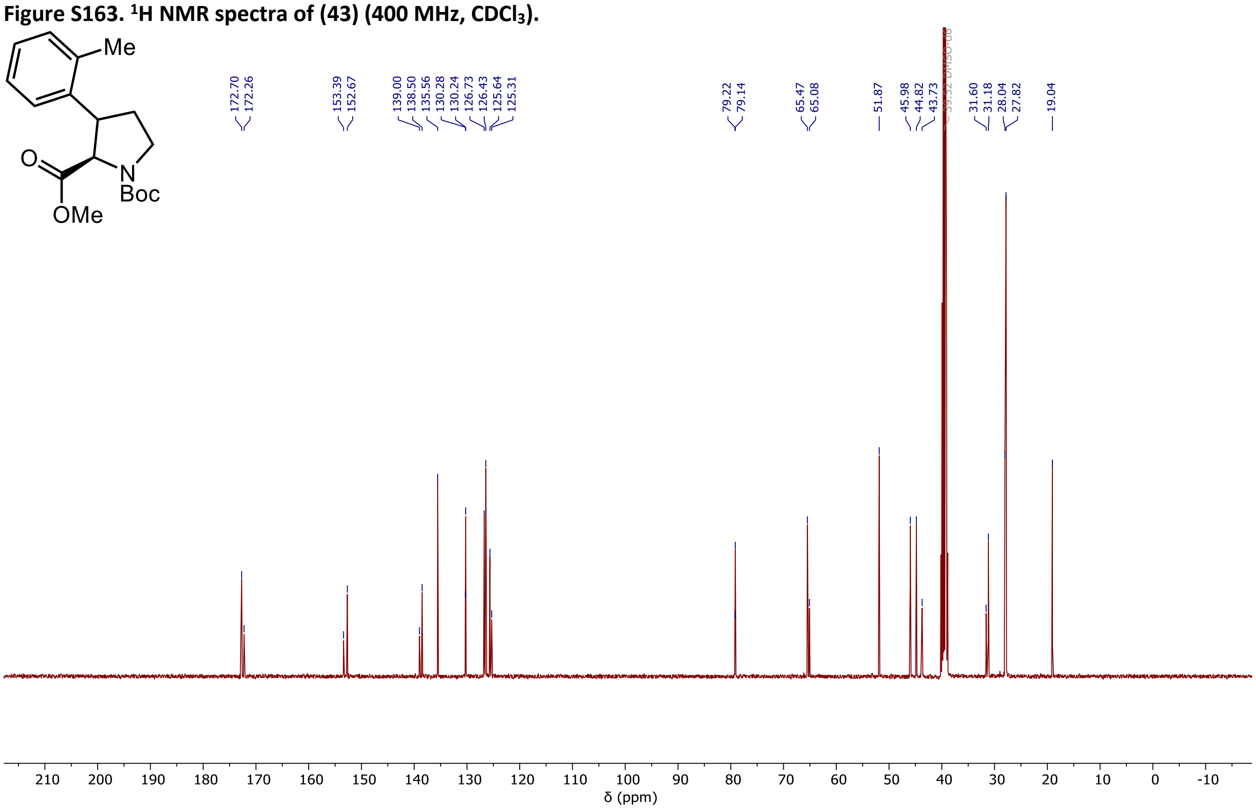


**Figure S164. $^{13}$C NMR spectra of (43) (101 MHz, $CDCl_3$).**

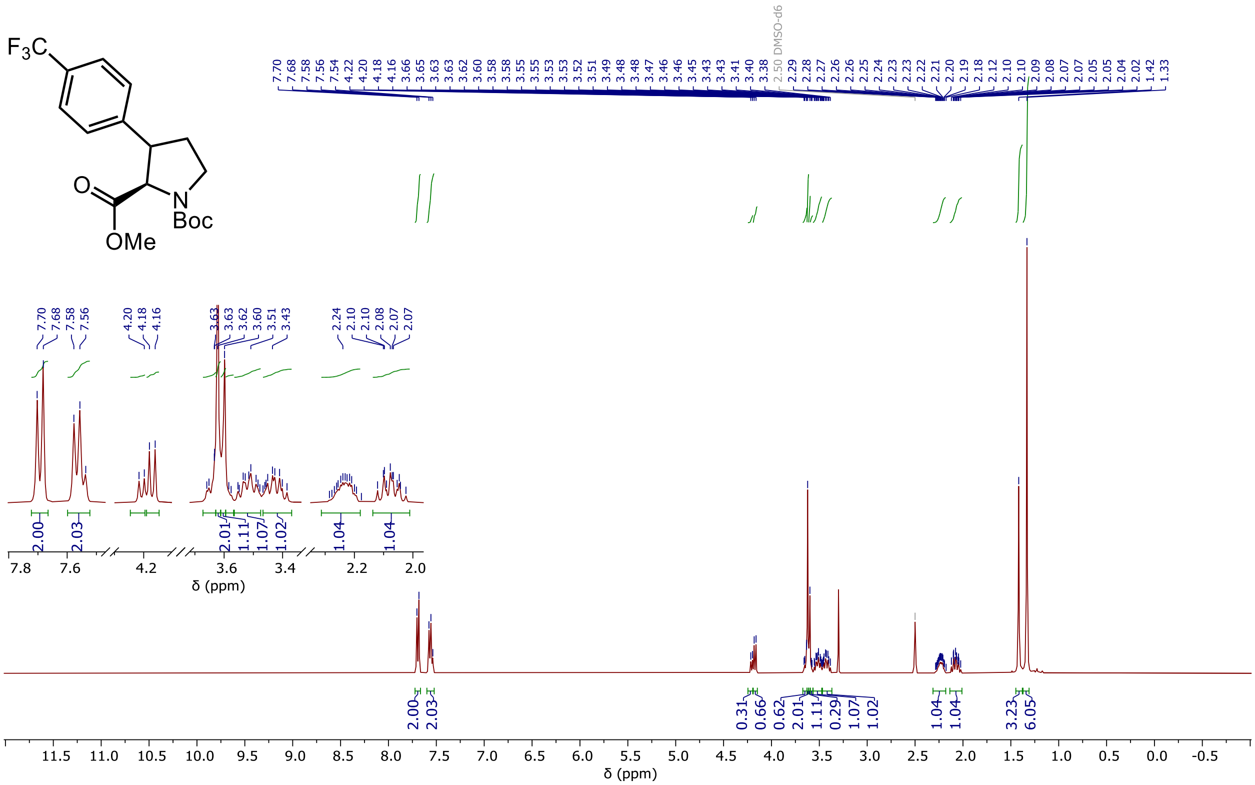


**Figure S165. $^{1}$H NMR spectra of (44) (400 MHz, DMSO-d$_6$).**

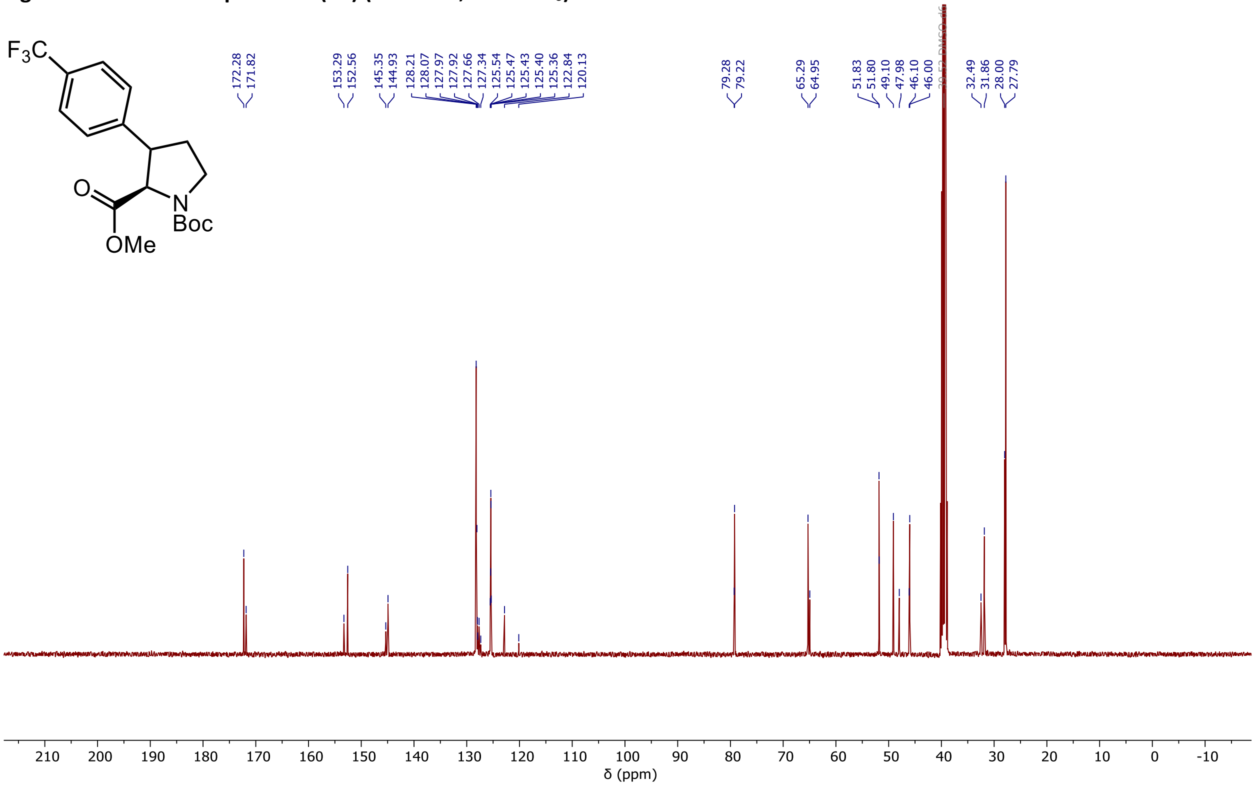


**Figure S166. $^{13}$C NMR spectra of (44) (101 MHz, DMSO-d$_6$).**

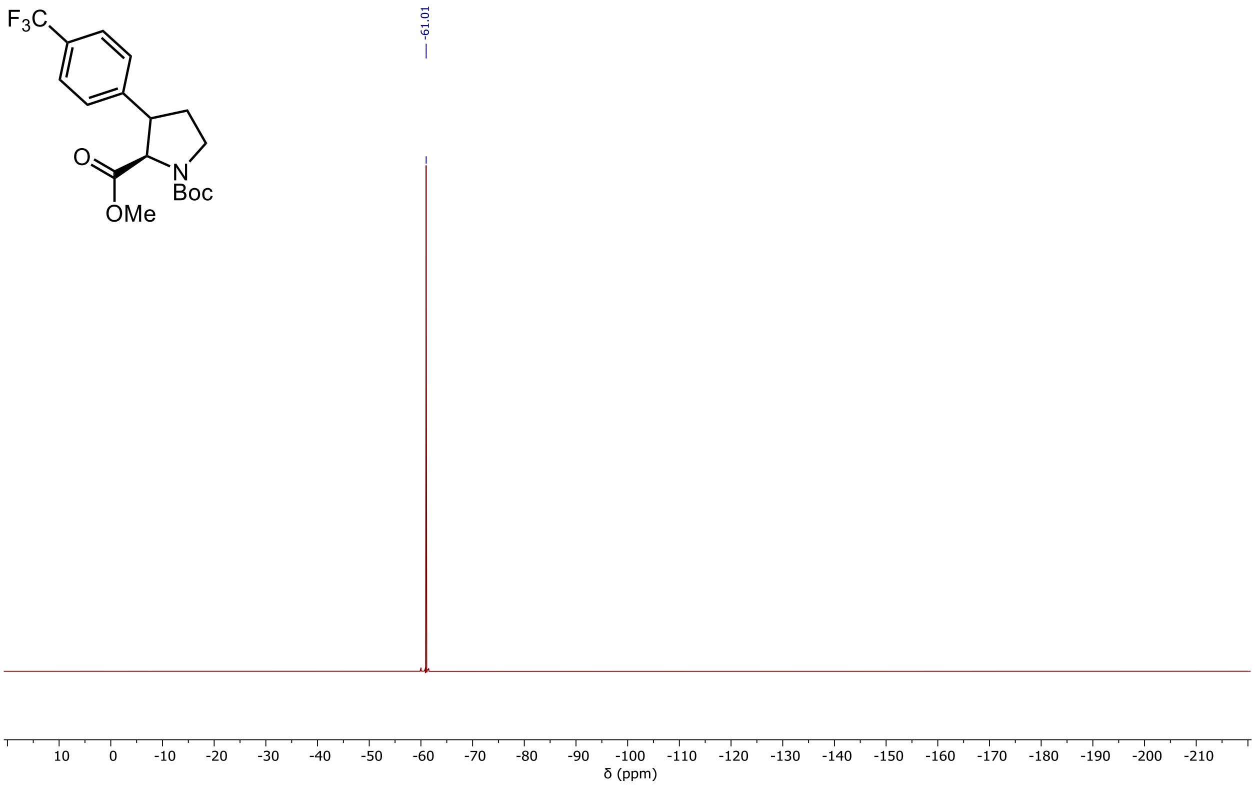


**Figure S167.** $^{19}$**F NMR spectra of (44) (376 MHz, DMSO-d$_6$).**

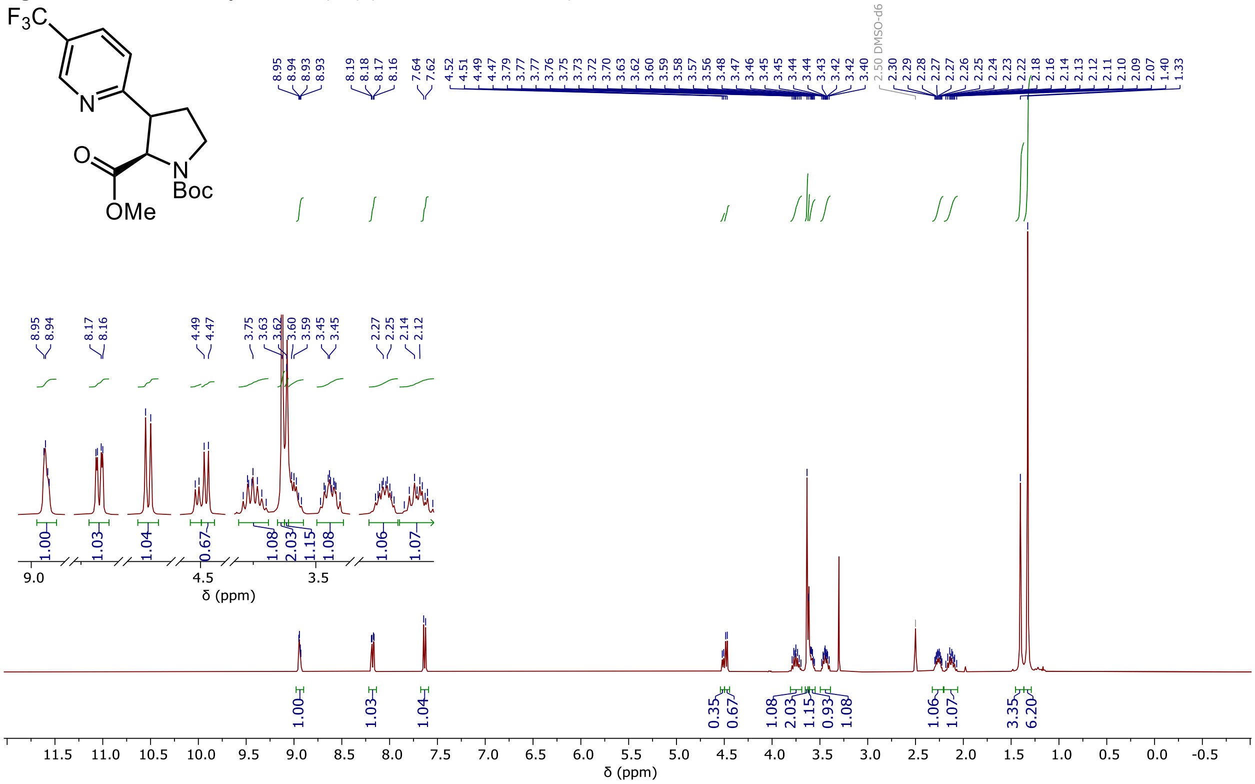


**Figure S168.** $^{1}$**H NMR spectra of (45) (400 MHz, DMSO-d$_6$).**

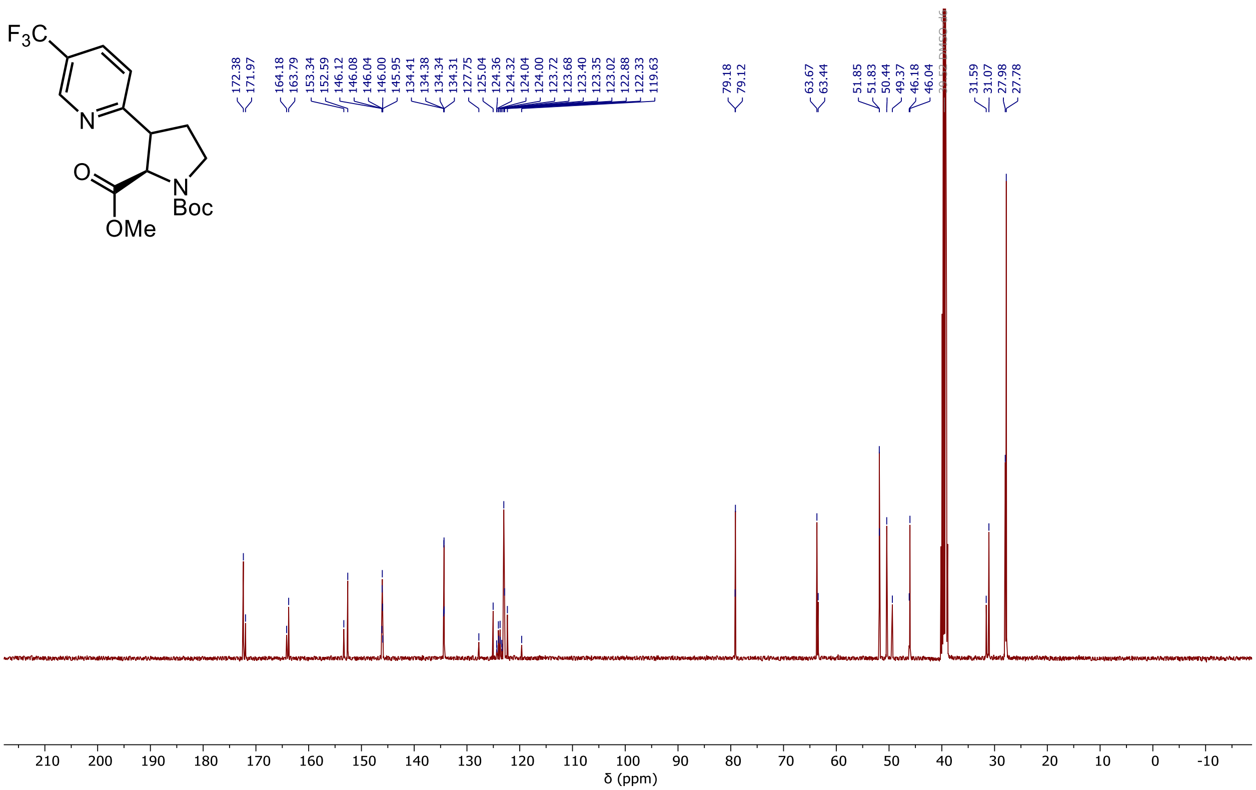


**Figure S169. $^{13}$C NMR spectra of (45) (101 MHz, DMSO-d$_6$).**

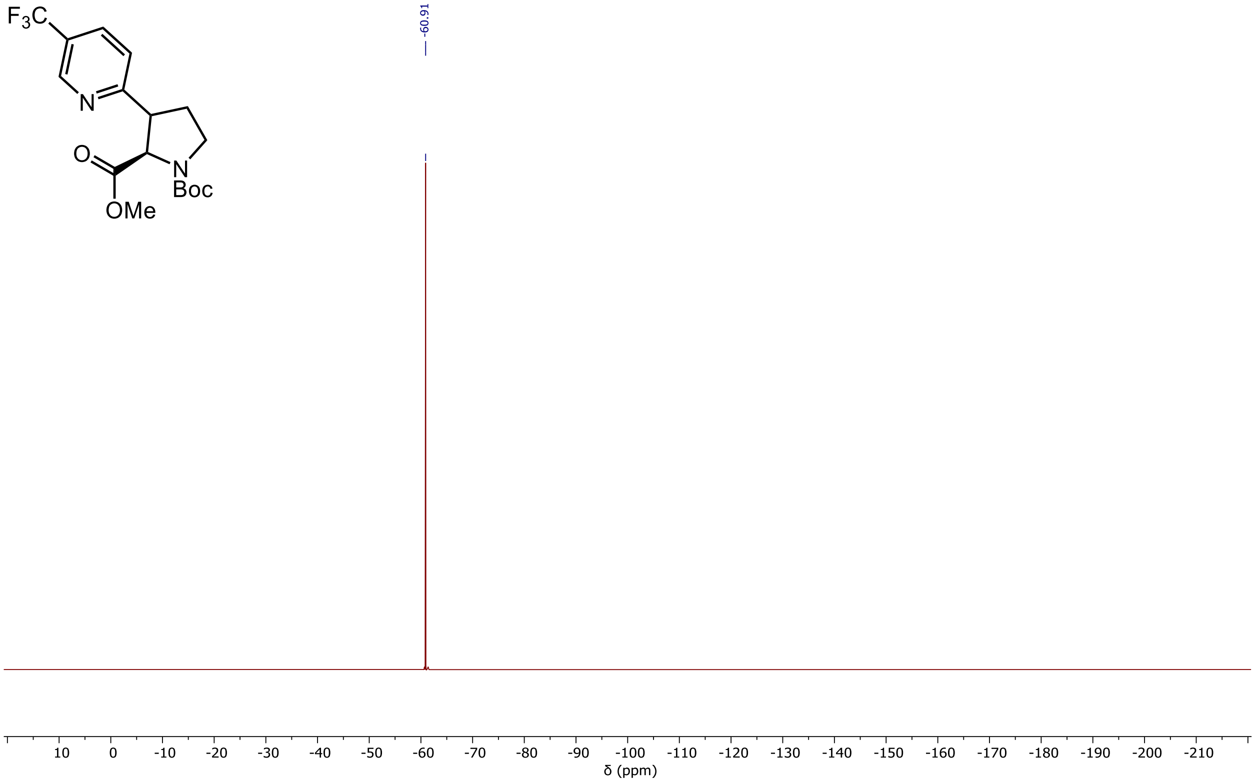


**Figure S170. $^{19}$F NMR spectra of (45) (376 MHz, DMSO-d$_6$).**

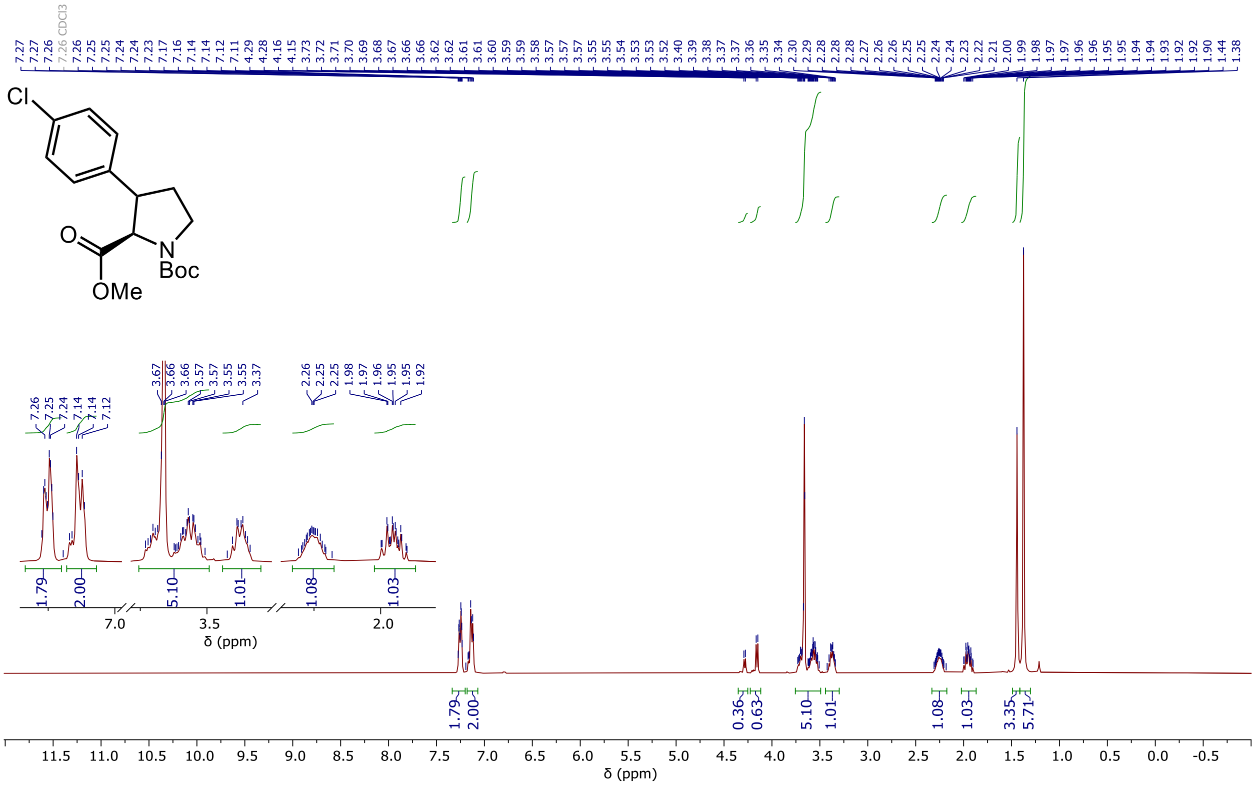

**Figure S171.** $^{1}$**H NMR spectra of (46) (400 MHz, DMSO-d$_6$).**

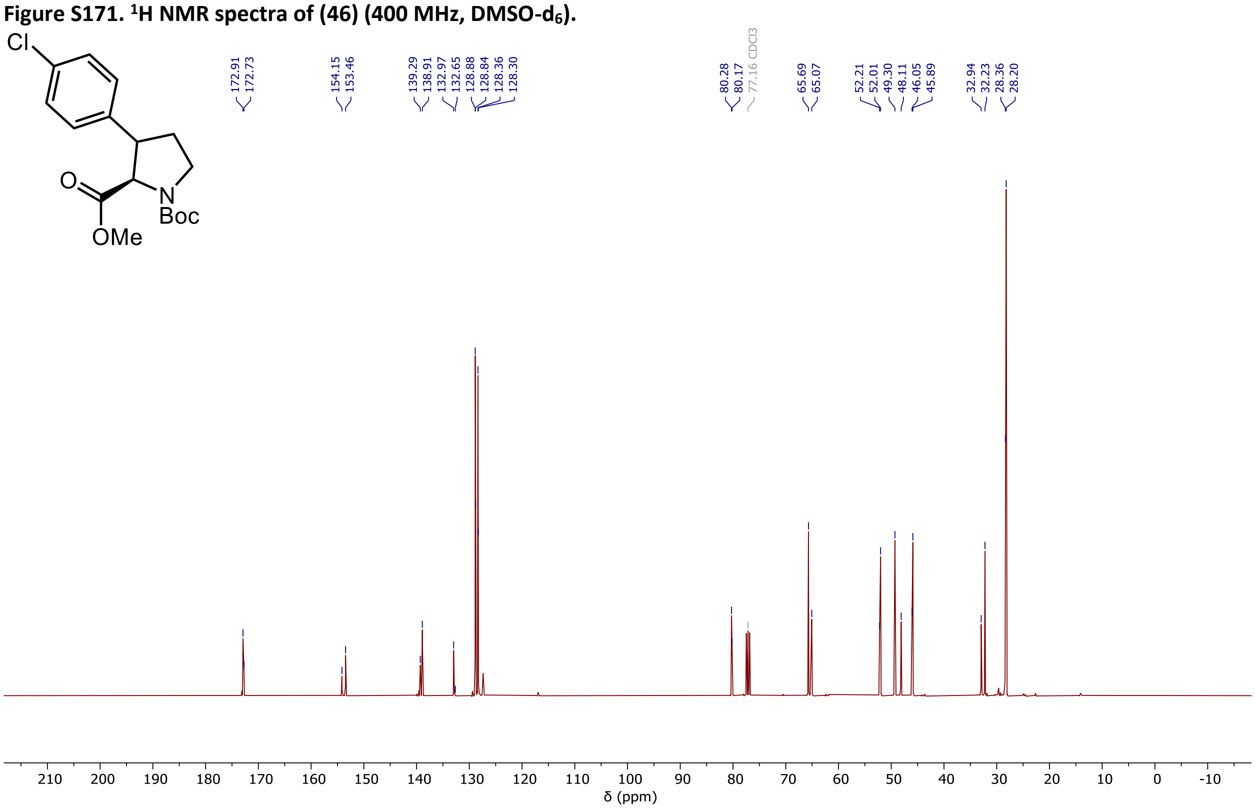

**Figure S172.** $^{13}$**C NMR spectra of (46) (101 MHz, DMSO-d$_6$).**
